# How Well Can Multivariate and Univariate GWAS Distinguish Between True and Spurious Pleiotropy?

**DOI:** 10.3389/fgene.2020.602526

**Published:** 2021-01-08

**Authors:** Samuel B. Fernandes, Kevin S. Zhang, Tiffany M. Jamann, Alexander E. Lipka

**Affiliations:** Department of Crop Science, University of Illinois at Urbana-Champaign, Urbana, IL, United States

**Keywords:** Simulation, multi-trait, Unified Mixed-Model, QTN, maize, soybean, LD

## Abstract

Quantification of the simultaneous contributions of loci to multiple traits, a phenomenon called pleiotropy, is facilitated by the increased availability of high-throughput genotypic and phenotypic data. To understand the prevalence and nature of pleiotropy, the ability of multivariate and univariate genome-wide association study (GWAS) models to distinguish between pleiotropic and non-pleiotropic loci in linkage disequilibrium (LD) first needs to be evaluated. Therefore, we used publicly available maize and soybean genotypic data to simulate multiple pairs of traits that were either (i) controlled by quantitative trait nucleotides (QTNs) on separate chromosomes, (ii) controlled by QTNs in various degrees of LD with each other, or (iii) controlled by a single pleiotropic QTN. We showed that multivariate GWAS could not distinguish between QTNs in LD and a single pleiotropic QTN. In contrast, a unique QTN detection rate pattern was observed for univariate GWAS whenever the simulated QTNs were in high LD or pleiotropic. Collectively, these results suggest that multivariate and univariate GWAS should both be used to infer whether or not causal mutations underlying peak GWAS associations are pleiotropic. Therefore, we recommend that future studies use a combination of multivariate and univariate GWAS models, as both models could be useful for identifying and narrowing down candidate loci with potential pleiotropic effects for downstream biological experiments.

## 1. Introduction

The number of traits available from state-of-the-art phenotyping techniques typically exceeds the number of genes in many species' genomes. For instance, the human genome contains over 20, 000 genes (Wagner and Zhang, 2011), but the Human Metabolome Database (Wishart et al., 2007) alone has collected more than 114, 000 metabolite traits. A direct consequence is that many genes likely control more than one of these traits, a phenomenon known as pleiotropy (Visscher and Yang, 2016). The identification and characterization of this phenomenon has been the subject of extensive research in the 100+ years following the first attributed use of the term “pleiotropy” in Platt (1910) Stearns (2010). Examples of important genes with pleiotropic effects in plant science include *Lg1* and its contribution to inflorescence and leaf traits in maize (Foster et al., 2004; Lewis et al., 2014) and multiple disease resistance attributed to *GH3-2* in rice (Fu et al., 2011) and *Lr67* in wheat (Moore et al., 2015). With the recent acquisition of high-throughput phenotype and genotype data, it is now possible to directly identify pleiotropic causal mutations (Wagner and Zhang, 2011). The abundance of such high-throughput data in conjunction with a plethora of tools available for quantifying genotype-to-phenotype associations (Marchini et al., 2007; Purcell et al., 2007; Lipka et al., 2012; Zhou and Stephens, 2014) is providing increasing evidence for pleiotropic genes involved in evolution (Smith, 2016; Auge et al., 2019), disease resistance (Wisser et al., 2011; Lopez-Zuniga et al., 2019; Qiu et al., 2020), yield (Ward et al., 2019), and many other traits (Jiang et al., 2019; Rice et al., 2020). These analyses have also led to opposing views for (Boyle et al., 2017) and against (Wray et al., 2018) the ubiquitousness of pleiotropy in complex trait variation, particularly in the form of the omnigenic model. This model assumes that the same set of small-effect regulatory genes explain the vast majority of complex disease resistance traits expressed in a disease-relevant cell (Boyle et al., 2017).

One of the most commonly used approaches for quantifying genotype-to-phenotype relationships is the genome-wide association study (GWAS), which has been used to investigate pleiotropy (Wisser et al., 2011; Schaid et al., 2016; Rice et al., 2020). However, a significant drawback of a GWAS is that most of the markers available in typical high-throughput genotypic data are not causal. Instead, they are in imperfect linkage disequilibrium (LD) with the causal mutations of a given trait. This LD obfuscates the ability to distinguish a single pleiotropic causal mutation underlying multiple traits from multiple non-pleiotropic causal mutations in LD with each other (Gianola et al., 2015). Furthermore, it would only be possible to differentiate between a set of multiple non-pleiotropic causal mutations and one pleiotropic causal mutation if the former were in imperfect LD (Kemper et al., 2018). The scenario of tightly-linked non-pleiotropic causal mutations being mistaken for one pleiotropic causal mutation is known as spurious pleiotropy (Solovieff et al., 2013; van Rheenen et al., 2019). In addition to hindering the characterization of biological processes underlying trait variability, the presence of spurious pleiotropy in GWAS results could have serious negative downstream breeding ramifications (Chen and Lübberstedt, 2010). For instance, if two separate causal mutations in LD with antagonistic effects each control one of two correlated traits, breeders could allocate resources toward increasing population size to find individuals with recombination between these causal mutations (Schulthess et al., 2017). However, if a set of GWAS results are misinterpreted as suggesting that one pleiotropic causal mutation is present (i.e., the scenario of spurious pleiotropy is realized), then such efforts to increase the population size may never be undertaken.

Many studies use the term cross-phenotype to refer to markers with strong statistical associations with multiple traits (Tyler et al., 2016). Several univariate and multivariate GWAS approaches have been implemented to detect cross-phenotype associations (Zhou and Stephens, 2014; Cichonska et al., 2016; Joo et al., 2016), with multi-trait models shown to be optimum under many circumstances (Yang and Wang, 2012; Porter and O'Reilly, 2017; Melo et al., 2019; Pitchers et al., 2019; Rice et al., 2020). Although there is great value in detecting cross-phenotype associations, there is still a critical need to distinguish whether the underlying causal mutation(s) are pleiotropic or are non-pleiotropic but in strong LD. We hypothesized that one of the major reasons underlying the difficulty in distinguishing between these two scenarios is that the most widely-used univariate and multivariate GWAS models are insufficient for making such a distinction. Therefore, we used publicly available maize and soybean genotypic data to simulate pairs of correlated traits that were either (i) controlled by non-pleiotropic quantitative trait nucleotides (QTNs) on separate chromosomes, (ii) controlled by non-pleiotropic QTNs in various degrees of LD with each other, or (iii) controlled by a single pleiotropic QTN. We then assessed the ability of state-of-the-art univariate and multivariate GWAS models to identify these QTNs. We predicted that as the amount of LD between the non-pleiotropic QTNs increased, the multivariate GWAS results would more closely resemble those from traits controlled by a single pleiotropic QTN.

## 2. Materials and Methods

### 2.1. Maize and Soybean Data

In this study, we used publicly available molecular marker data from two crop species, specifically maize (*Zea mays* L.) and soybean (*Glycine max* L.). These two species were selected because of their contrasting rates of LD decay; while soybean tends to have long-range LD (Hyten et al., 2007; Zhang et al., 2015), more rapid LD decay is typically observed in maize (Gore et al., 2009; Romay et al., 2013). The maize data were comprised of 2, 815 accessions from the North Central Regional Plant Introduction Station (NCRPIS) panel (Romay et al., 2013), while the soybean data consisted of a random sample of 2, 815 accessions in maturity groups III and IV from SoyBase (Song et al., 2015). To investigate the impact of sample size on the results, for each data set, we considered the full set of *S*_1_ = 2, 815 accessions, a subsample of *S*_2_ = 1, 000 accessions, and a subsample of *S*_3_ = 500 individuals. The accessions of *S*_3_ were randomly sampled from *S*_2_, whereas the accessions of *S*_2_ were randomly sampled from *S*_1_, i.e., *S*_3_ ⊂ *S*_2_ ⊂ *S*_1_. All subsamples were obtained using the (*vcftools --max-indv*) command in vcftools (Danecek et al., 2011). Details on how to access the datasets are provided in the Supplementary Material.

The maize data included 681, 257 single-nucleotide polymorphisms (SNPs) obtained through genotyping-by-sequencing (Romay et al., 2013), available at http://cbsusrv04.tc.cornell.edu/users/panzea/download.aspx?filegroupid=6. The soybean data were downloaded from SoyBase (Song et al., 2015) at http://soybase.org/snps/download.php, and consisted of 42, 291 SNPs obtained with the SoySNP50K (Song et al., 2013). The same filters were applied to both datasets using vcftools. These filters included removing all SNPs with more than 5% missing data. Additionally, Plink was used to conduct LD pruning, where the LD parameter was set to *r*^2^ = 0.9 (*--indep-pairwise 100 10 0.9*) (Purcell et al., 2007). Thus, only markers that were in an LD of *r*^2^ ≥ 0.9 were filtered out. Only SNPs from chromosomal DNA that passed the minor allele count threshold of 5 in *S*_3_ were included in this simulation study. Consequently, the final data sets used for simulation were 44, 930 SNPs for maize, and 18, 364 for soybean.

### 2.2. Trait Simulation

**Figure 1:**
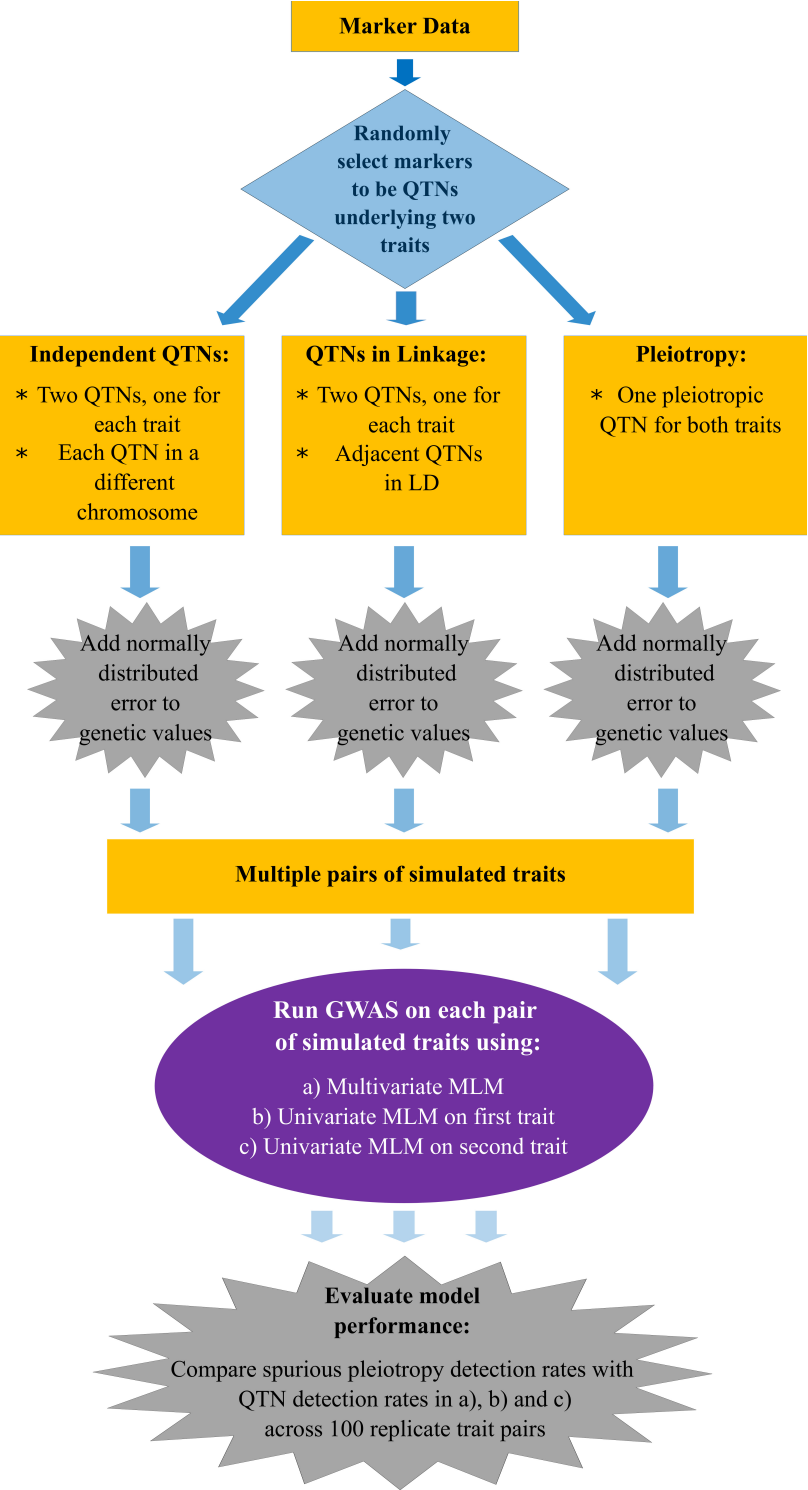
Flow chart with the methods used to simulate, conduct a genome-wide association study (GWAS), and detect quantitative trait nucleotides (QTNs) in multiple traits.

The flow chart presented in Figure 1 summarizes the main aspects of the simulation study we conducted. In brief, we simulated pairs of traits controlled by either pleiotropic or non-pleiotropic QTNs. Each pair of traits was simulated with the simplePHENOTYPES (Fernandes and Lipka, 2020) package in the R software (R Core Team, 2020). We were specifically interested in comparing and contrasting the behavior of single peak-associated SNPs from GWAS, similar in magnitude to those reported in Rice et al. (2020), over multiple simulation replicates. Thus, all individual traits were controlled by exactly one additive QTN selected from either the maize or soybean marker data. For each pair of replicate traits, a maximum of two QTNs were selected. To investigate the impact of LD between two non-pleiotropic QTNs on the GWAS results, we sampled QTNs in three different scenarios. First, the QTNs were sampled from different chromosomes (called “Independent QTNs” in Figure 1). Such a configuration of QTNs was achieved by simulating trait pairs independently in simplePHENOTYPES. For a given set of input parameter values (Table 1), this process was repeated until 100 replicate trait pairs, with each trait in a pair controlled by a QTN on a different chromosome, were obtained. Next, we simulated trait pairs where the maximum amount of LD between the two linked non-pleiotropic QTNs controlling each trait was specified (called “QTNs in linkage” in Figure 1). We simulated this configuration in simplePHENOTYPES by specifying *architecture* = “*LD*,” and indicated the amount of maximum desired LD between pairs of selected SNPs through the *ld* input parameter. We controlled this amount of LD both directly (i.e., the LD between the two QTNs) and indirectly (the LD between each QTN and a marker located in between). Finally, we simulated pairs of correlated traits that were controlled by a single pleiotropic QTN (called “Pleiotropy” in Figure 1). This configuration was specified in simplePHENOTYPES by *architecture* = “*pleiotropic*.”

**Table 1:** Description of the input parameter values considered to simulate each pair of traits in the simulation study.

			***h***^2^^***d***^			
**QTN selection^a^**	**Type of LD^b^**	**MAF^c^**	**Trait 1**	**Trait 2**	**Sample size**	**Species**
QTNs independently selected	Direct	0.05	0.30	0.30	500	Maize
LD controlled at < 0.01	Indirect	0.40	0.30	0.80	1,000	Soybean
LD controlled at < 0.98			0.80	0.80	2,815	
One pleiotropic QTN						

*Each combination of input parameter values resulted in 216 simulation scenarios*.

a *QTN, quantitative trait nucleotide*.

b *LD, linkage disequilibirum (r^2^)*.

c *MAF, minor allele frequency*.

d *h^2^, narrow-sense heritability*.

Table 1 provides a summary of the input parameters considered in the simulation study. Briefly, three configurations of narrow-sense heritability (*h*^2^) were simulated: two with the same *h*^2^ for both traits and one with a different *h*^2^ for each trait. The latter configuration is a common situation in breeding programs, where a trait of interest with small heritability is correlated to a trait of less interest but with a higher *h*^2^ (Fernandes et al., 2018). Because a single QTN controlled each trait, and the non-genetic variance was a function of the inputted heritability, the additive effect size of every QTN in this study was set to the same value, namely 0.10. To evaluate the impact of rare vs. common variants on the results, we also considered the minor allele frequencies (MAFs) of the selected markers as an input parameter (see Table 2 for details). Altogether, we simulated 216 scenarios, where each scenario consisted of a unique combination of input parameters. Each scenario was replicated 100 times using the option *vary*_*QTN* = *TRUE* in simplePHENOTYPES, meaning that a different pair of QTNs were selected for each replicate.

**Table 2:** Description of how minor allele frequency (MAF) was controlled in the simulation study.

**QTN^*a*^ configuration**	**MAF control**
QTNs independently selected	Both QTNs selected based on MAF
LD^*b*^ between QTNs directly controlled	QTN for first trait selected based on MAF
LD between QTNs indirectly controlled	Common marker located between QTNs selected based on MAF
One pleiotropic QTN	Pleiotropic QTN selected based on MAF

a *QTN, quantitative trait nucleotide*.

b *LD, linkage disequilibirum*.

We used simplePHENOTYPES' option “*remove*_*QTN* = *TRUE*” to simulate the frequently occurring scenario of the causal mutations not being included in the marker sets. Thus, for each of the 100 replicate trait pairs evaluated at a given scenario, the marker data were saved without the SNPs used as QTNs. Accordingly, for all traits, we conducted GWAS on all markers except the one selected to be the QTN.

### 2.3. Genome-Wide Association Studies

Multivariate and univariate GWAS was conducted on all simulated traits. For each replicate trait pair, we used the multivariate version of the unified mixed linear model (MLM) (Yu et al., 2006) implemented in GEMMA (Zhou and Stephens, 2014) to conduct the multivariate GWAS. In this analysis, a given replicate trait pair was included in this model as the multivariate response variable. The multivariate MLM was fitted in GEMMA using the commands (“*gemma --bfile bed_file -lmm 2 -miss 0.001 -maf 0.001 -r2 0.999999 -n 1 2 -k kinship.txt -o output”*), with the kinship matrix (VanRaden, 2008) calculated with the AGHmatrix R package (Amadeu et al., 2016). Similarly, for each of the two simulated traits contributing to a replicate trait pair, an analogous univariate unified MLM was fitted in the GEMMA software using all of the same commands except for *-n 1*. No fixed-effect covariates accounting for subpopulation structure were included in any GWAS model because (i) subpopulation structure did not explicitly contribute to the variability of the simulated traits, and (ii) all QTNs were randomly sampled irrespective of the degree to which their alleles segregated by subpopulations.

### 2.4. QTN Detection Rate for Univariate and Multivariate GWAS

For each simulation scenario, we compared the proportion of 100 replicate trait pairs in which the multivariate MLM identified a signal in the vicinity of the QTN(s) and the proportion in which the univariate MLM identified a signal in the vicinity of the QTN controlling the tested trait. We applied the Benjamini and Hochberg (1995) procedure to control the genome-wide false discovery rates (FDR) at 10%, and 5% for each model ran on each replicate trait pair. A SNP-trait association passing this threshold was deemed to be in the vicinity of a given QTN if it was within 10 kb (in maize) or 1 Mb (in soybean) of the QTN. These physical window sizes roughly correspond to a pairwise LD decay of *r*^2^ = 0.10 in both species (Supplementary Figures 1, 2). To compare the influence of window sizes on the results, we also considered window sizes of 1 kb in maize and 10 kb in soybean; these results are presented in Supplementary Figures 20–29, 40–49.

For a given replicate trait pair, the multivariate MLM (which tested *H*_0_: No association between the tested SNP and any trait in the multivariate model) was said to have identified a QTN if at least one SNP with an FDR-adjusted *P*-value <0.10 (or 0.05 when the FDR was controlled at 5%) was located within the surrounding physical window. Similarly, for a given trait in a replicate trait pair, the univariate MLM (which tested *H*_0_: No association between the tested SNP and the trait in the univariate model) was said to have correctly identified the QTN underlying that trait if at least one SNP with an FDR-adjusted *P*-value <0.10 (or 0.05) was located within the physical window of that QTN. Thus, across the 100 replicate trait pairs simulated at each setting, we recorded the following percentages:

1. The percentage of replicate trait pairs where a given GWAS model identified the QTN underlying the first trait.
2. The percentage of replicate trait pairs where a given GWAS model identified the QTN underlying the second trait.
3. The percentage of replicate trait pairs where a given GWAS model identified both QTNs underlying both traits.
4. The percentage of replicate trait pairs where a given GWAS model identified at least one statistically significantly associated marker outside of both windows for both traits.

When these percentages 1–3 were calculated for the multivariate GWAS under the “Independent QTNs” and “QTNs in linkage” scenario, they were referred to as the spurious pleiotropy detection rate. Otherwise, these proportions were called QTN detection rates. For both multivariate and univariate GWAS, the percentages calculated in 4 were called the error rate. Finally, as a measure of regional LD, i.e., LD in the region surrounding the selected QTN, we calculated the LD (*r*^2^) between the selected QTN and the 20 SNPs upstream and the 20 SNPs downstream.

## 3. Results

In general, the results were similar across sample sizes and heritabilities. Unless noted otherwise, we highlight below the findings at the relatively moderate sample size of 1, 000 individuals, heritability of trait pairs set to *h*^2^ = (0.30, 0.80), 10% FDR and window size of 10 kb for maize and 1 Mb for soybean. We chose to present these particular heritabilities because of the aforementioned interest in correlated traits with contrasting heritabilities among breeders (Fernandes et al., 2018). For completeness, results for the remaining sample sizes and heritabilities are included in the Supplementary Material.

### 3.1. Observed MAFs Were Similar to User-Inputted Values, but Observed LD Was Lower

**Figure 2:**
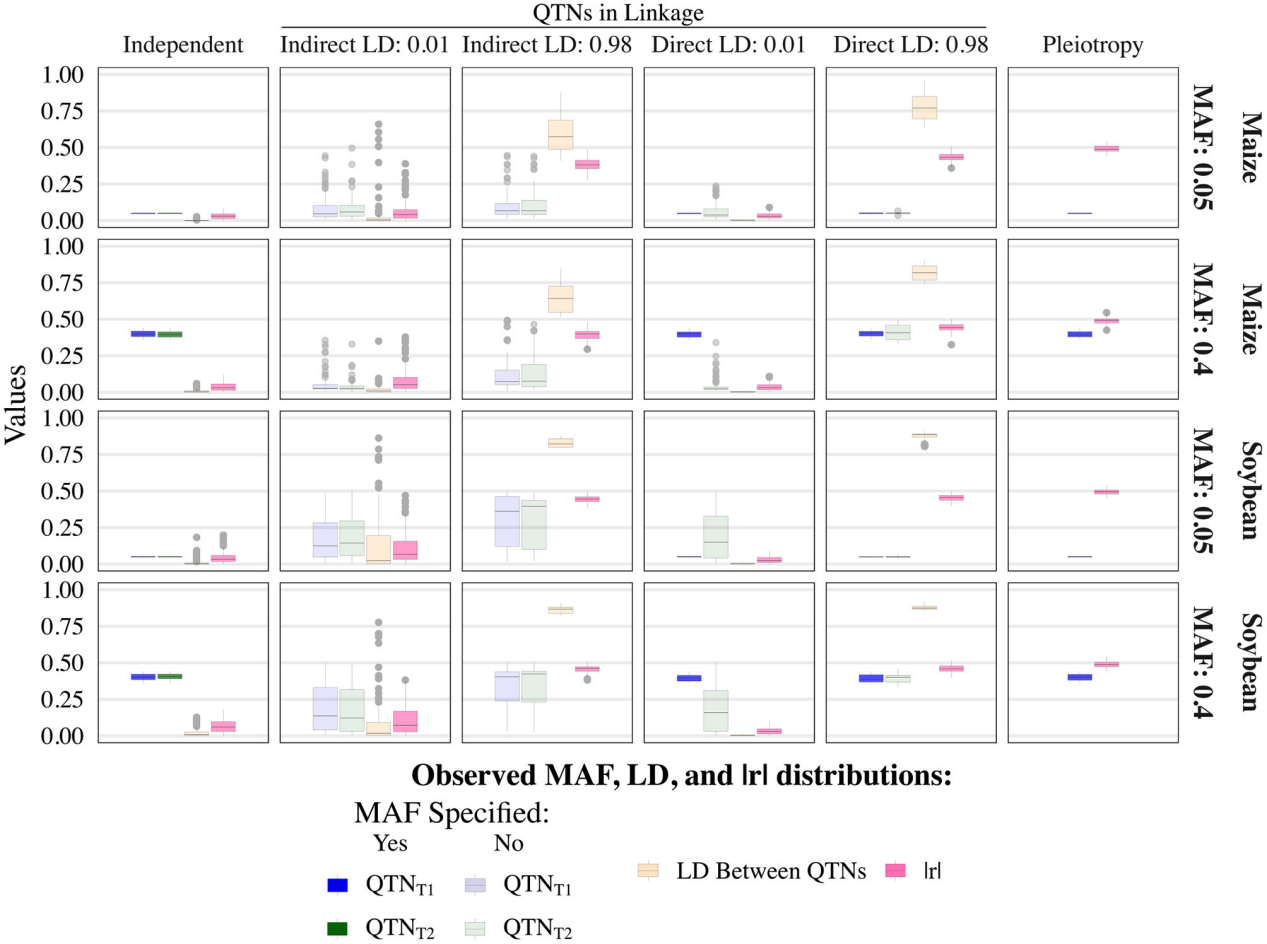
Observed minor allele frequencies (MAF) for quantitative trait nucleotides (QTN) controlling trait 1 (QTN_T1_) and trait 2 (QTN_T2_), and the observed linkage disequilibrium (LD) between them, measured as *r*^2^, for the sample size of 1, 000 and narrow-sense heritability of 0.3 and 0.8, for traits 1 and 2, respectively. Darker colors indicate QTNs that had MAF directly controlled by an input parameter of the simulation, whereas lighter colors indicate QTNs where MAF was not controlled. The simulated genetic architecture is listed in the horizontal and vertical titles.

The various user-inputted parameters in simplePHENOYPTES enabled control of the MAFs of QTNs, as well as the LD between non-pleiotropic QTNs, to a certain extent. For QTNs where we specified the MAFs as an input parameter (indicated by a darker color in Figure 2; Supplementary Figures 6–9), the observed MAF distributions were similar to the user-inputted values. For QTNs where the MAFs were not directly controlled as an input parameter (indicated by a lighter color in Figure 2), most observed MAFs tended to be lower in maize than in soybean.

As expected, the observed LD between non-pleiotropic QTN pairs tended to be higher in soybean than in maize, although outlying instances of similar levels of high LD were observed in maize (Figure 2; Supplementary Figures 6–9). Surprisingly, the distribution of LD between non-pleiotropic QTN under the independent QTNs scenario yielded outlying LD values greater than what was observed under the direct control of LD at *r*^2^ = 0.01. Because each pair of independent QTNs were simulated on separate chromosomes, we attribute these outlying values to interchromosomal LD. Thus, these simulated traits yielded pairs of non-pleiotropic QTNs with contrasting levels of LD between each other, enabling a thorough evaluation of the performance of univariate and multivariate GWAS models.

### 3.2. QTN and Spurious Pleiotropy Detection Rates Varied Across Sample Sizes, Heritabilities and QTN MAFs

**Figure 3:**
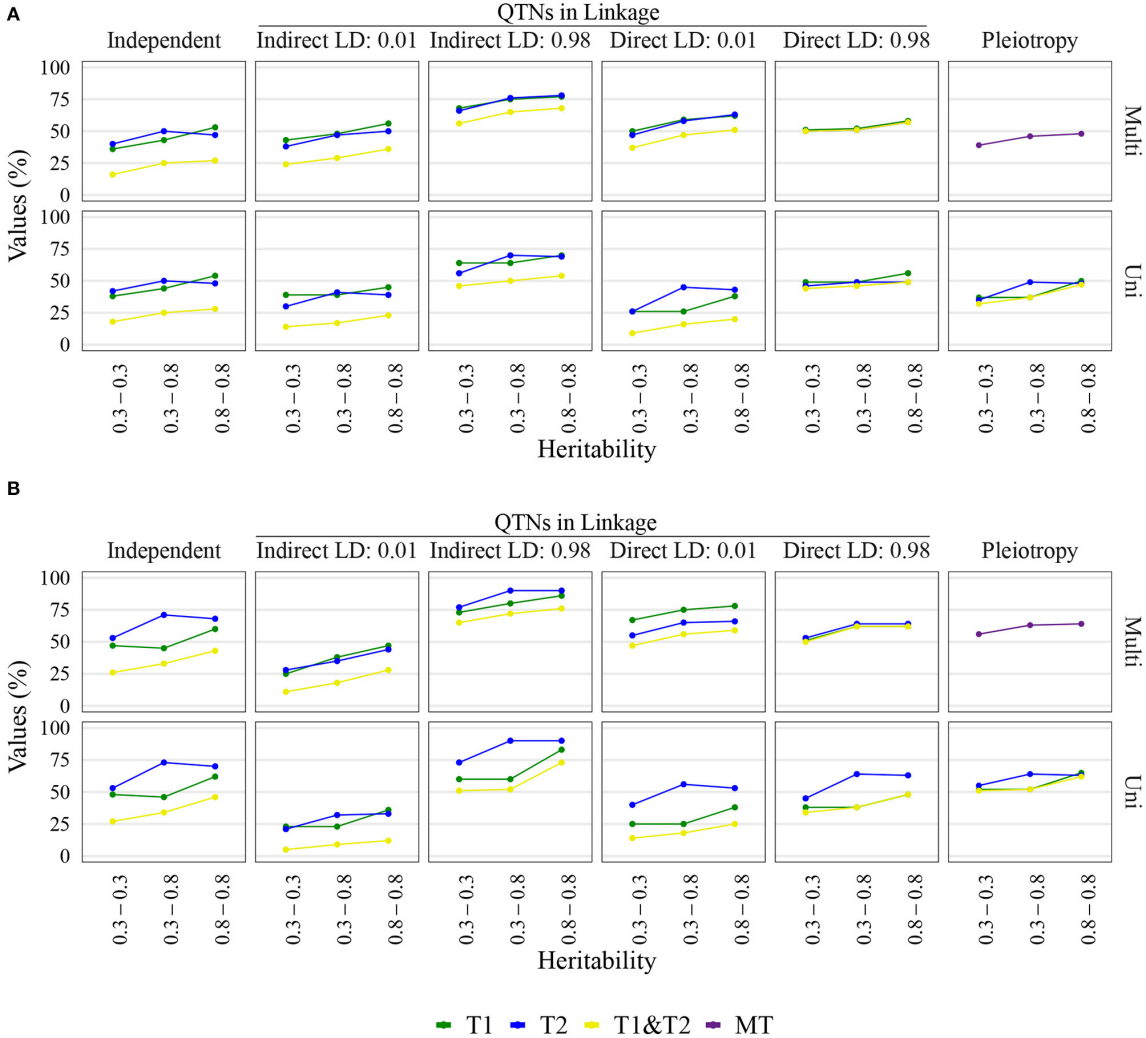
Quantitative trait nucleotide (QTN) and spurious pleiotropy detection rate (Y-axis) achieved by multivariate (Multi) and univariate (Uni) GWAS, relative to the QTN controlling trait 1 (T1), trait 2 (T2), and both QTN simultaneously (T1&T2) or, in the pleiotropic scenario, relative to the pleiotropic QTN (MT). These values were obtained for maize with a sample size of 1, 000. The X-axis displays the narrow-sense heritability for Trait 1 (bottom value) and Trait 2 (top value). **(A)** Inputted minor allele frequency (MAF) of 0.05; **(B)** MAF of 0.4.

**Figure 4:**
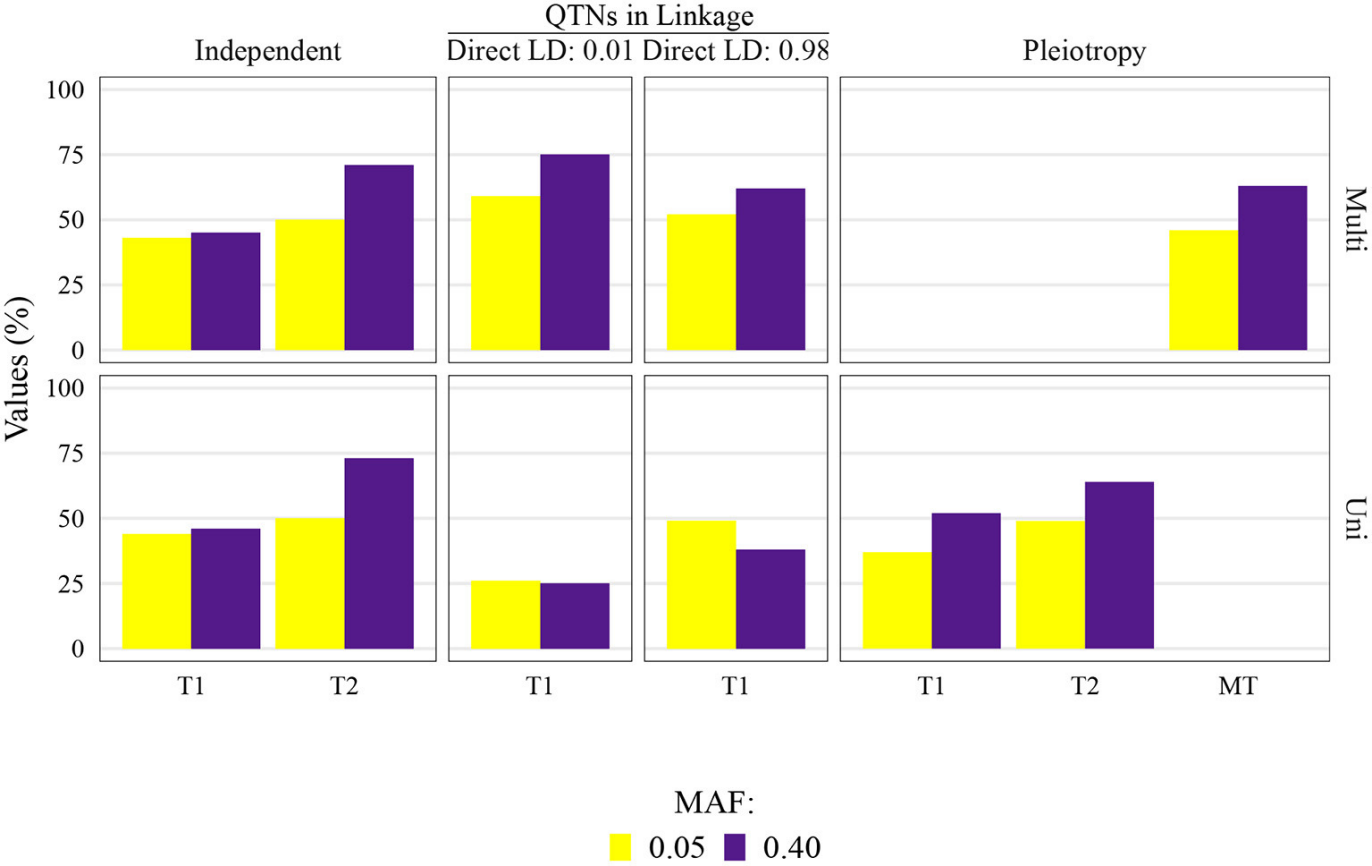
Quantitative trait nucleotide (QTN) and spurious pleiotropy detection rate (Y-axis) in scenarios for which minor allele frequency (MAF) was directly controlled by a simulation input parameter. These values were obtained by multivariate (Multi) and univariate (Uni) GWAS, relative to the QTN controlling trait 1 (T1), and trait 2 (T2), or in the pleiotropic scenario, relative to the pleiotropic QTN (MT). This figure shows results for maize with a sample size of 1, 000, and a narrow-sense heritability of 0.3 and 0.8, for Trait 1 and Trait 2, respectively.

**Figure 5:**
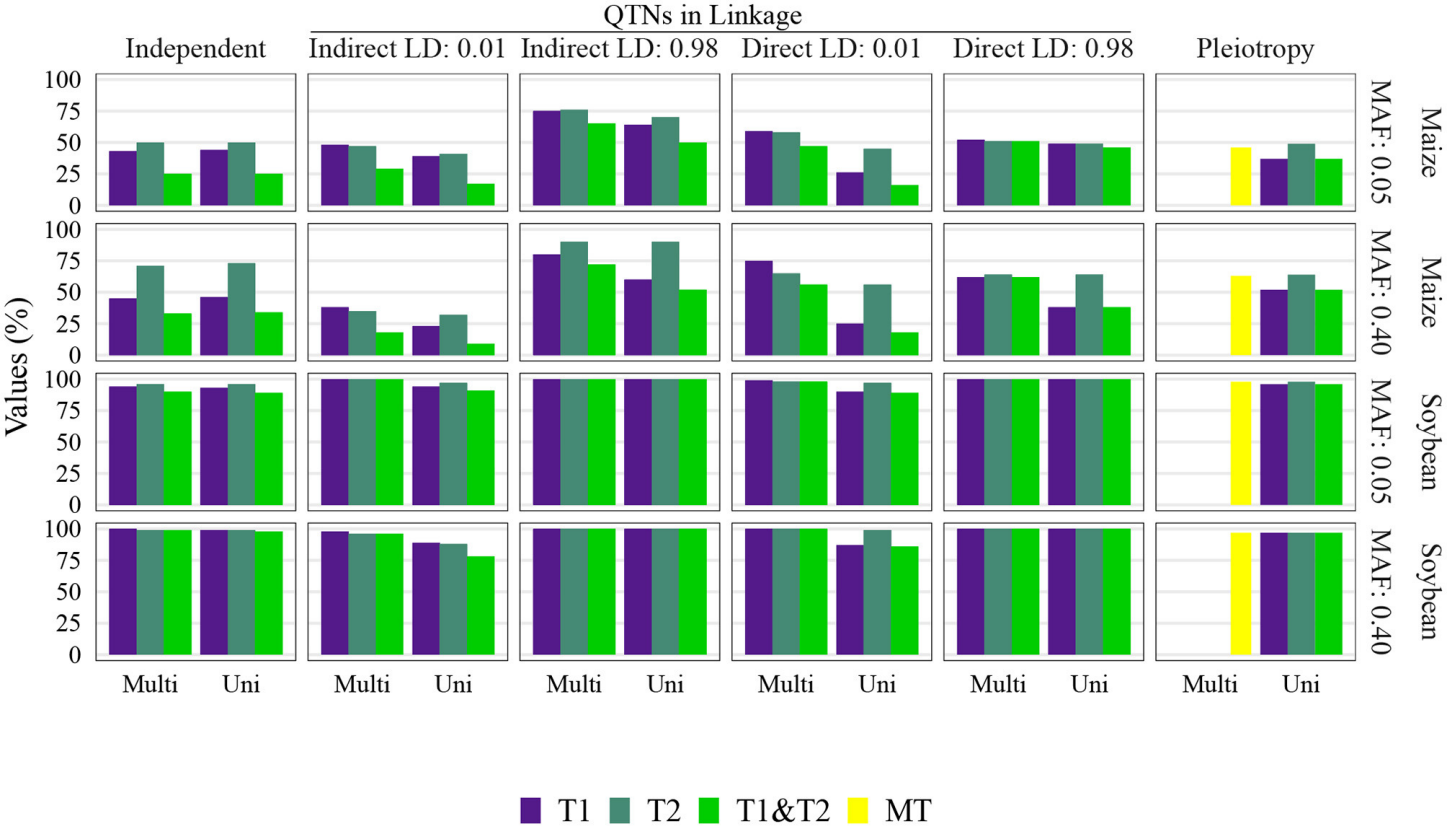
Quantitative trait nucleotide (QTN) and spurious pleiotropy detection rate (Y-axis) achieved by multivariate (Multi) and univariate (Uni) GWAS (X-axis), relative to the QTN controlling trait 1 (T1), trait 2 (T2), and both QTN simultaneously (T1&T2) or, in the pleiotropic scenario, relative to the pleiotropic QTN (MT). The simulated genetic architecture is listed in the horizontal and vertical titles. These values were obtained with a sample size of 1, 000, and a narrow-sense heritability of 0.3 and 0.8, for traits 1 and 2, respectively. MAF, minor allele frequencies.

The QTN and spurious pleiotropy detection rates generally increased as the sample size increased (Supplementary Figures 10–12, 20–22, 30–32, 40–42). Similarly, these rates increased monotonically as the heritabilities increased (Figure 3, Supplementary Figures 10–12, 20–22, 30–32, 40–42). The overall high QTN and spurious pleiotropy detection rates in soybean precluded the discernment of any notable trends in the GWAS approaches' performance across the observed MAFs (Supplementary Figures 10–12, 20–22, 30–32, 40–42). However, in maize, we noted that for most settings, higher QTN and spurious pleiotropy detection rates tended to be observed for QTNs where the MAFs were specified to be around 0.40 instead of 0.05 (Figure 4). In general, all the conclusions were similar when varying the FDR and window size. The largest difference in this regard was noted in soybean, specifically in that a considerably higher QTN and spurious pleiotropy detection rate was noted whenever the multiple testing was adjusted at 10% FDR and the window size was 1 Mb (Figure 5; Supplementary Figures 16–19).

### 3.3. Observed Multivariate GWAS Performance for Non-pleiotropic QTNs in Linkage and a Single Pleiotropic QTN

The multivariate GWAS results are presented in their entirety in Figure 5 and Supplementary Figures 16–19, 26–29, 36–39, 46–49). In general, high spurious pleiotropy detection rates were observed under the “QTNs in Linkage” scenario. Specifically, for QTNs that were in high LD, we observed that the multivariate GWAS spurious pleiotropy detection rate of both QTNs (depicted as the light green bar in Figure 5) tended to be similar to or greater than the multivariate GWAS detection rate of the pleiotropic QTNs (yellow bar in Figure 5). Interestingly, we also noted a trend in the ability of multivariate GWAS to identify each individual non-pleiotropic QTN in LD (depicted as the purple and blue-green bars in Figure 5). That is, with the exception of indirect LD of 0.01, we observed that all individual multivariate GWAS spurious pleiotropy detection rates were higher than the corresponding multivariate GWAS detection rates on the pleiotropy scenario. The pattern of error rate, i.e., significant markers detected outside the predefined window size, was similar to the QTN and spurious pleiotropy detection rate (Supplementary Figures 50–89). The only notably different result when considering the error rate was observed in the independent QTNs scenario, where it resulted in a reduced error rate compared to the other genetic architectures.

### 3.4. Univariate GWAS Displayed Distinct Detection Patterns for Non-pleiotropic QTNs in High LD and Single Pleiotropic QTNs

Univariate GWAS tended to yield distinct patterns of QTN detection under both (i) high LD between non-pleiotropic QTNs and (ii) pleiotropy (depicted as the two rightmost columns of Figure 5; Supplementary Figures 16–19, 26–29, 36–39, 46–49). Specifically, the simultaneous detection rate of the QTNs for both traits (depicted as the green bars in Figure 5) tended to be relatively similar to the individual QTN detection rates for each trait (depicted as the purple and blue-green bars Figure 5). For the remaining scenarios where non-pleiotropic QTN were simulated (presented in the four leftmost columns of Figure 5), we contrastingly observed that the simultaneous detection rate of each pair of non-pleiotropic QTNs tended to be less similar to the individual QTN detection rates. These results suggest that univariate GWAS could be extremely useful for distinguishing between a single pleiotropic QTN and two or more non-pleiotropic QTNs in linkage. In the scenarios of high LD, the SNPs selected to be QTNs were located in regions of slightly higher LD (Supplementary Figures 3–5). Consequently, the univariate QTN detection rate was slightly higher in the instances where QTNs in LD were simulated. In most cases, the error rate was similar across different settings.

## 4. Discussion

The full potential of GWAS to contribute to the identification of pleiotropy will not be realized until its ability to distinguish between a single pleiotropic causal mutation and multiple non-pleiotropic causal mutations in LD is scrutinized in real genomic data. Therefore, we used publicly available maize and soybean marker data to conduct a simulation study that quantified the QTN and spurious pleiotropy detection rates of both pleiotropic and non-pleiotropic QTNs for two widely-used statistical models in plant GWAS. We specifically used the univariate and multivariate MLM and controlled for multiple testing at 10% FDR. Our results showed that even at surprisingly small LD between non-pleiotropic QTNs, the multivariate GWAS model tended to yield high spurious pleiotropy detection rates. Because of the high spurious pleiotropy detection rates we inferred that multivariate GWAS was unable to distinguish between a single pleiotropic QTN and two non-pleiotropic QTNs in LD. We also observed that for pleiotropic QTNs, the univariate GWAS model's simultaneous QTN detection rates for both traits were similar to the QTN detection rates for the individual traits; such a degree of similarity was observed only at non-pleiotropic QTNs pairs in the highest amount of pairwise LD that we specified in our simulation parameters. Collectively, these results suggest that the univariate GWAS model might be useful in conjunction with multivariate GWAS model for distinguishing between true and spurious pleiotropy.

### 4.1. High Spurious Pleiotropy Detection Rates From Multivariate GWAS Were Observed Under LD

The potential of multivariate GWAS models has been demonstrated in many studies (Galesloot et al., 2014; Zhou and Stephens, 2014; Pitchers et al., 2019; Rice et al., 2020). Our results agree with this previous work, as the observed ability of multivariate GWAS to identify QTNs was generally high for all scenarios particularly in soybean. The fact that the multivariate GWAS was able to detect non-pleiotropic QTNs is not surprising because the null hypothesis for most multivariate tests of association, including those used for the multivariate MLM, is *H*_0_: No association between the tested SNP and any trait (Schaid et al., 2016; Salinas et al., 2018). Thus, the multivariate MLM's detection of non-pleiotropic QTN, and more specifically spurious pleiotropy under the “QTNs in linkage” scenario, should not be regarded as false positives because these events technically occur in the alternative hypothesis. Nevertheless, the outcome of spurious pleiotropy underscores an intrinsic lack of resolution to distinguish between pleiotropic and non-pleiotropic QTNs.

The observed performance of multivariate GWAS at the various levels of LD between non-pleiotropic QTNs on the same chromosome was insightful. Although a previous study showed that multivariate GWAS could not distinguish between a single pleiotropic QTN and multiple non-pleiotropic QTNs in LD (Chebib and Guillaume, 2019), we expected that at low levels of LD between non-pleiotropic QTNs, the spurious pleiotropy detection rates would be similar to QTN detection rates under the scenario where non-pleiotropic QTNs were simulated on separate chromosomes. Furthermore, we predicted that as the amount of LD between the non-pleiotropic QTNs increased, the spurious pleiotropy detection rate of multivariate GWAS would become similar to the observed multivariate GWAS detection rate of a single pleiotropic QTN. Instead, we observed that even at LD levels of *r*^2^ < 0.01 between non-pleiotropic QTNs, the multivariate GWAS model yielded high spurious pleiotropy detection rates, a trend that was analogous to the QTN detection rates observed for traits controlled by one pleiotropic QTN. Interestingly, for the most stringent control of LD between non-pleiotropic QTNs on the same chromosome (i.e., *r*^2^ < 0.01), the maximum amount of observed LD was less than some outlying values of interchromosomal LD between non-pleiotropic QTNs simulated on separate chromosomes (Figure 2). These results were contrary to our prior expectations, and we consequently made two main conclusions. First, we confirmed that multivariate GWAS is a potentially useful tool for identifying causal mutations. Second, multivariate GWAS, particularly the multivariate unified MLM, alone is insufficient for distinguishing between multiple QTNs in LD and a single pleiotropic QTN, irrespective of the amount of LD between the QTNs.

### 4.2. Univariate GWAS Is Potentially Useful for Identifying Pleiotropy

One of the most useful findings from this study was the subtle differences in univariate GWAS QTN detection rates for both non-pleiotropic QTNs in high LD and pleiotropic QTNs. We hypothesize that if incorporated into standard GWAS analyses, these subtle differences could play a crucial role in inferring whether or not a certain set of GWAS results suggest pleiotropy. Although there is a critical need for future studies to investigate the most appropriate use of univariate GWAS in such a role, our results suggest two steps for using univariate GWAS for this purpose. First, a univariate GWAS could be conducted on each trait separately. Second, an *a posteriori* analysis could then be used to determine how frequently each univariate GWAS detects a signal. If a signal is consistently detected across several univariate analyses of individual traits, this could provide evidence that a pleiotropic causal mutation is underlying the signals detected from GWAS.

### 4.3. Considerations for Further Studies on the Ability of GWAS to Identify Loci Controlling Multiple Traits

Our findings build upon other studies (e.g., Chebib and Guillaume, 2019), indicating that caution should be used when interpreting multivariate GWAS results. Moreover, it highlights the usefulness of univariate GWAS in making conclusions regarding trait genetic architecture. However, some potential weaknesses of our study should be considered when designing future research. In particular, the inconsistent amount of local LD levels surrounding QTNs selected from different genetic architectures is a potential source of bias. We opted to consider a fixed window size when detecting the QTNs; this favors a comparison across different sample sizes, but because the LD will vary, so will the chance of detecting a QTN in that specific window.

In particular, when we simulated traits with QTNs in high LD “QTNs in Linkage” scenario, we observed that they were typically selected from genomic regions that contained at least one pair of SNPs in high LD. Thus, the local LD in these regions tended to be biased upwards. For the remaining simulation scenarios, the amount of local LD was not biased upwards, as can be seen in Supplementary Figures 3–5. We infer that these differences in local LD might have influenced the observed QTN and spurious pleiotropy detection rates in this study. A potential solution for this issue would be to simulate pleiotropy and linked QTNs based on marker data with SNPs evenly spaced.

One final suggestion for future research is to investigate the impact of (i) the residual correlation between traits and (ii) the sign of QTN effect sizes on the performance of univariate and multivariate GWAS. As described in Jiang and Zeng (1995), the power of multivariate approaches should be less than those of the univariate ones whenever the direction of residual correlation (i.e., whether the sign of the residual correlation is positive or negative) is the same as those of the product of QTN effect sizes, regardless of whether these QTNs are in linkage or are pleiotropic. Thus, it is critical to determine if the overall patterns of QTN and spurious pleiotropy detection observed in this study are similar under genetic architectures where multivariate GWAS is theoretically expected to yield lower power than univariate GWAS.

## 5. Conclusion

The main conclusion from this study is that the use of either univariate or multivariate GWAS alone is insufficient for rigorously dissecting the genetic architecture of multiple traits. Association studies should instead use both univariate and multivariate models, as we demonstrated that both of these models are useful. Although our results suggest that multivariate GWAS cannot distinguish between a single pleiotropic QTN and multiple non-pleiotropic QTNs in LD, we confirmed that multivariate models are potentially useful for analyzing traits that are controlled by causal mutations that are either pleiotropic or in LD with each other. Once the genomic regions most likely to contain relevant causal mutations are identified through multivariate GWAS, univariate analyses could then be applied, potentially through the *a posteriori* analysis proposed in the Discussion, to shed light on whether or not the underlying causal mutations are pleiotropic. Such use of univariate and multivariate analyses in a concerted manner could maximize the amount of information ascertained from the GWAS of multiple traits, and potentially provide biological researchers with a smaller list of candidate loci that are likely to contribute to their variability.

## Data Availability Statement

The original contributions presented in the study are included in the article/Supplementary Material, further inquiries can be directed to the corresponding author/s.

## Author Contributions

SF, AL, and TJ designed the experiments. SF and KZ conducted the experiments. SF, AL, KZ, and TJ wrote and edited the manuscript. All authors contributed to the article and approved the submitted version.

## Conflict of Interest

The authors declare that the research was conducted in the absence of any commercial or financial relationships that could be construed as a potential conflict of interest.

## Supplementary Material

R scripts can also be found at: https://github.com/samuelbfernandes/fernandes_et_al_2020.
