# Supplementary material for: How Well Can Multivariate and Univariate GWAS Distinguish Between True and Spurious Pleiotropy?
Source: Front Genet. 2021 Jan 8;11:602526. doi: 10.3389/fgene.2020.602526 (PMC7873880; doi:10.3389/fgene.2020.602526)
Supplement: Supplementary file 1 [file Data_Sheet_1.PDF]

How well can multivariate and univariate GWAS distinguish between true and spurious pleiotropy?

Samuel B. Fernandes*, Kevin Zhang, Tiffany M. Jamann, and Alexander E. Lipka†

Supplementary File

This is the supplementary file that includes figures and scripts for Fernandes et al. (2020). All the simulations below were run on simplePHENOTYPES v1.2.13.

Figures

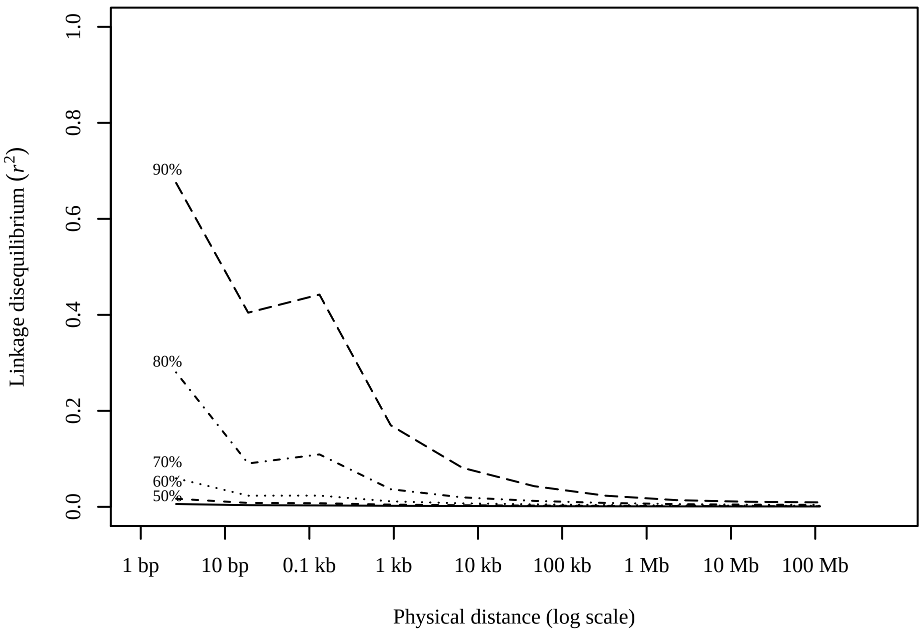

Figure S1: Linkage disequilibrium (r^2) decay plot (physical distance in log scale) of the filtered maize data set. The different lines represent different percentiles (90, 80, 70, 60, and 50 %).

*samuelf@illinois.edu
†alipka@illinois.edu

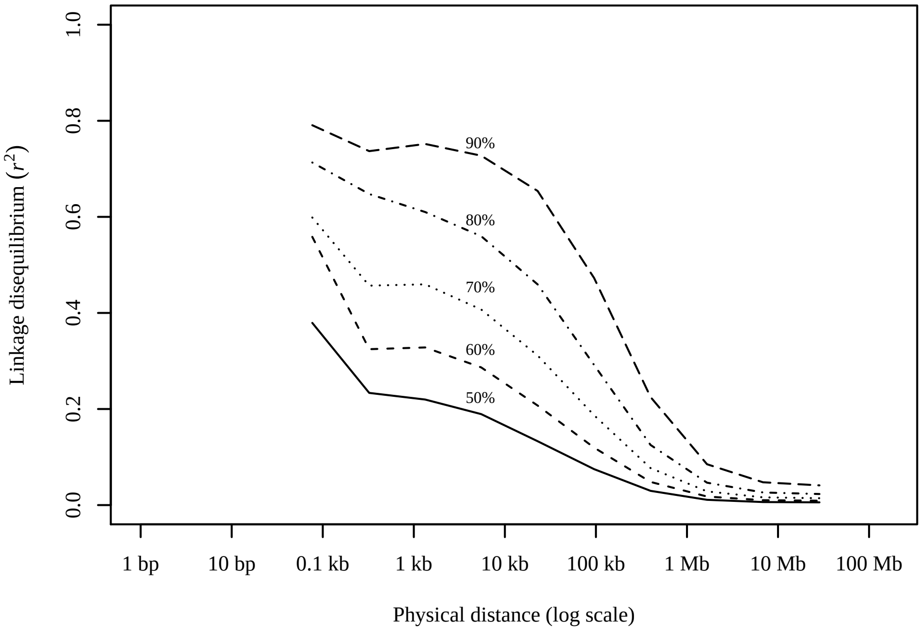

Figure S2: Linkage disequilibrium (r^2) decay plot (physical distance in log scale) of the filtered soybean data set. The different lines represent different percentiles (90, 80, 70, 60, and 50 %).

Regional Linkage Disequilibrium (LD) and Simulation Diagnostics

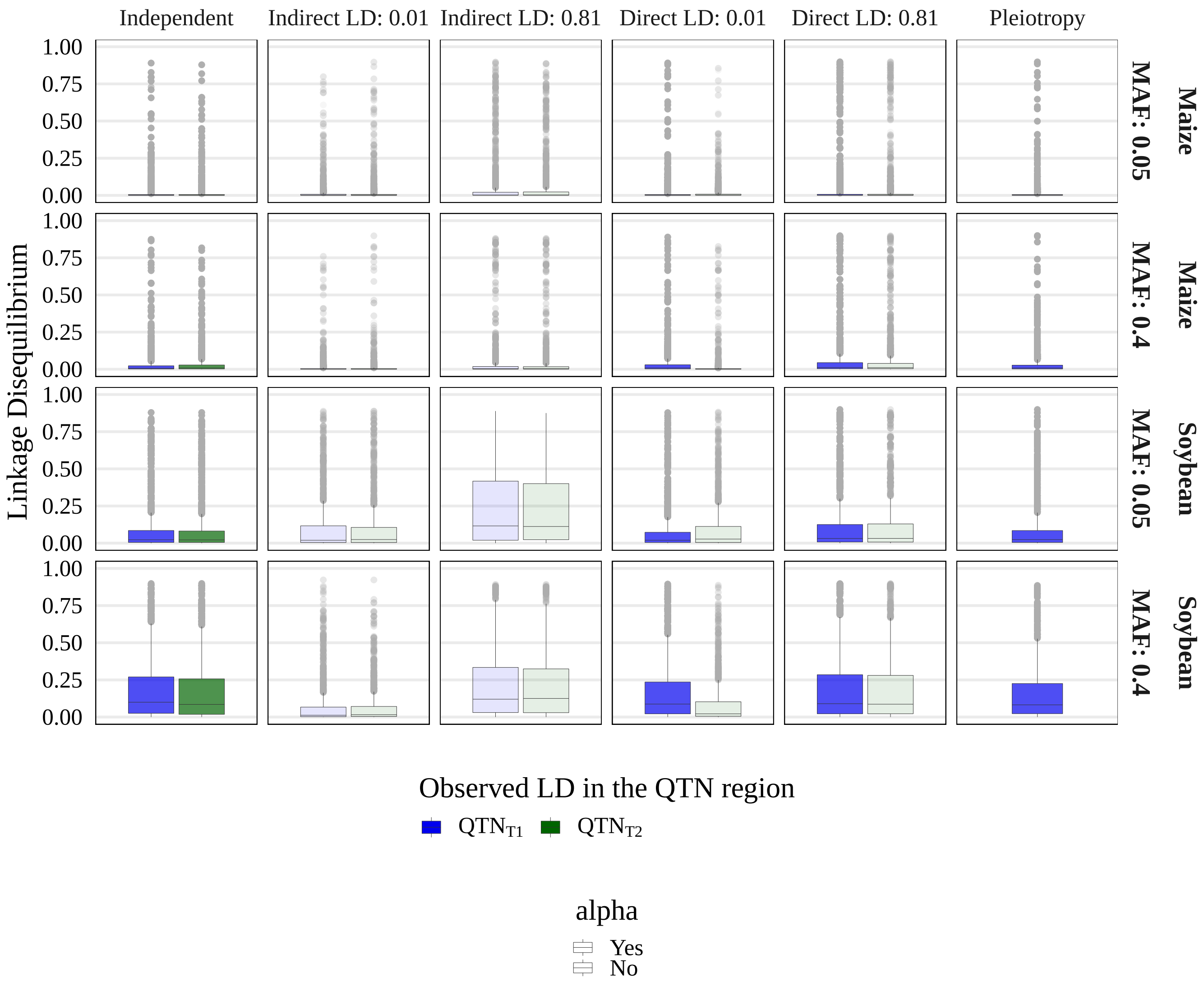

Figure S3: Observed linkage disequilibrium between quantitative trait nucleotides (QTN) and the 20 SNPs upstream and the 20 SNPs downstream for trait 1 (QTN_{T1}) and trait 2 (QTN_{T2}), minor allele frequencies (MAF) of 0.05 and 0.4, and a sample size of 500. Darker colors indicate QTNs that had MAF directly controlled by an input parameter of the simulation, whereas lighter colors indicate QTNs where MAF was not controlled. The simulated genetic architecture is listed in the horizontal and vertical titles.

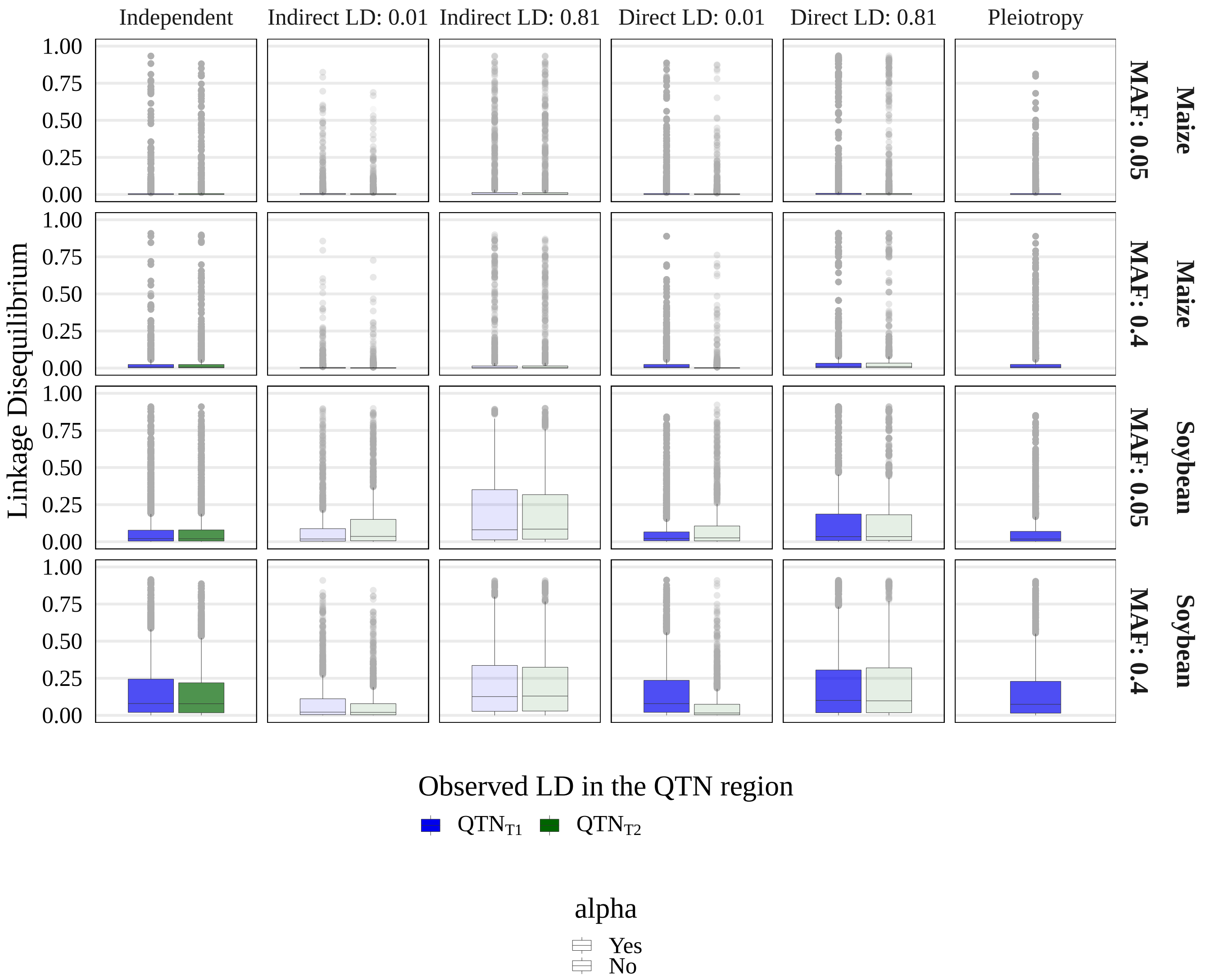

Figure S4: Observed linkage disequilibrium between quantitative trait nucleotides (QTN) and the 20 SNPs upstream and the 20 SNPs downstream for trait 1 (QTN$_{T1}$) and trait 2 (QTN$_{T2}$), minor allele frequencies (MAF) of 0.05 and 0.4, and a sample size of $1,000$. Darker colors indicate QTNs that had MAF directly controlled by an input parameter of the simulation, whereas lighter colors indicate QTNs where MAF was not controlled. The simulated genetic architecture is listed in the horizontal and vertical titles.

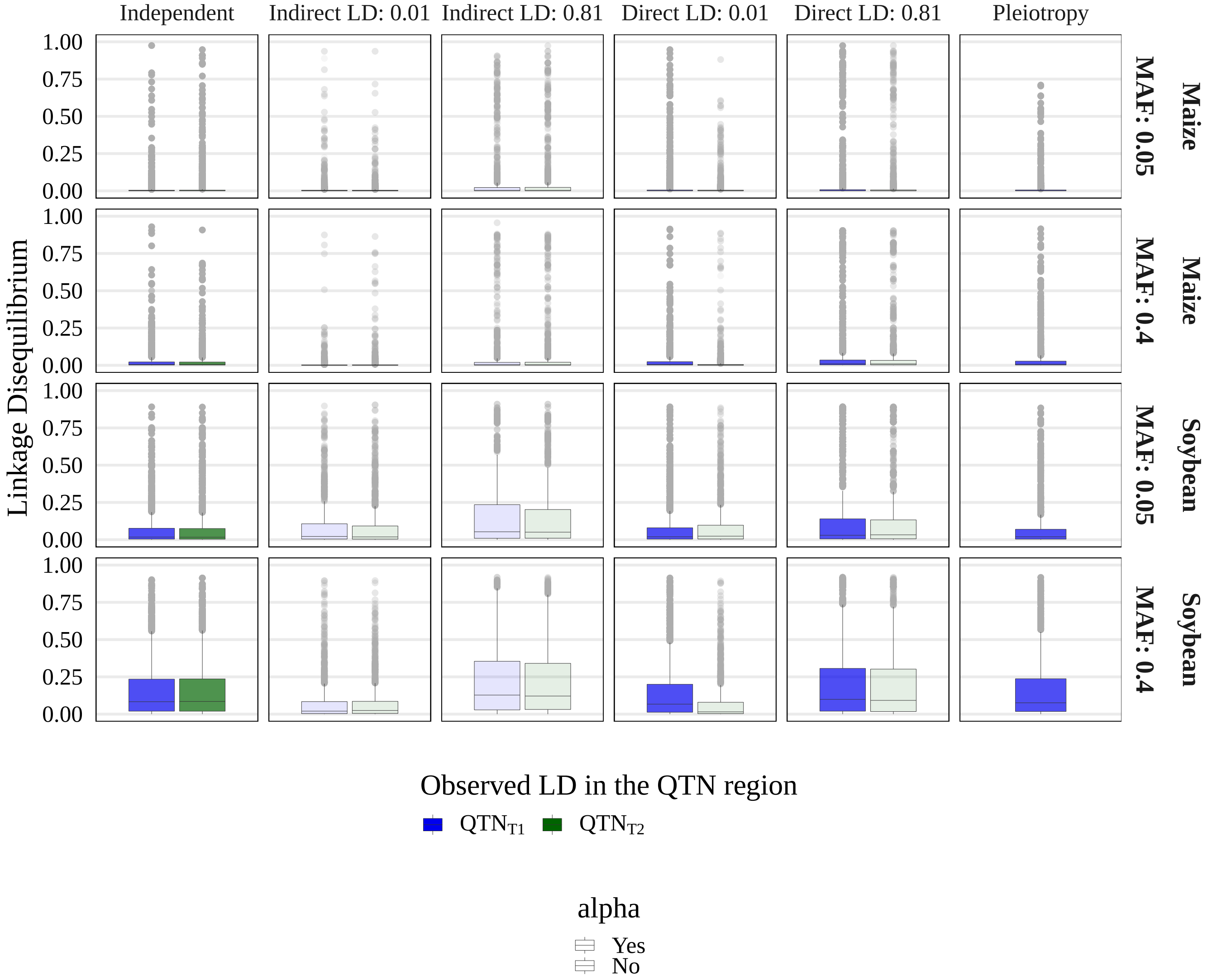

Figure S5: Observed linkage disequilibrium between quantitative trait nucleotides (QTN) and the 20 SNPs upstream and the 20 SNPs downstream for trait 1 (QTN_{T1}) and trait 2 (QTN_{T2}), minor allele frequencies (MAF) of 0.05 and 0.4, and a sample size of $2,815$. Darker colors indicate QTNs that had MAF directly controlled by an input parameter of the simulation, whereas lighter colors indicate QTNs where MAF was not controlled. The simulated genetic architecture is listed in the horizontal and vertical titles.

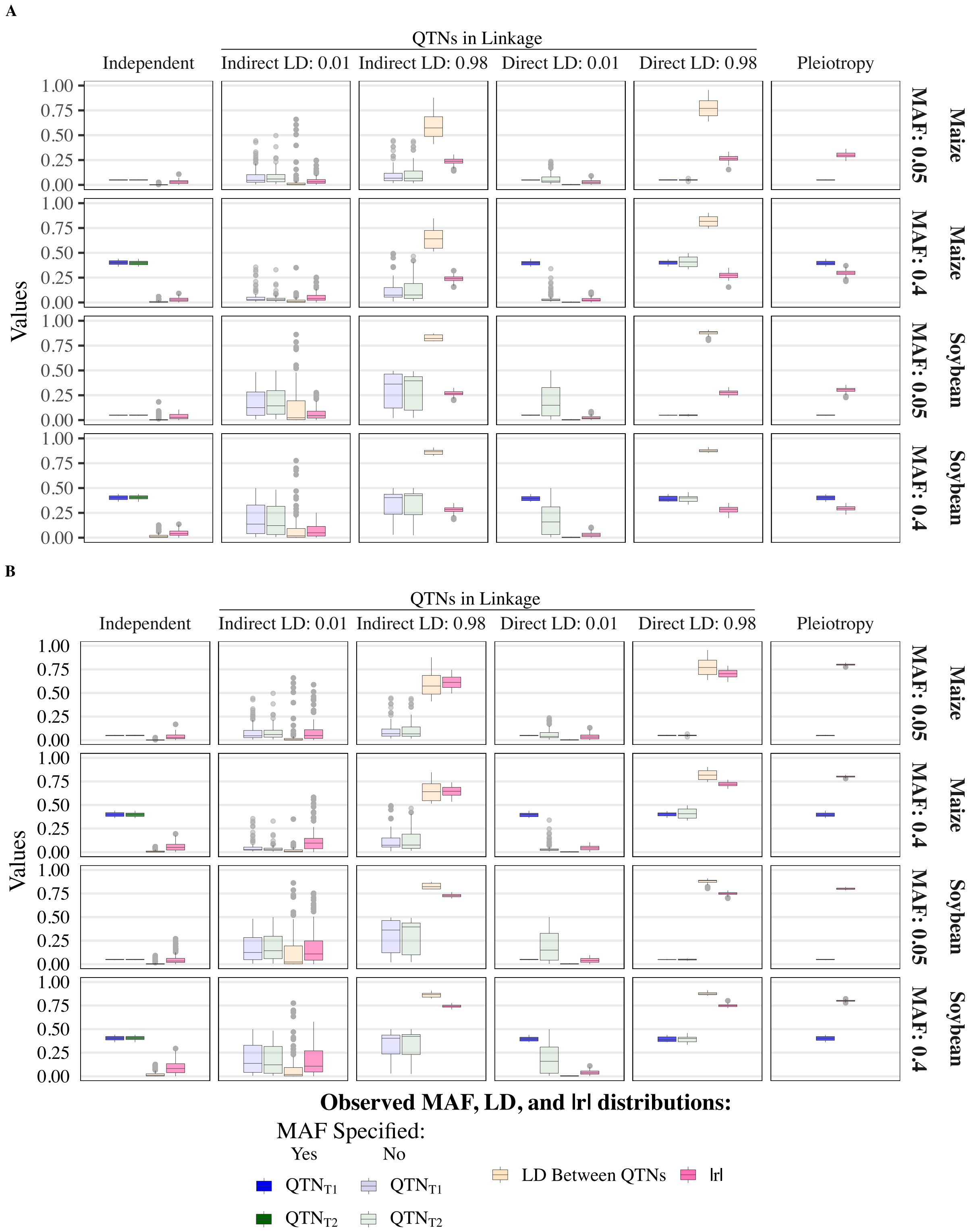

Figure S6: Observed minor allele frequencies (MAF) for quantitative trait nucleotides (QTN) controlling trait 1 (QTN_{T1}) and trait 2 (QTN_{T2}), and the observed linkage disequilibrium (LD) and absolue phenotypic correlation (|r|) between them, measured as r^2, for the sample size of $1,000$ for traits 1 and 2, respectively. A) Narrow-sense heritability of 0.3 for both traits. B) Narrow-sense heritability of 0.8 for both traits. Darker colors indicate QTNs that had MAF directly controlled by an input parameter of the simulation, whereas lighter colors indicate QTNs where MAF was not controlled. The simulated genetic architecture is listed in the horizontal and vertical titles.

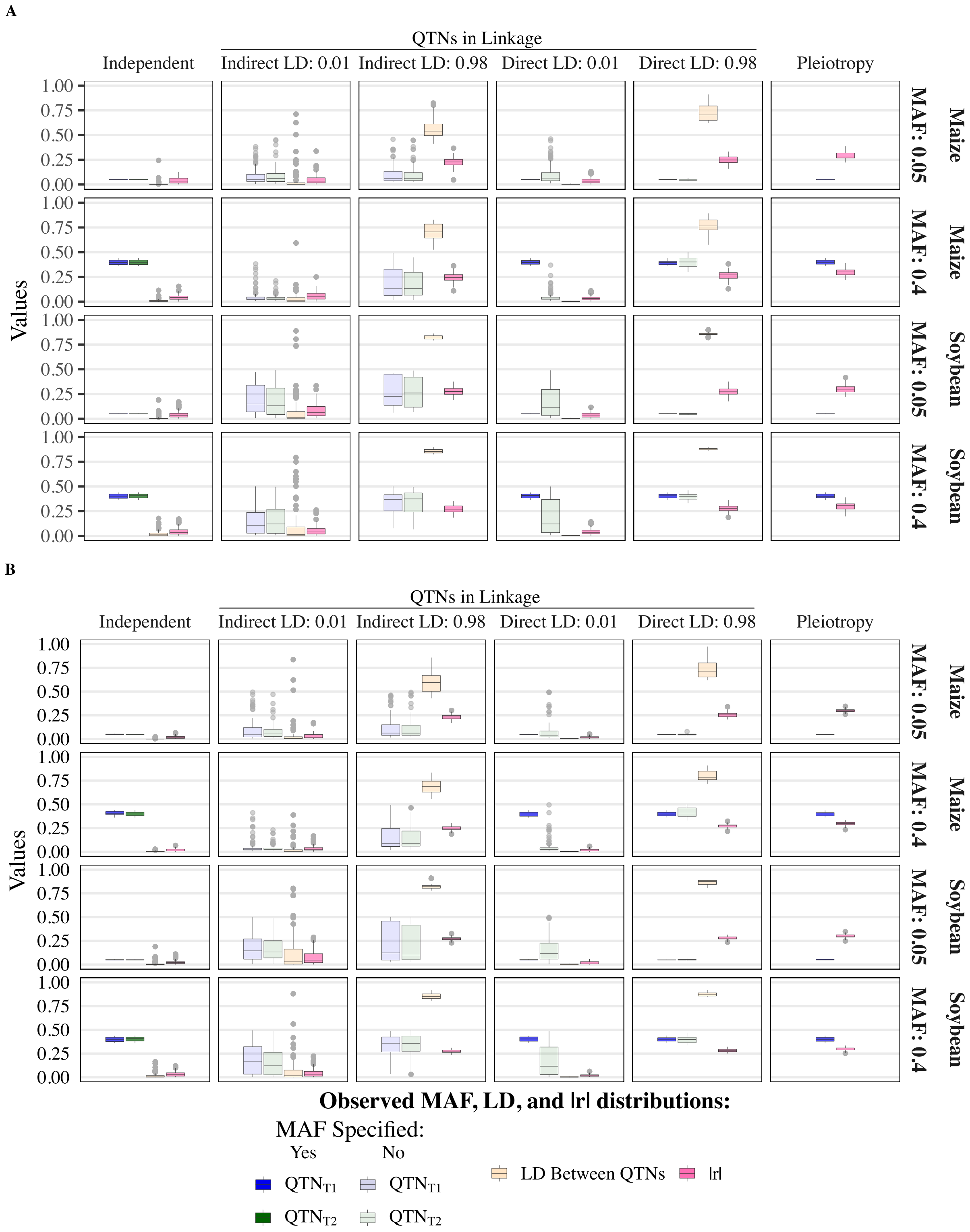

Figure S7: Observed minor allele frequencies (MAF) for quantitative trait nucleotides (QTN) controlling trait 1 (QTN_{T1}) and trait 2 (QTN_{T2}), and the observed linkage disequilibrium (LD) and absolue phenotypic correlation (|r|) between them, measured as r^2, for the narrow-sense heritability of 0.3 for both traits. A) Sample size of 500. B) Sample size of 2815. Darker colors indicate QTNs that had MAF directly controlled by an input parameter of the simulation, whereas lighter colors indicate QTNs where MAF was not controlled. The simulated genetic architecture is listed in the horizontal and vertical titles.

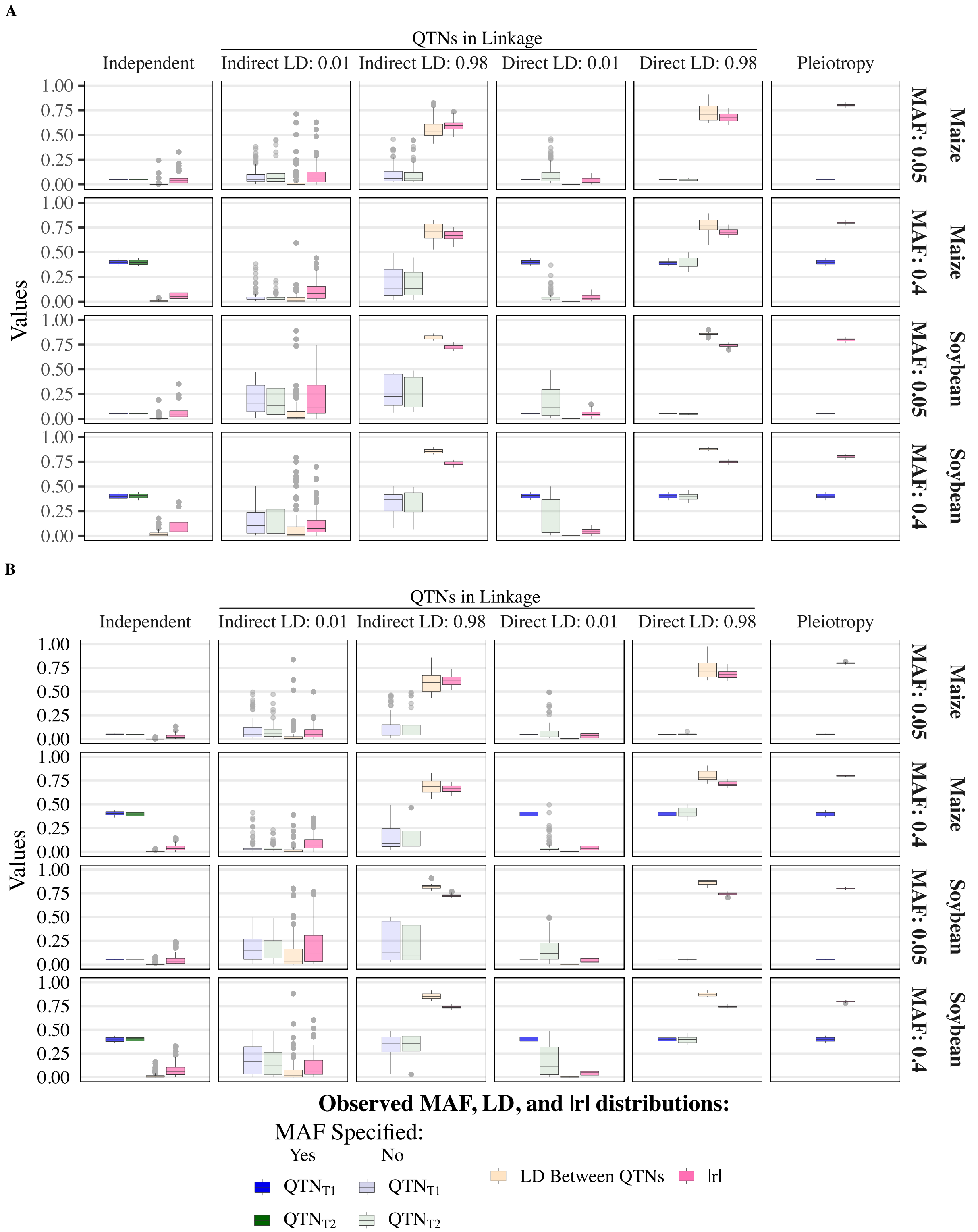

Figure S8: Observed minor allele frequencies (MAF) for quantitative trait nucleotides (QTN) controlling trait 1 (QTN_{T1}) and trait 2 (QTN_{T2}), and the observed linkage disequilibrium (LD) and absolue phenotypic correlation (|r|) between them, measured as r^2, for the narrow-sense heritability of 0.8 for both traits. A) Sample size of 500. B) Sample size of 2815. Darker colors indicate QTNs that had MAF directly controlled by an input parameter of the simulation, whereas lighter colors indicate QTNs where MAF was not controlled. The simulated genetic architecture is listed in the horizontal and vertical titles.

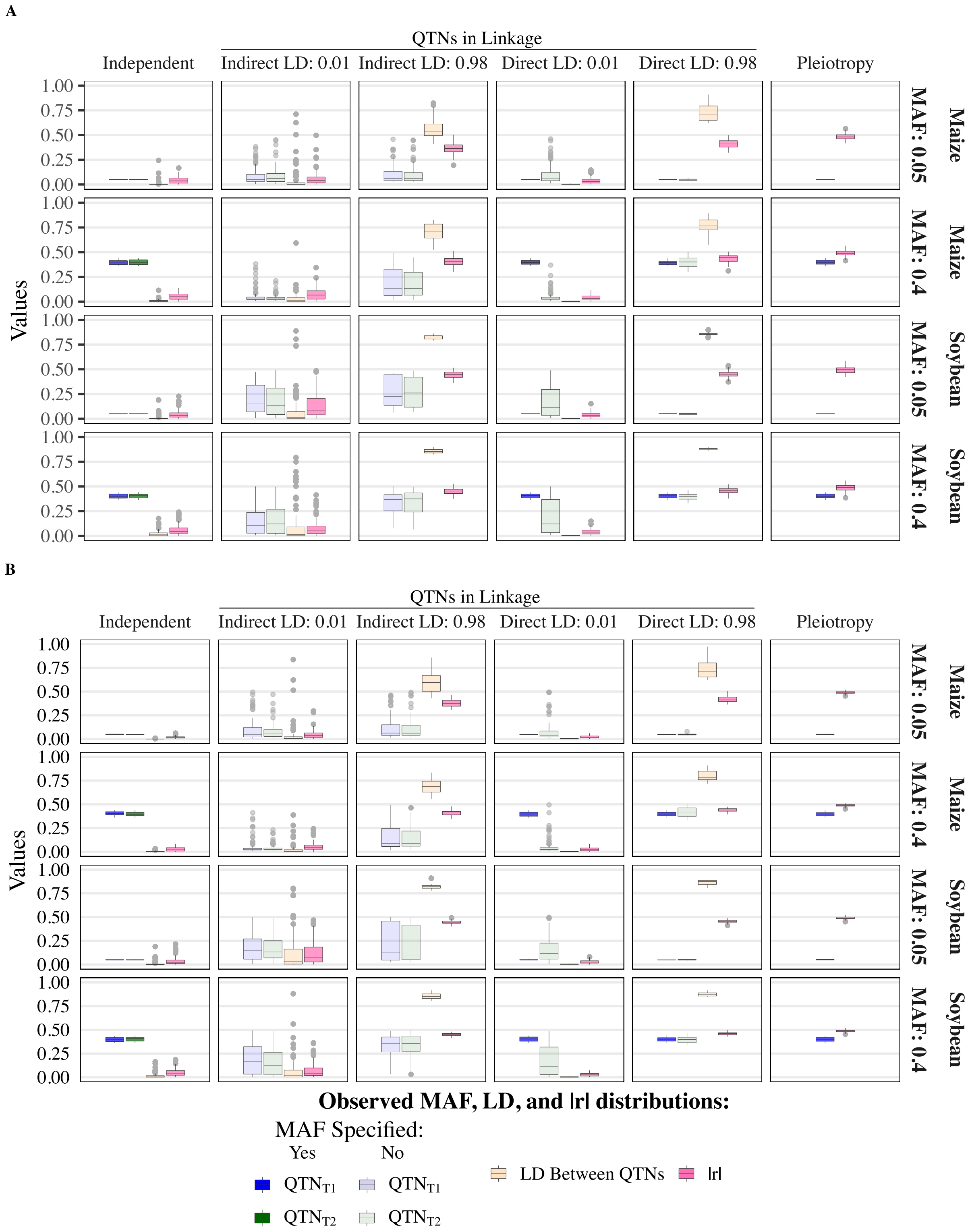

Figure S9: Observed minor allele frequencies (MAF) for quantitative trait nucleotides (QTN) controlling trait 1 (QTN_{T1}) and trait 2 (QTN_{T2}), and the observed linkage disequilibrium (LD) and absolue phenotypic correlation (|r|) between them, measured as r^2, for the narrow-sense heritability of 0.3 and 0.8 for traits 1 and 2, respectively. A) Sample size of 500. B) Sample size of 2815. Darker colors indicate QTNs that had MAF directly controlled by an input parameter of the simulation, whereas lighter colors indicate QTNs where MAF was not controlled. The simulated genetic architecture is listed in the horizontal and vertical titles.

QTN detection with an FDR of 0.1 and window size of 10 Kb for maize and 1 Mb for soybean

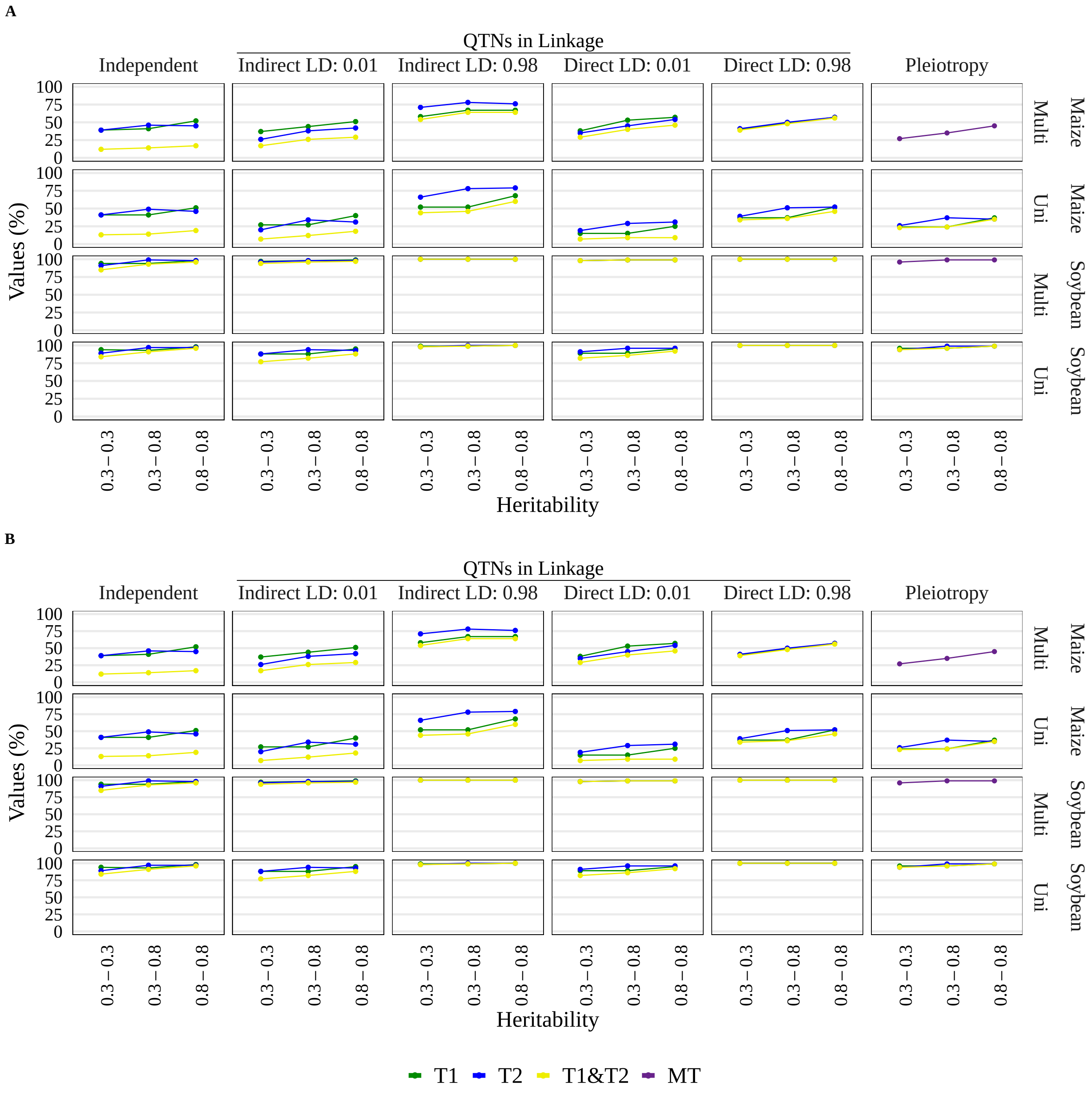

Figure S10: Quantitative trait nucleotide (QTN) and spurious pleiotropy detection rate (Y-axis) achieved by multivariate (Multi) and univariate (Uni) GWAS, relative to the QTN controlling trait 1 (T1), trait 2 (T2), and both QTN simultaneously (T1&T2) or, in the pleiotropic scenario, relative to the pleiotropic QTN (MT). These values were obtained for maize and soybean with a sample size of 500. The X-axis displays the narrow-sense heritability for Trait 1 (bottom value) and Trait 2 (top value). A) inputted minor allele frequency (MAF) of 0.05; B) MAF of 0.4. The false discovery rate rate was 0.1 and the window size was 10 kb for maize and 1 Mb for soybean.

A

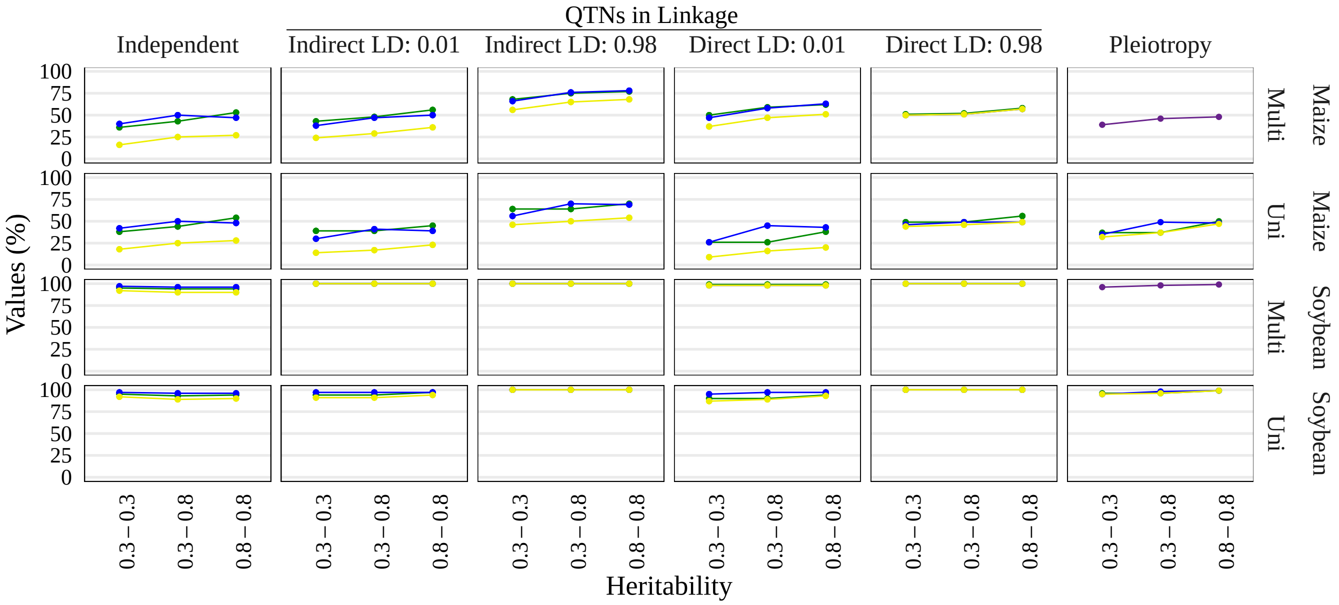

B

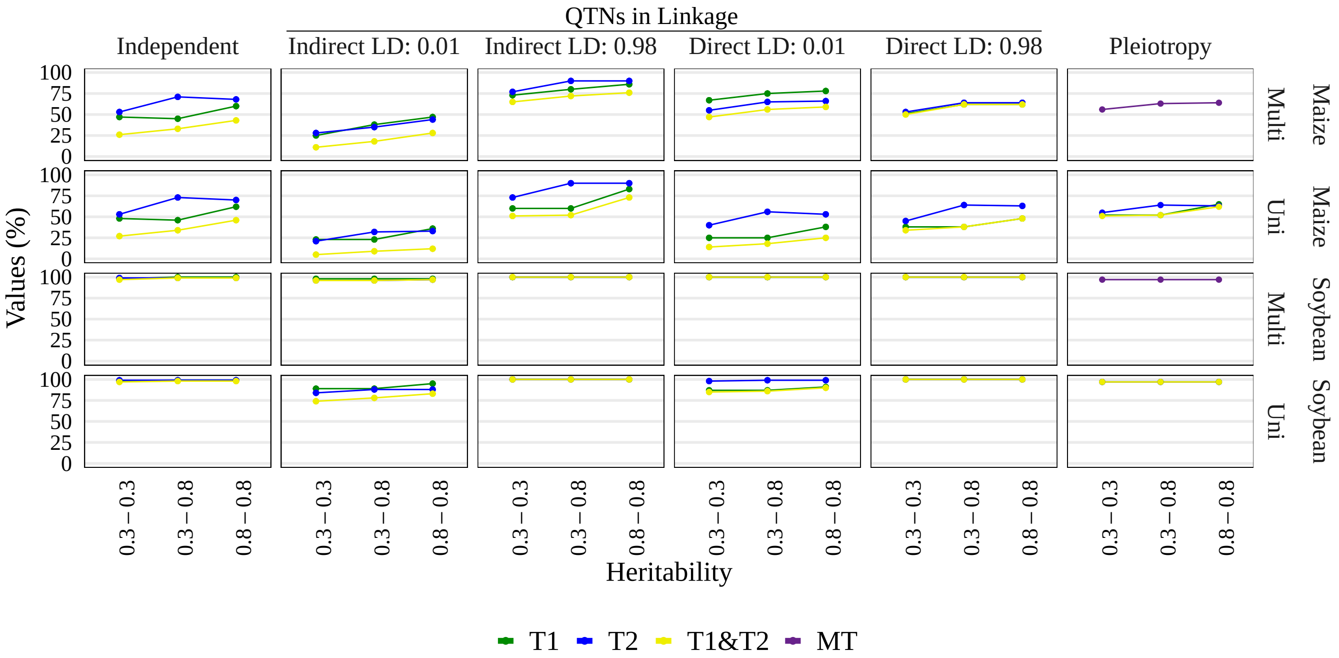

Figure S11: Quantitative trait nucleotide (QTN) and spurious pleiotropy detection rate (Y-axis) achieved by multivariate (Multi) and univariate (Uni) GWAS, relative to the QTN controlling trait 1 (T1), trait 2 (T2), and both QTN simultaneously (T1&T2) or, in the pleiotropic scenario, relative to the pleiotropic QTN (MT). These values were obtained for maize and soybean with a sample size of 1,000. The X-axis displays the narrow-sense heritability for Trait 1 (bottom value) and Trait 2 (top value). A) inputted minor allele frequency (MAF) of 0.05; B) MAF of 0.4. The false discovery rate rate was 0.1 and the window size was 10 kb for maize and 1 Mb for soybean.

A

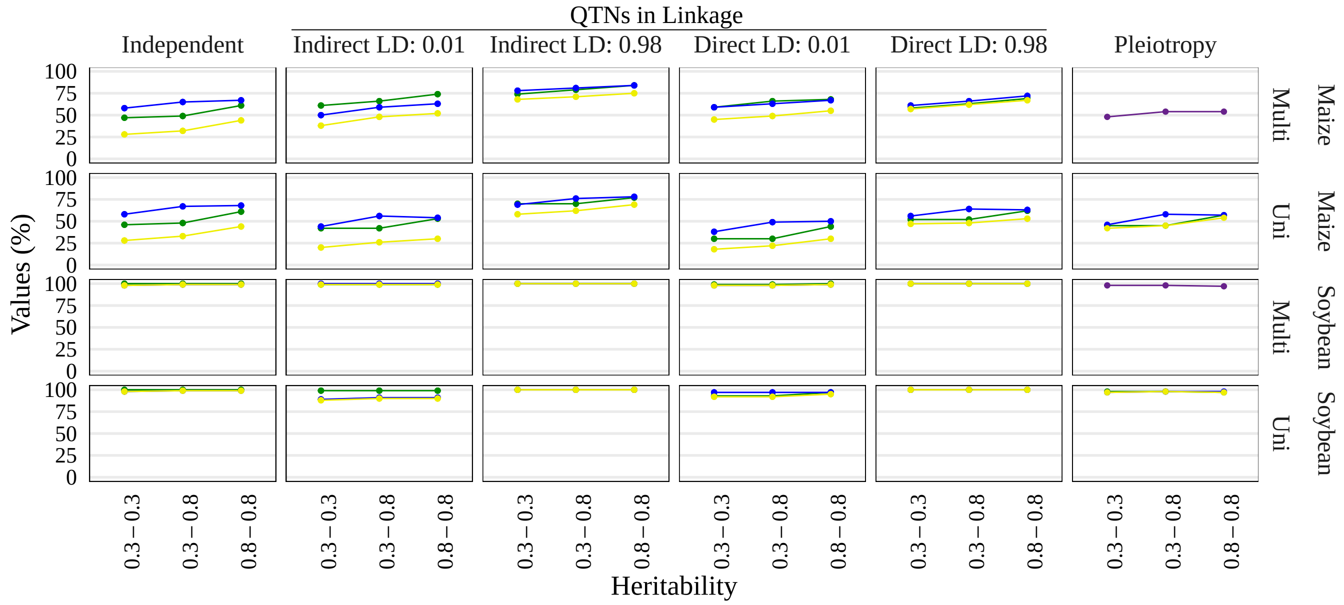

B

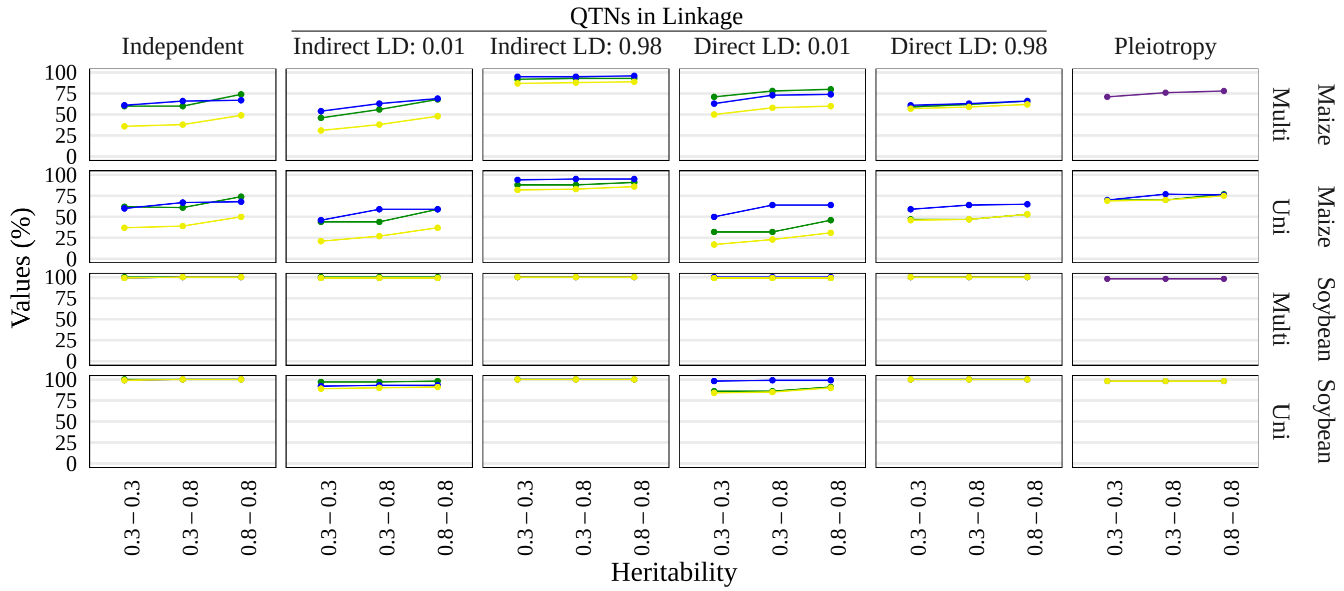

Figure S12: Quantitative trait nucleotide (QTN) and spurious pleiotropy detection rate (Y-axis) achieved by multivariate (Multi) and univariate (Uni) GWAS, relative to the QTN controlling trait 1 (T1), trait 2 (T2), and both QTN simultaneously (T1&T2) or, in the pleiotropic scenario, relative to the pleiotropic QTN (MT). These values were obtained for maize and soybean with a sample size of 2,815. The X-axis displays the narrow-sense heritability for Trait 1 (bottom value) and Trait 2 (top value). A) inputted minor allele frequency (MAF) of 0.05; B) MAF of 0.4. The false discovery rate rate was 0.1 and the window size was 10 kb for maize and 1 Mb for soybean.

A

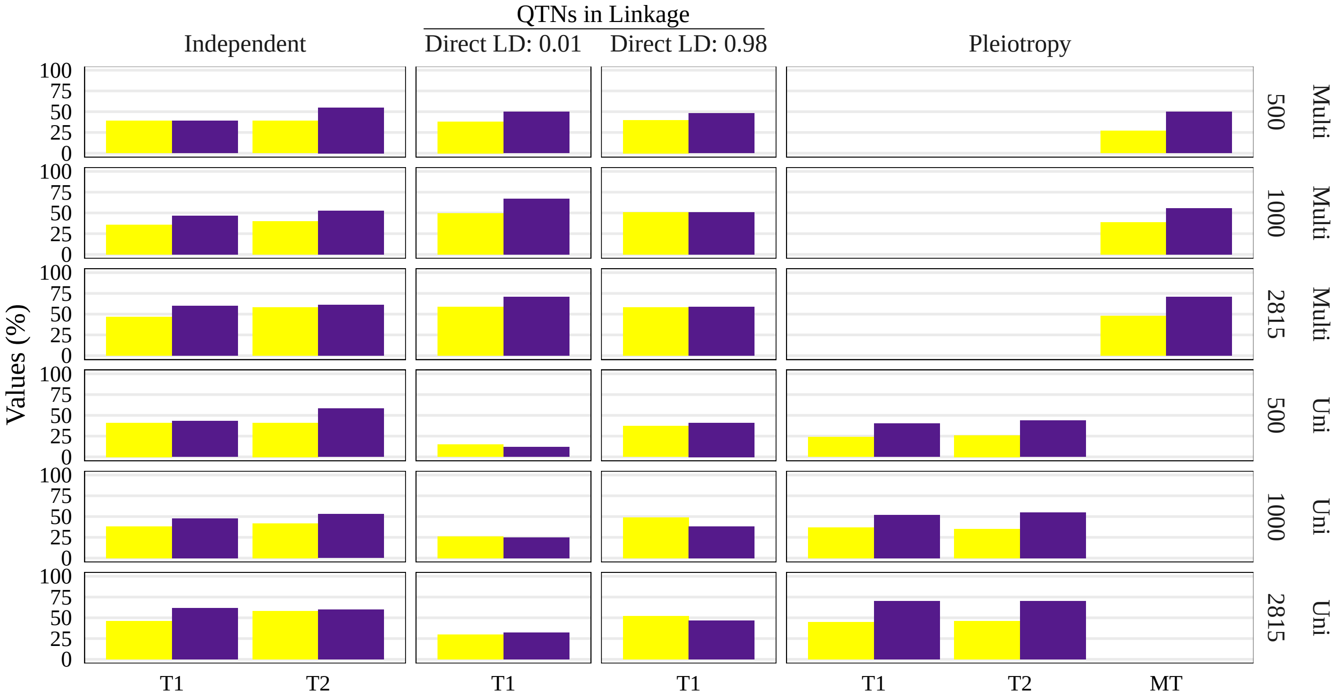

B

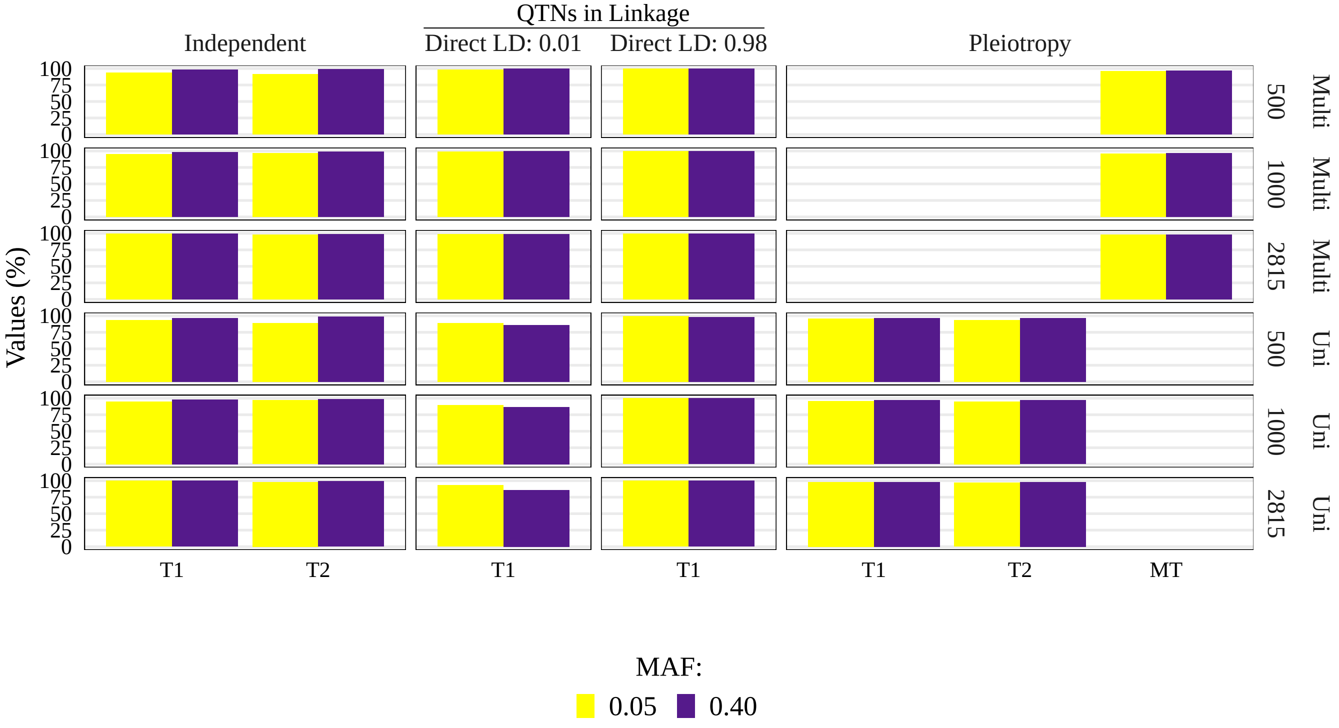

Figure S13: Quantitative trait nucleotide (QTN) and spurious pleiotropy detection rate (Y-axis) in scenarios for which minor allele frequency (MAF) was directly controlled by a simulation input parameter. These values were obtained by multivariate (Multi) and univariate (Uni) GWAS, relative to the QTN controlling trait 1 (T1), trait 2 (T2) or, in the pleiotropic scenario, relative to the pleiotropic QTN (MT). This figure shows results for a narrow-sense heritability of 0.3 for both traits. A) Maize; B) Soybean. The false discovery rate rate was 0.1 and the window size was 10 kb for maize and 1 Mb for soybean.

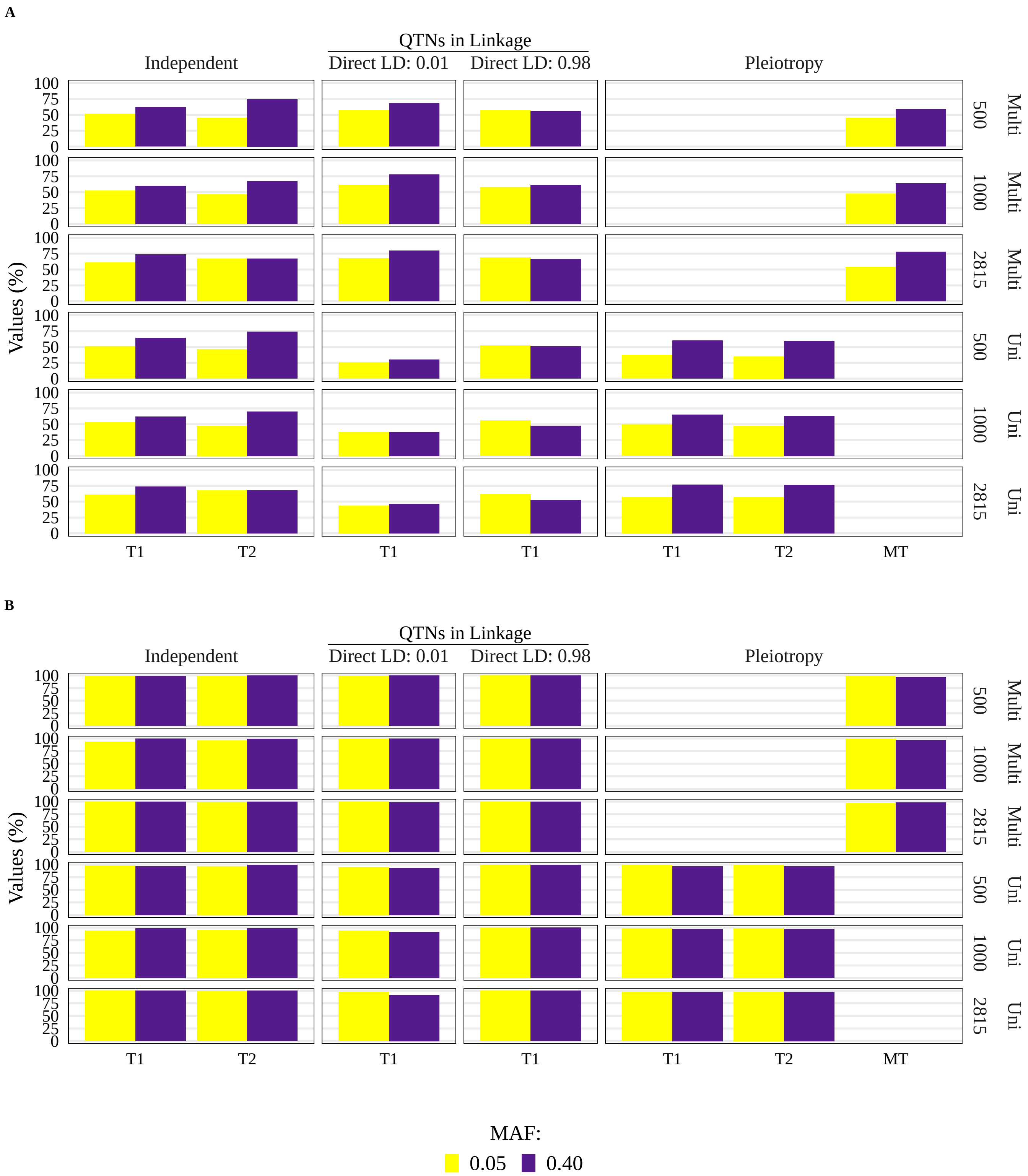

Figure S14: Quantitative trait nucleotide (QTN) and spurious pleiotropy detection rate (Y-axis) in scenarios for which minor allele frequency (MAF) was directly controlled by a simulation input parameter. These values were obtained by multivariate (Multi) and univariate (Uni) GWAS, relative to the QTN controlling trait 1 (T1), trait 2 (T2) or, in the pleiotropic scenario, relative to the pleiotropic QTN (MT). This figure shows results for a narrow-sense heritability of 0.8 for both traits. A) Maize; B) Soybean. The false discovery rate rate was 0.1 and the window size was 10 kb for maize and 1 Mb for soybean.

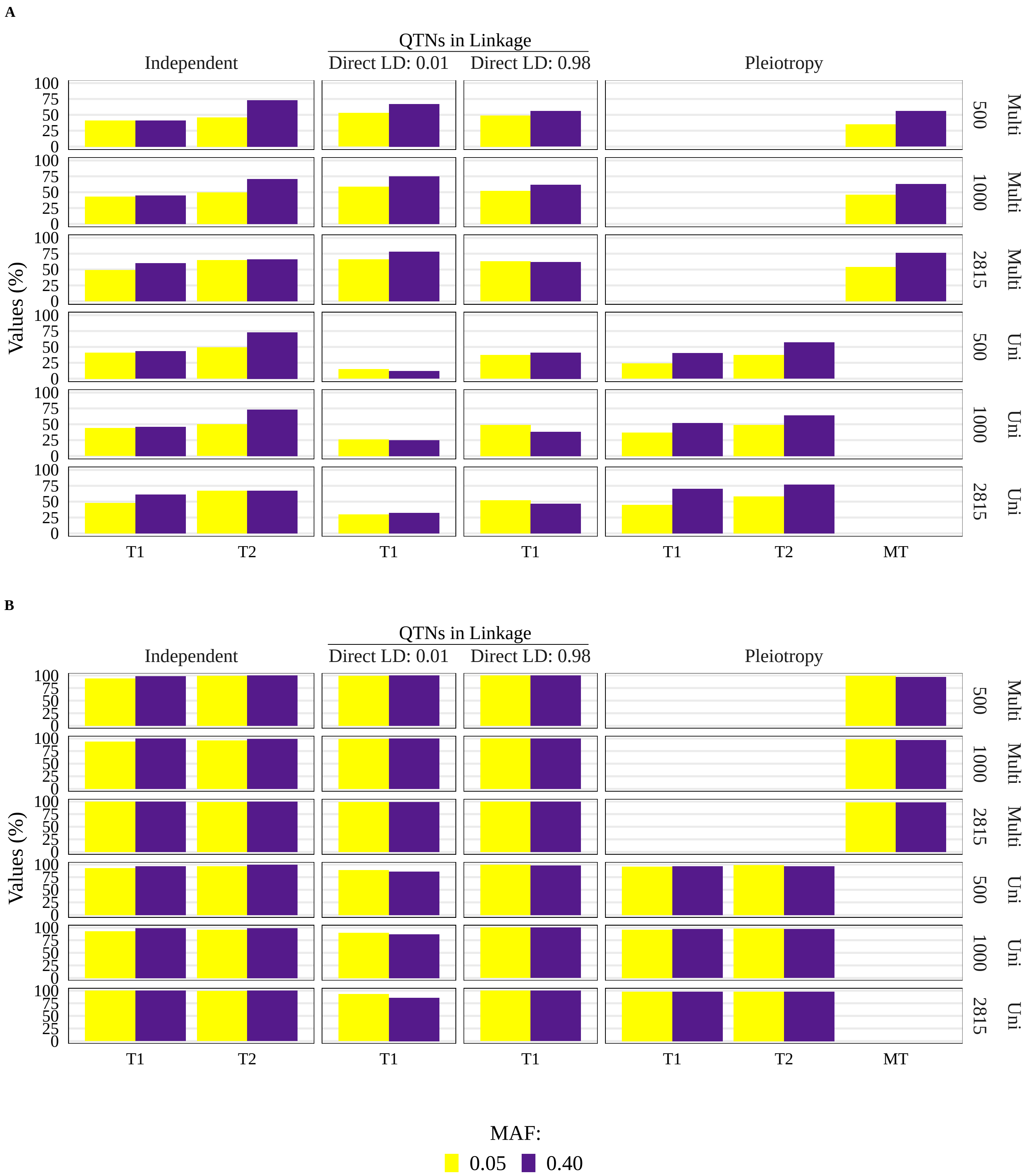

Figure S15: Quantitative trait nucleotide (QTN) and spurious pleiotropy detection rate (Y-axis) in scenarios for which minor allele frequency (MAF) was directly controlled by a simulation input parameter. These values were obtained by multivariate (Multi) and univariate (Uni) GWAS, relative to the QTN controlling trait 1 (T1), trait 2 (T2) or, in the pleiotropic scenario, relative to the pleiotropic QTN (MT). This figure shows results for a narrow-sense heritability of 0.3 for trait 1 and 0.8 for trait 2. A) Maize; B) Soybean. The false discovery rate rate was 0.1 and the window size was 10 kb for maize and 1 Mb for soybean.

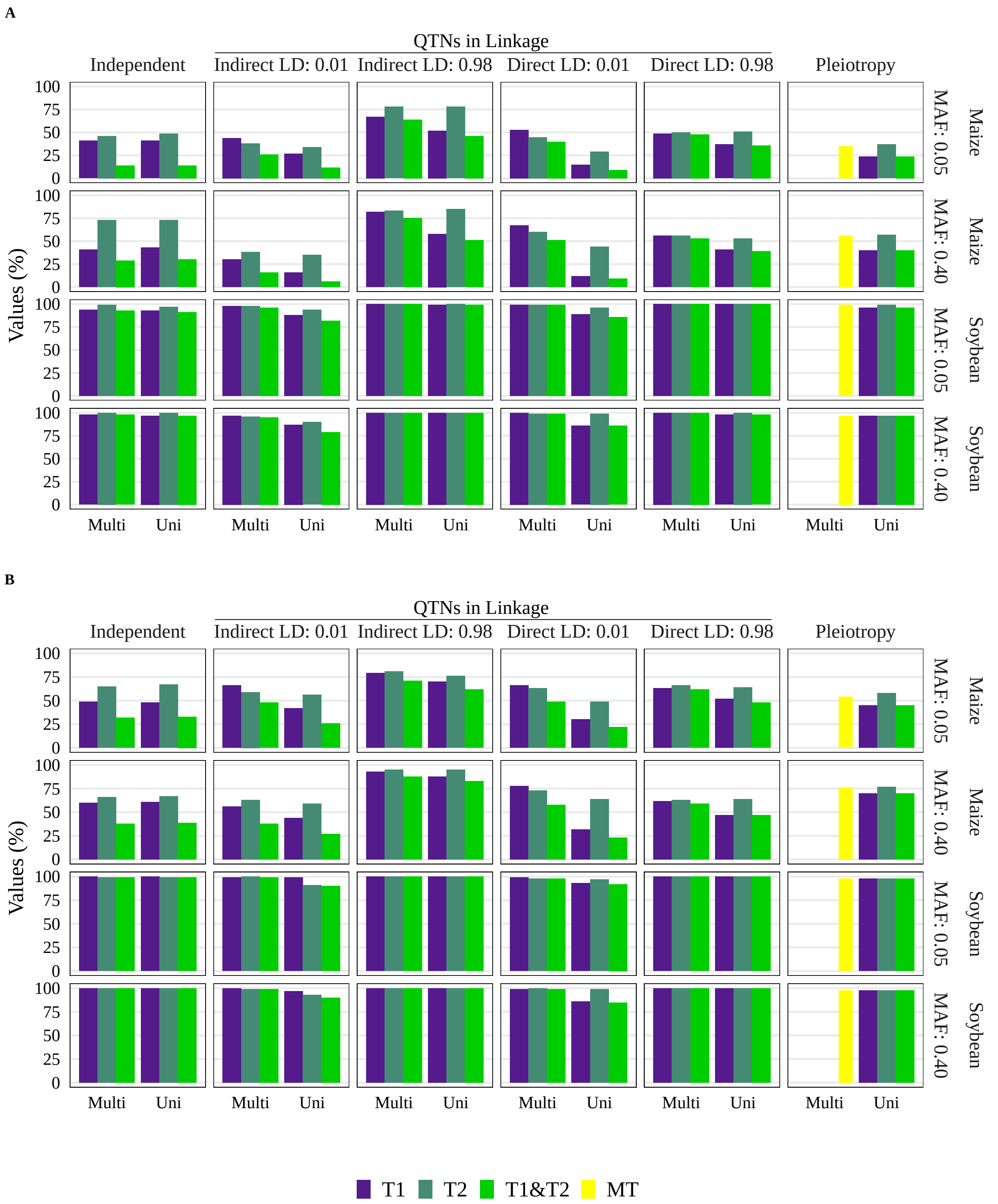

Figure S16: Quantitative trait nucleotide (QTN) and spurious pleiotropy detection rate (Y-axis) achieved by multivariate (Multi) and univariate (Uni) GWAS (X-axis), relative to the QTN controlling trait 1 (T1), trait 2 (T2), and both QTN simultaneously (T1&T2) or, in the pleiotropic scenario, relative to the pleiotropic QTN (MT). The simulated genetic architecture is listed in the horizontal and vertical titles. These values were obtained with a narrow-sense heritability of 0.3 and 0.8 for traits 1 and 2, respectively. A) Sample size of 500; B) Sample size of 2815. MAF: minor allele frequencies. The false discovery rate rate was 0.1 and the window size was 10 kb for maize and 1 Mb for soybean.

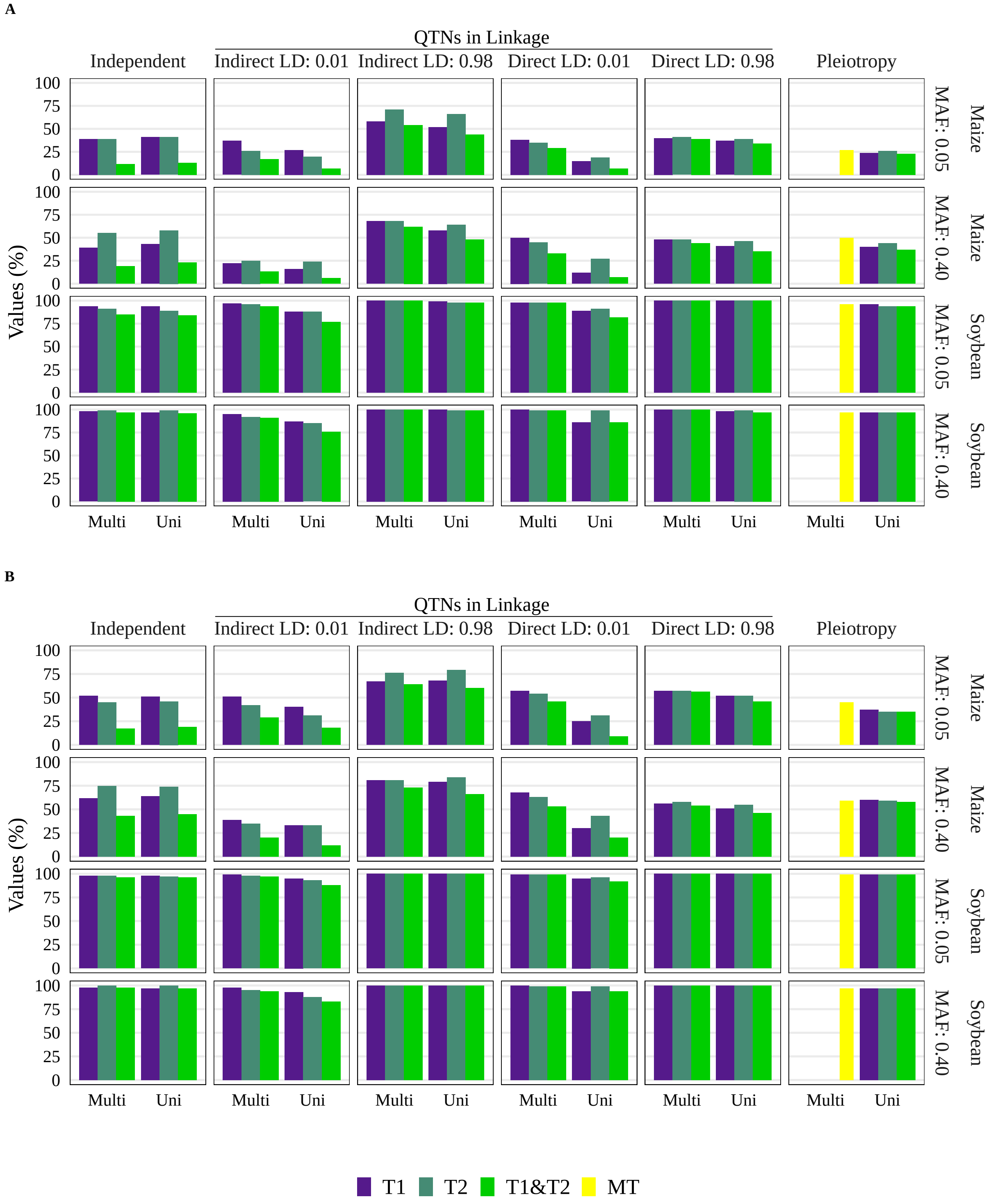

Figure S17: Quantitative trait nucleotide (QTN) and spurious pleiotropy detection rate (Y-axis) achieved by multivariate (Multi) and univariate (Uni) GWAS (X-axis), relative to the QTN controlling trait 1 (T1), trait 2 (T2), and both QTN simultaneously (T1&T2) or, in the pleiotropic scenario, relative to the pleiotropic QTN (MT). The simulated genetic architecture is listed in the horizontal and vertical titles. These values were obtained with a sample size of 500; A) a narrow-sense heritability of 0.3 for both traits; B) a narrow-sense heritability of 0.8 for both traits. MAF: minor allele frequencies. The false discovery rate rate was 0.1 and the window size was 10 kb for maize and 1 Mb for soybean.

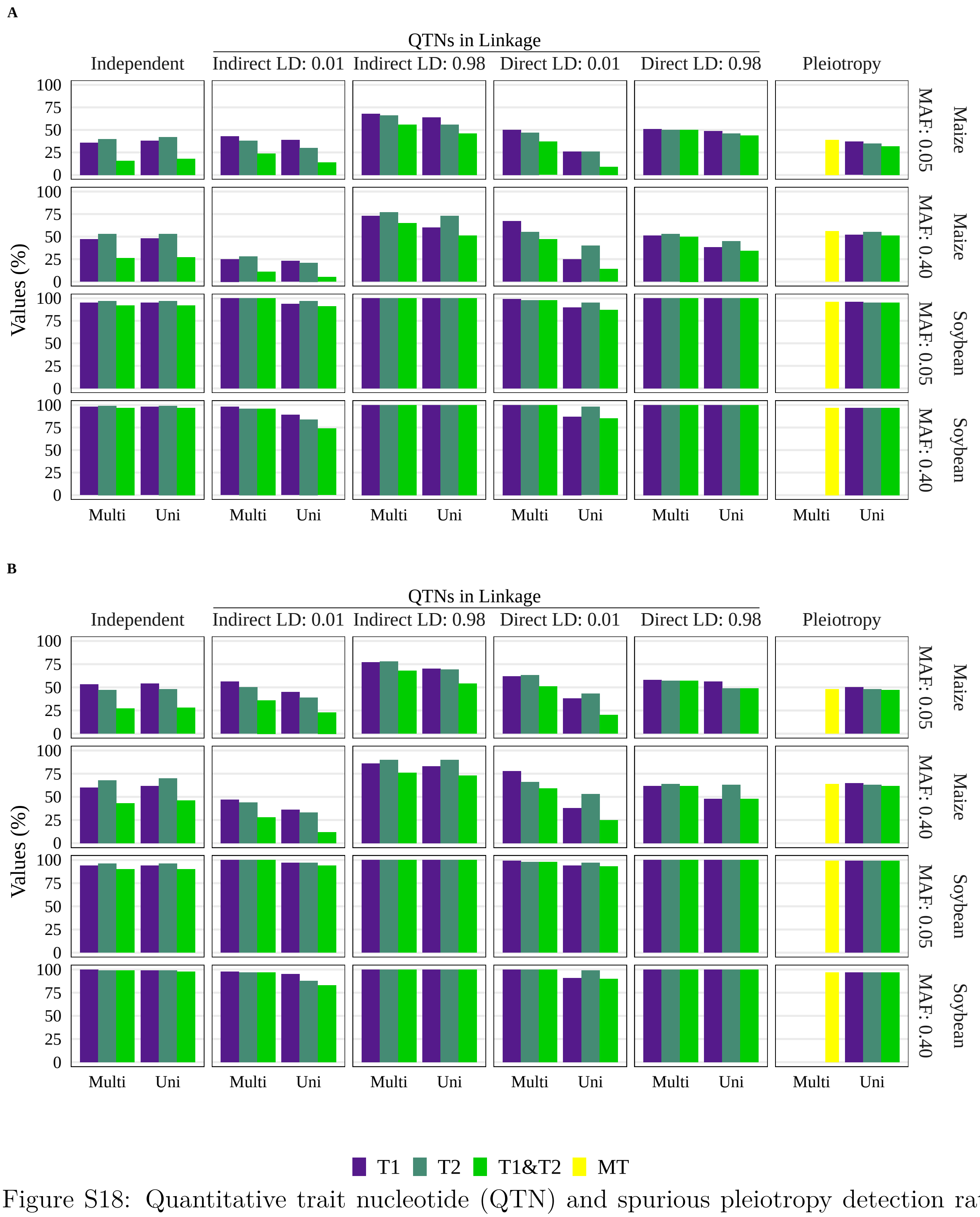

Figure S18: Quantitative trait nucleotide (QTN) and spurious pleiotropy detection rate (Y-axis) achieved by multivariate (Multi) and univariate (Uni) GWAS (X-axis), relative to the QTN controlling trait 1 (T1), trait 2 (T2), and both QTN simultaneously (T1&T2) or, in the pleiotropic scenario, relative to the pleiotropic QTN (MT). The simulated genetic architecture is listed in the horizontal and vertical titles. These values were obtained with a sample size of 1, 000; A) a narrow-sense heritability of 0.3 for both traits; B) a narrow-sense heritability of 0.8 for both traits. MAF: minor allele frequencies. The false discovery rate rate was 0.1 and the window size was 10 kb for maize and 1 Mb for soybean.

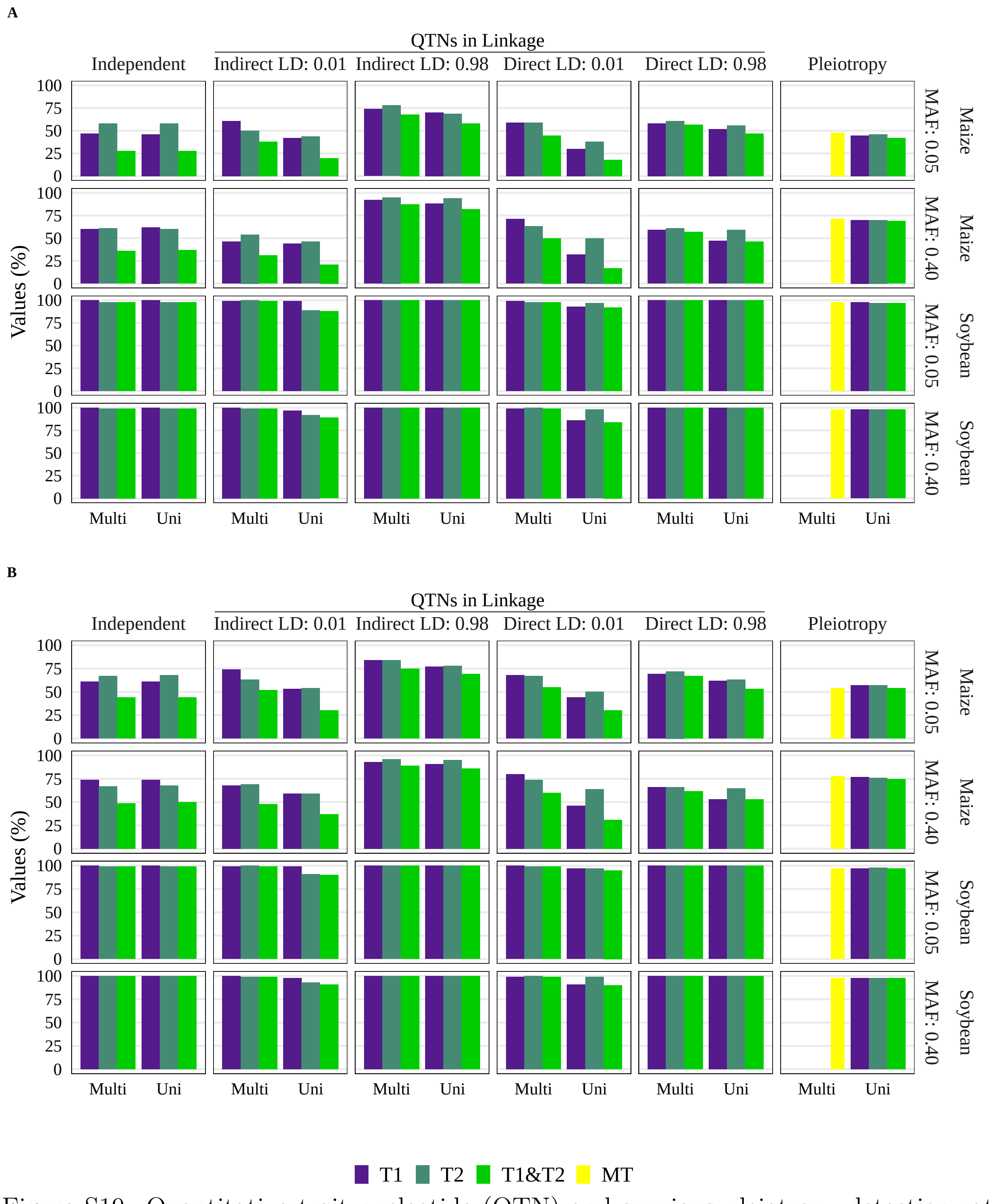

Figure S19: Quantitative trait nucleotide (QTN) and spurious pleiotropy detection rate (Y-axis) achieved by multivariate (Multi) and univariate (Uni) GWAS (X-axis), relative to the QTN controlling trait 1 (T1), trait 2 (T2), and both QTN simultaneously (T1&T2) or, in the pleiotropic scenario, relative to the pleiotropic QTN (MT). The simulated genetic architecture is listed in the horizontal and vertical titles. These values were obtained with a sample size of 2, 815; A) a narrow-sense heritability of 0.3 for both traits; B) a narrow-sense heritability of 0.8 for both traits. MAF: minor allele frequencies. The false discovery rate rate was 0.1 and the window size was 10 kb for maize and 1 Mb for soybean.

QTN detection with an FDR of 0.1 and window size of 1 Kb for maize and 10 Kb for soybean

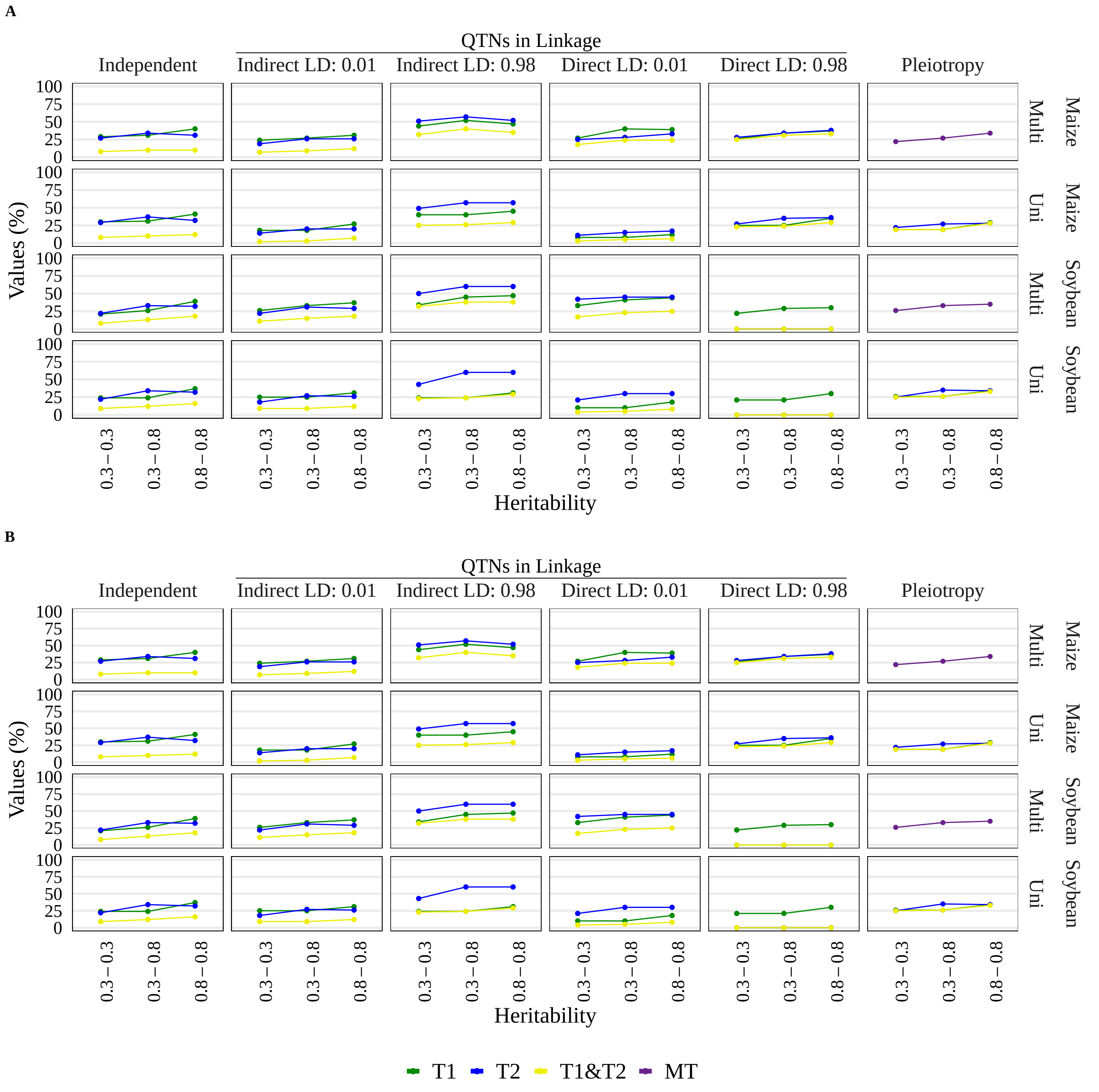

Figure S20: Quantitative trait nucleotide (QTN) and spurious pleiotropy detection rate (Y-axis) achieved by multivariate (Multi) and univariate (Uni) GWAS, relative to the QTN controlling trait 1 (T1), trait 2 (T2), and both QTN simultaneously (T1&T2) or, in the pleiotropic scenario, relative to the pleiotropic QTN (MT). These values were obtained for maize and soybean with a sample size of 500. The X-axis displays the narrow-sense heritability for Trait 1 (bottom value) and Trait 2 (top value). A) inputted minor allele frequency (MAF) of 0.05; B) MAF of 0.4. The false discovery rate rate was 0.1 and the window size was 1 kb for maize and 10 Kb for soybean.

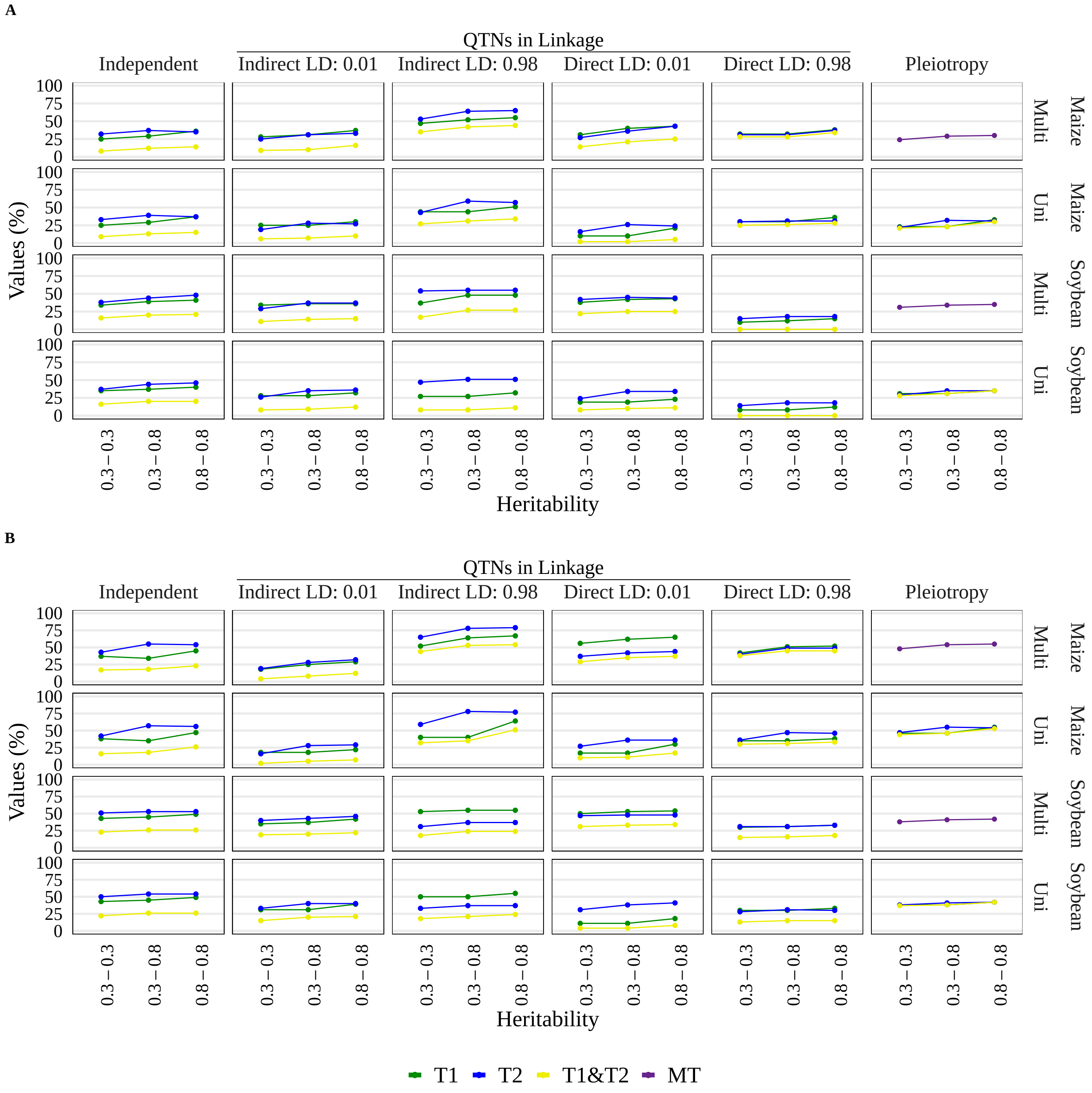

Figure S21: Quantitative trait nucleotide (QTN) and spurious pleiotropy detection rate (Y-axis) achieved by multivariate (Multi) and univariate (Uni) GWAS, relative to the QTN controlling trait 1 (T1), trait 2 (T2), and both QTN simultaneously (T1&T2) or, in the pleiotropic scenario, relative to the pleiotropic QTN (MT). These values were obtained for maize and soybean with a sample size of 1,000. The X-axis displays the narrow-sense heritability for Trait 1 (bottom value) and Trait 2 (top value). A) inputted minor allele frequency (MAF) of 0.05; B) MAF of 0.4. The false discovery rate rate was 0.1 and the window size was 1 kb for maize and 10 Kb for soybean.

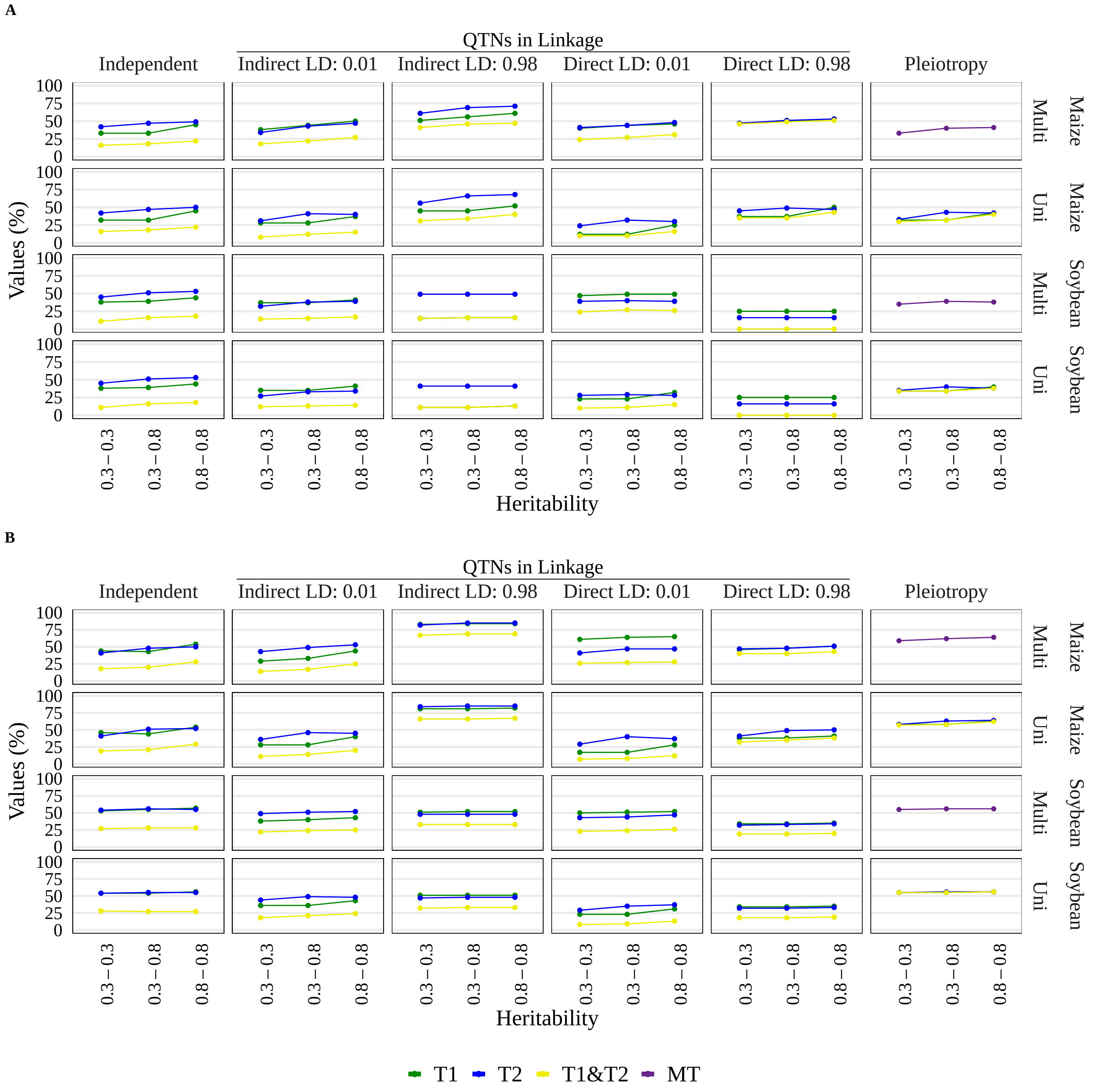

Figure S22: Quantitative trait nucleotide (QTN) and spurious pleiotropy detection rate (Y-axis) achieved by multivariate (Multi) and univariate (Uni) GWAS, relative to the QTN controlling trait 1 (T1), trait 2 (T2), and both QTN simultaneously (T1&T2) or, in the pleiotropic scenario, relative to the pleiotropic QTN (MT). These values were obtained for maize and soybean with a sample size of 2,815. The X-axis displays the narrow-sense heritability for Trait 1 (bottom value) and Trait 2 (top value). A) inputted minor allele frequency (MAF) of 0.05; B) MAF of 0.4. The false discovery rate rate was 0.1 and the window size was 1 kb for maize and 10 Kb for soybean.

A

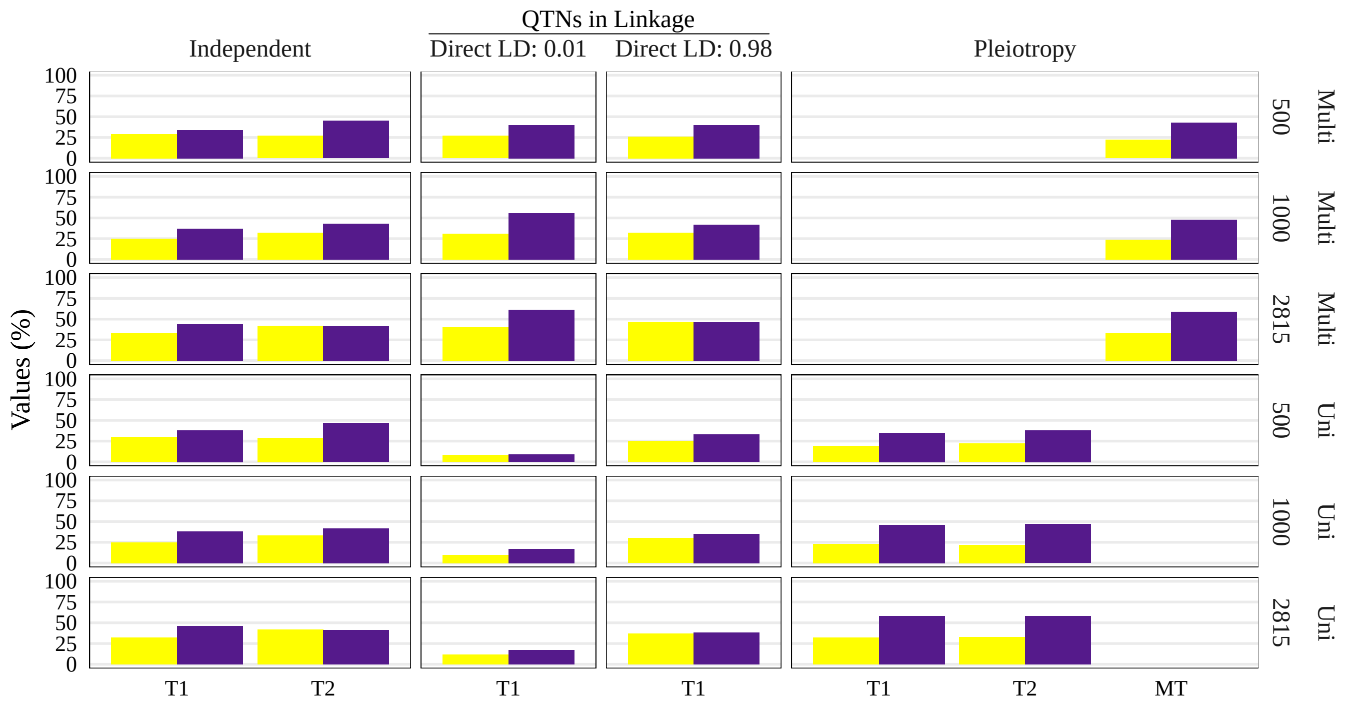

B

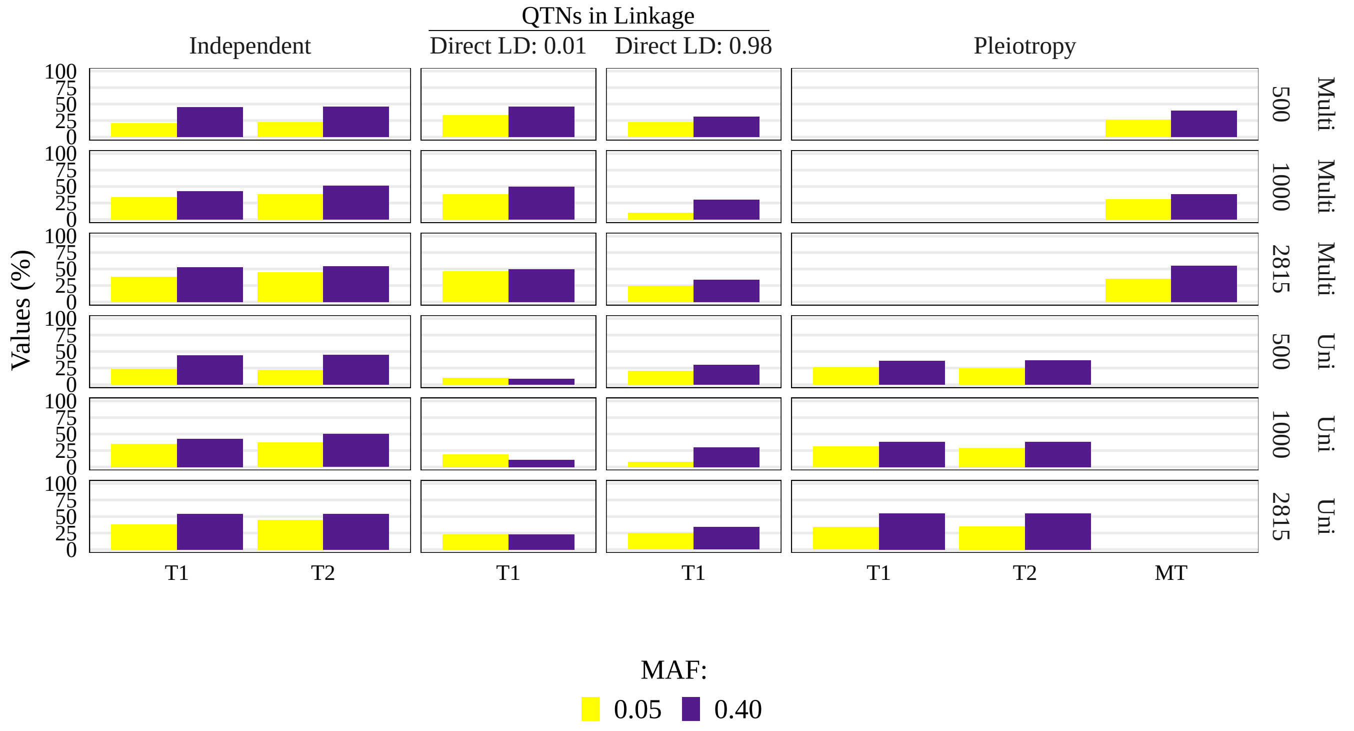

Figure S23: Quantitative trait nucleotide (QTN) and spurious pleiotropy detection rate (Y-axis) in scenarios for which minor allele frequency (MAF) was directly controlled by a simulation input parameter. These values were obtained by multivariate (Multi) and univariate (Uni) GWAS, relative to the QTN controlling trait 1 (T1), trait 2 (T2) or, in the pleiotropic scenario, relative to the pleiotropic QTN (MT). This figure shows results for a narrow-sense heritability of 0.3 for both traits. A) Maize; B) Soybean. The false discovery rate rate was 0.1 and the window size was 1 kb for maize and 10 Kb for soybean.

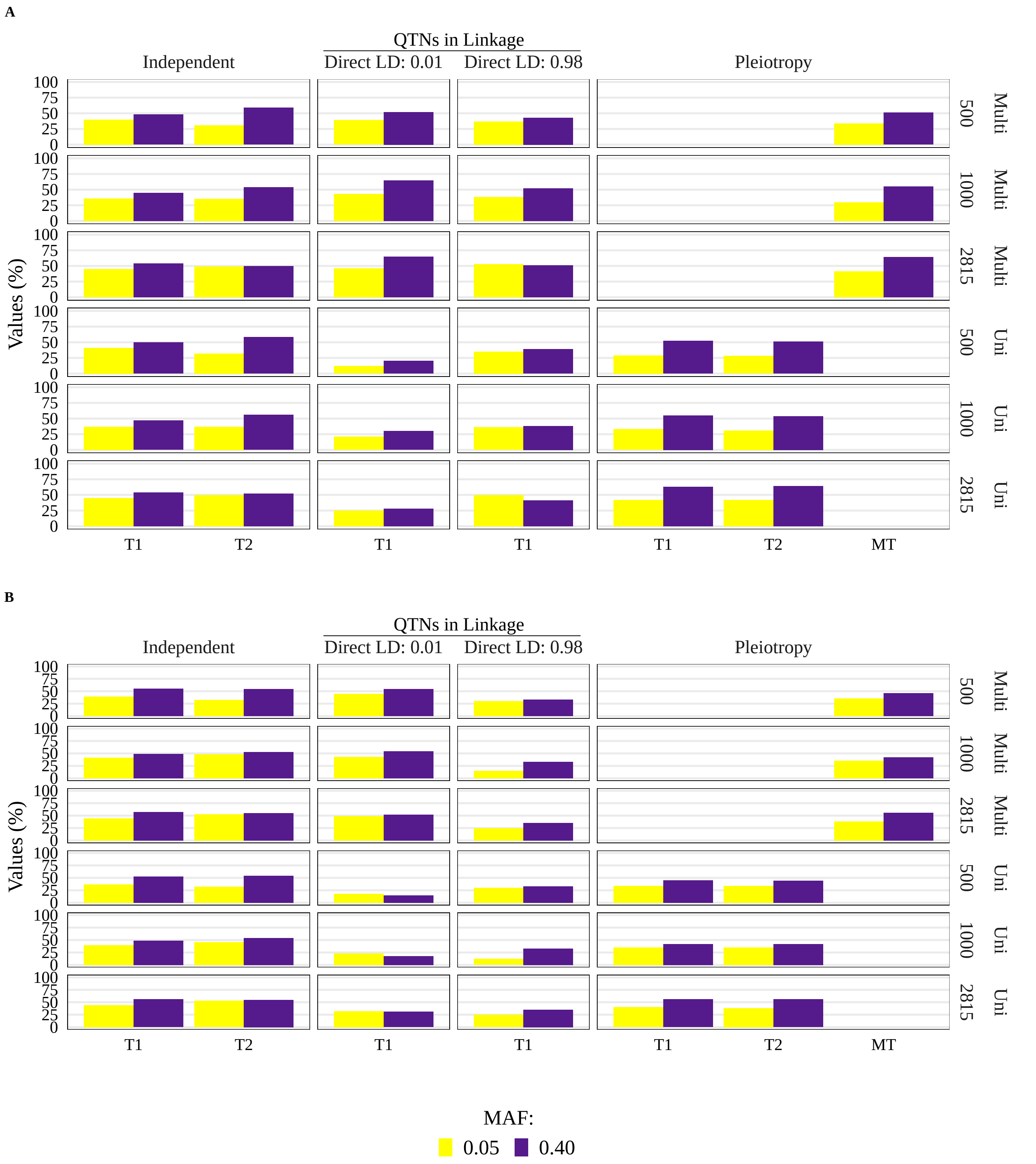

Figure S24: Quantitative trait nucleotide (QTN) and spurious pleiotropy detection rate (Y-axis) in scenarios for which minor allele frequency (MAF) was directly controlled by a simulation input parameter. These values were obtained by multivariate (Multi) and univariate (Uni) GWAS, relative to the QTN controlling trait 1 (T1), trait 2 (T2) or, in the pleiotropic scenario, relative to the pleiotropic QTN (MT). This figure shows results for a narrow-sense heritability of 0.8 for both traits. A) Maize; B) Soybean. The false discovery rate rate was 0.1 and the window size was 1 kb for maize and 10 Kb for soybean.

A

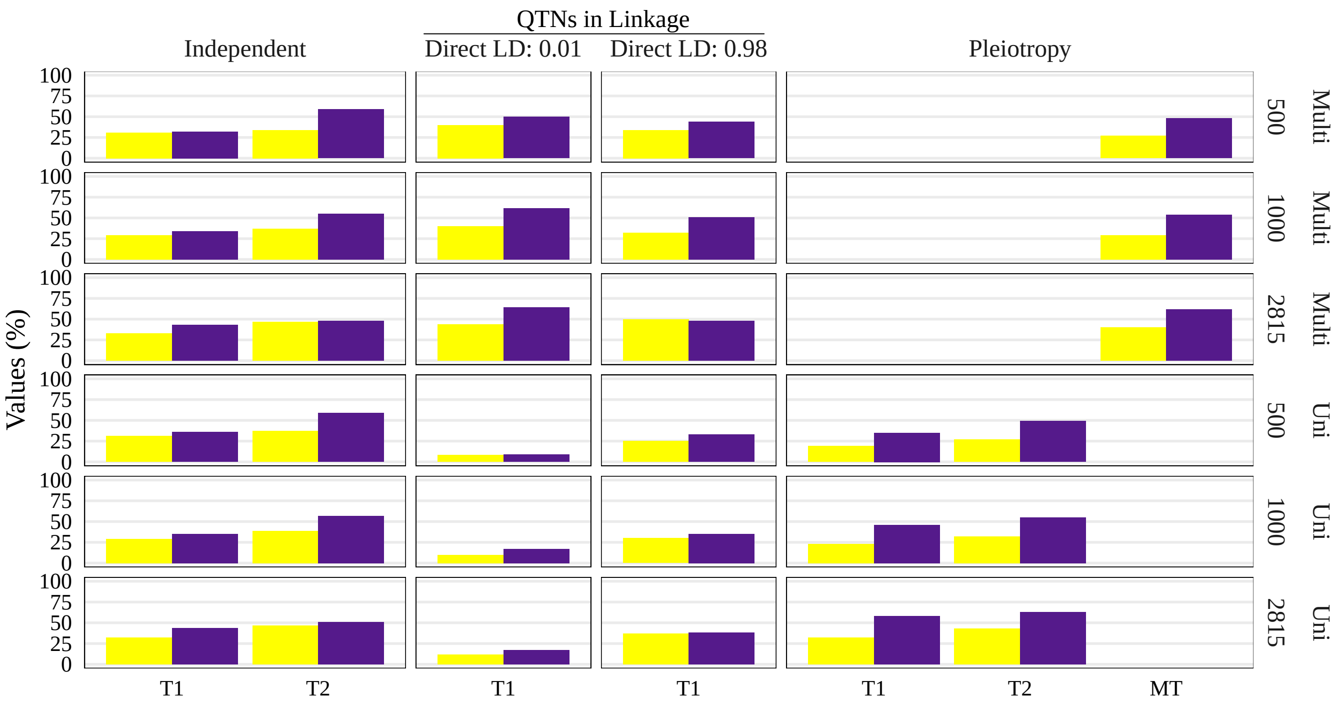

B

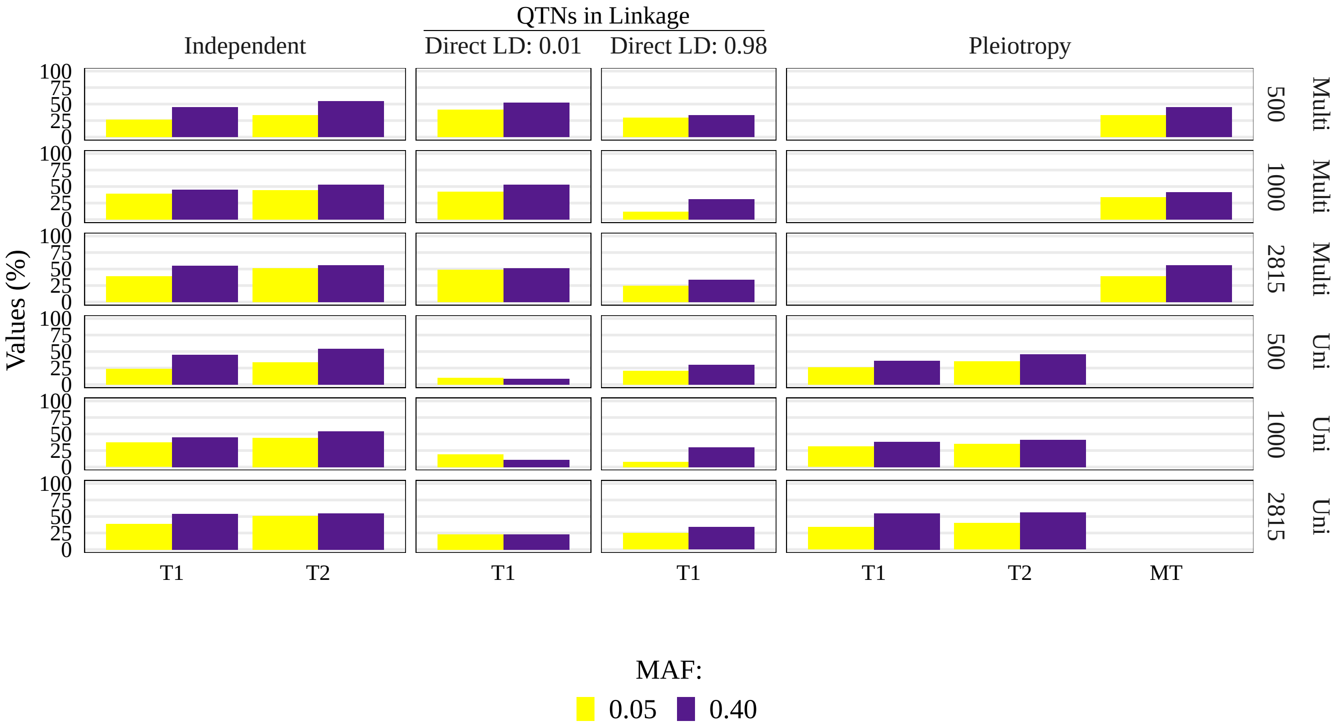

Figure S25: Quantitative trait nucleotide (QTN) and spurious pleiotropy detection rate (Y-axis) in scenarios for which minor allele frequency (MAF) was directly controlled by a simulation input parameter. These values were obtained by multivariate (Multi) and univariate (Uni) GWAS, relative to the QTN controlling trait 1 (T1), trait 2 (T2) or, in the pleiotropic scenario, relative to the pleiotropic QTN (MT). This figure shows results for a narrow-sense heritability of 0.3 for trait 1 and 0.8 for trait 2. A) Maize; B) Soybean. The false discovery rate rate was 0.1 and the window size was 1 kb for maize and 10 Kb for soybean.

A

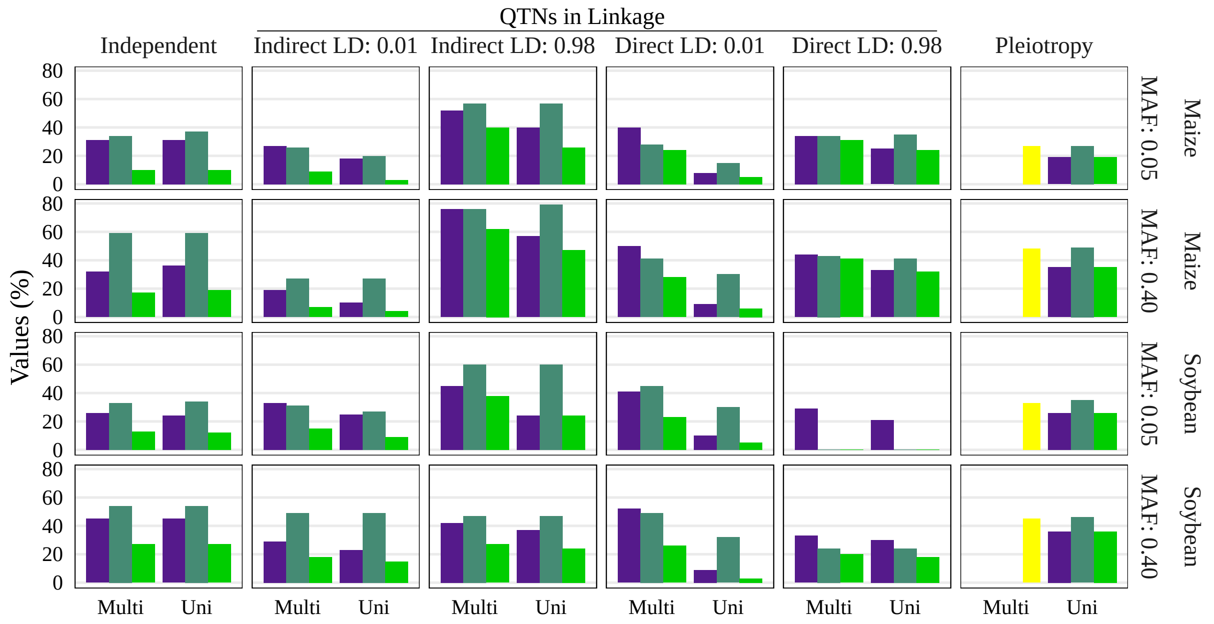

B

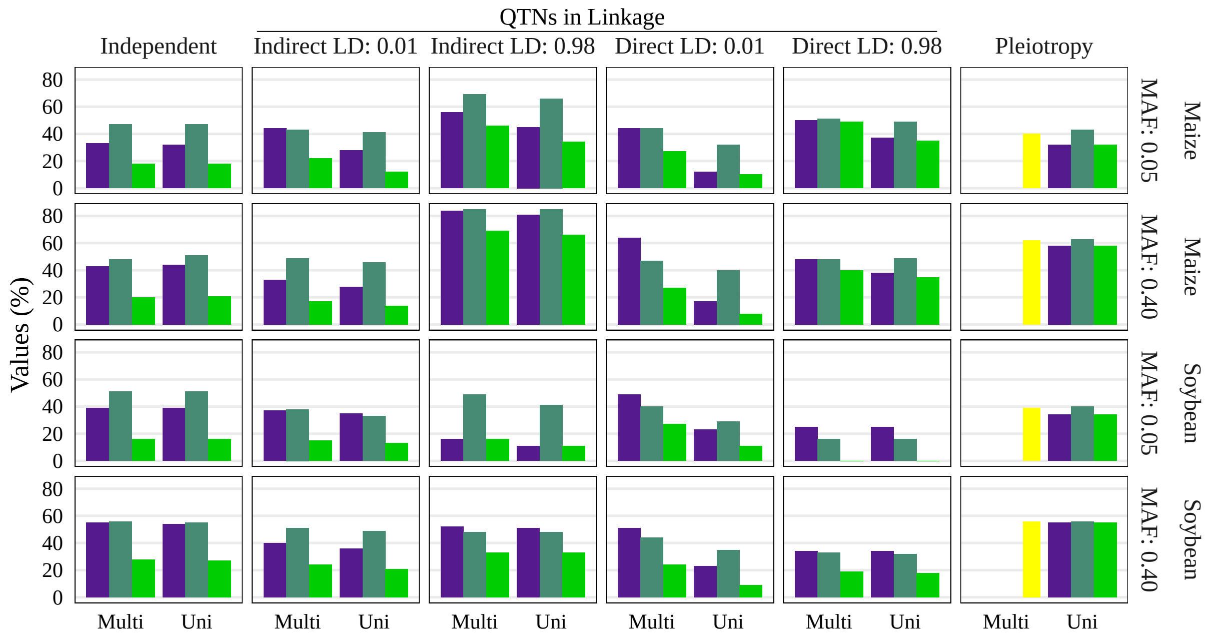

Figure S26: Quantitative trait nucleotide (QTN) and spurious pleiotropy detection rate (Y-axis) achieved by multivariate (Multi) and univariate (Uni) GWAS (X-axis), relative to the QTN controlling trait 1 (T1), trait 2 (T2), and both QTN simultaneously (T1&T2) or, in the pleiotropic scenario, relative to the pleiotropic QTN (MT). The simulated genetic architecture is listed in the horizontal and vertical titles. These values were obtained with a narrow-sense heritability of 0.3 and 0.8 for traits 1 and 2, respectively. A) Sample size of 500; B) Sample size of 2815. MAF: minor allele frequencies. The false discovery rate rate was 0.1 and the window size was 1 kb for maize and 10 Kb for soybean.

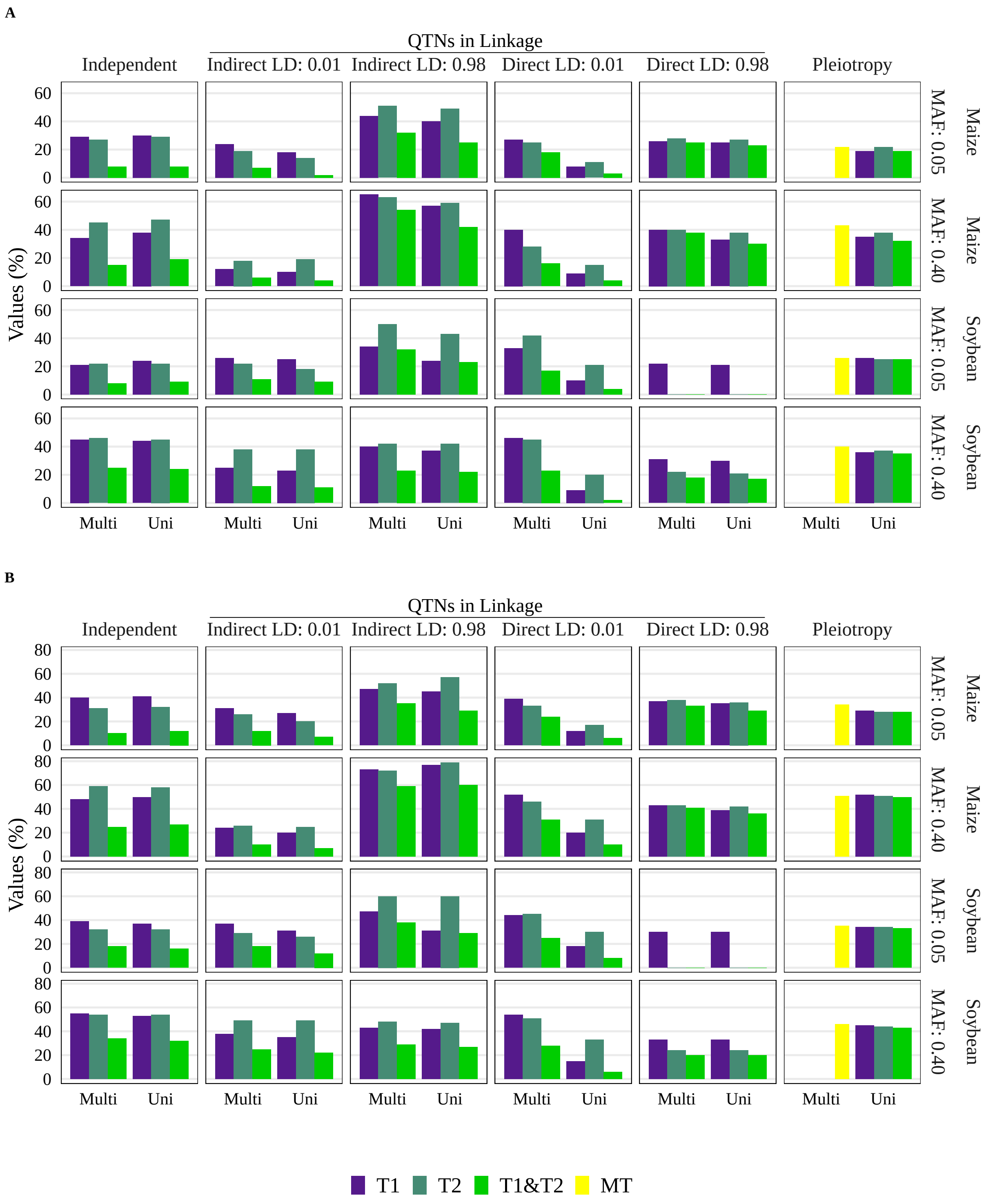

Figure S27: Quantitative trait nucleotide (QTN) and spurious pleiotropy detection rate (Y-axis) achieved by multivariate (Multi) and univariate (Uni) GWAS (X-axis), relative to the QTN controlling trait 1 (T1), trait 2 (T2), and both QTN simultaneously (T1&T2) or, in the pleiotropic scenario, relative to the pleiotropic QTN (MT). The simulated genetic architecture is listed in the horizontal and vertical titles. These values were obtained with a sample size of 500; A) a narrow-sense heritability of 0.3 for both traits; B) a narrow-sense heritability of 0.8 for both traits. MAF: minor allele frequencies. The false discovery rate rate was 0.1 and the window size was 1 kb for maize and 10 Kb for soybean.

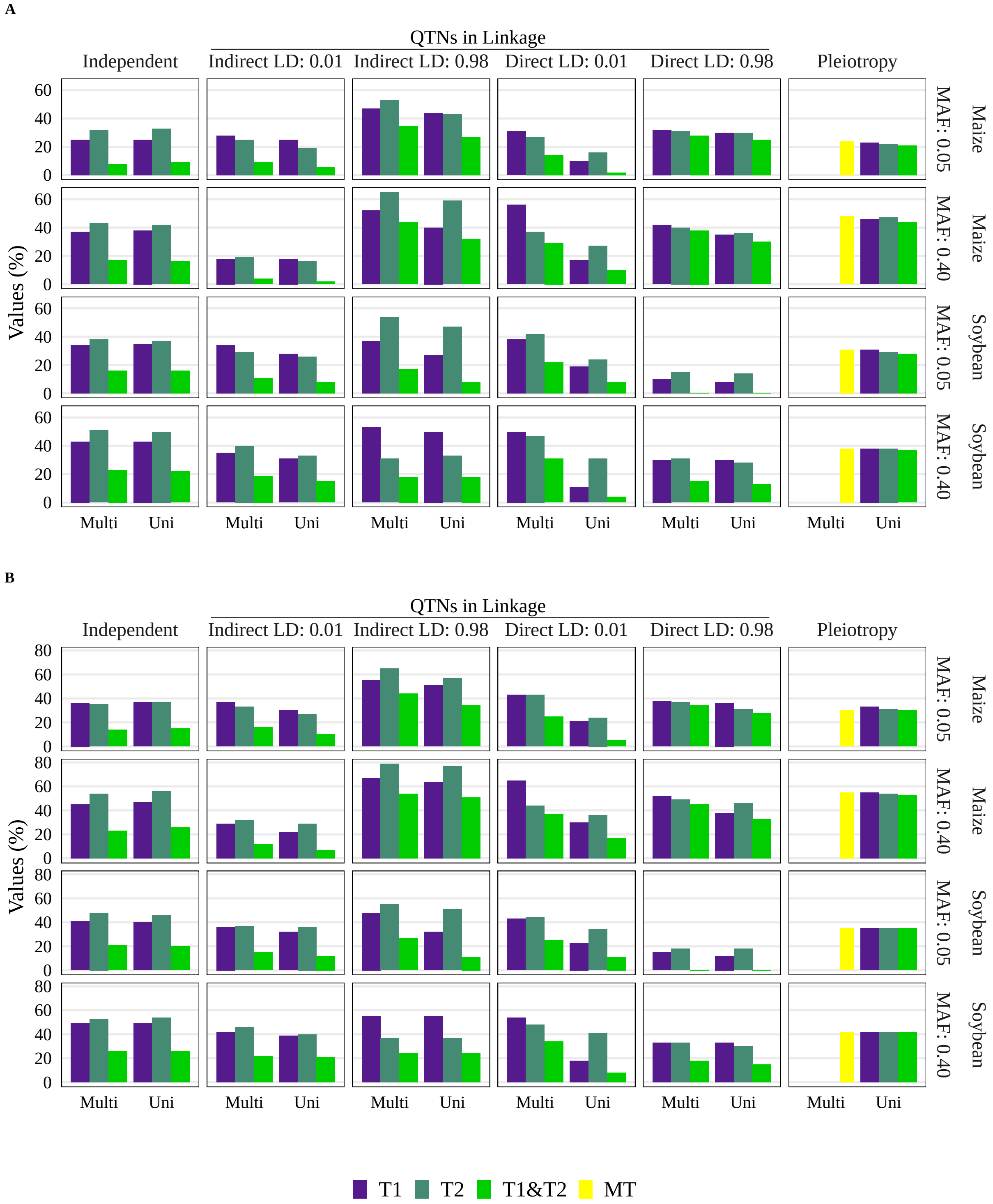

Figure S28: Quantitative trait nucleotide (QTN) and spurious pleiotropy detection rate (Y-axis) achieved by multivariate (Multi) and univariate (Uni) GWAS (X-axis), relative to the QTN controlling trait 1 (T1), trait 2 (T2), and both QTN simultaneously (T1&T2) or, in the pleiotropic scenario, relative to the pleiotropic QTN (MT). The simulated genetic architecture is listed in the horizontal and vertical titles. These values were obtained with a sample size of 1, 000; A) a narrow-sense heritability of 0.3 for both traits; B) a narrow-sense heritability of 0.8 for both traits. MAF: minor allele frequencies. The false discovery rate rate was 0.1 and the window size was 1 kb for maize and 10 Kb for soybean.

A

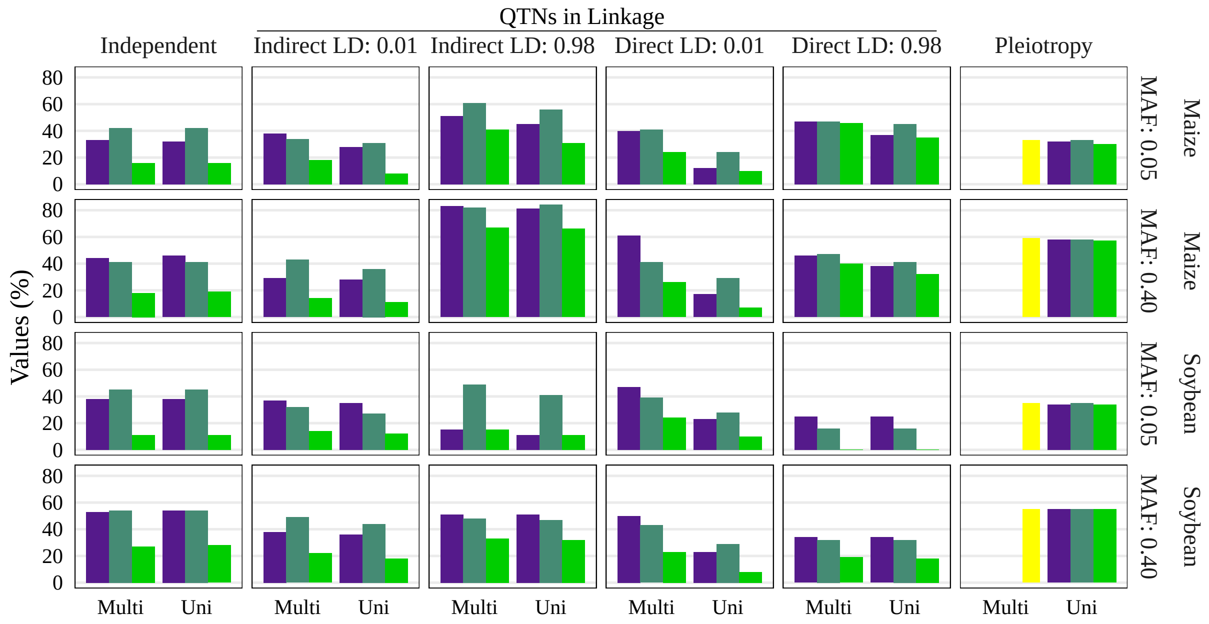

B

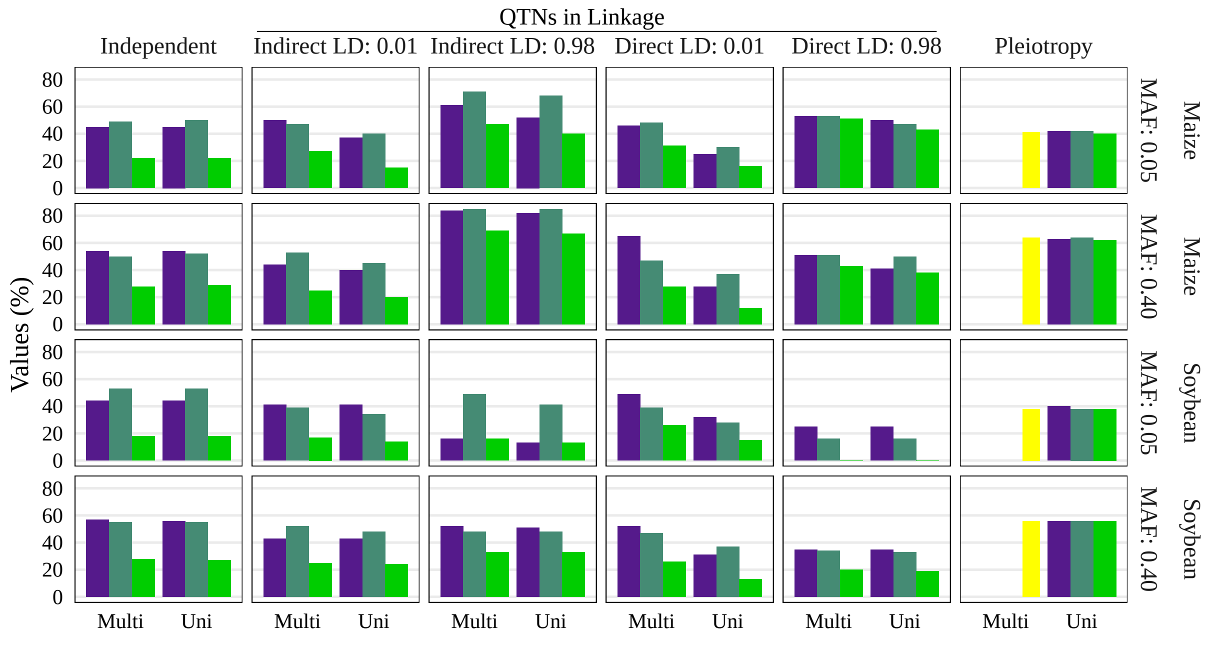

Figure S29: Quantitative trait nucleotide (QTN) and spurious pleiotropy detection rate (Y-axis) achieved by multivariate (Multi) and univariate (Uni) GWAS (X-axis), relative to the QTN controlling trait 1 (T1), trait 2 (T2), and both QTN simultaneously (T1&T2) or, in the pleiotropic scenario, relative to the pleiotropic QTN (MT). The simulated genetic architecture is listed in the horizontal and vertical titles. These values were obtained with a sample size of 2, 815; A) a narrow-sense heritability of 0.3 for both traits; B) a narrow-sense heritability of 0.8 for both traits. MAF: minor allele frequencies. The false discovery rate rate was 0.1 and the window size was 1 kb for maize and 10 Kb for soybean.

QTN detection with an FDR of 0.05 and window size of 10 Kb for maize and 1Mb for soybean

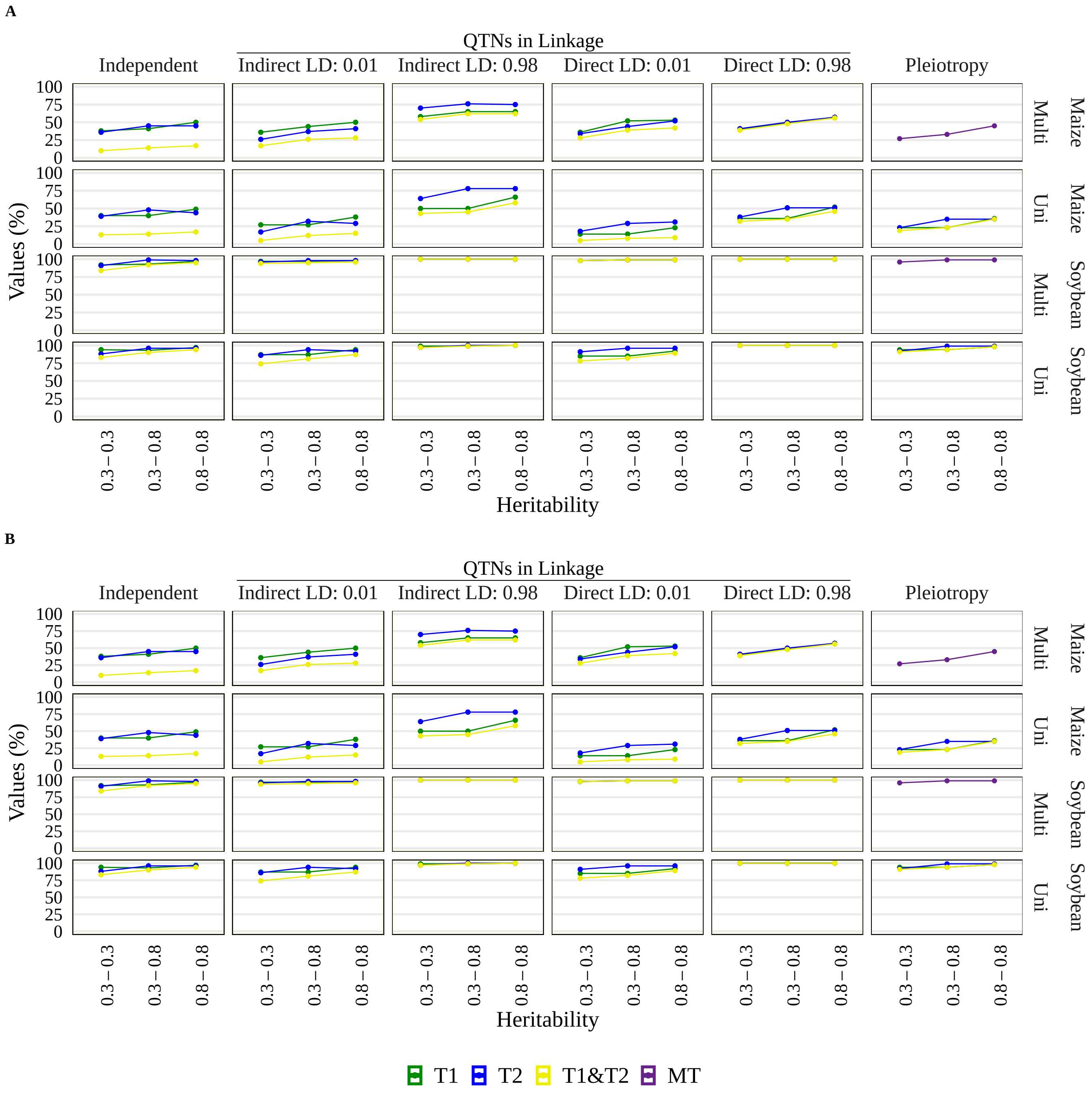

Figure S30: Quantitative trait nucleotide (QTN) and spurious pleiotropy detection rate (Y-axis) achieved by multivariate (Multi) and univariate (Uni) GWAS, relative to the QTN controlling trait 1 (T1), trait 2 (T2), and both QTN simultaneously (T1&T2) or, in the pleiotropic scenario, relative to the pleiotropic QTN (MT). These values were obtained for maize and soybean with a sample size of 500. The X-axis displays the narrow-sense heritability for Trait 1 (bottom value) and Trait 2 (top value). A) inputted minor allele frequency (MAF) of 0.05; B) MAF of 0.4. The false discovery rate rate was 0.05 and the window size was 10 kb for maize and 1 Mb for soybean.

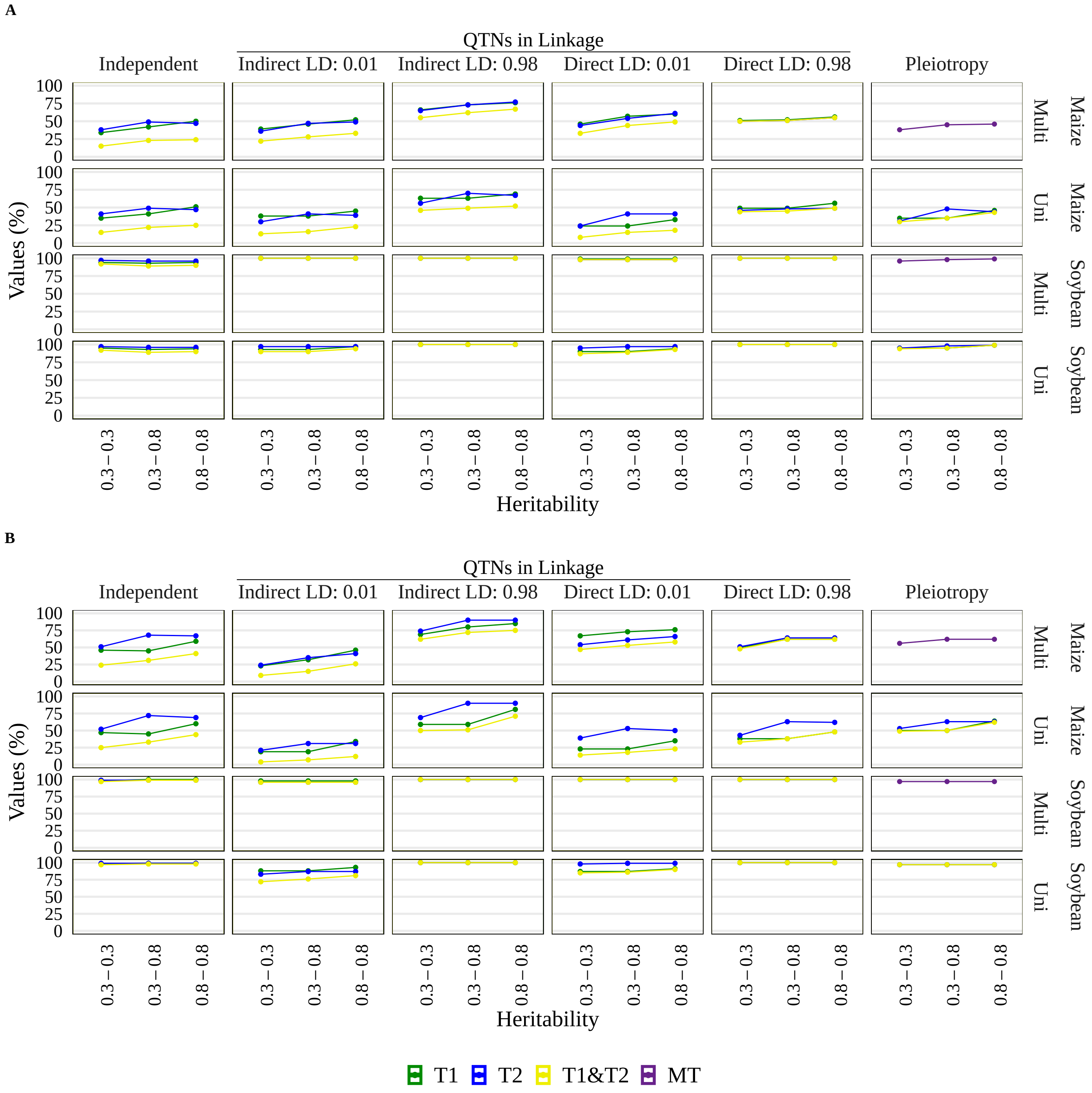

Figure S31: Quantitative trait nucleotide (QTN) and spurious pleiotropy detection rate (Y-axis) achieved by multivariate (Multi) and univariate (Uni) GWAS, relative to the QTN controlling trait 1 (T1), trait 2 (T2), and both QTN simultaneously (T1&T2) or, in the pleiotropic scenario, relative to the pleiotropic QTN (MT). These values were obtained for maize and soybean with a sample size of 1, 000. The X-axis displays the narrow-sense heritability for Trait 1 (bottom value) and Trait 2 (top value). A) inputted minor allele frequency (MAF) of 0.05; B) MAF of 0.4. The false discovery rate rate was 0.05 and the window size was 10 kb for maize and 1 Mb for soybean.

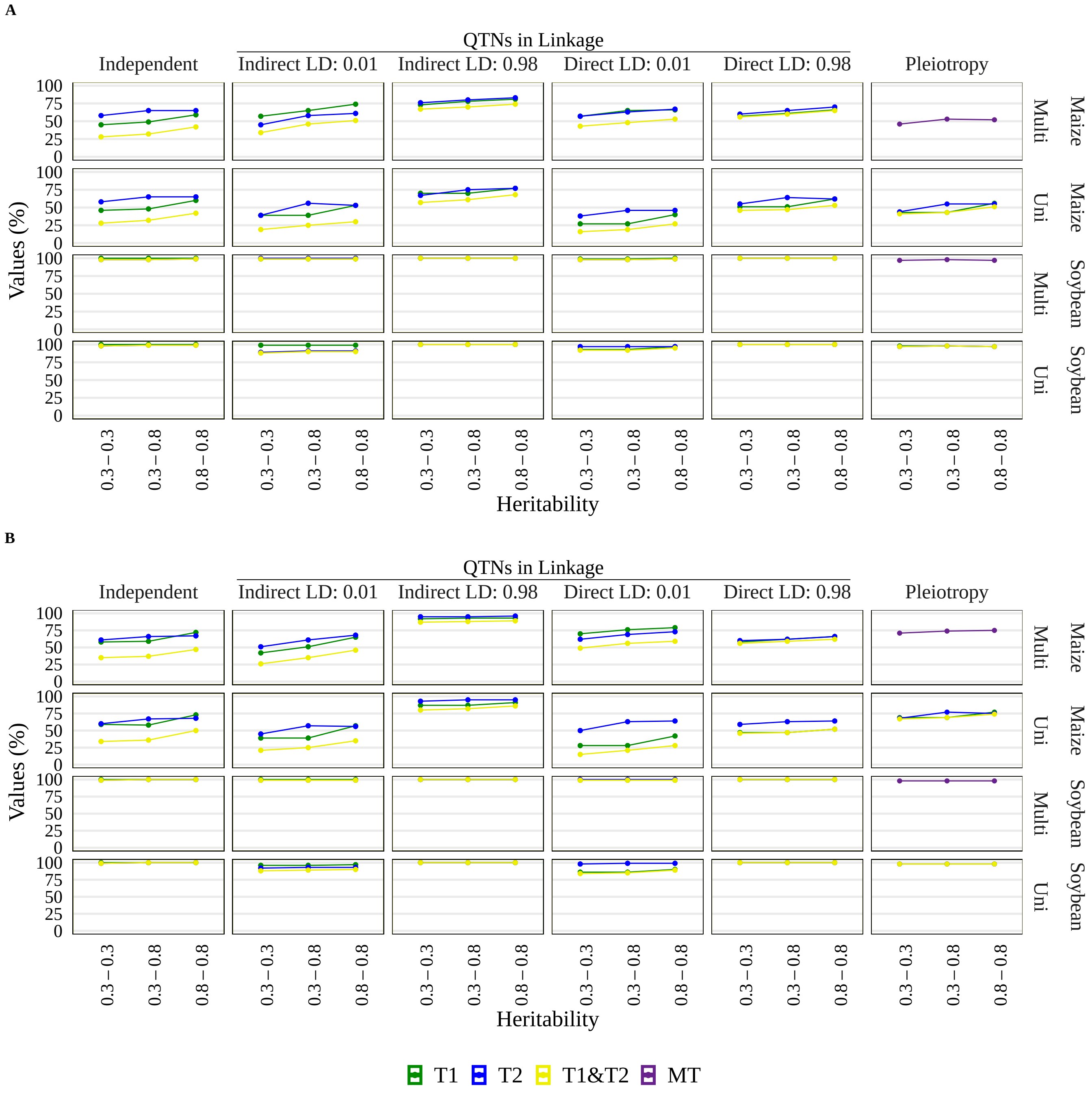

Figure S32: Quantitative trait nucleotide (QTN) and spurious pleiotropy detection rate (Y-axis) achieved by multivariate (Multi) and univariate (Uni) GWAS, relative to the QTN controlling trait 1 (T1), trait 2 (T2), and both QTN simultaneously (T1&T2) or, in the pleiotropic scenario, relative to the pleiotropic QTN (MT). These values were obtained for maize and soybean with a sample size of 2, 815. The X-axis displays the narrow-sense heritability for Trait 1 (bottom value) and Trait 2 (top value). A) inputted minor allele frequency (MAF) of 0.05; B) MAF of 0.4. The false discovery rate rate was 0.05 and the window size was 10 kb for maize and 1 Mb for soybean.

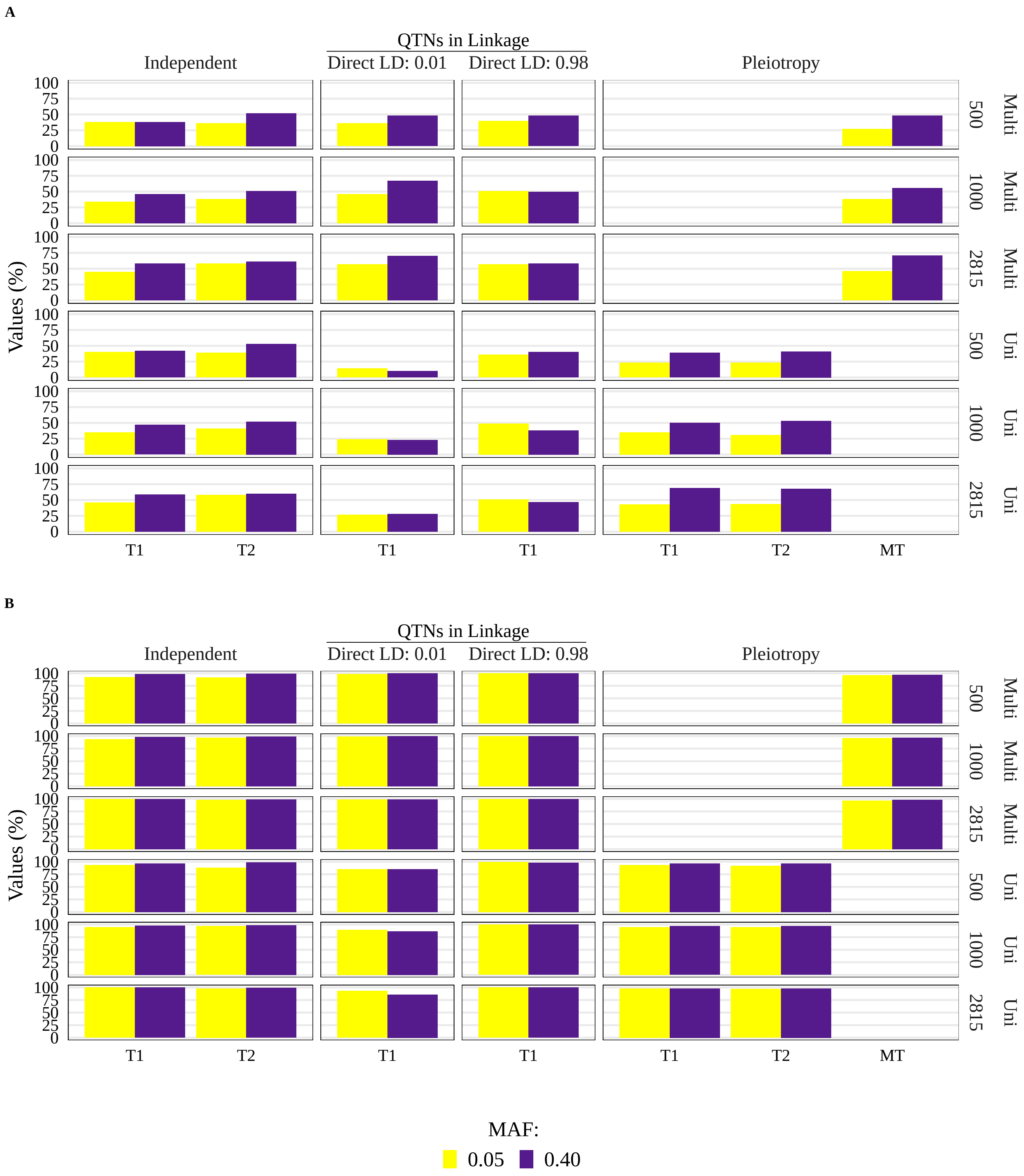

Figure S33: Quantitative trait nucleotide (QTN) and spurious pleiotropy detection rate (Y-axis) in scenarios for which minor allele frequency (MAF) was directly controlled by a simulation input parameter. These values were obtained by multivariate (Multi) and univariate (Uni) GWAS, relative to the QTN controlling trait 1 (T1), trait 2 (T2) or, in the pleiotropic scenario, relative to the pleiotropic QTN (MT). This figure shows results for a narrow-sense heritability of 0.3 for both traits. A) Maize; B) Soybean. The false discovery rate rate was 0.05 and the window size was 10 kb for maize and 1 Mb for soybean.

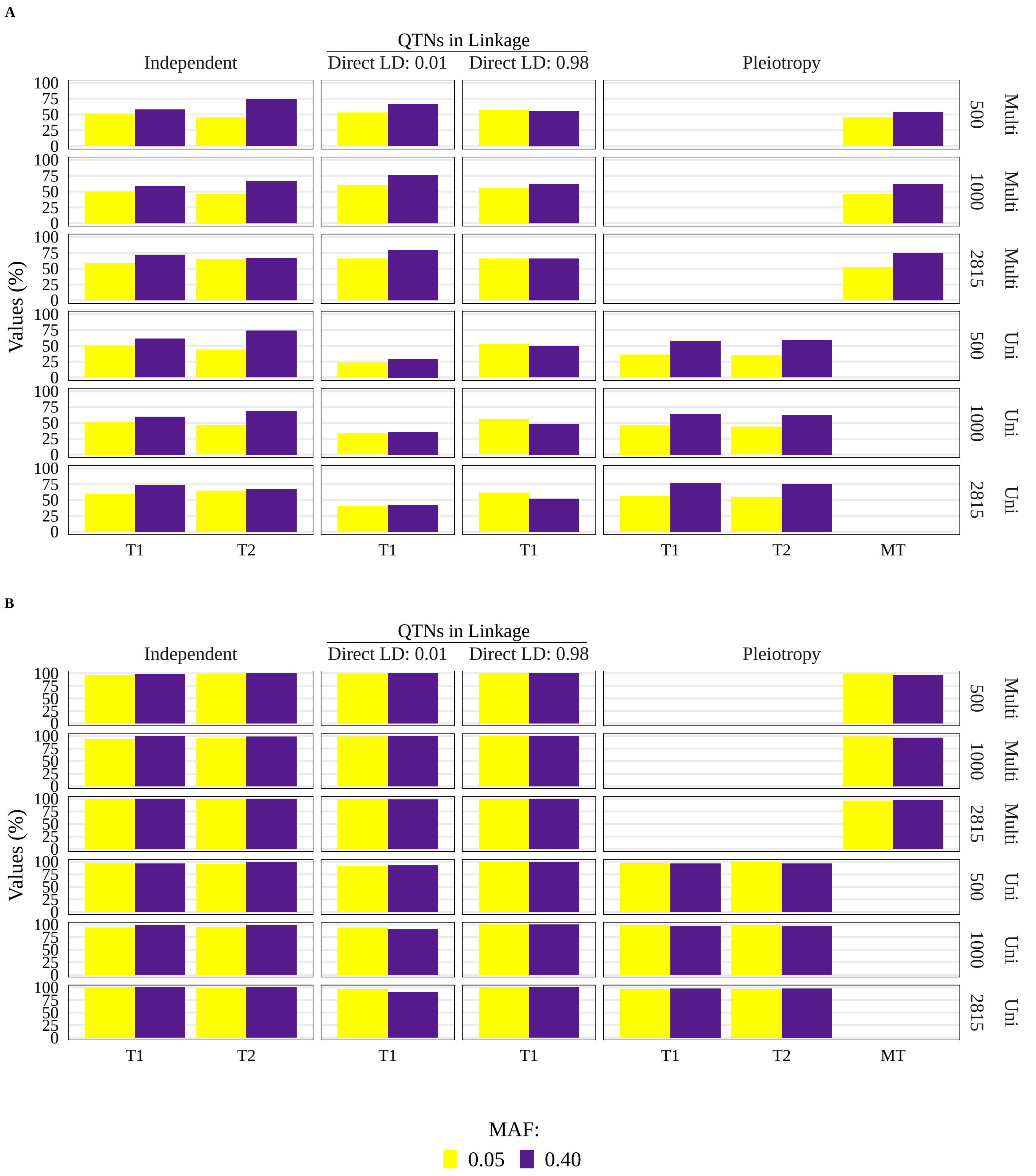

Figure S34: Quantitative trait nucleotide (QTN) and spurious pleiotropy detection rate (Y-axis) in scenarios for which minor allele frequency (MAF) was directly controlled by a simulation input parameter. These values were obtained by multivariate (Multi) and univariate (Uni) GWAS, relative to the QTN controlling trait 1 (T1), trait 2 (T2) or, in the pleiotropic scenario, relative to the pleiotropic QTN (MT). This figure shows results for a narrow-sense heritability of 0.8 for both traits. A) Maize; B) Soybean. The false discovery rate rate was 0.05 and the window size was 10 kb for maize and 1 Mb for soybean.

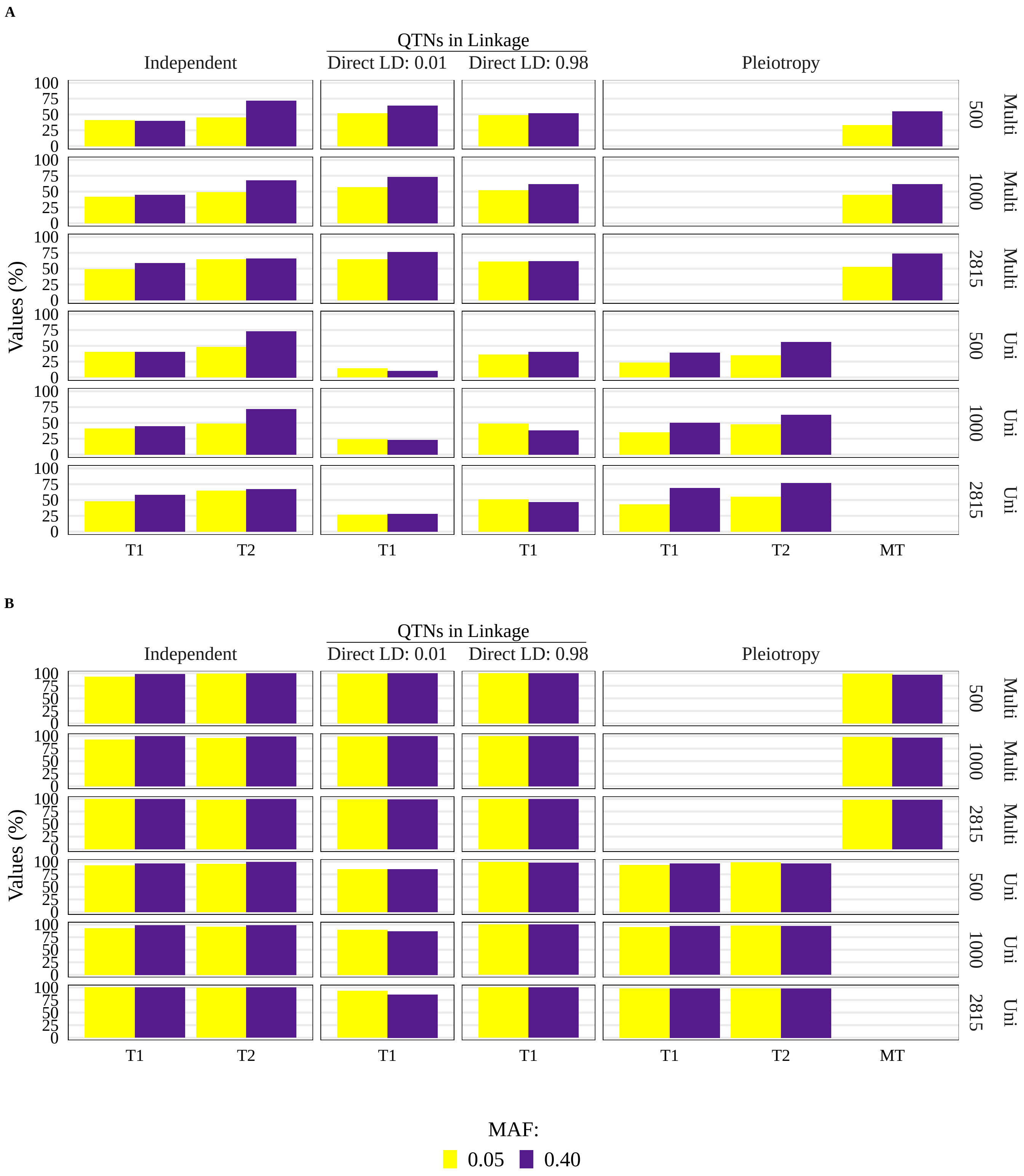

Figure S35: Quantitative trait nucleotide (QTN) and spurious pleiotropy detection rate (Y-axis) in scenarios for which minor allele frequency (MAF) was directly controlled by a simulation input parameter. These values were obtained by multivariate (Multi) and univariate (Uni) GWAS, relative to the QTN controlling trait 1 (T1), trait 2 (T2) or, in the pleiotropic scenario, relative to the pleiotropic QTN (MT). This figure shows results for a narrow-sense heritability of 0.3 for trait 1 and 0.8 for trait 2. A) Maize; B) Soybean. The false discovery rate rate was 0.05 and the window size was 10 kb for maize and 1 Mb for soybean.

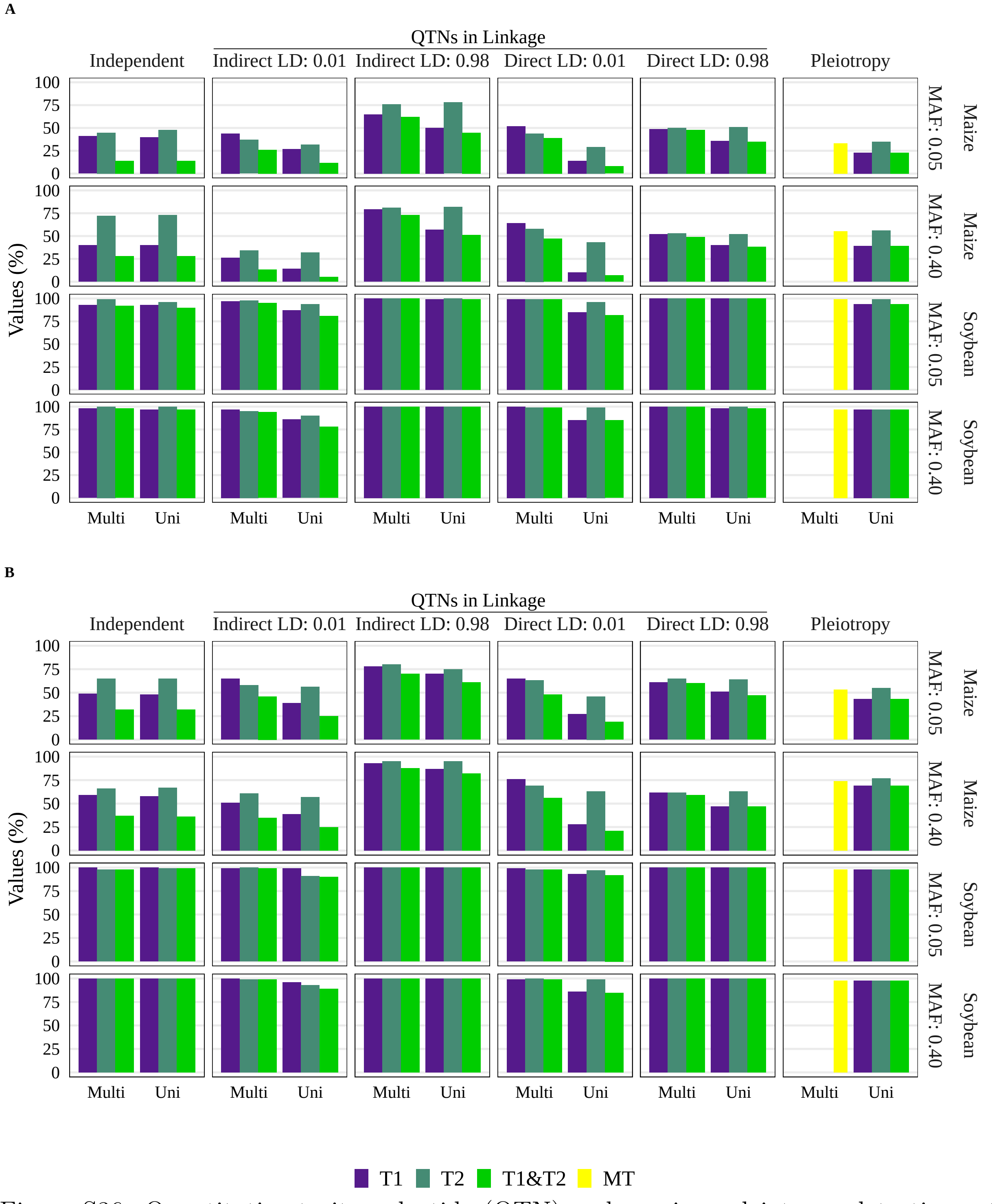

Figure S36: Quantitative trait nucleotide (QTN) and spurious pleiotropy detection rate (Y-axis) achieved by multivariate (Multi) and univariate (Uni) GWAS (X-axis), relative to the QTN controlling trait 1 (T1), trait 2 (T2), and both QTN simultaneously (T1&T2) or, in the pleiotropic scenario, relative to the pleiotropic QTN (MT). The simulated genetic architecture is listed in the horizontal and vertical titles. These values were obtained with a narrow-sense heritability of 0.3 and 0.8 for traits 1 and 2, respectively. A) Sample size of 500; B) Sample size of 2815. MAF: minor allele frequencies. The false discovery rate rate was 0.05 and the window size was 10 kb for maize and 1 Mb for soybean.

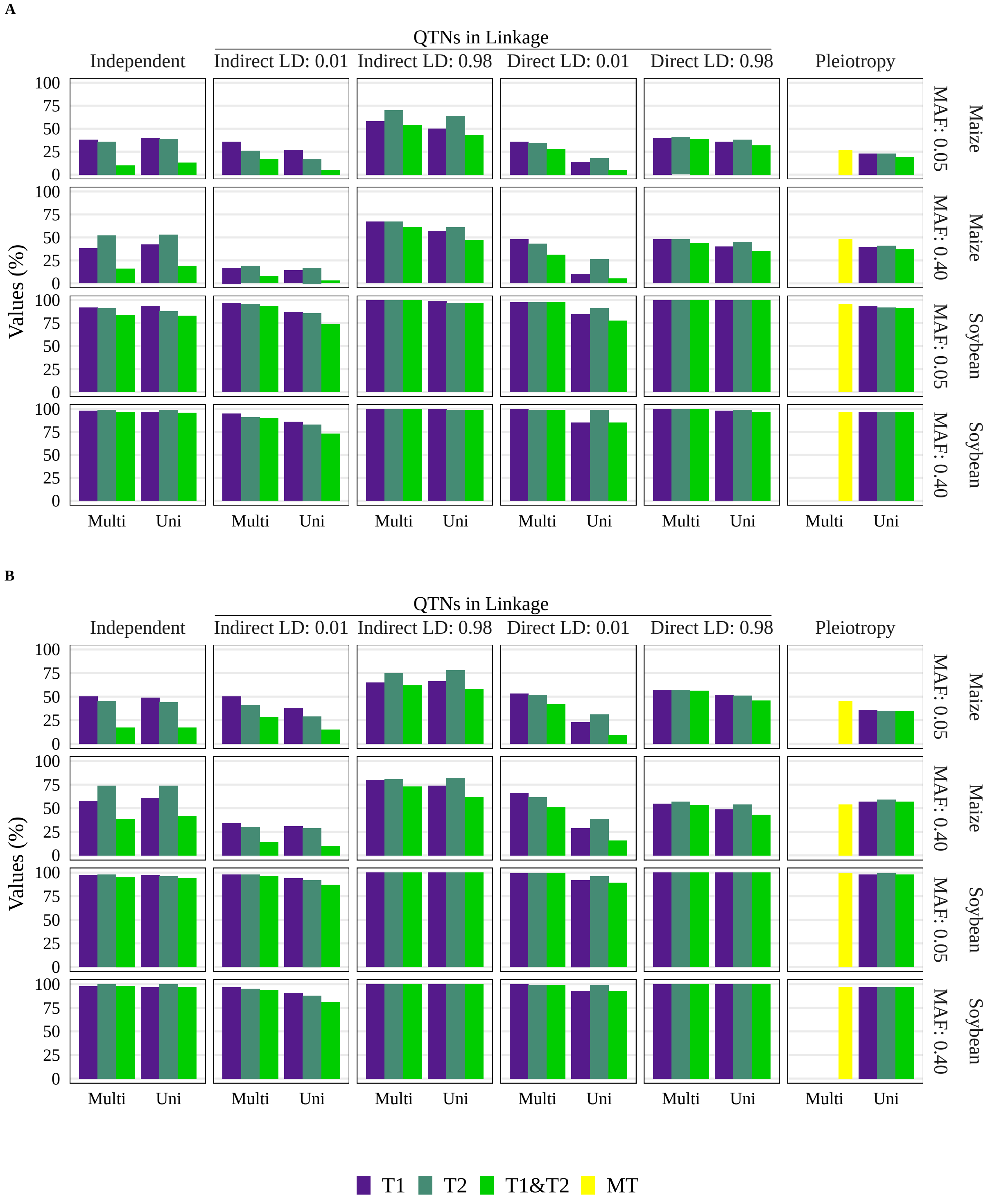

Figure S37: Quantitative trait nucleotide (QTN) and spurious pleiotropy detection rate (Y-axis) achieved by multivariate (Multi) and univariate (Uni) GWAS (X-axis), relative to the QTN controlling trait 1 (T1), trait 2 (T2), and both QTN simultaneously (T1&T2) or, in the pleiotropic scenario, relative to the pleiotropic QTN (MT). The simulated genetic architecture is listed in the horizontal and vertical titles. These values were obtained with a sample size of 500; A) a narrow-sense heritability of 0.3 for both traits; B) a narrow-sense heritability of 0.8 for both traits. MAF: minor allele frequencies. The false discovery rate rate was 0.05 and the window size was 10 kb for maize and 1 Mb for soybean.

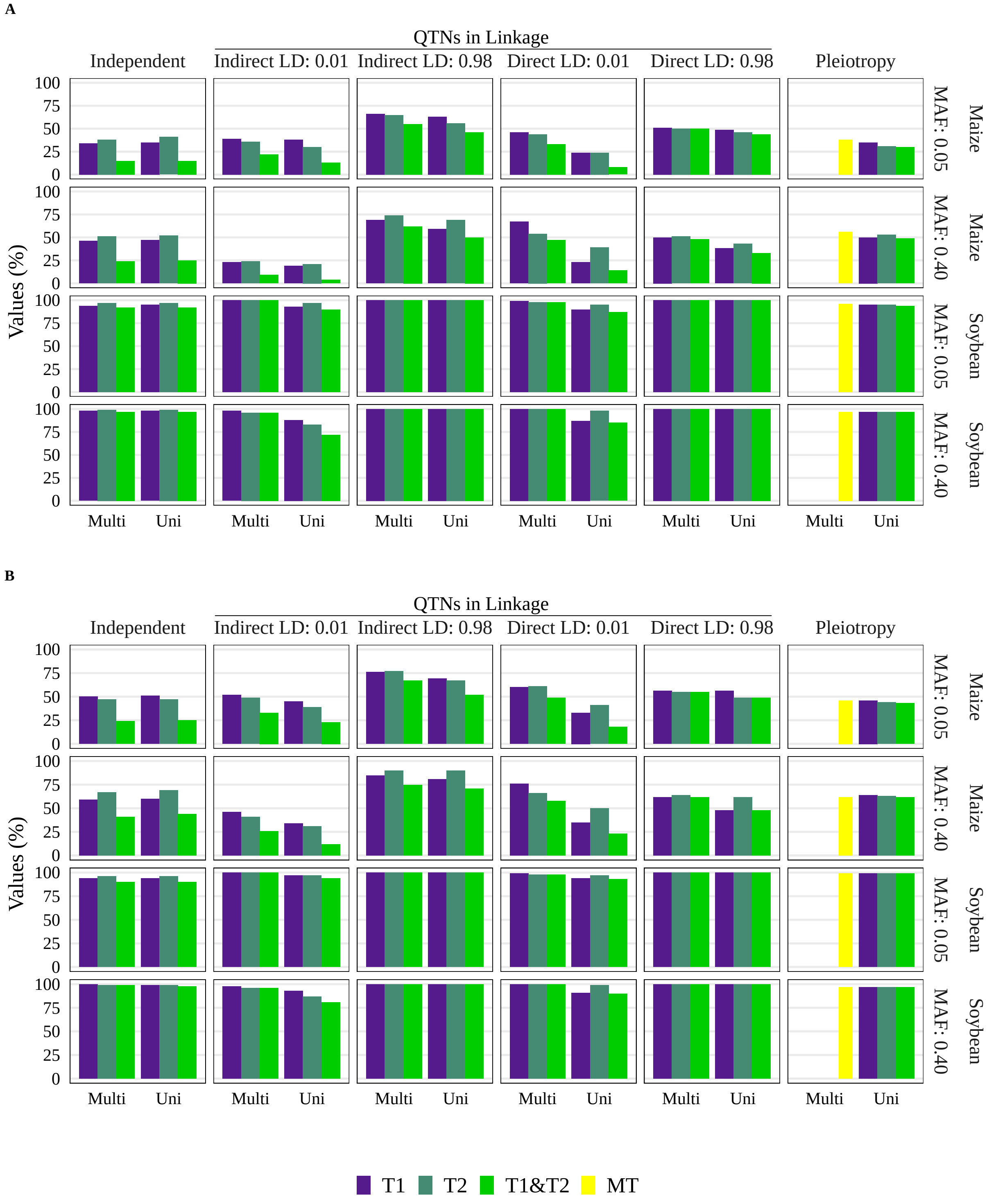

Figure S38: Quantitative trait nucleotide (QTN) and spurious pleiotropy detection rate (Y-axis) achieved by multivariate (Multi) and univariate (Uni) GWAS (X-axis), relative to the QTN controlling trait 1 (T1), trait 2 (T2), and both QTN simultaneously (T1&T2) or, in the pleiotropic scenario, relative to the pleiotropic QTN (MT). The simulated genetic architecture is listed in the horizontal and vertical titles. These values were obtained with a sample size of 1, 000; A) a narrow-sense heritability of 0.3 for both traits; B) a narrow-sense heritability of 0.8 for both traits. MAF: minor allele frequencies. The false discovery rate rate was 0.05 and the window size was 10 kb for maize and 1 Mb for soybean.

A

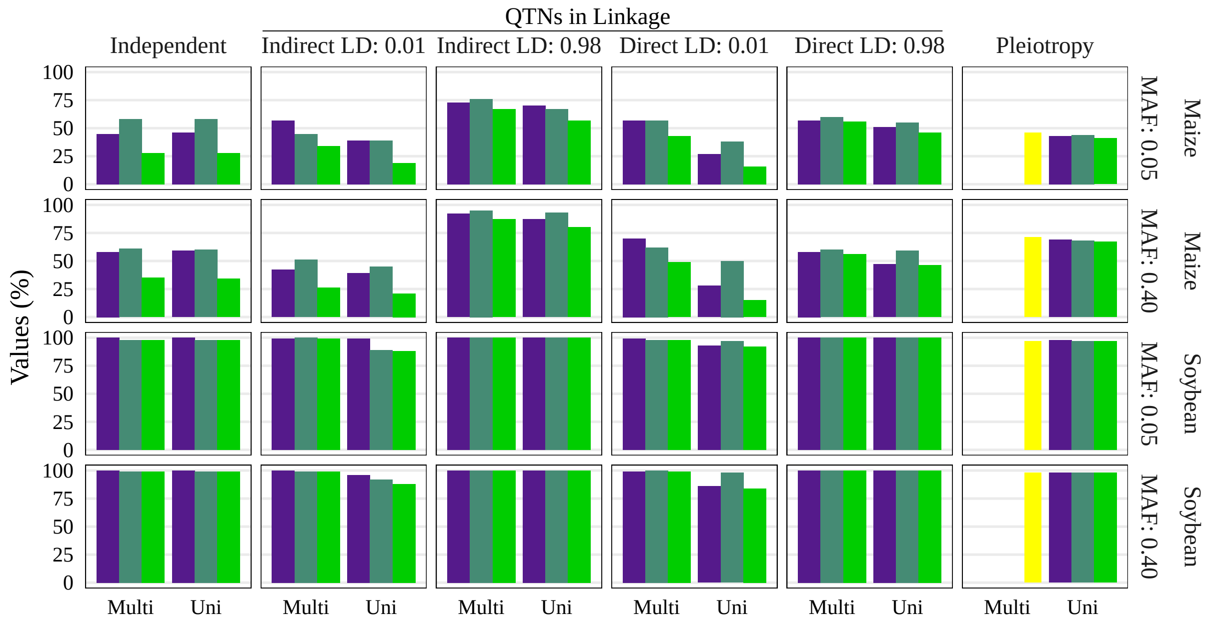

B

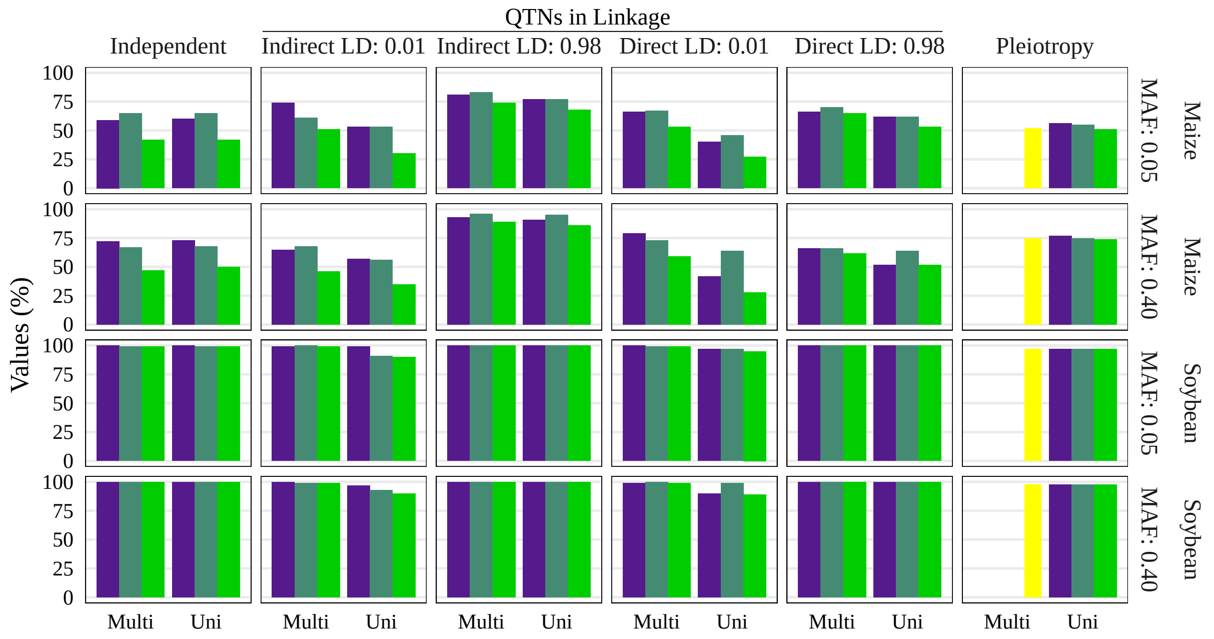

Figure S39: Quantitative trait nucleotide (QTN) and spurious pleiotropy detection rate (Y-axis) achieved by multivariate (Multi) and univariate (Uni) GWAS (X-axis), relative to the QTN controlling trait 1 (T1), trait 2 (T2), and both QTN simultaneously (T1&T2) or, in the pleiotropic scenario, relative to the pleiotropic QTN (MT). The simulated genetic architecture is listed in the horizontal and vertical titles. These values were obtained with a sample size of 2, 815; A) a narrow-sense heritability of 0.3 for both traits; B) a narrow-sense heritability of 0.8 for both traits. MAF: minor allele frequencies. The false discovery rate rate was 0.05 and the window size was 10 kb for maize and 1 Mb for soybean.

QTN detection with an FDR of 0.05 and window size of 1 Kb for maize and 10 Kb for soybean

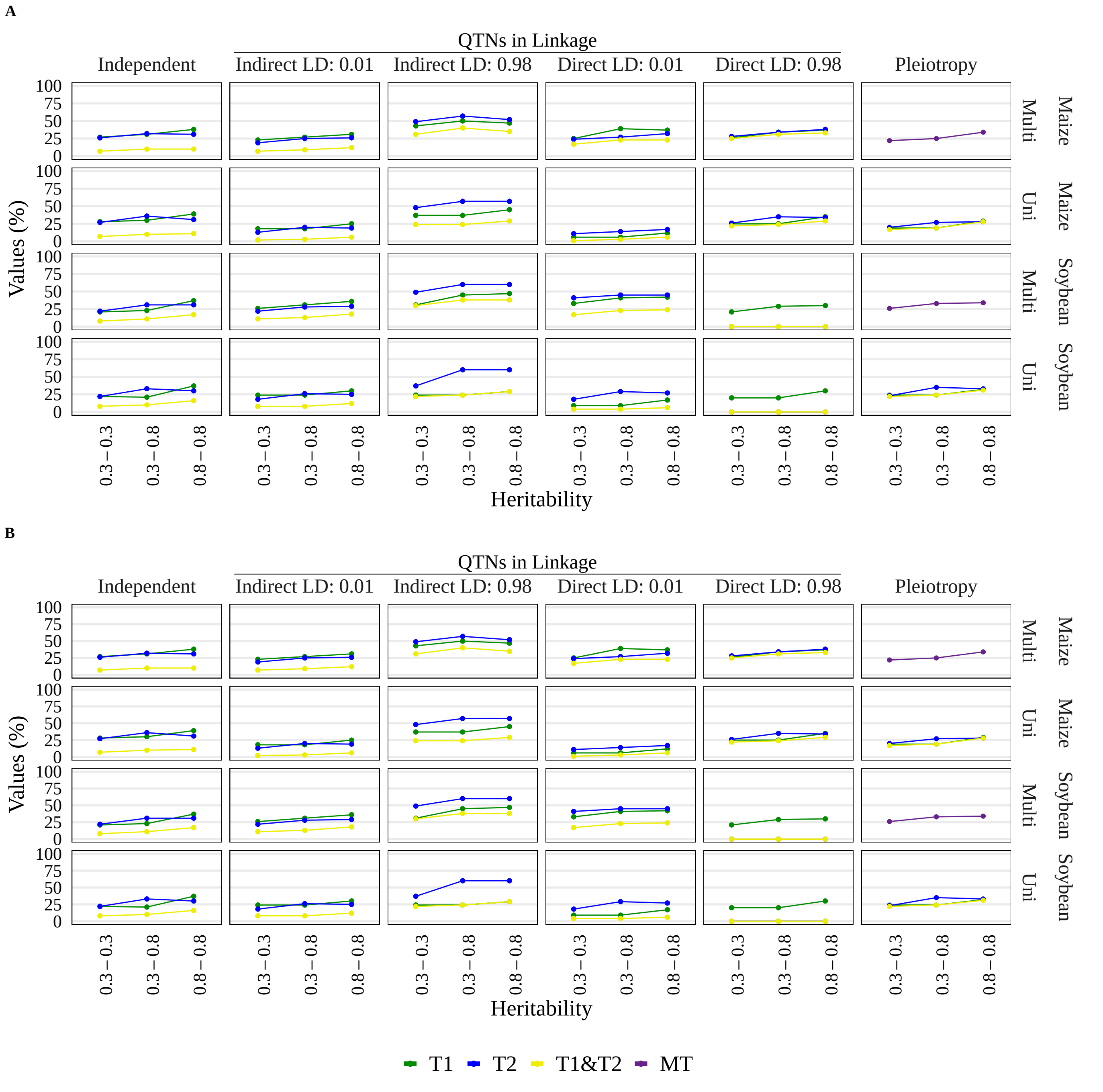

Figure S40: Quantitative trait nucleotide (QTN) and spurious pleiotropy detection rate (Y-axis) achieved by multivariate (Multi) and univariate (Uni) GWAS, relative to the QTN controlling trait 1 (T1), trait 2 (T2), and both QTN simultaneously (T1&T2) or, in the pleiotropic scenario, relative to the pleiotropic QTN (MT). These values were obtained for maize and soybean with a sample size of 500. The X-axis displays the narrow-sense heritability for Trait 1 (bottom value) and Trait 2 (top value). A) inputted minor allele frequency (MAF) of 0.05; B) MAF of 0.4. The false discovery rate rate was 0.05 and the window size was 1 kb for maize and 10 Kb for soybean.

A

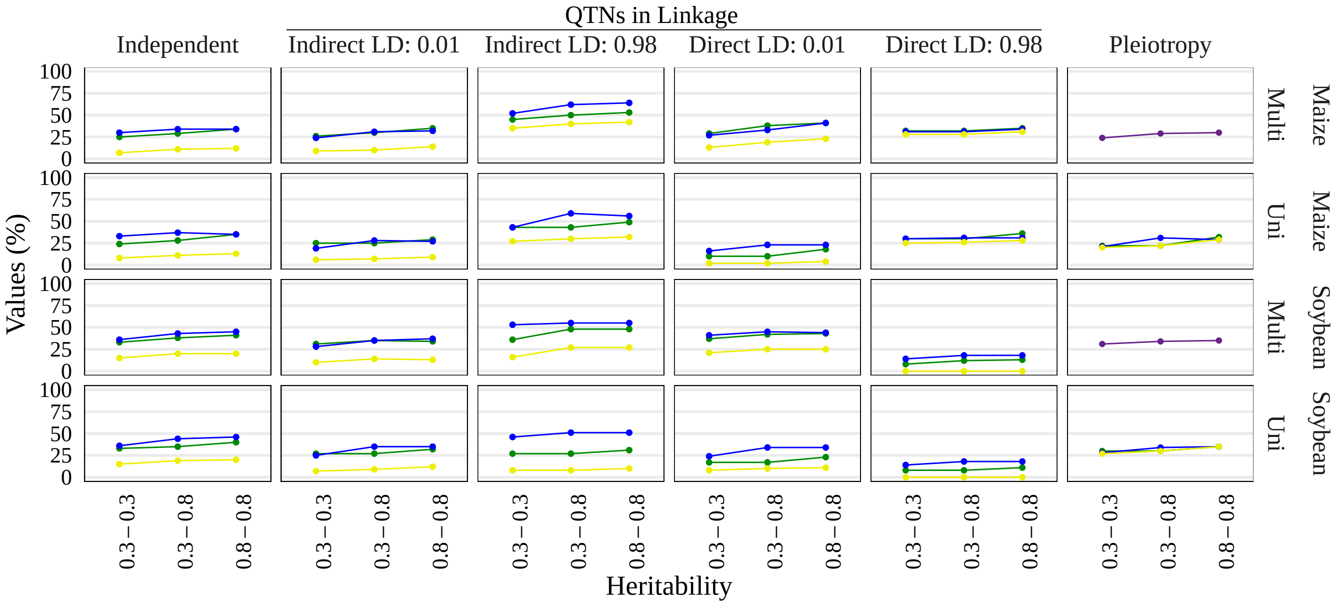

B

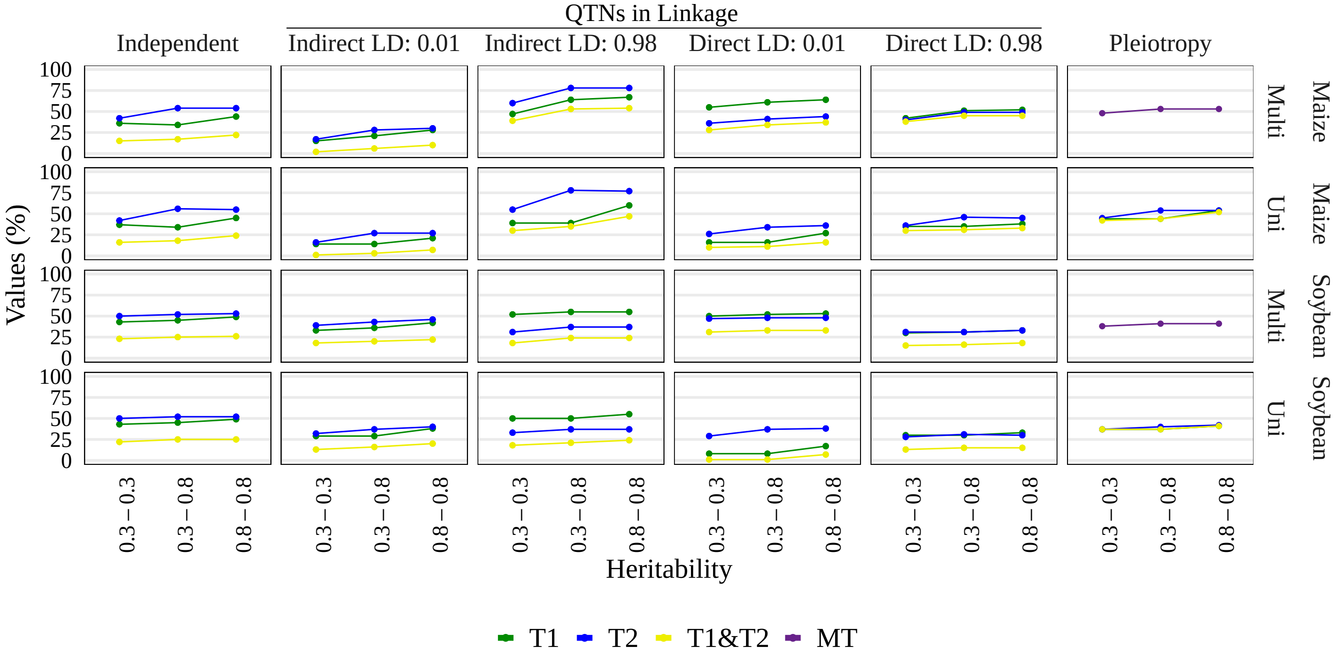

Figure S41: Quantitative trait nucleotide (QTN) and spurious pleiotropy detection rate (Y-axis) achieved by multivariate (Multi) and univariate (Uni) GWAS, relative to the QTN controlling trait 1 (T1), trait 2 (T2), and both QTN simultaneously (T1&T2) or, in the pleiotropic scenario, relative to the pleiotropic QTN (MT). These values were obtained for maize and soybean with a sample size of 1,000. The X-axis displays the narrow-sense heritability for Trait 1 (bottom value) and Trait 2 (top value). A) inputted minor allele frequency (MAF) of 0.05; B) MAF of 0.4. The false discovery rate rate was 0.05 and the window size was 1 kb for maize and 10 Kb for soybean.

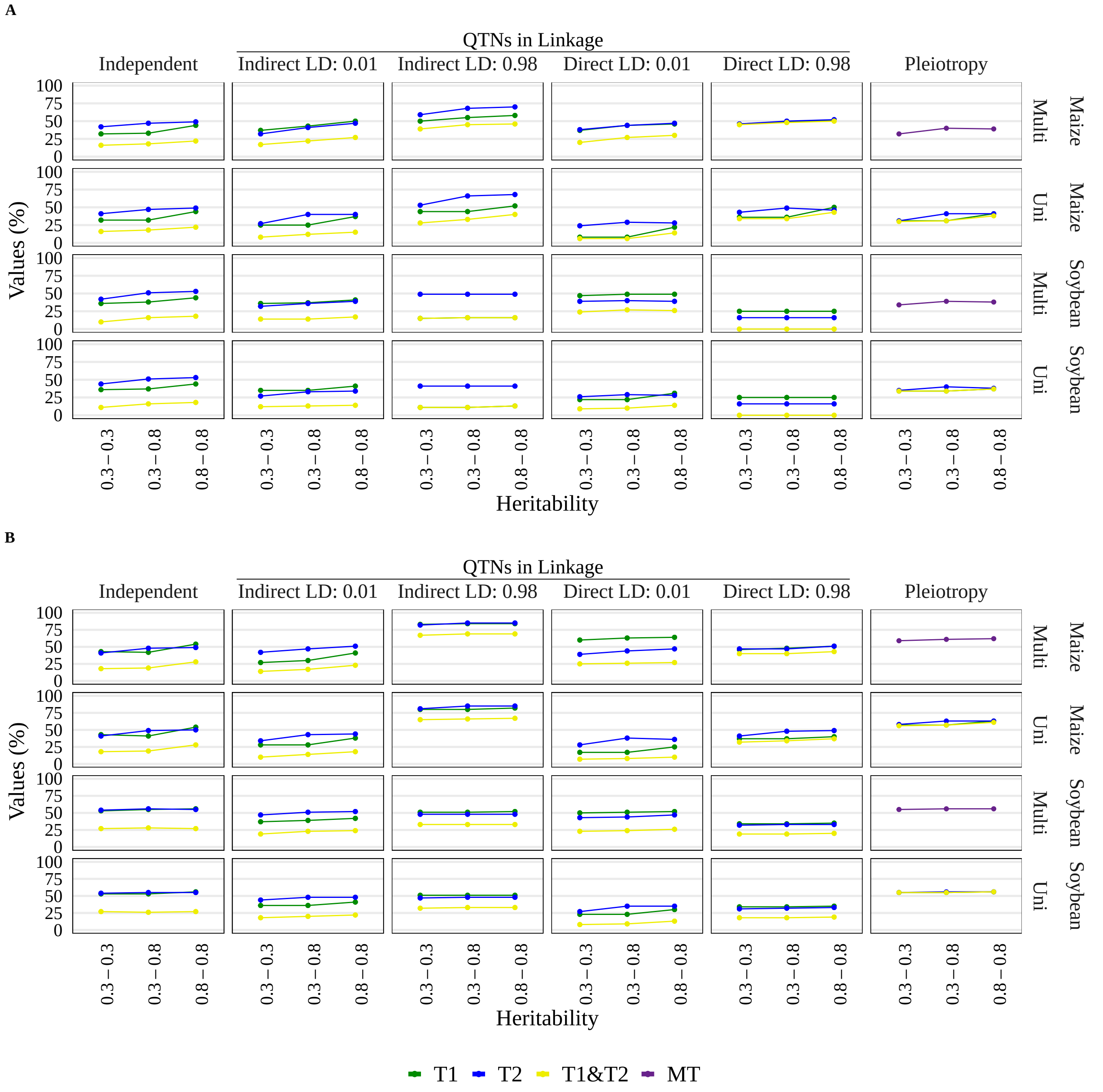

Figure S42: Quantitative trait nucleotide (QTN) and spurious pleiotropy detection rate (Y-axis) achieved by multivariate (Multi) and univariate (Uni) GWAS, relative to the QTN controlling trait 1 (T1), trait 2 (T2), and both QTN simultaneously (T1&T2) or, in the pleiotropic scenario, relative to the pleiotropic QTN (MT). These values were obtained for maize and soybean with a sample size of 2, 815. The X-axis displays the narrow-sense heritability for Trait 1 (bottom value) and Trait 2 (top value). A) inputted minor allele frequency (MAF) of 0.05; B) MAF of 0.4. The false discovery rate rate was 0.05 and the window size was 1 kb for maize and 10 Kb for soybean.

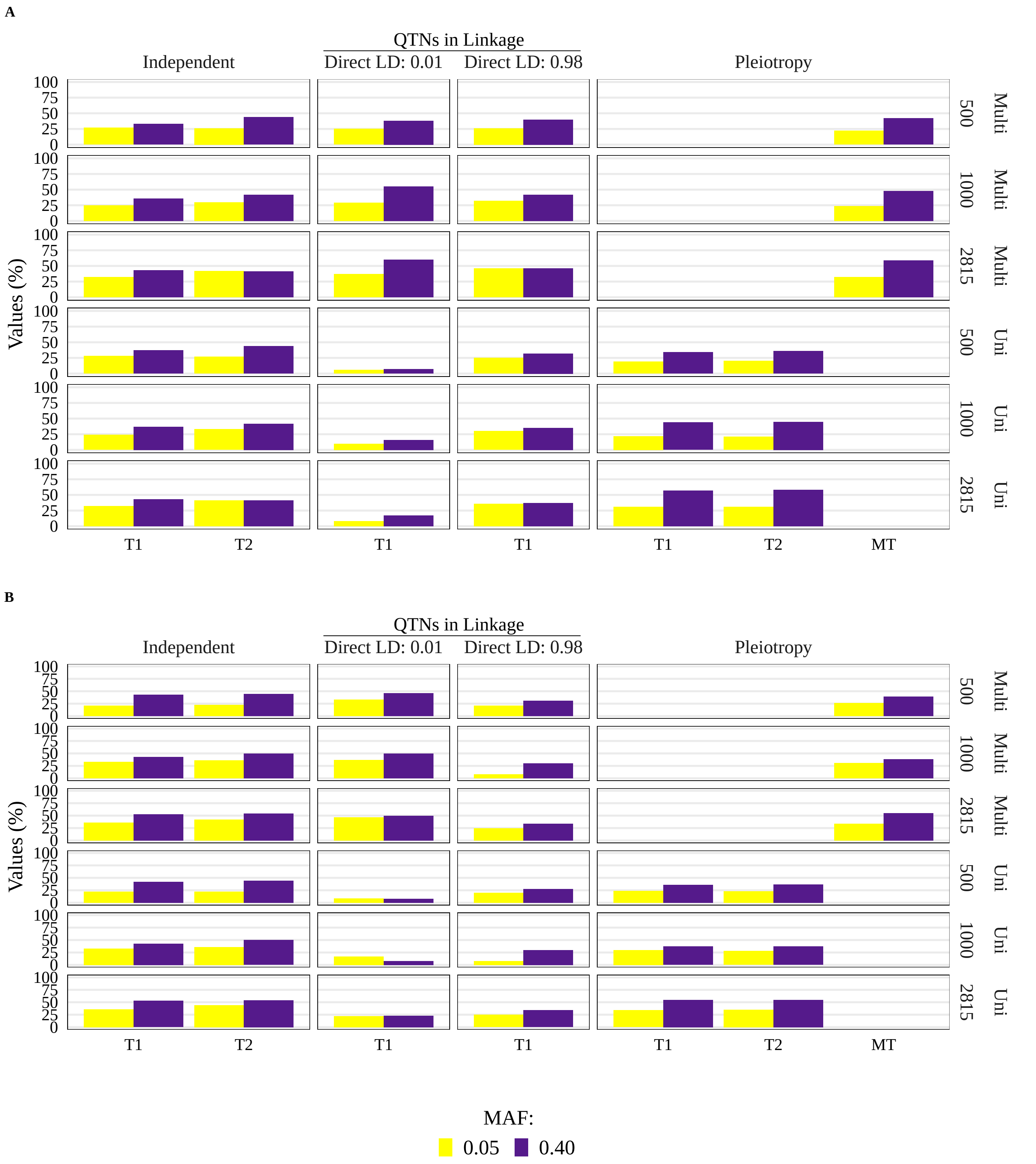

Figure S43: Quantitative trait nucleotide (QTN) and spurious pleiotropy detection rate (Y-axis) in scenarios for which minor allele frequency (MAF) was directly controlled by a simulation input parameter. These values were obtained by multivariate (Multi) and univariate (Uni) GWAS, relative to the QTN controlling trait 1 (T1), trait 2 (T2) or, in the pleiotropic scenario, relative to the pleiotropic QTN (MT). This figure shows results for a narrow-sense heritability of 0.3 for both traits. A) Maize; B) Soybean. The false discovery rate rate was 0.05 and the window size was 1 kb for maize and 10 Kb for soybean.

A

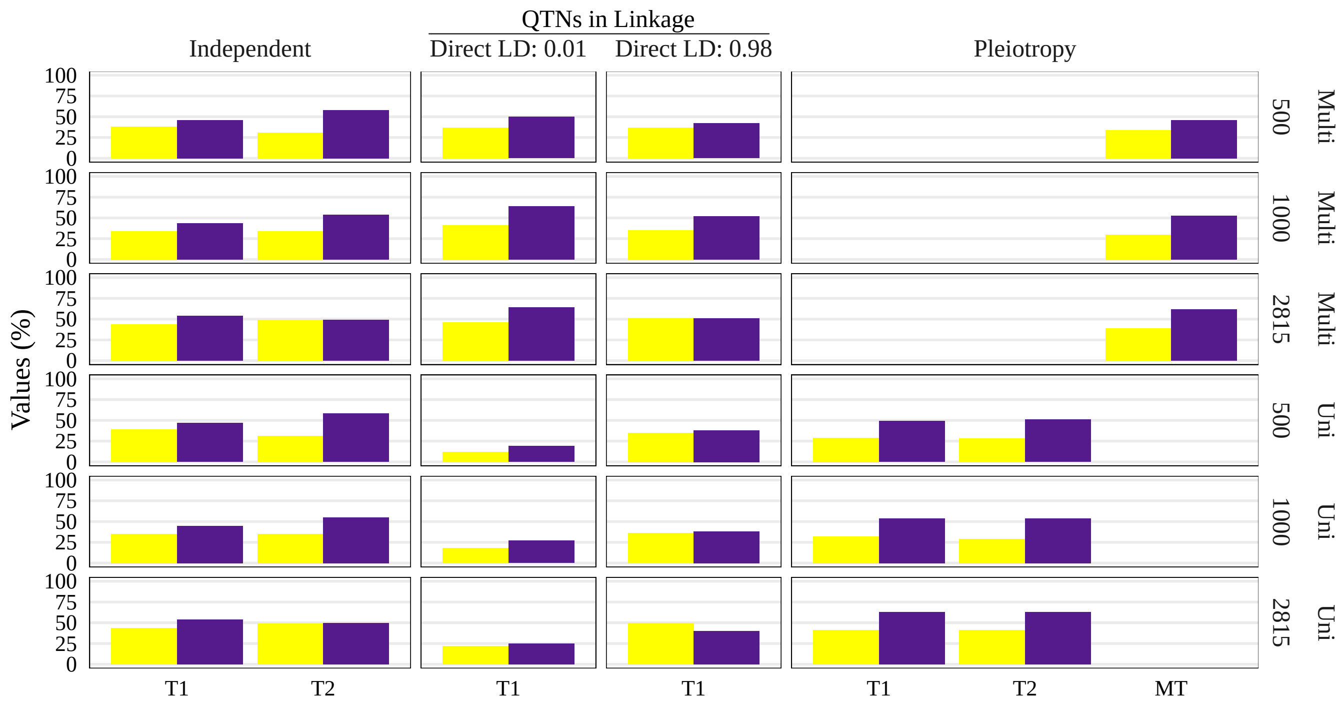

B

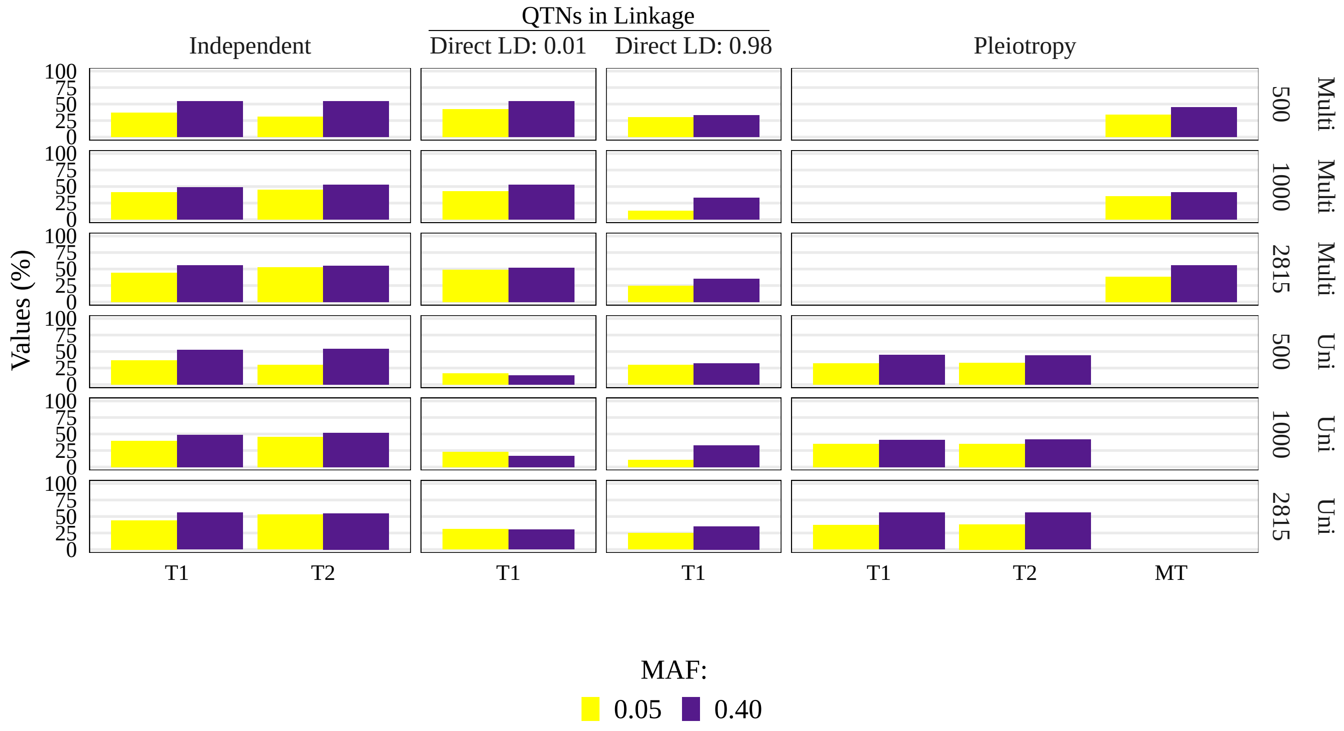

Figure S44: Quantitative trait nucleotide (QTN) and spurious pleiotropy detection rate (Y-axis) in scenarios for which minor allele frequency (MAF) was directly controlled by a simulation input parameter. These values were obtained by multivariate (Multi) and univariate (Uni) GWAS, relative to the QTN controlling trait 1 (T1), trait 2 (T2) or, in the pleiotropic scenario, relative to the pleiotropic QTN (MT). This figure shows results for a narrow-sense heritability of 0.8 for both traits. A) Maize; B) Soybean. The false discovery rate rate was 0.05 and the window size was 1 kb for maize and 10 Kb for soybean.

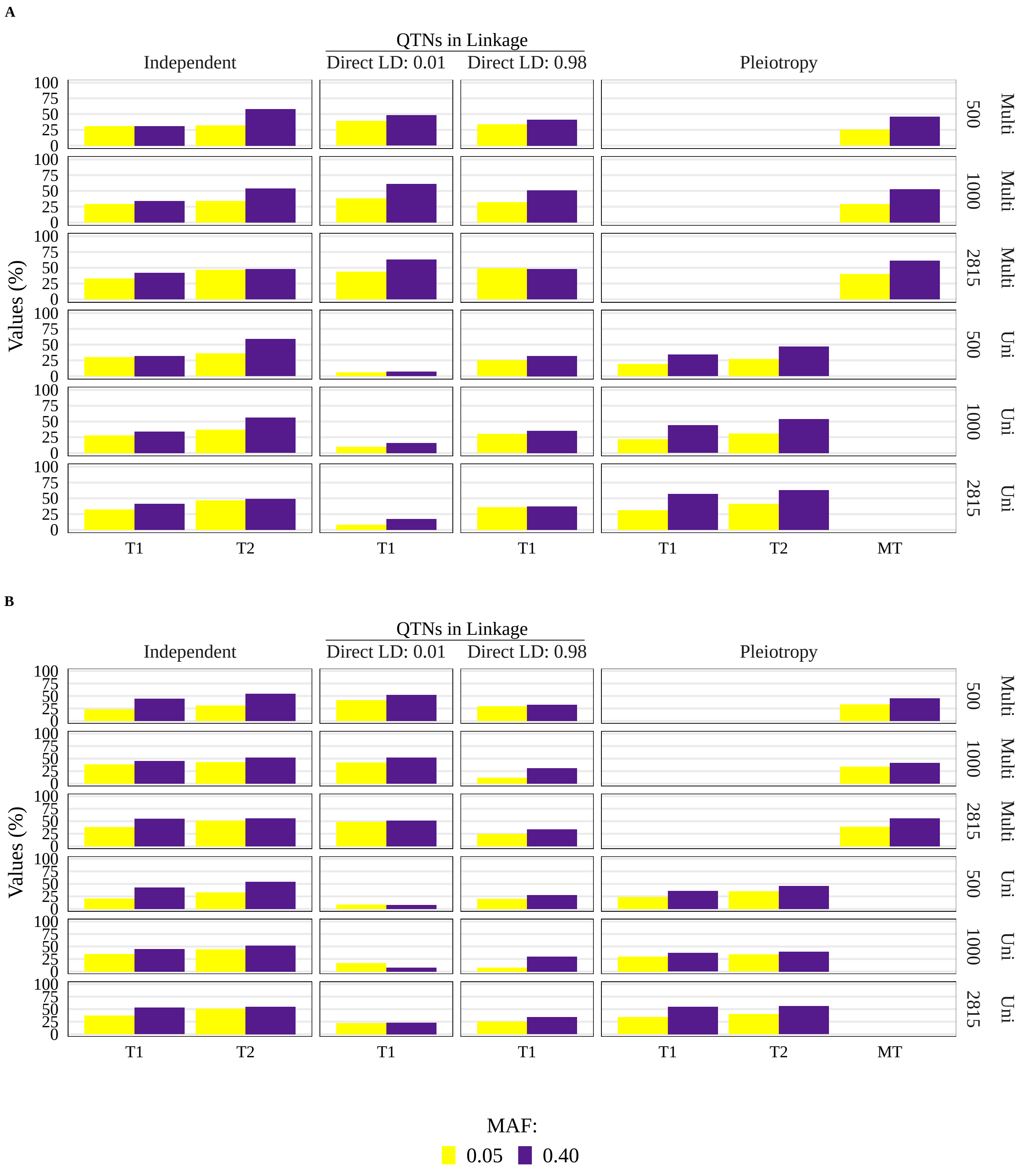

Figure S45: Quantitative trait nucleotide (QTN) and spurious pleiotropy detection rate (Y-axis) in scenarios for which minor allele frequency (MAF) was directly controlled by a simulation input parameter. These values were obtained by multivariate (Multi) and univariate (Uni) GWAS, relative to the QTN controlling trait 1 (T1), trait 2 (T2) or, in the pleiotropic scenario, relative to the pleiotropic QTN (MT). This figure shows results for a narrow-sense heritability of 0.3 for trait 1 and 0.8 for trait 2. A) Maize; B) Soybean. The false discovery rate rate was 0.05 and the window size was 1 kb for maize and 10 Kb for soybean.

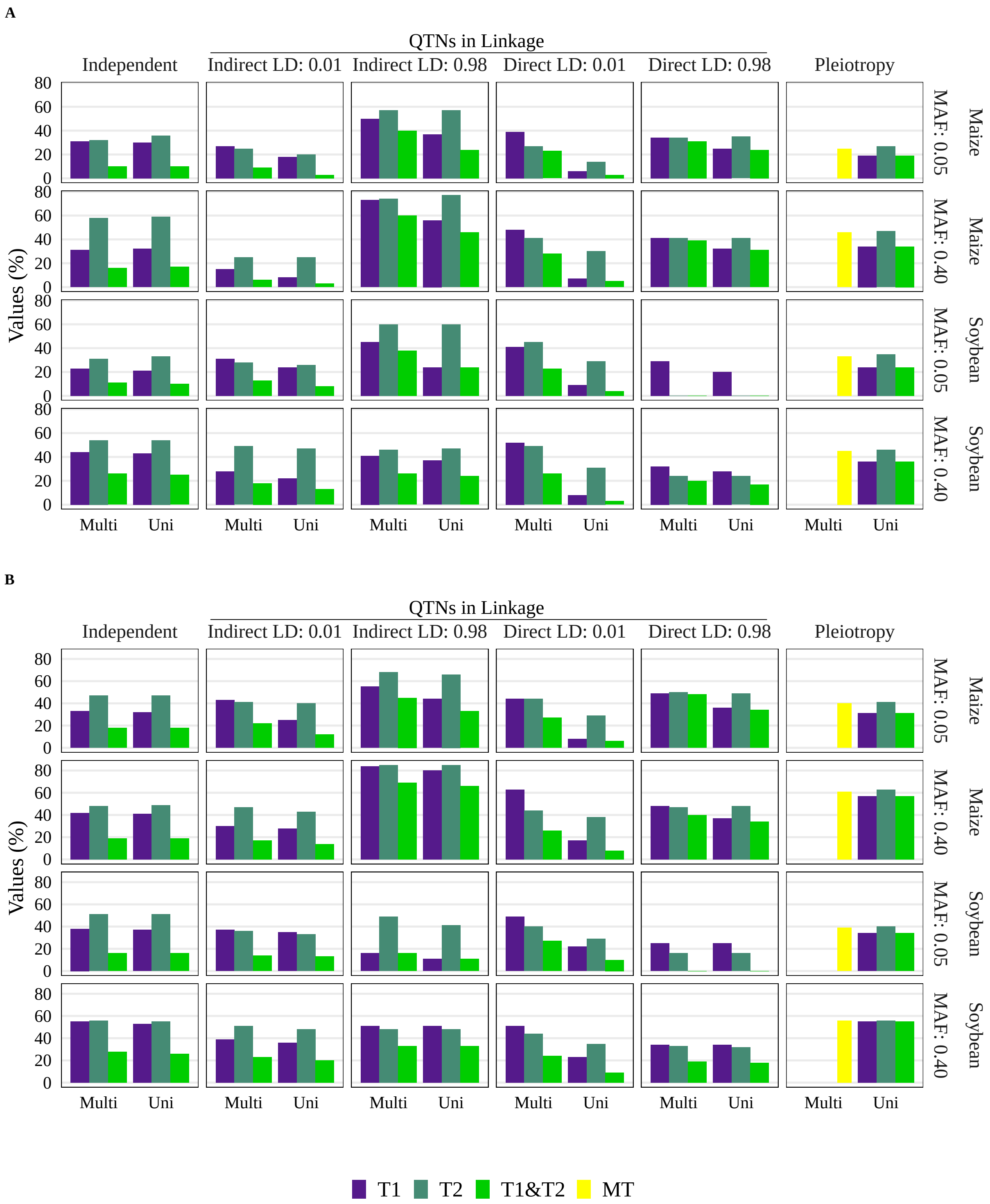

Figure S46: Quantitative trait nucleotide (QTN) and spurious pleiotropy detection rate (Y-axis) achieved by multivariate (Multi) and univariate (Uni) GWAS (X-axis), relative to the QTN controlling trait 1 (T1), trait 2 (T2), and both QTN simultaneously (T1&T2) or, in the pleiotropic scenario, relative to the pleiotropic QTN (MT). The simulated genetic architecture is listed in the horizontal and vertical titles. These values were obtained with a narrow-sense heritability of 0.3 and 0.8 for traits 1 and 2, respectively. A) Sample size of 500; B) Sample size of 2815. MAF: minor allele frequencies. The false discovery rate rate was 0.05 and the window size was 1 kb for maize and 10 Kb for soybean.

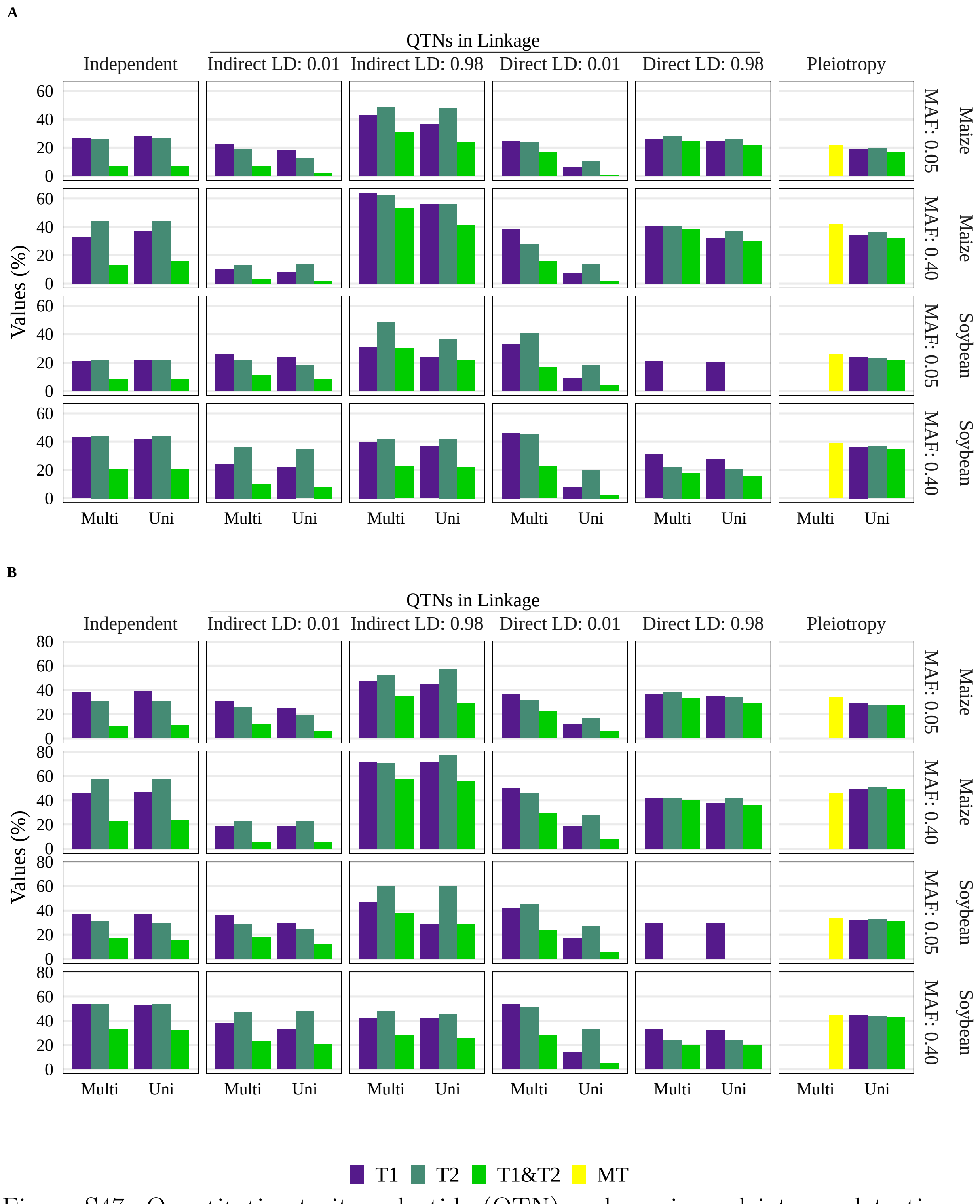

Figure S47: Quantitative trait nucleotide (QTN) and spurious pleiotropy detection rate (Y-axis) achieved by multivariate (Multi) and univariate (Uni) GWAS (X-axis), relative to the QTN controlling trait 1 (T1), trait 2 (T2), and both QTN simultaneously (T1&T2) or, in the pleiotropic scenario, relative to the pleiotropic QTN (MT). The simulated genetic architecture is listed in the horizontal and vertical titles. These values were obtained with a sample size of 500; A) a narrow-sense heritability of 0.3 for both traits; B) a narrow-sense heritability of 0.8 for both traits. MAF: minor allele frequencies. The false discovery rate rate was 0.05 and the window size was 1 kb for maize and 10 Kb for soybean.

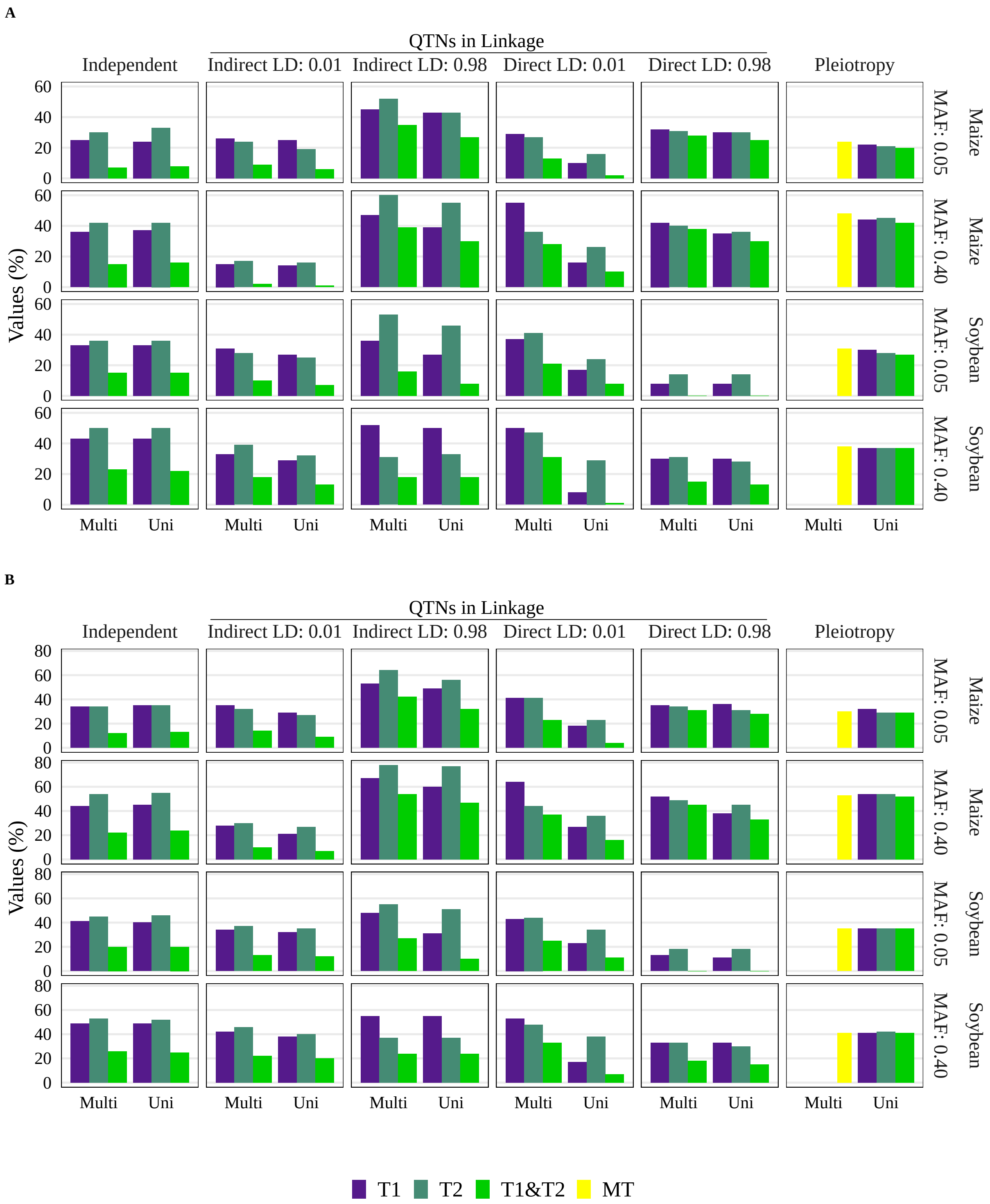

Figure S48: Quantitative trait nucleotide (QTN) and spurious pleiotropy detection rate (Y-axis) achieved by multivariate (Multi) and univariate (Uni) GWAS (X-axis), relative to the QTN controlling trait 1 (T1), trait 2 (T2), and both QTN simultaneously (T1&T2) or, in the pleiotropic scenario, relative to the pleiotropic QTN (MT). The simulated genetic architecture is listed in the horizontal and vertical titles. These values were obtained with a sample size of 1, 000; A) a narrow-sense heritability of 0.3 for both traits; B) a narrow-sense heritability of 0.8 for both traits. MAF: minor allele frequencies. The false discovery rate rate was 0.05 and the window size was 1 kb for maize and 10 Kb for soybean.

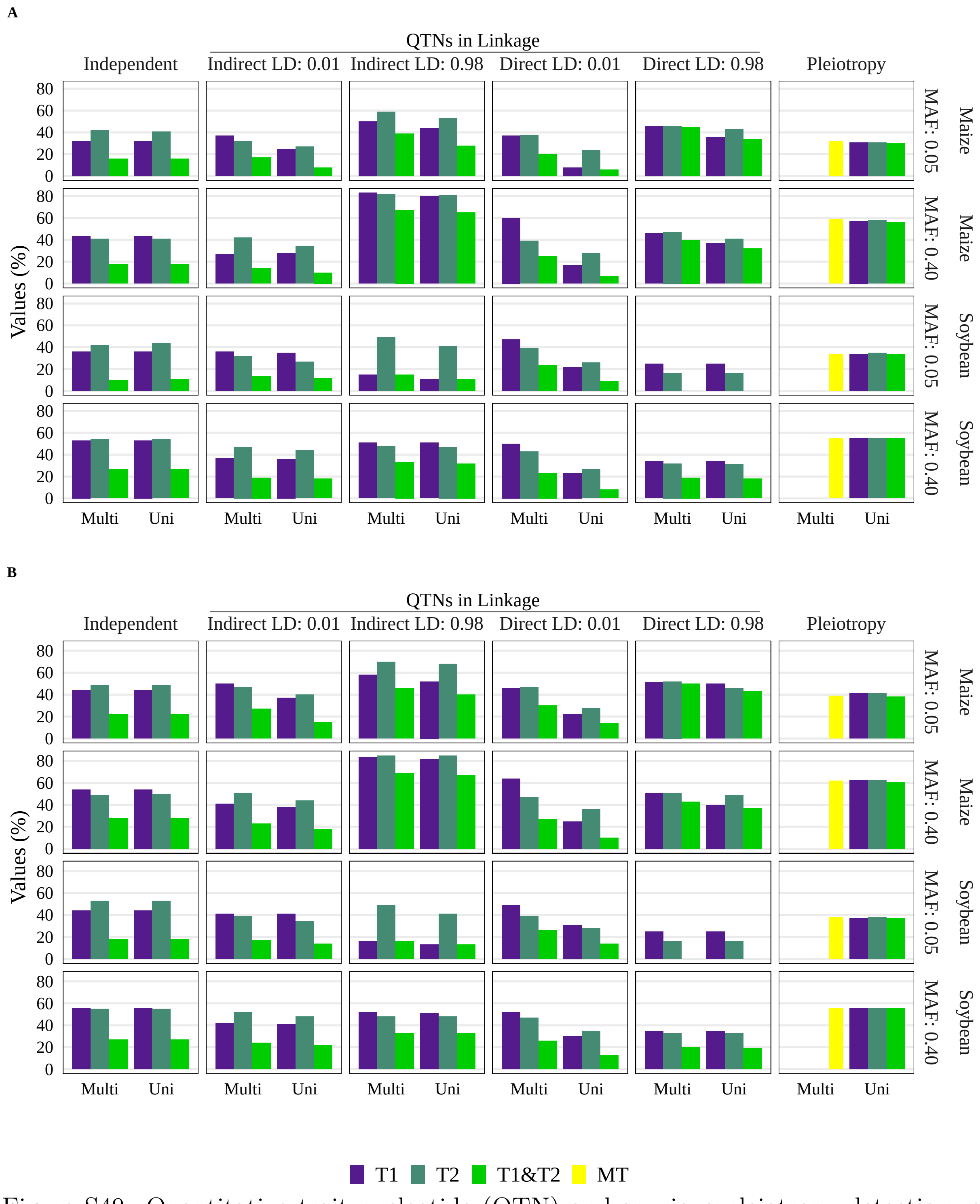

Figure S49: Quantitative trait nucleotide (QTN) and spurious pleiotropy detection rate (Y-axis) achieved by multivariate (Multi) and univariate (Uni) GWAS (X-axis), relative to the QTN controlling trait 1 (T1), trait 2 (T2), and both QTN simultaneously (T1&T2) or, in the pleiotropic scenario, relative to the pleiotropic QTN (MT). The simulated genetic architecture is listed in the horizontal and vertical titles. These values were obtained with a sample size of 2, 815; A) a narrow-sense heritability of 0.3 for both traits; B) a narrow-sense heritability of 0.8 for both traits. MAF: minor allele frequencies. The false discovery rate rate was 0.05 and the window size was 1 kb for maize and 10 Kb for soybean.

Error rate with an FDR of 0.1 and window size of 10 Kb for maize and 1 Mb for soybean

A

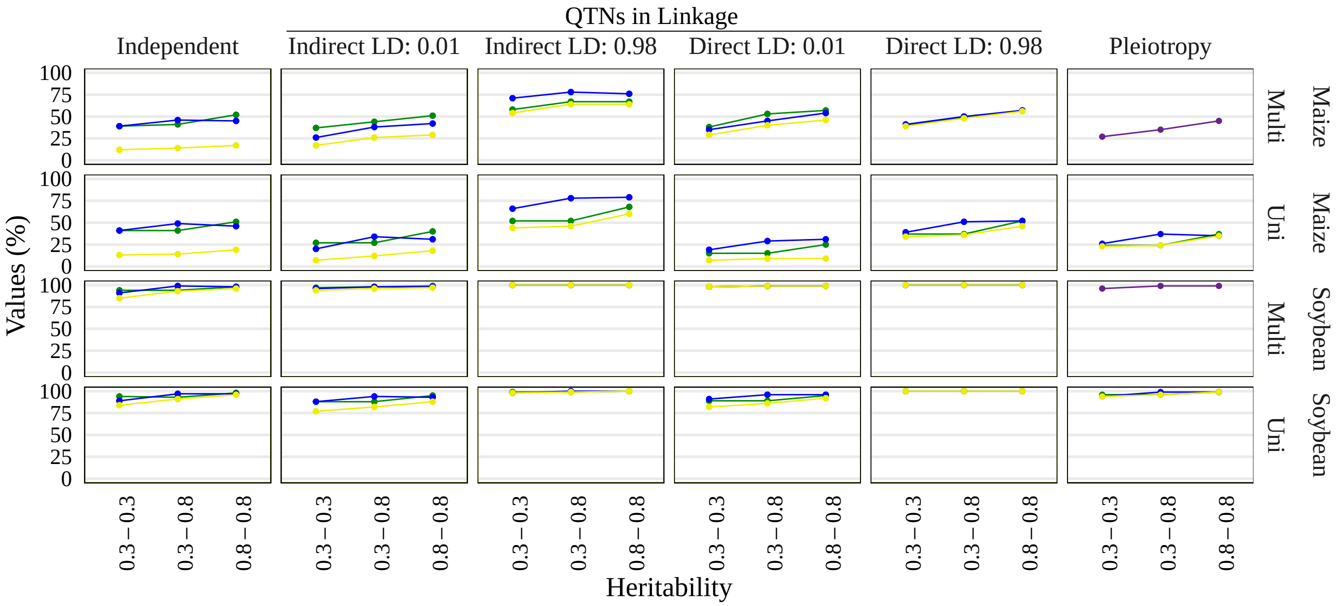

B

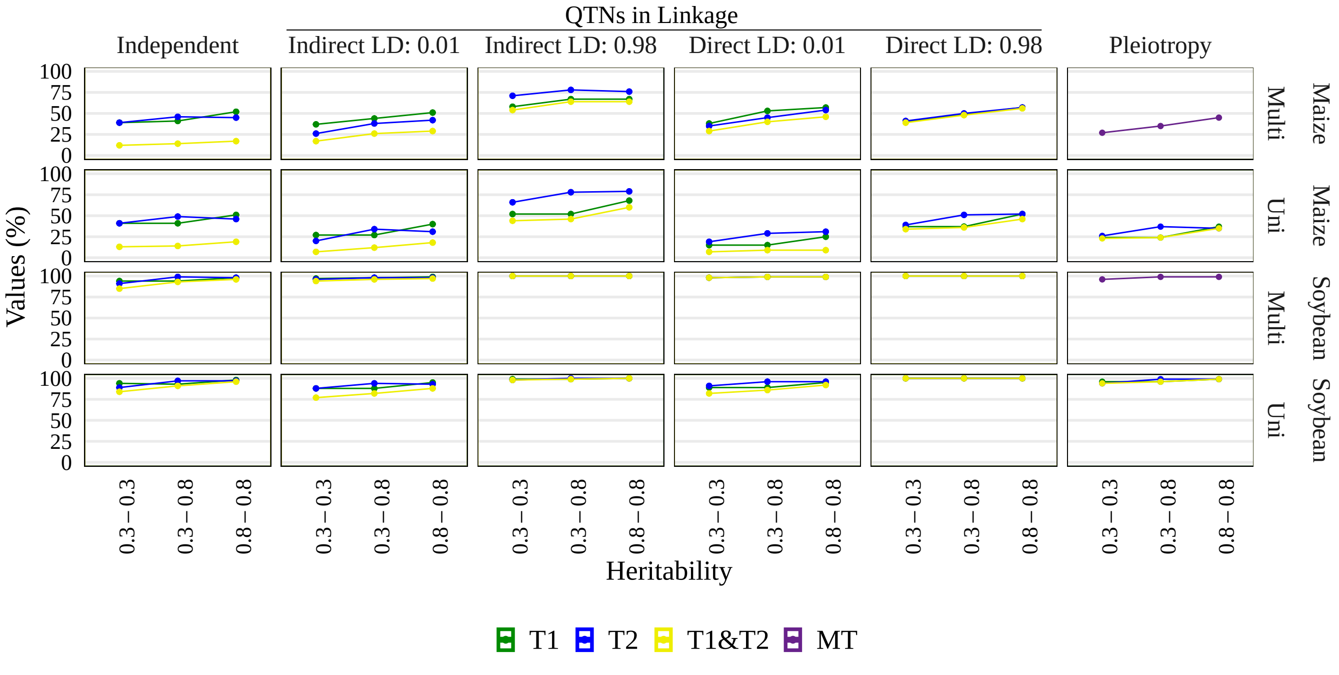

Figure S50: Error rate (measured as the detection of significant SNPs outside the window size) (Y-axis) achieved by multivariate (Multi) and univariate (Uni) GWAS, relative to the QTN controlling trait 1 (T1), trait 2 (T2), and both QTN simultaneously (T1&T2) or, in the pleiotropic scenario, relative to the pleiotropic QTN (MT). These values were obtained for maize and soybean with a sample size of 500. The X-axis displays the narrow-sense heritability for Trait 1 (bottom value) and Trait 2 (top value). A) inputted minor allele frequency (MAF) of 0.05; B) MAF of 0.4. The false discovery rate rate was 0.1 and the window size was 10 kb for maize and 1 Mb for soybean.

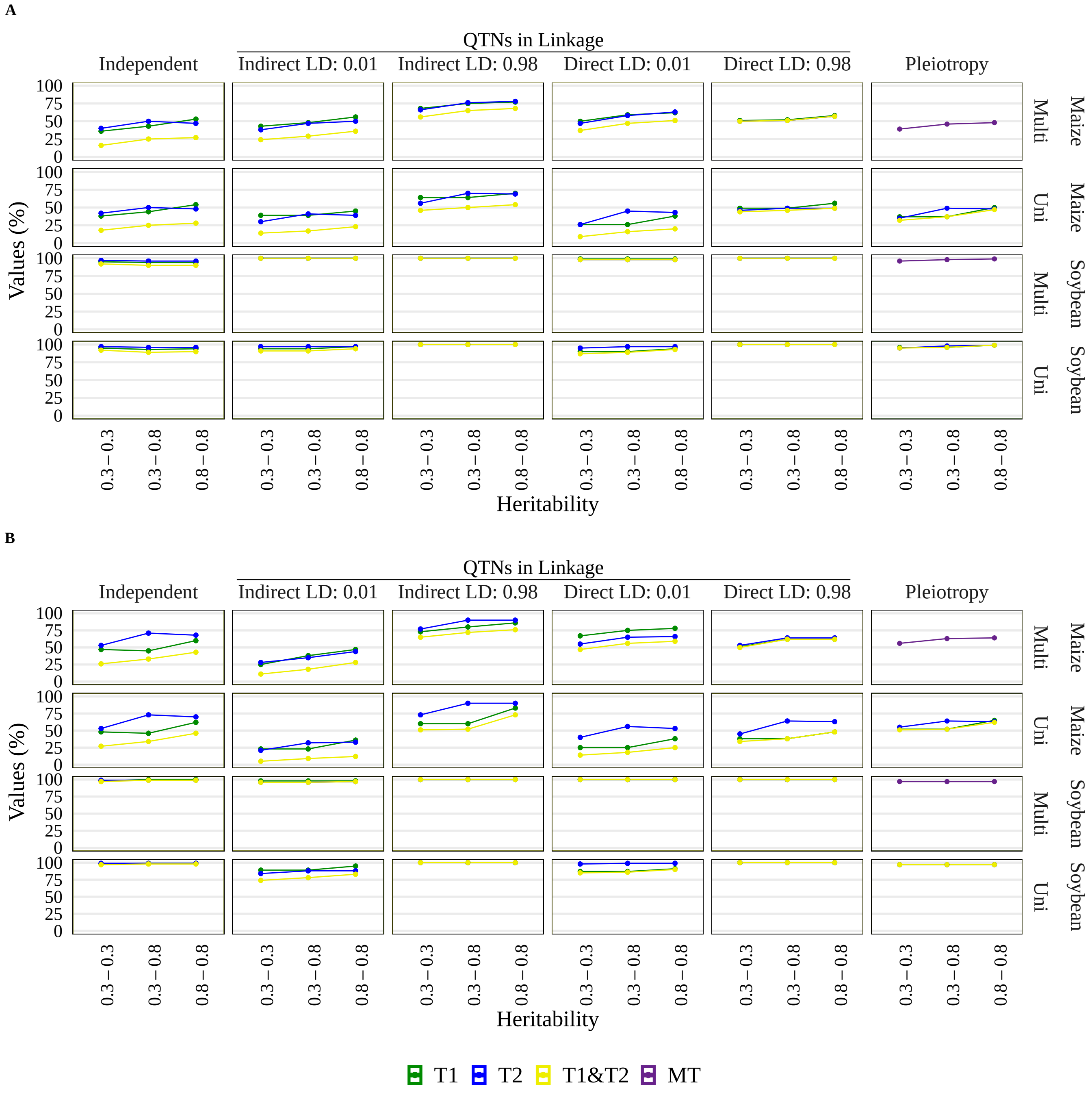

Figure S51: Error rate (measured as the detection of significant SNPs outside the window size) (Y-axis) achieved by multivariate (Multi) and univariate (Uni) GWAS, relative to the QTN controlling trait 1 (T1), trait 2 (T2), and both QTN simultaneously (T1&T2) or, in the pleiotropic scenario, relative to the pleiotropic QTN (MT). These values were obtained for maize and soybean with a sample size of 1,000. The X-axis displays the narrow-sense heritability for Trait 1 (bottom value) and Trait 2 (top value). A) inputted minor allele frequency (MAF) of 0.05; B) MAF of 0.4. The false discovery rate rate was 0.1 and the window size was 10 kb for maize and 1 Mb for soybean.

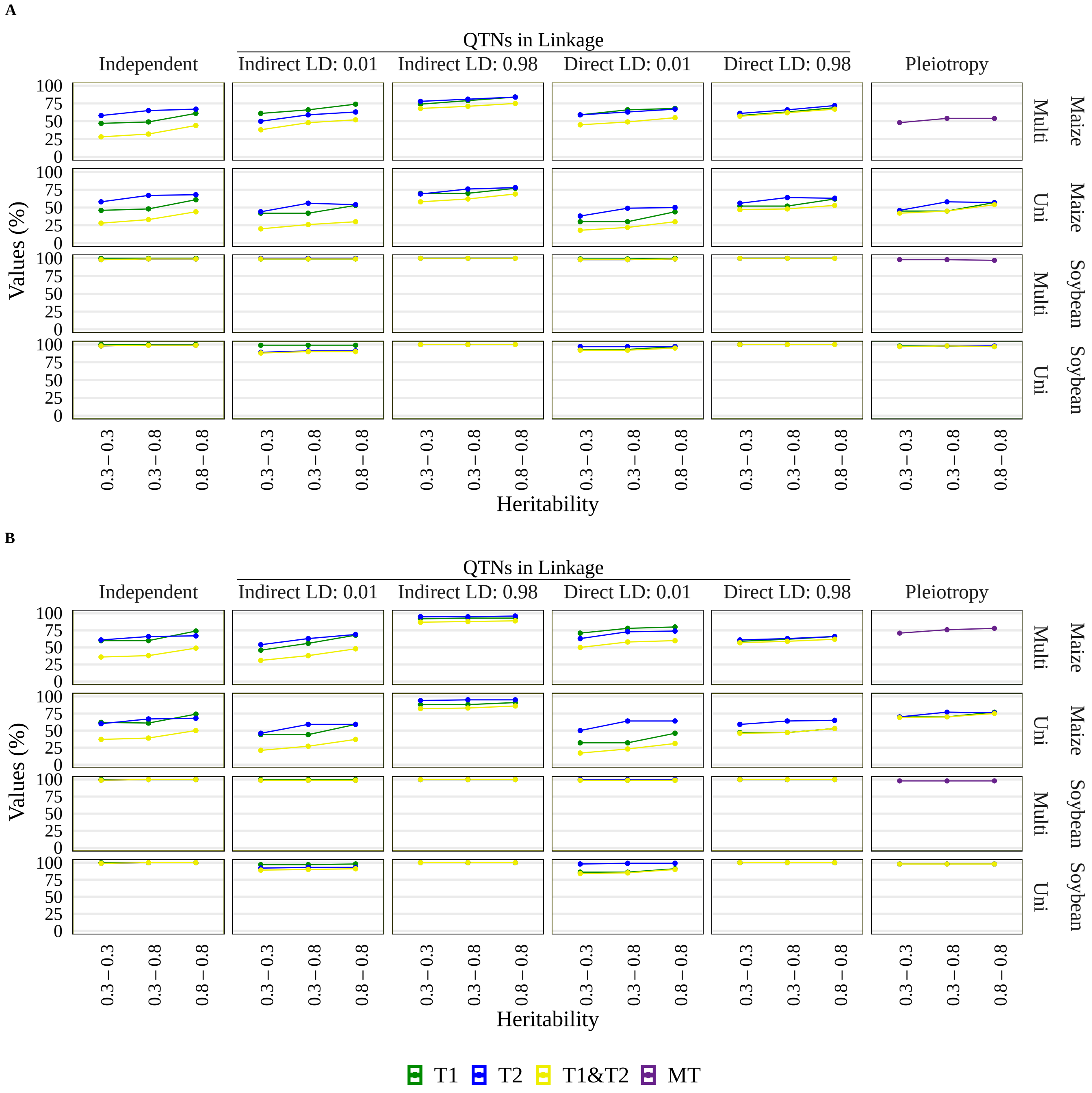

Figure S52: Error rate (measured as the detection of significant SNPs outside the window size) (Y-axis) achieved by multivariate (Multi) and univariate (Uni) GWAS, relative to the QTN controlling trait 1 (T1), trait 2 (T2), and both QTN simultaneously (T1&T2) or, in the pleiotropic scenario, relative to the pleiotropic QTN (MT). These values were obtained for maize and soybean with a sample size of 2,815. The X-axis displays the narrow-sense heritability for Trait 1 (bottom value) and Trait 2 (top value). A) inputted minor allele frequency (MAF) of 0.05; B) MAF of 0.4. The false discovery rate rate was 0.1 and the window size was 10 kb for maize and 1 Mb for soybean.

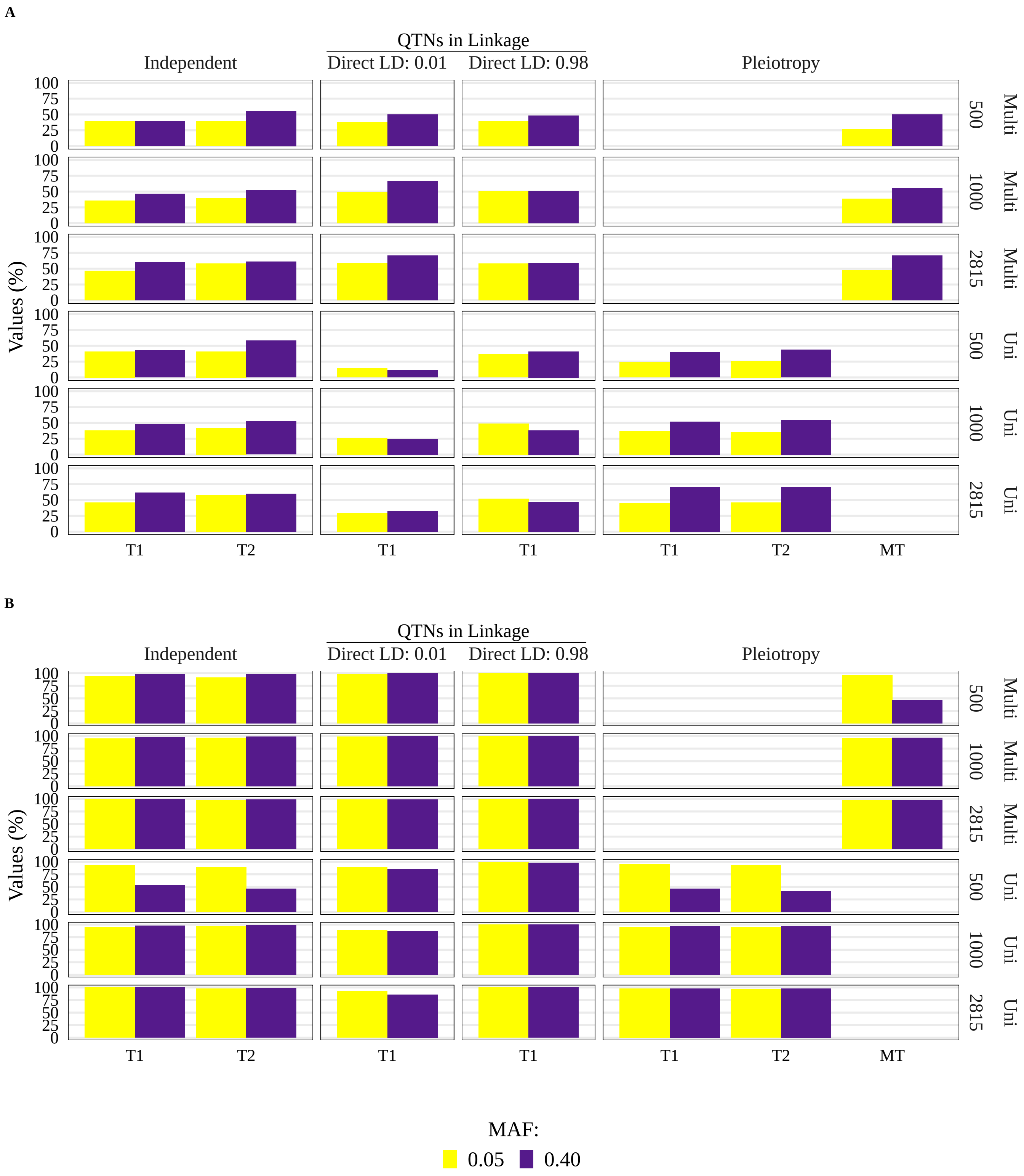

Figure S53: Error rate (measured as the detection of significant SNPs outside the window size) (Y-axis) in scenarios for which minor allele frequency (MAF) was directly controlled by a simulation input parameter. These values were obtained by multivariate (Multi) and univariate (Uni) GWAS, relative to the QTN controlling trait 1 (T1), trait 2 (T2) or, in the pleiotropic scenario, relative to the pleiotropic QTN (MT). This figure shows results for a narrow-sense heritability of 0.3 for both traits. A) Maize; B) Soybean. The false discovery rate rate was 0.1 and the window size was 10 kb for maize and 1 Mb for soybean.

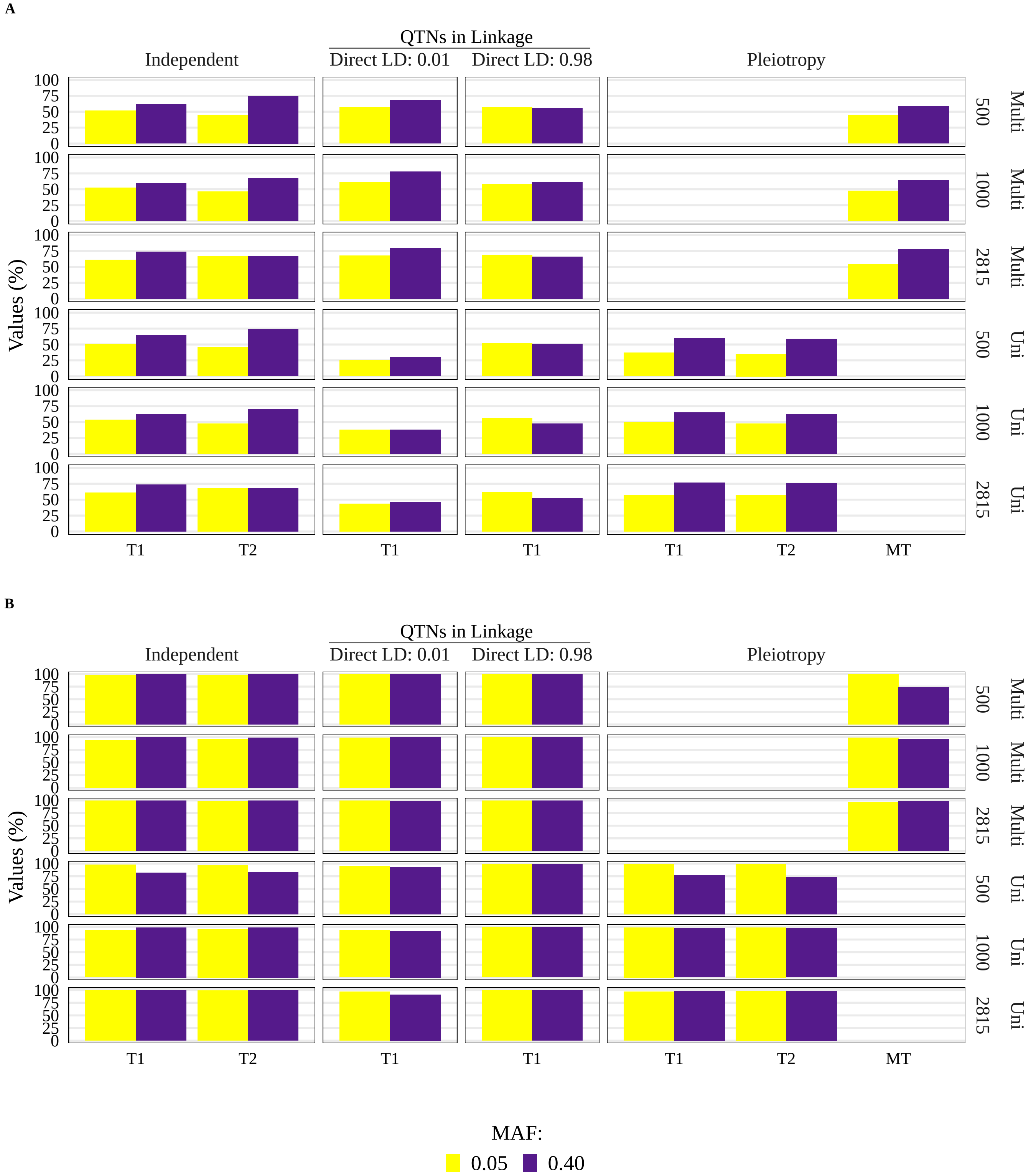

Figure S54: Error rate (measured as the detection of significant SNPs outside the window size) (Y-axis) in scenarios for which minor allele frequency (MAF) was directly controlled by a simulation input parameter. These values were obtained by multivariate (Multi) and univariate (Uni) GWAS, relative to the QTN controlling trait 1 (T1), trait 2 (T2) or, in the pleiotropic scenario, relative to the pleiotropic QTN (MT). This figure shows results for a narrow-sense heritability of 0.8 for both traits. A) Maize; B) Soybean. The false discovery rate rate was 0.1 and the window size was 10 kb for maize and 1 Mb for soybean.

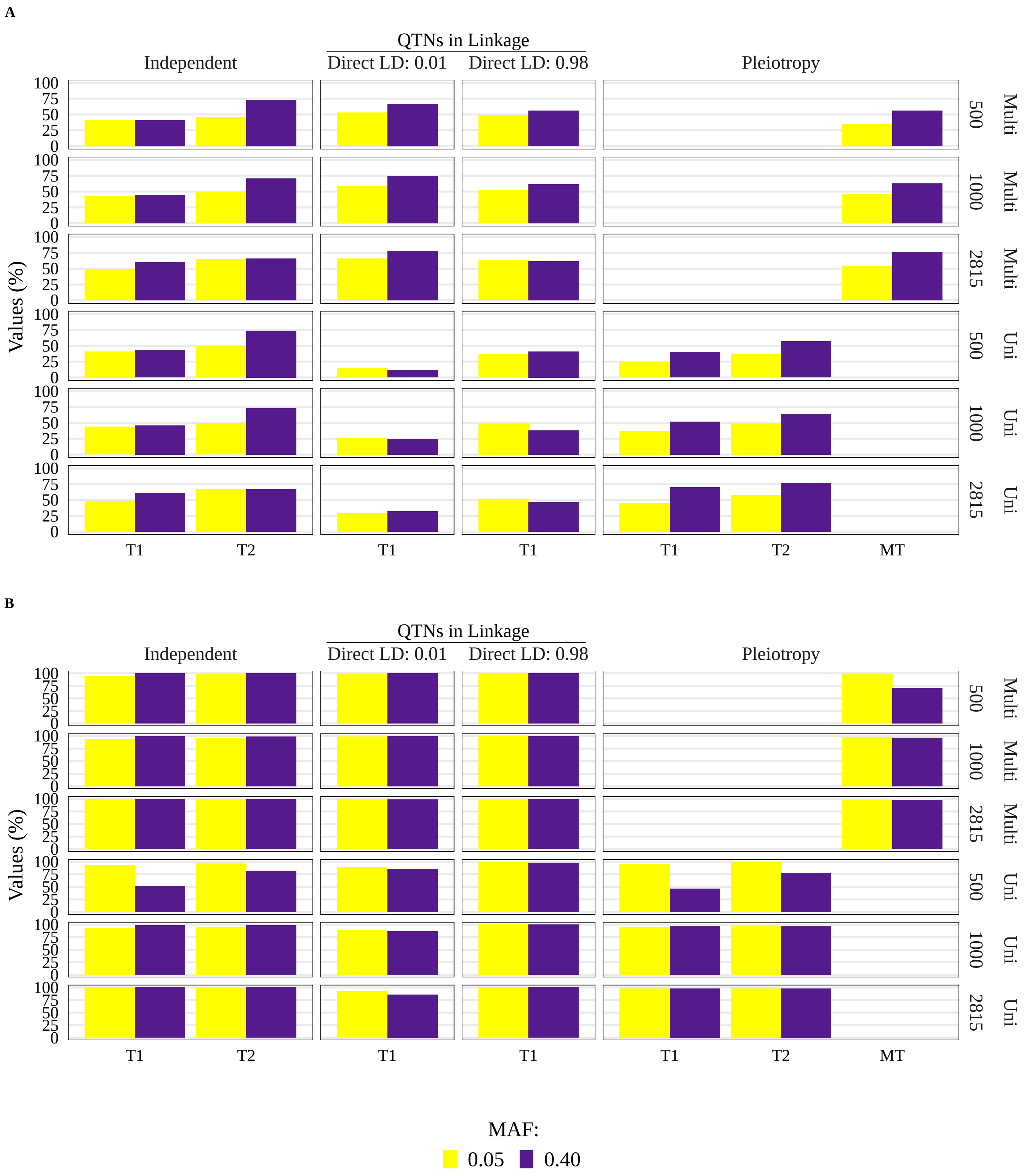

Figure S55: Error rate (measured as the detection of significant SNPs outside the window size) (Y-axis) in scenarios for which minor allele frequency (MAF) was directly controlled by a simulation input parameter. These values were obtained by multivariate (Multi) and univariate (Uni) GWAS, relative to the QTN controlling trait 1 (T1), trait 2 (T2) or, in the pleiotropic scenario, relative to the pleiotropic QTN (MT). This figure shows results for a narrow-sense heritability of 0.3 for trait 1 and 0.8 for trait 2. A) Maize; B) Soybean. The false discovery rate rate was 0.1 and the window size was 10 kb for maize and 1 Mb for soybean.

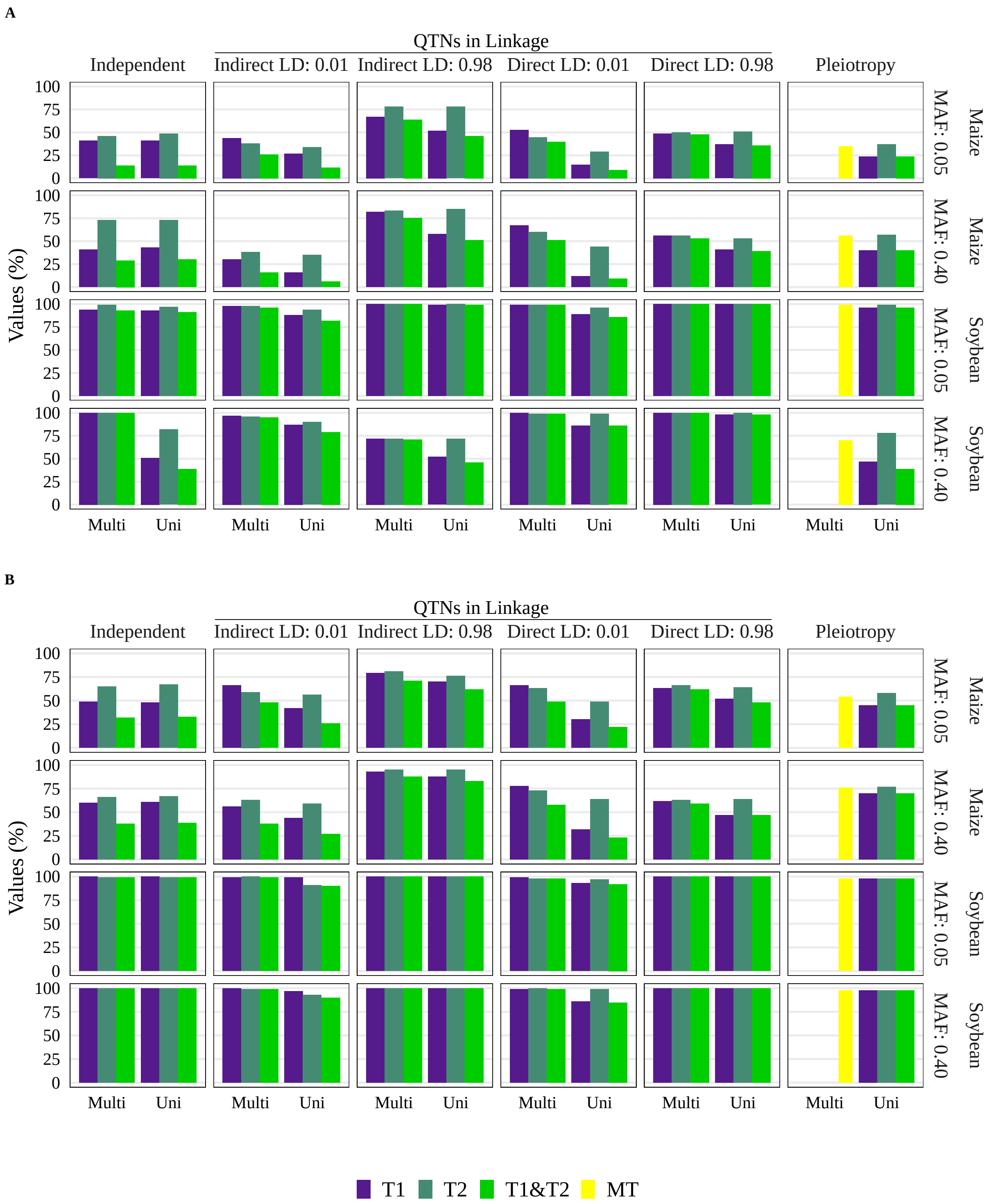

Figure S56: Error rate (measured as the detection of significant SNPs outside the window size) (Y-axis) achieved by multivariate (Multi) and univariate (Uni) GWAS (X-axis), relative to the QTN controlling trait 1 (T1), trait 2 (T2), and both QTN simultaneously (T1&T2) or, in the pleiotropic scenario, relative to the pleiotropic QTN (MT). The simulated genetic architecture is listed in the horizontal and vertical titles. These values were obtained with a narrow-sense heritability of 0.3 and 0.8 for traits 1 and 2, respectively. A) Sample size of 500; B) Sample size of 2815. MAF: minor allele frequencies. The false discovery rate rate was 0.1 and the window size was 10 kb for maize and 1 Mb for soybean.

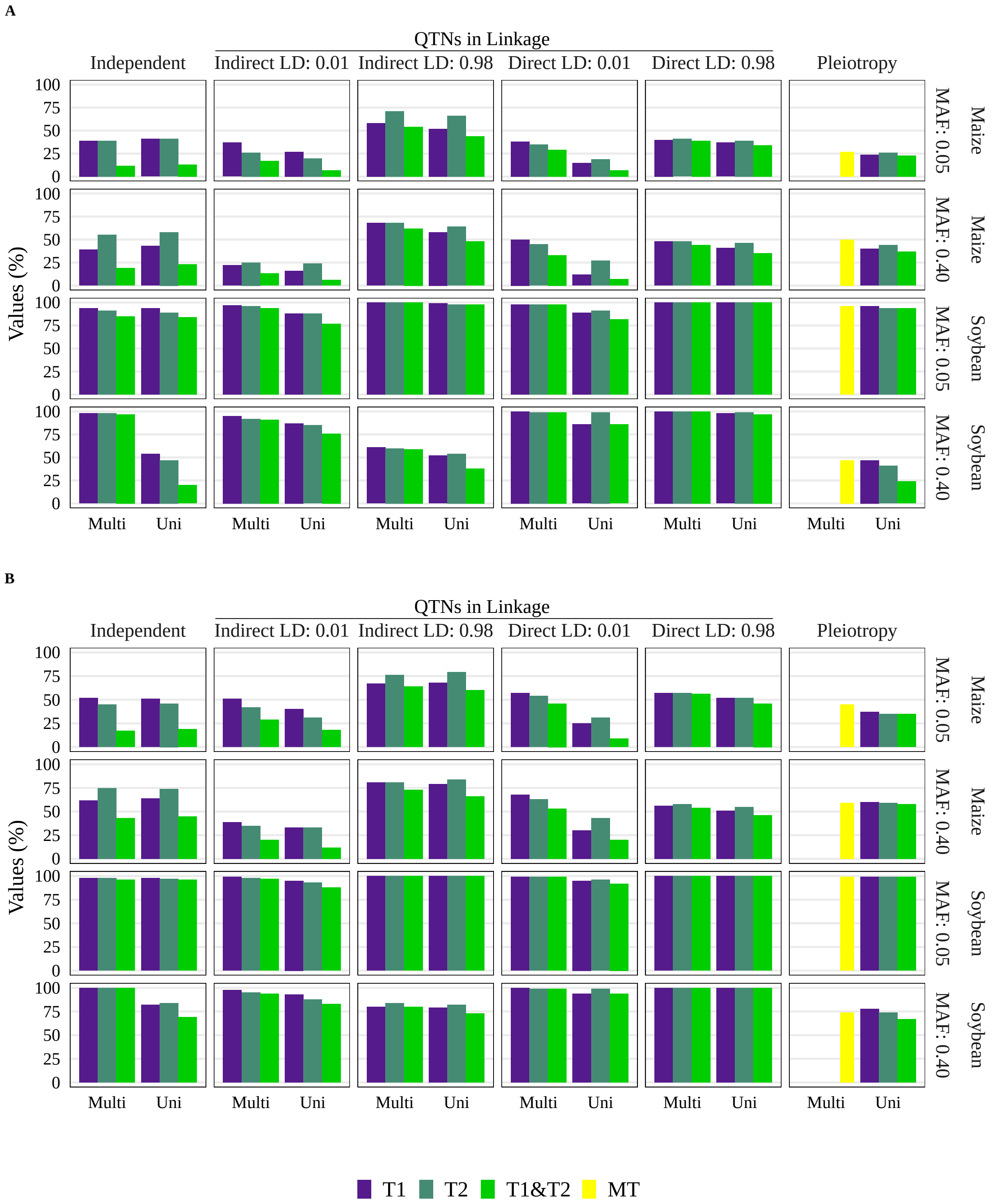

Figure S57: Error rate (measured as the detection of significant SNPs outside the window size) (Y-axis) achieved by multivariate (Multi) and univariate (Uni) GWAS (X-axis), relative to the QTN controlling trait 1 (T1), trait 2 (T2), and both QTN simultaneously (T1&T2) or, in the pleiotropic scenario, relative to the pleiotropic QTN (MT). The simulated genetic architecture is listed in the horizontal and vertical titles. These values were obtained with a sample size of 500; A) a narrow-sense heritability of 0.3 for both traits; B) a narrow-sense heritability of 0.8 for both traits. MAF: minor allele frequencies. The false discovery rate rate was 0.1 and the window size was 10 kb for maize and 1 Mb for soybean.

A

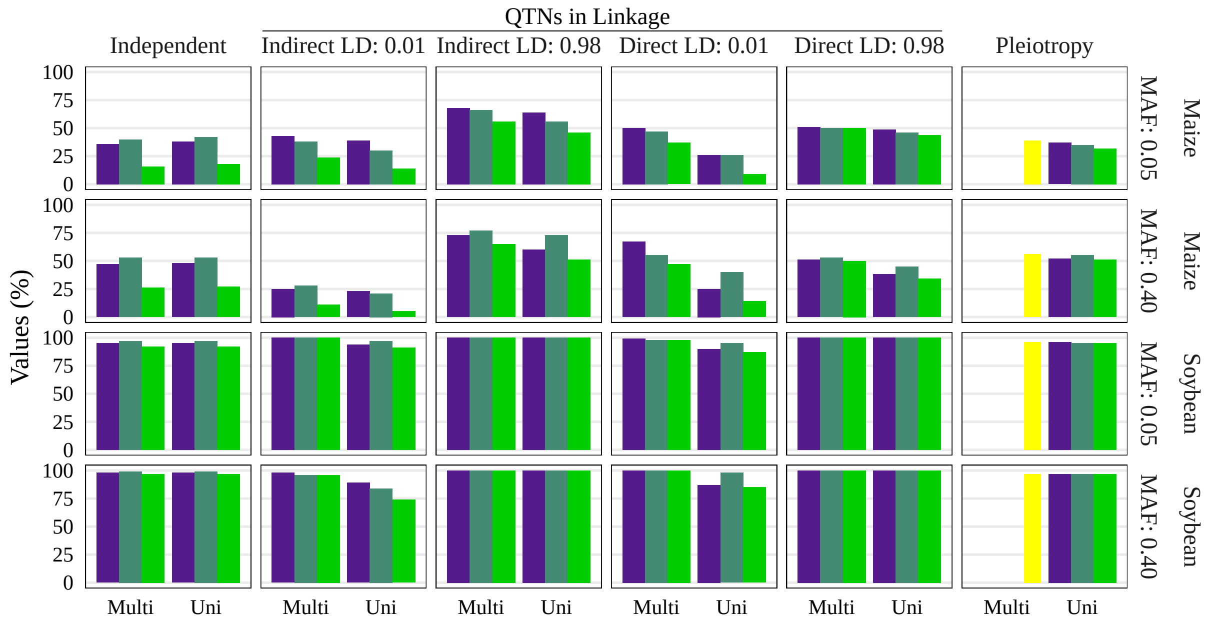

B

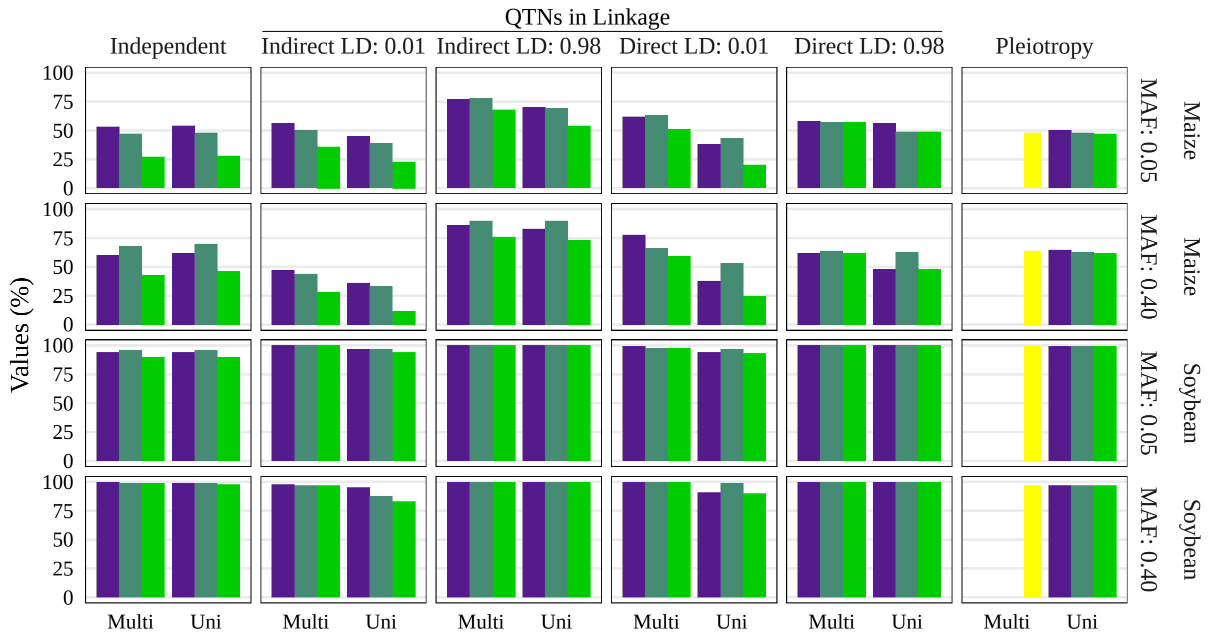

Figure S58: Error rate (measured as the detection of significant SNPs outside the window size) (Y-axis) achieved by multivariate (Multi) and univariate (Uni) GWAS (X-axis), relative to the QTN controlling trait 1 (T1), trait 2 (T2), and both QTN simultaneously (T1&T2) or, in the pleiotropic scenario, relative to the pleiotropic QTN (MT). The simulated genetic architecture is listed in the horizontal and vertical titles. These values were obtained with a sample size of 1, 000; A) a narrow-sense heritability of 0.3 for both traits; B) a narrow-sense heritability of 0.8 for both traits. MAF: minor allele frequencies. The false discovery rate rate was 0.1 and the window size was 10 kb for maize and 1 Mb for soybean.

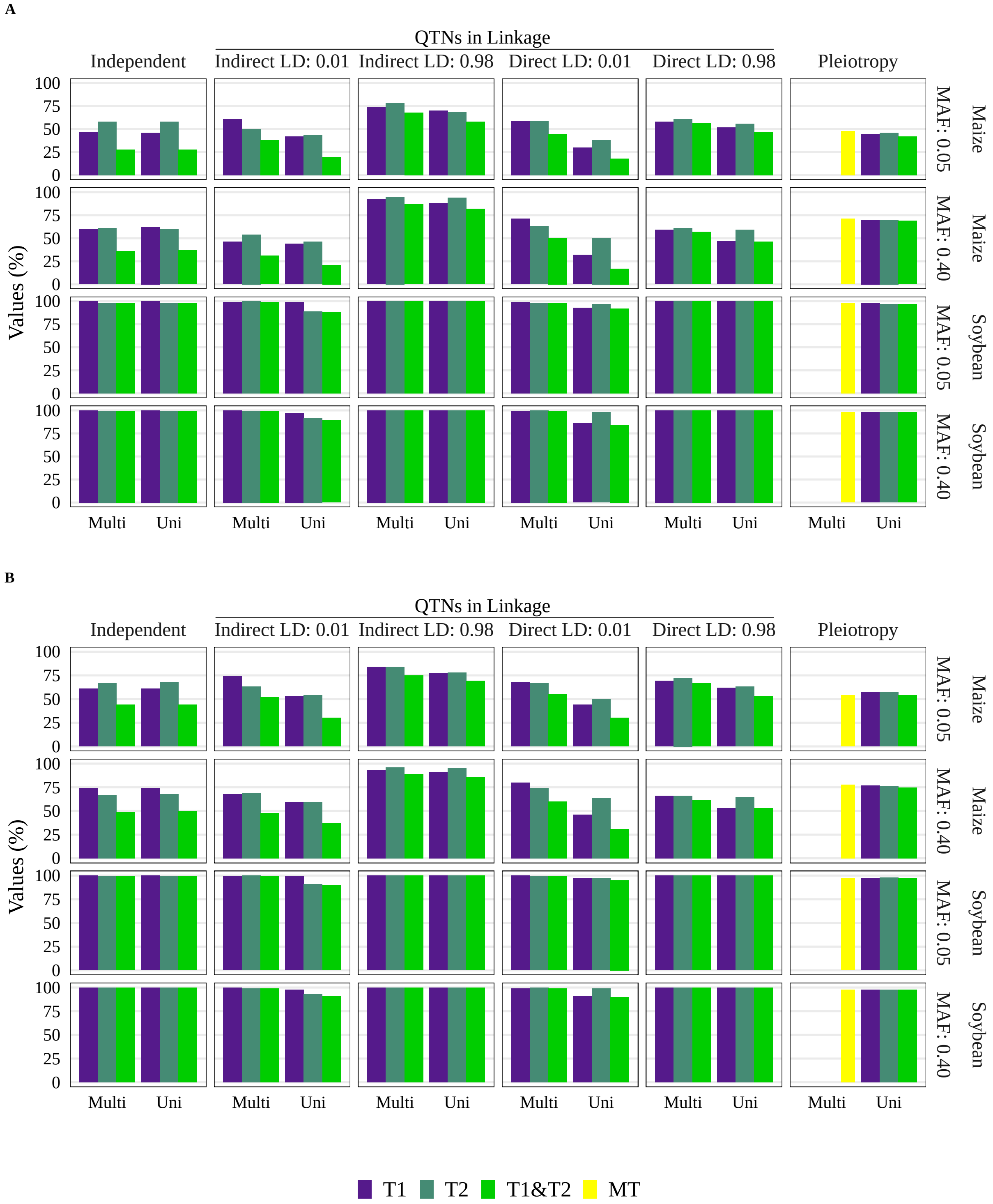

Figure S59: Error rate (measured as the detection of significant SNPs outside the window size) (Y-axis) achieved by multivariate (Multi) and univariate (Uni) GWAS (X-axis), relative to the QTN controlling trait 1 (T1), trait 2 (T2), and both QTN simultaneously (T1&T2) or, in the pleiotropic scenario, relative to the pleiotropic QTN (MT). The simulated genetic architecture is listed in the horizontal and vertical titles. These values were obtained with a sample size of 2, 815; A) a narrow-sense heritability of 0.3 for both traits; B) a narrow-sense heritability of 0.8 for both traits. MAF: minor allele frequencies. The false discovery rate rate was 0.1 and the window size was 10 kb for maize and 1 Mb for soybean.

Error rate with an FDR of 0.1 and window size of 1 Kb for maize and 10 kb for soybean

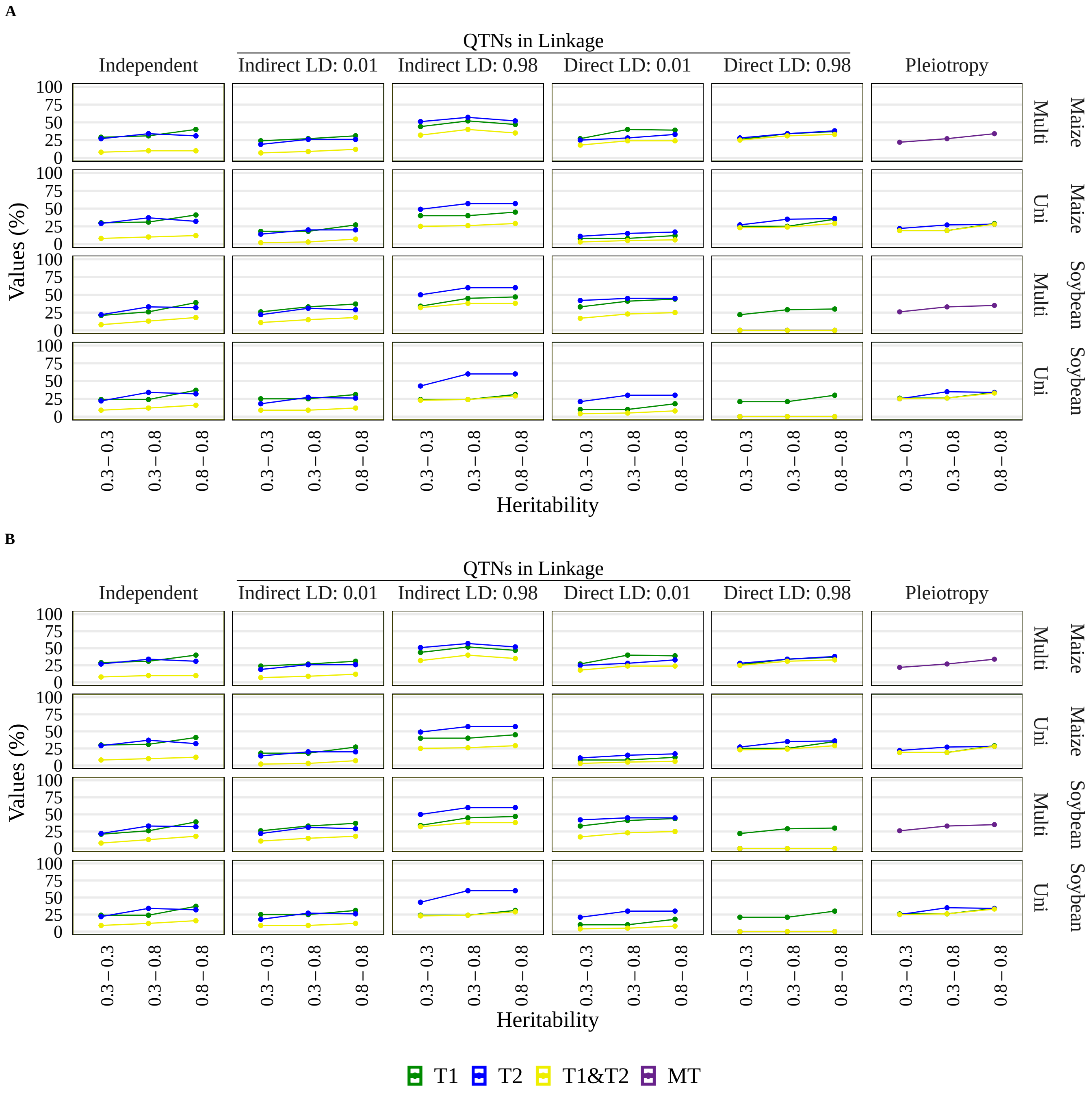

Figure S60: Error rate (measured as the detection of significant SNPs outside the window size) (Y-axis) achieved by multivariate (Multi) and univariate (Uni) GWAS, relative to the QTN controlling trait 1 (T1), trait 2 (T2), and both QTN simultaneously (T1&T2) or, in the pleiotropic scenario, relative to the pleiotropic QTN (MT). These values were obtained for maize and soybean with a sample size of 500. The X-axis displays the narrow-sense heritability for Trait 1 (bottom value) and Trait 2 (top value). A) inputted minor allele frequency (MAF) of 0.05; B) MAF of 0.4. The false discovery rate rate was 0.1 and the window size was 1 kb for maize and 10 Kb for soybean.

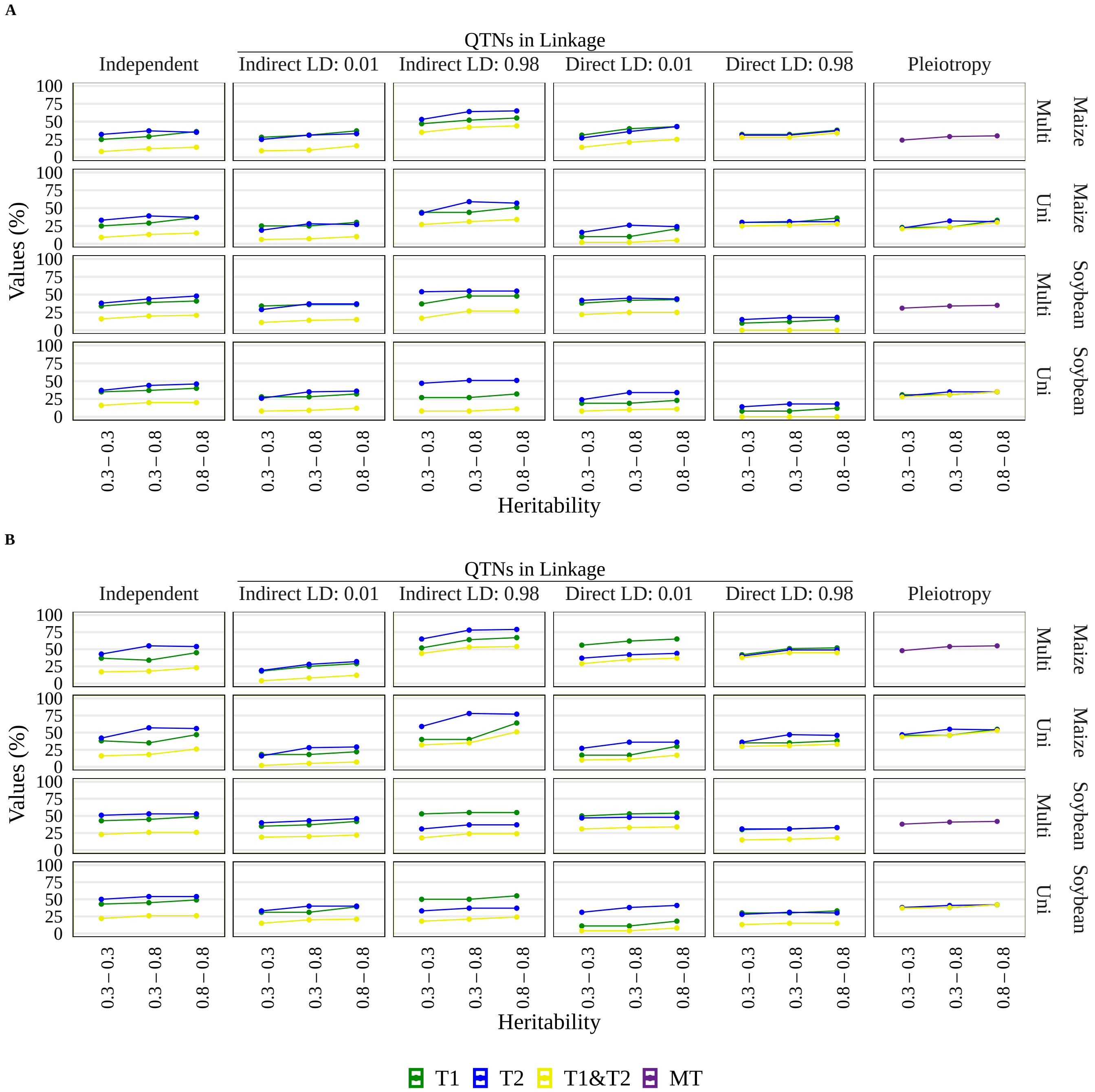

Figure S61: Error rate (measured as the detection of significant SNPs outside the window size) (Y-axis) achieved by multivariate (Multi) and univariate (Uni) GWAS, relative to the QTN controlling trait 1 (T1), trait 2 (T2), and both QTN simultaneously (T1&T2) or, in the pleiotropic scenario, relative to the pleiotropic QTN (MT). These values were obtained for maize and soybean with a sample size of 1,000. The X-axis displays the narrow-sense heritability for Trait 1 (bottom value) and Trait 2 (top value). A) inputted minor allele frequency (MAF) of 0.05; B) MAF of 0.4. The false discovery rate rate was 0.1 and the window size was 1 kb for maize and 10 Kb for soybean.

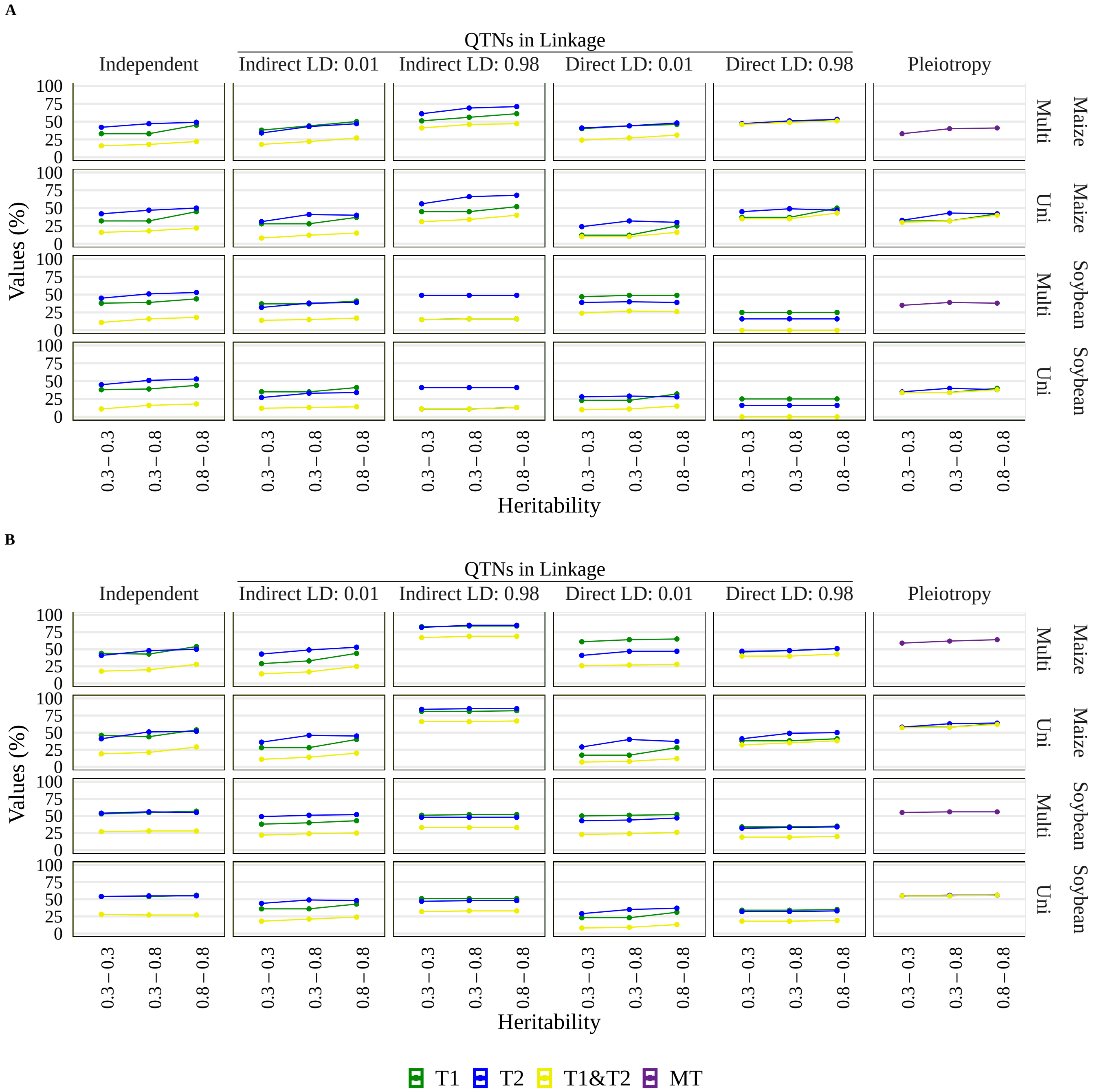

Figure S62: Error rate (measured as the detection of significant SNPs outside the window size) (Y-axis) achieved by multivariate (Multi) and univariate (Uni) GWAS, relative to the QTN controlling trait 1 (T1), trait 2 (T2), and both QTN simultaneously (T1&T2) or, in the pleiotropic scenario, relative to the pleiotropic QTN (MT). These values were obtained for maize and soybean with a sample size of 2,815. The X-axis displays the narrow-sense heritability for Trait 1 (bottom value) and Trait 2 (top value). A) inputted minor allele frequency (MAF) of 0.05; B) MAF of 0.4. The false discovery rate rate was 0.1 and the window size was 1 kb for maize and 10 Kb for soybean.

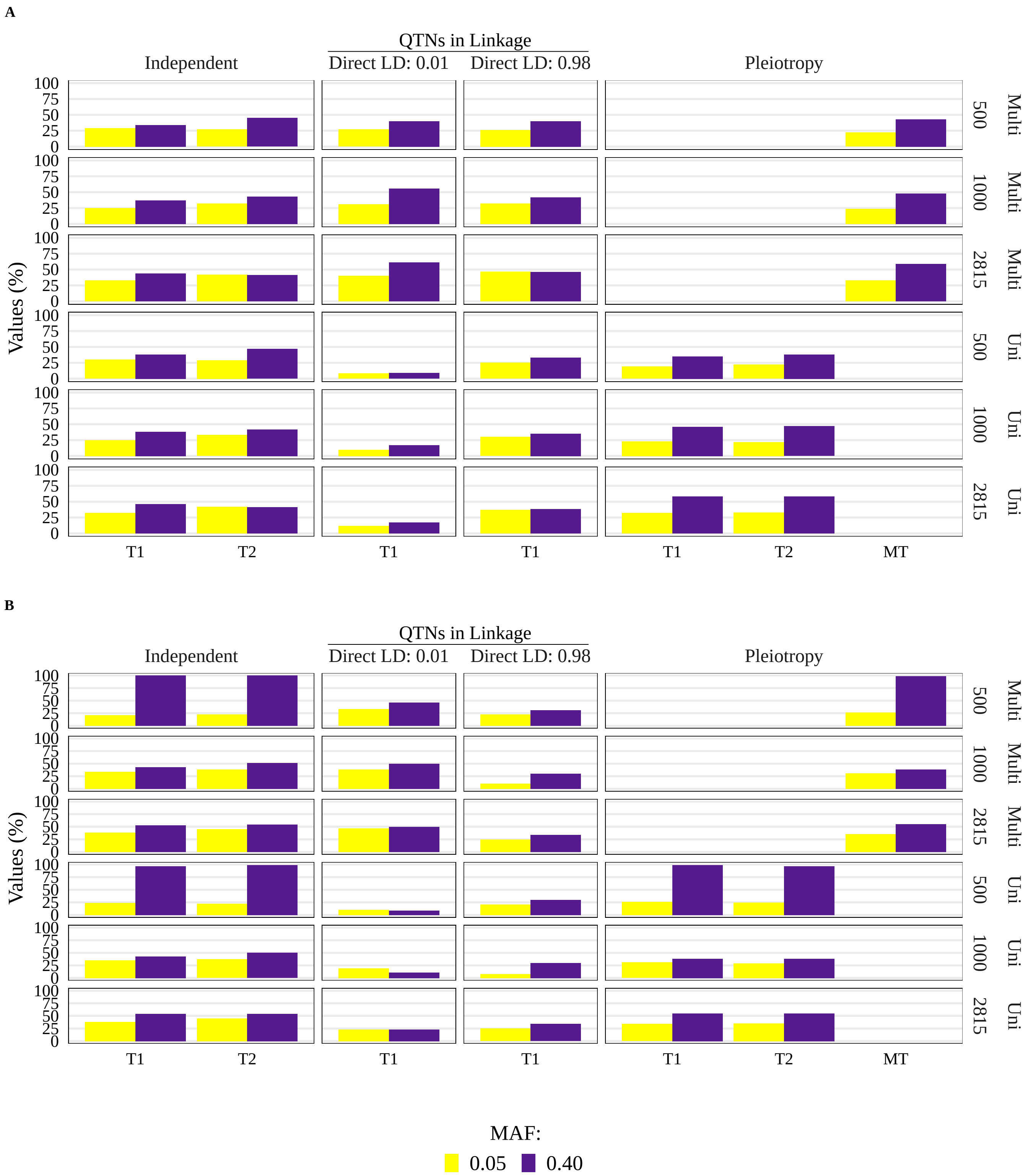

Figure S63: Error rate (measured as the detection of significant SNPs outside the window size) (Y-axis) in scenarios for which minor allele frequency (MAF) was directly controlled by a simulation input parameter. These values were obtained by multivariate (Multi) and univariate (Uni) GWAS, relative to the QTN controlling trait 1 (T1), trait 2 (T2) or, in the pleiotropic scenario, relative to the pleiotropic QTN (MT). This figure shows results for a narrow-sense heritability of 0.3 for both traits. A) Maize; B) Soybean. The false discovery rate rate was 0.1 and the window size was 1 kb for maize and 10 Kb for soybean.

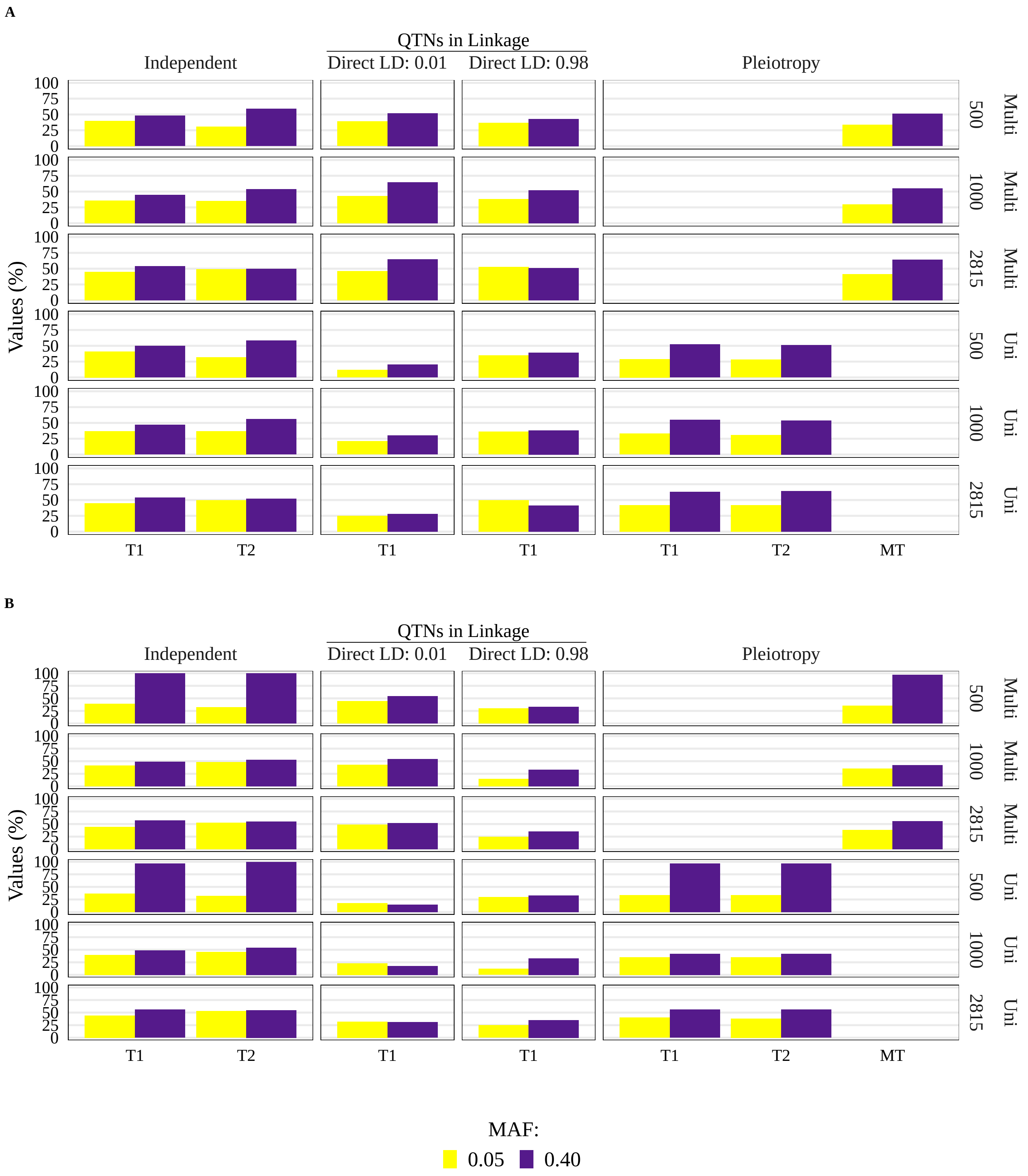

Figure S64: Error rate (measured as the detection of significant SNPs outside the window size) (Y-axis) in scenarios for which minor allele frequency (MAF) was directly controlled by a simulation input parameter. These values were obtained by multivariate (Multi) and univariate (Uni) GWAS, relative to the QTN controlling trait 1 (T1), trait 2 (T2) or, in the pleiotropic scenario, relative to the pleiotropic QTN (MT). This figure shows results for a narrow-sense heritability of 0.8 for both traits. A) Maize; B) Soybean. The false discovery rate rate was 0.1 and the window size was 1 kb for maize and 10 Kb for soybean.

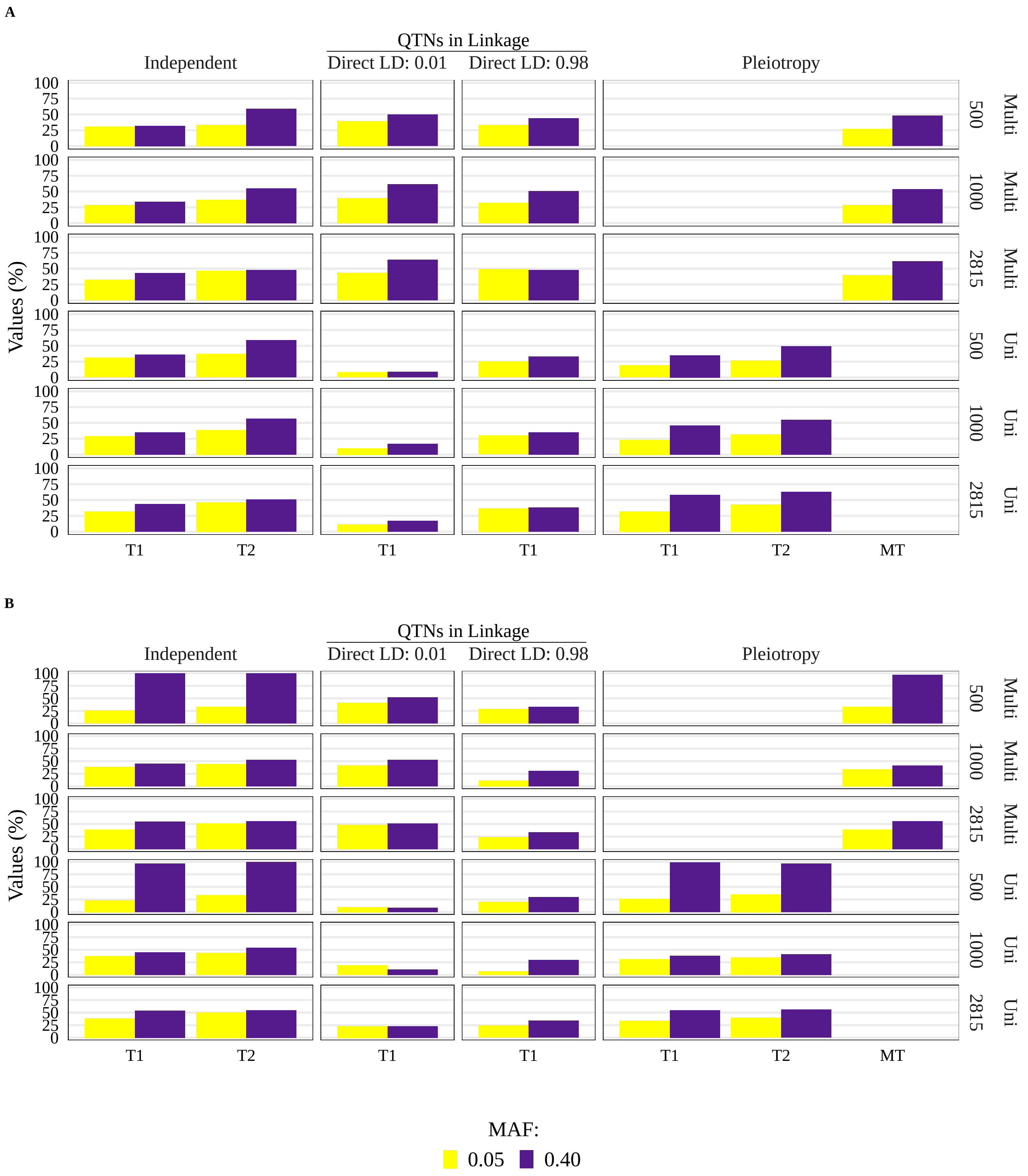

Figure S65: Error rate (measured as the detection of significant SNPs outside the window size) (Y-axis) in scenarios for which minor allele frequency (MAF) was directly controlled by a simulation input parameter. These values were obtained by multivariate (Multi) and univariate (Uni) GWAS, relative to the QTN controlling trait 1 (T1), trait 2 (T2) or, in the pleiotropic scenario, relative to the pleiotropic QTN (MT). This figure shows results for a narrow-sense heritability of 0.3 for trait 1 and 0.8 for trait 2. A) Maize; B) Soybean. The false discovery rate rate was 0.1 and the window size was 1 kb for maize and 10 Kb for soybean.

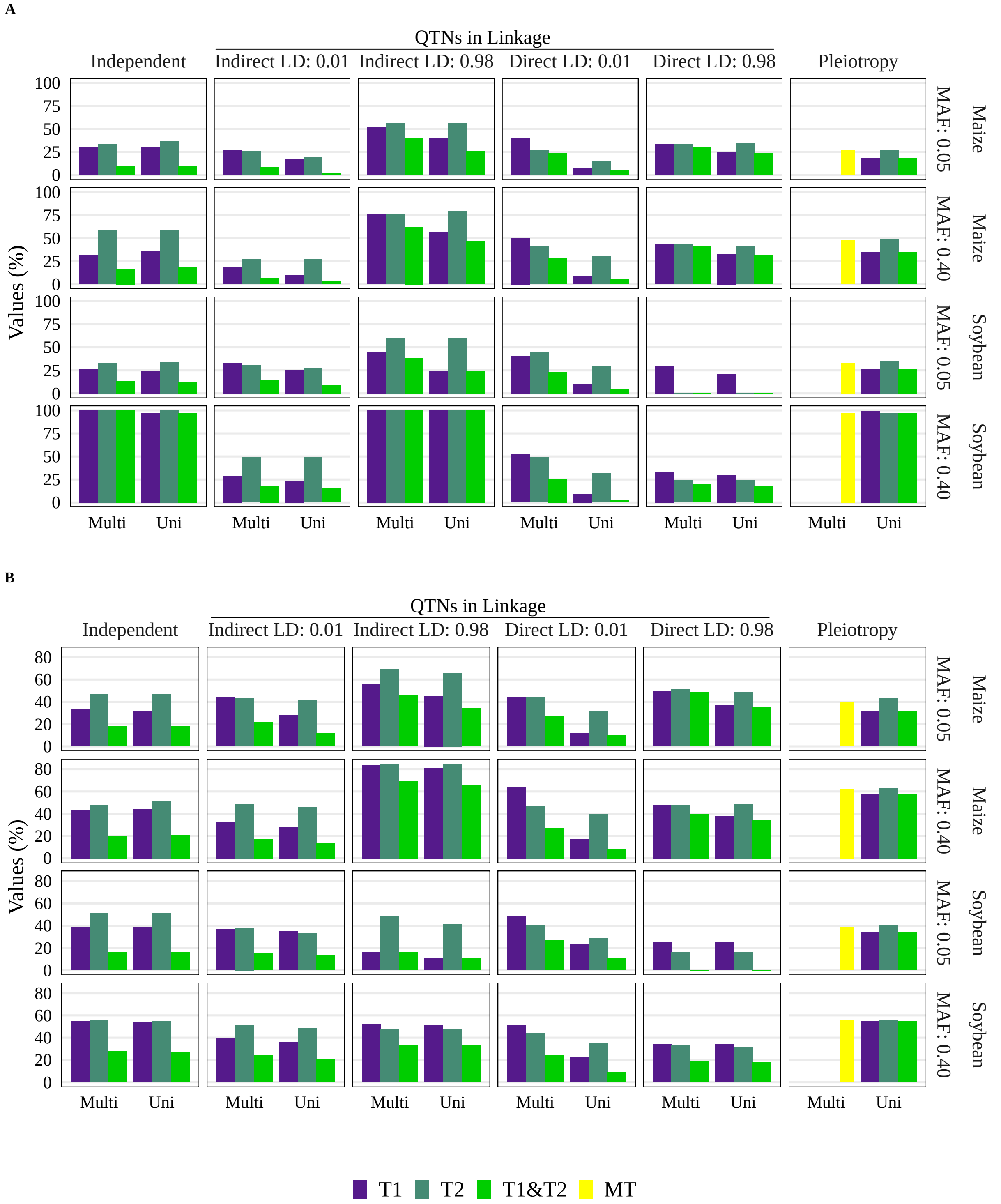

Figure S66: Error rate (measured as the detection of significant SNPs outside the window size) (Y-axis) achieved by multivariate (Multi) and univariate (Uni) GWAS (X-axis), relative to the QTN controlling trait 1 (T1), trait 2 (T2), and both QTN simultaneously (T1&T2) or, in the pleiotropic scenario, relative to the pleiotropic QTN (MT). The simulated genetic architecture is listed in the horizontal and vertical titles. These values were obtained with a narrow-sense heritability of 0.3 and 0.8 for traits 1 and 2, respectively. A) Sample size of 500; B) Sample size of 2815. MAF: minor allele frequencies. The false discovery rate rate was 0.1 and the window size was 1 kb for maize and 10 Kb for soybean.

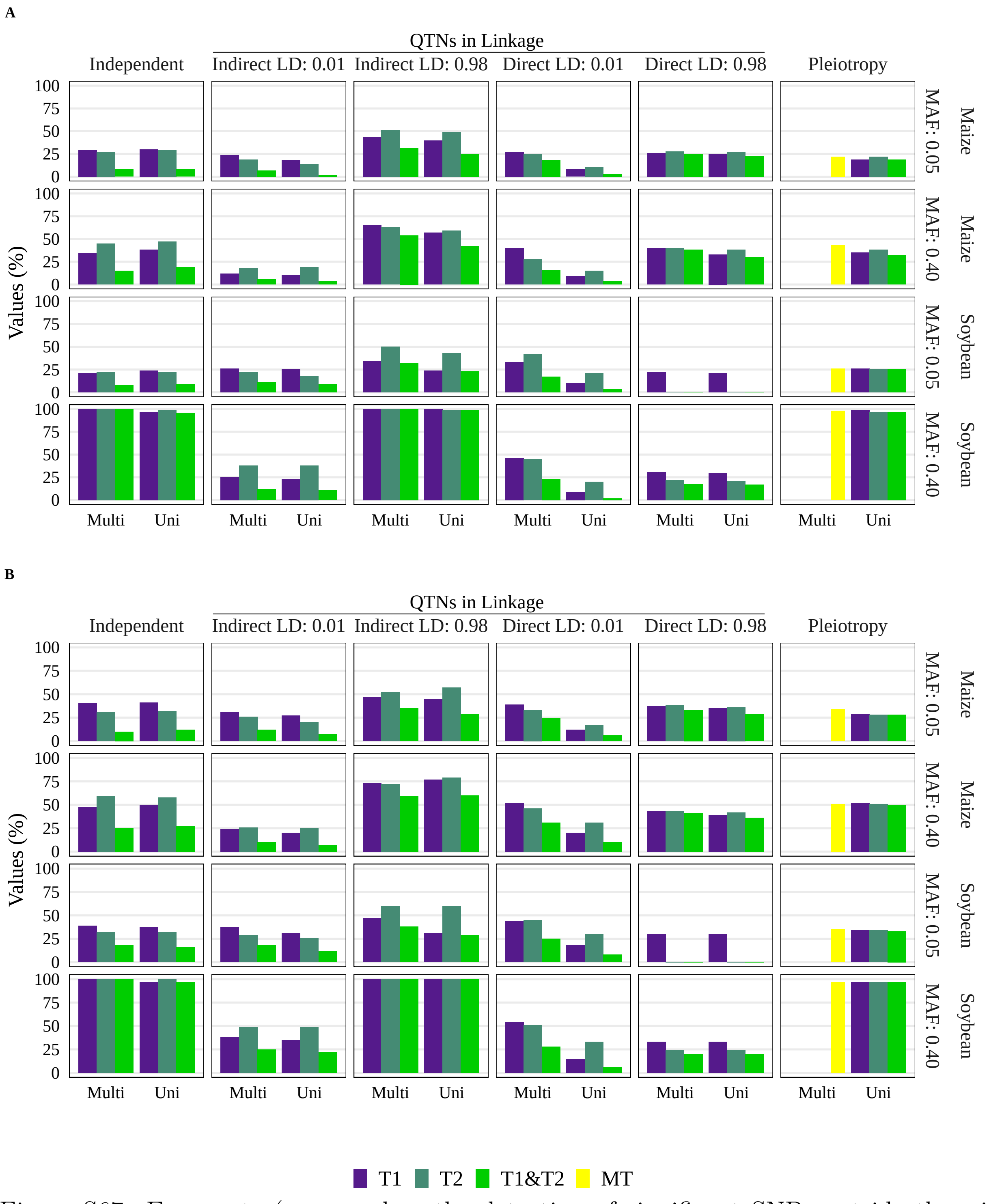

Figure S67: Error rate (measured as the detection of significant SNPs outside the window size) (Y-axis) achieved by multivariate (Multi) and univariate (Uni) GWAS (X-axis), relative to the QTN controlling trait 1 (T1), trait 2 (T2), and both QTN simultaneously (T1&T2) or, in the pleiotropic scenario, relative to the pleiotropic QTN (MT). The simulated genetic architecture is listed in the horizontal and vertical titles. These values were obtained with a sample size of 500; A) a narrow-sense heritability of 0.3 for both traits; B) a narrow-sense heritability of 0.8 for both traits. MAF: minor allele frequencies. The false discovery rate rate was 0.1 and the window size was 1 kb for maize and 10 Kb for soybean.

A

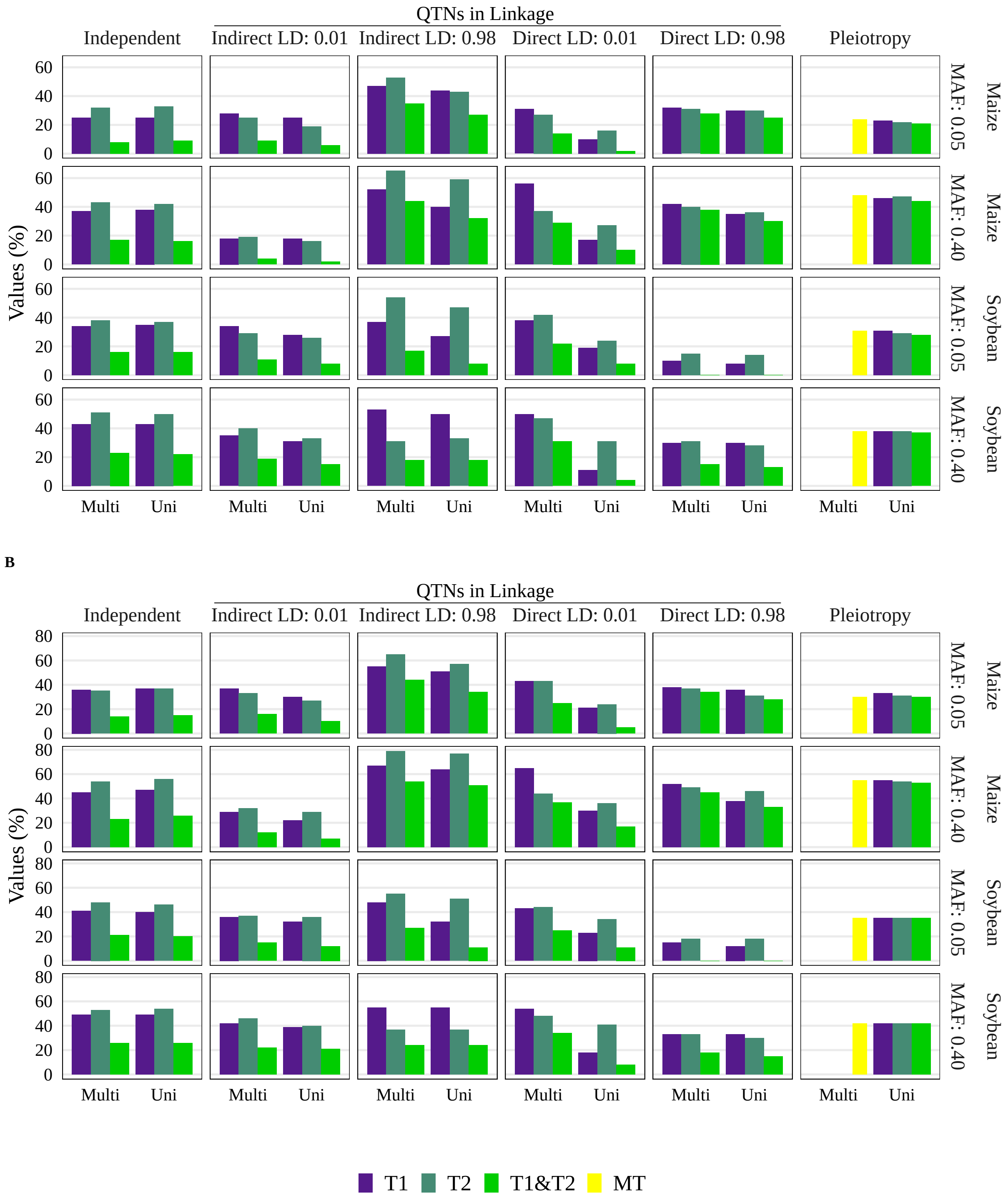

Figure S68: Error rate (measured as the detection of significant SNPs outside the window size) (Y-axis) achieved by multivariate (Multi) and univariate (Uni) GWAS (X-axis), relative to the QTN controlling trait 1 (T1), trait 2 (T2), and both QTN simultaneously (T1&T2) or, in the pleiotropic scenario, relative to the pleiotropic QTN (MT). The simulated genetic architecture is listed in the horizontal and vertical titles. These values were obtained with a sample size of 1, 000; A) a narrow-sense heritability of 0.3 for both traits; B) a narrow-sense heritability of 0.8 for both traits. MAF: minor allele frequencies. The false discovery rate rate was 0.1 and the window size was 1 kb for maize and 10 Kb for soybean.

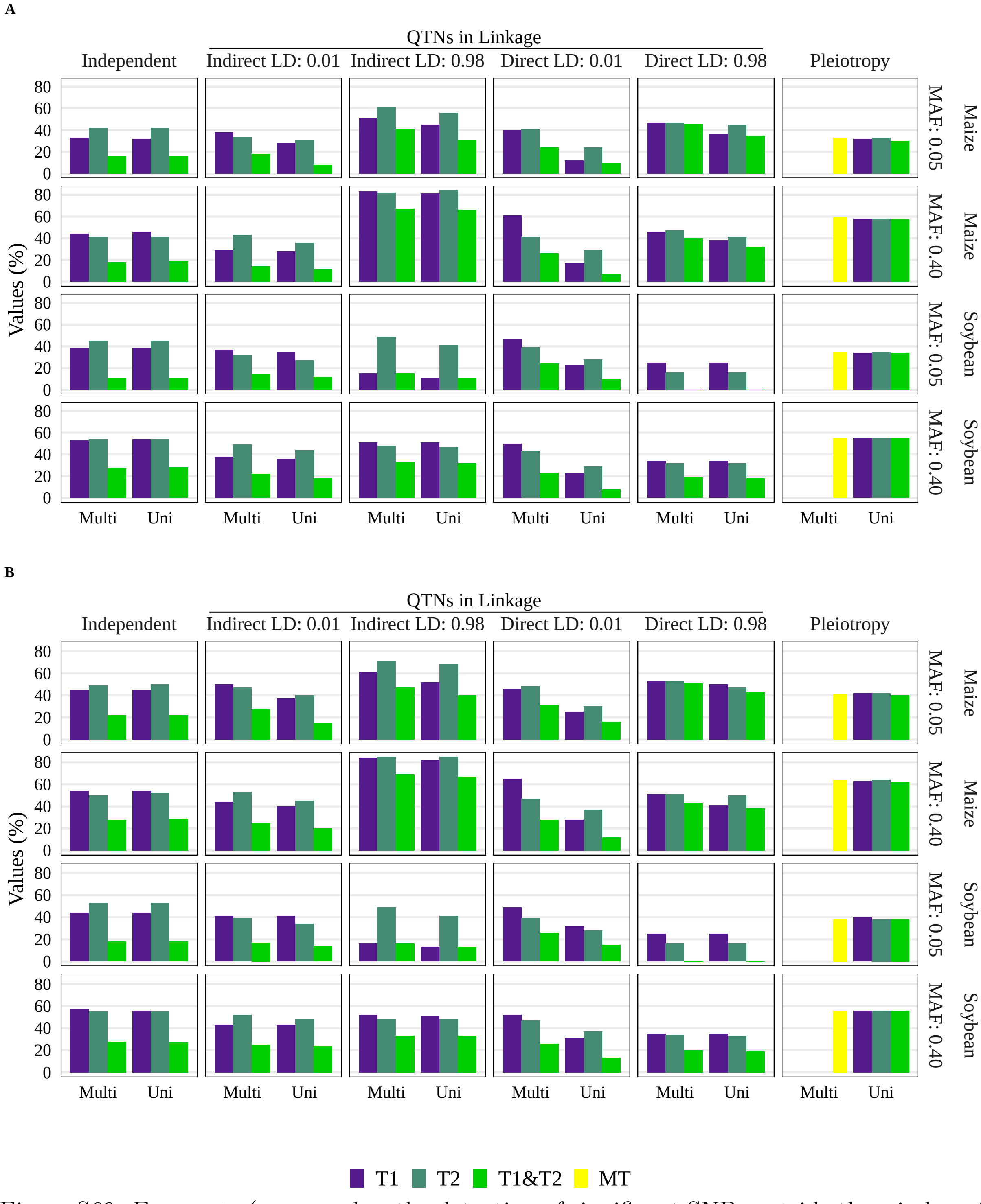

Figure S69: Error rate (measured as the detection of significant SNPs outside the window size) (Y-axis) achieved by multivariate (Multi) and univariate (Uni) GWAS (X-axis), relative to the QTN controlling trait 1 (T1), trait 2 (T2), and both QTN simultaneously (T1&T2) or, in the pleiotropic scenario, relative to the pleiotropic QTN (MT). The simulated genetic architecture is listed in the horizontal and vertical titles. These values were obtained with a sample size of 2, 815; A) a narrow-sense heritability of 0.3 for both traits; B) a narrow-sense heritability of 0.8 for both traits. MAF: minor allele frequencies. The false discovery rate rate was 0.1 and the window size was 1 kb for maize and 10 Kb for soybean.

Error rate with an FDR of 0.05 and window size of 10 Kb for maize and 1Mb for soybean

A

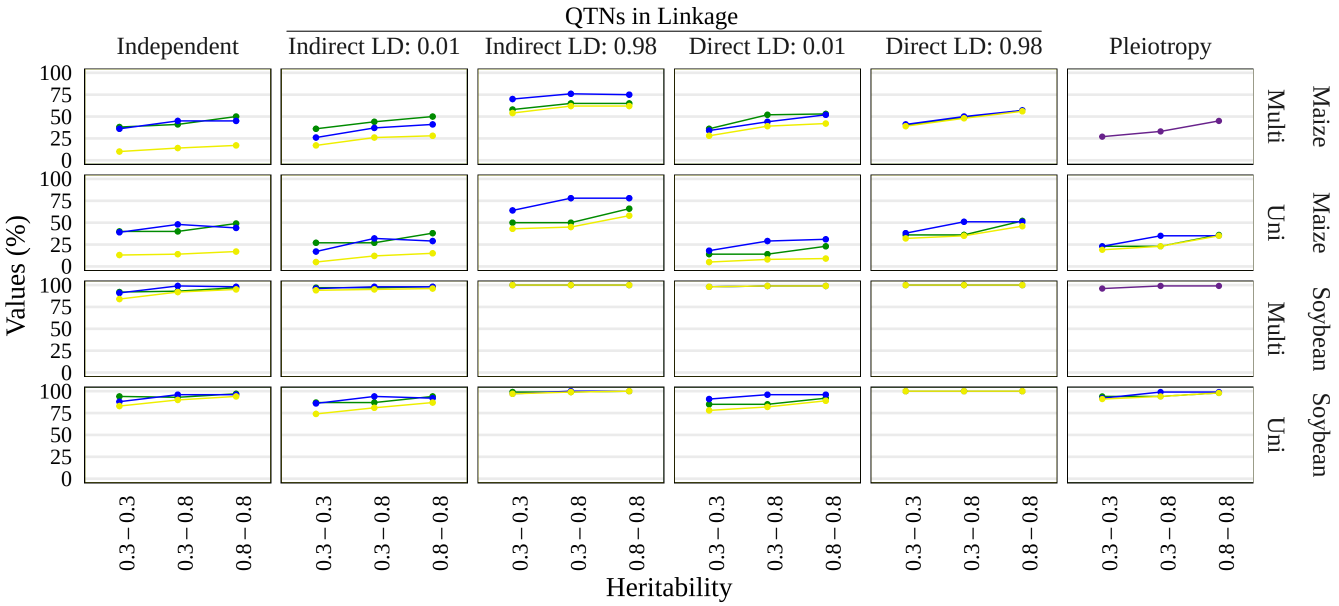

B

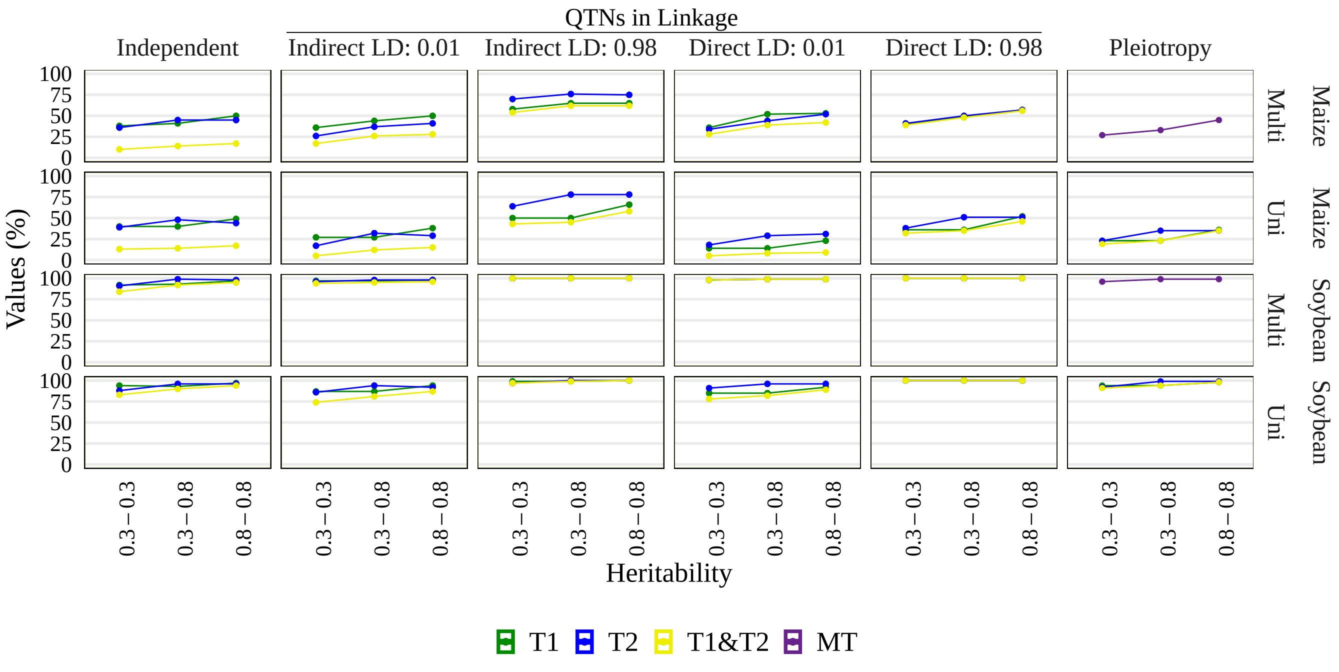

Figure S70: Error rate (measured as the detection of significant SNPs outside the window size) (Y-axis) achieved by multivariate (Multi) and univariate (Uni) GWAS, relative to the QTN controlling trait 1 (T1), trait 2 (T2), and both QTN simultaneously (T1&T2) or, in the pleiotropic scenario, relative to the pleiotropic QTN (MT). These values were obtained for maize and soybean with a sample size of 500. The X-axis displays the narrow-sense heritability for Trait 1 (bottom value) and Trait 2 (top value). A) inputted minor allele frequency (MAF) of 0.05; B) MAF of 0.4. The false discovery rate rate was 0.05 and the window size was 10 kb for maize and 1 Mb for soybean.

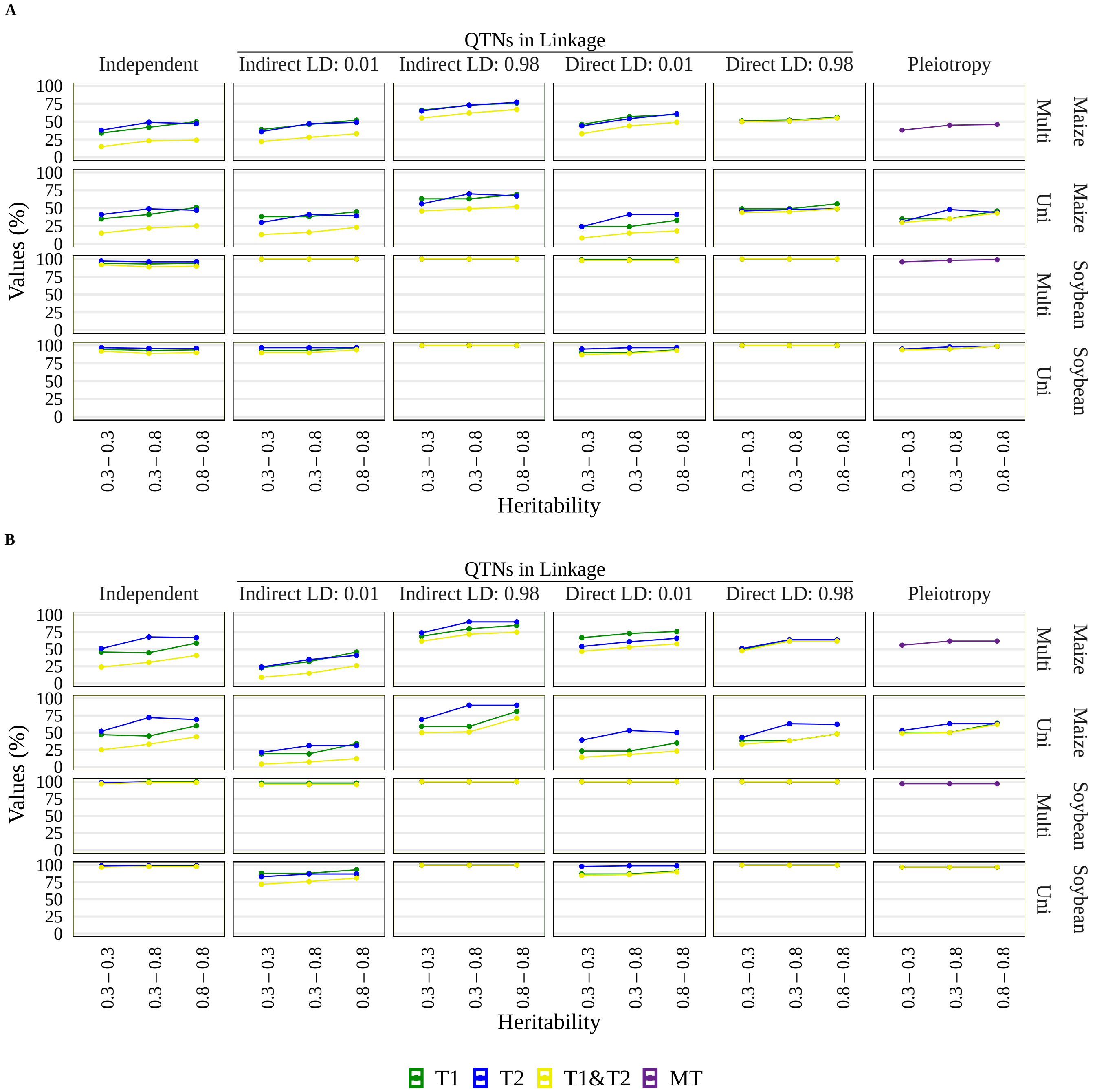

Figure S71: Error rate (measured as the detection of significant SNPs outside the window size) (Y-axis) achieved by multivariate (Multi) and univariate (Uni) GWAS, relative to the QTN controlling trait 1 (T1), trait 2 (T2), and both QTN simultaneously (T1&T2) or, in the pleiotropic scenario, relative to the pleiotropic QTN (MT). These values were obtained for maize and soybean with a sample size of 1,000. The X-axis displays the narrow-sense heritability for Trait 1 (bottom value) and Trait 2 (top value). A) inputted minor allele frequency (MAF) of 0.05; B) MAF of 0.4. The false discovery rate rate was 0.05 and the window size was 10 kb for maize and 1 Mb for soybean.

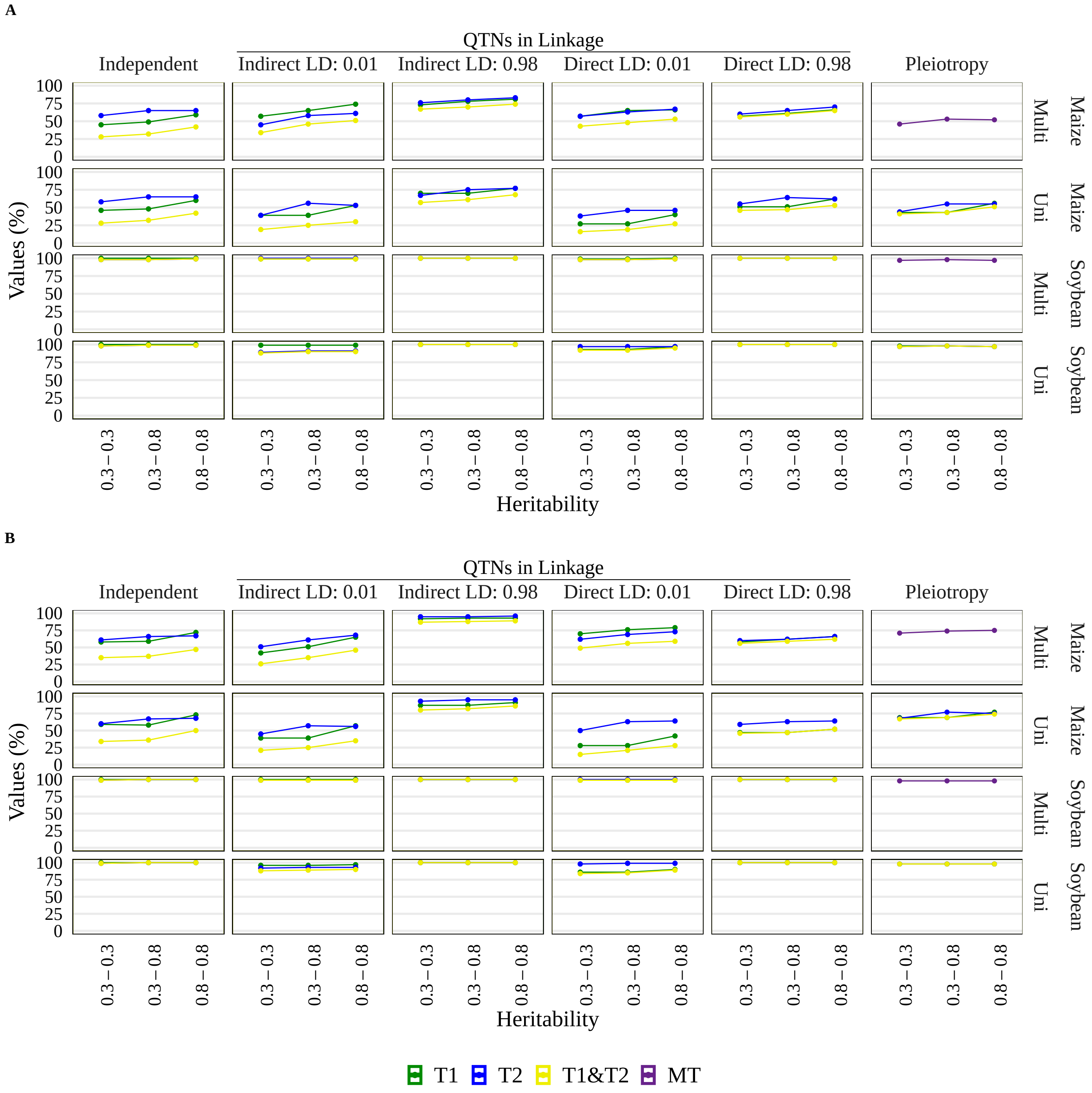

Figure S72: Error rate (measured as the detection of significant SNPs outside the window size) (Y-axis) achieved by multivariate (Multi) and univariate (Uni) GWAS, relative to the QTN controlling trait 1 (T1), trait 2 (T2), and both QTN simultaneously (T1&T2) or, in the pleiotropic scenario, relative to the pleiotropic QTN (MT). These values were obtained for maize and soybean with a sample size of 2,815. The X-axis displays the narrow-sense heritability for Trait 1 (bottom value) and Trait 2 (top value). A) inputted minor allele frequency (MAF) of 0.05; B) MAF of 0.4. The false discovery rate rate was 0.05 and the window size was 10 kb for maize and 1 Mb for soybean.

A

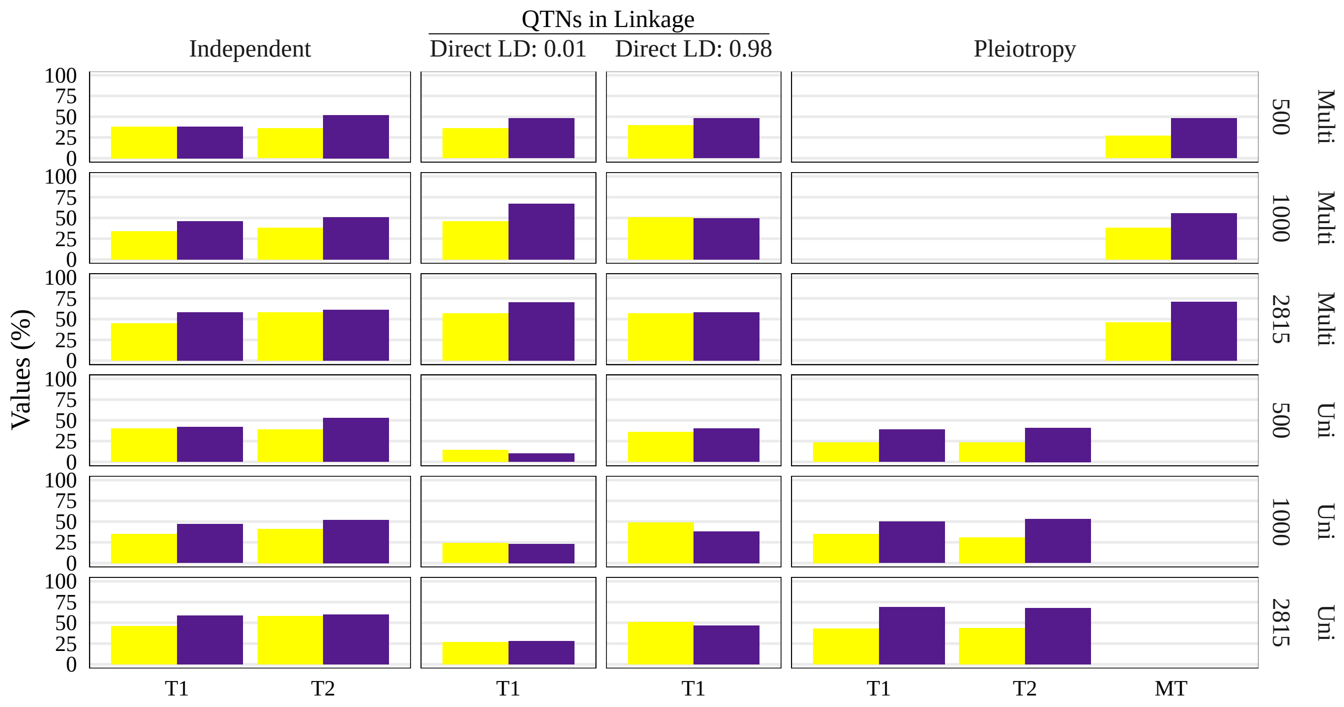

B

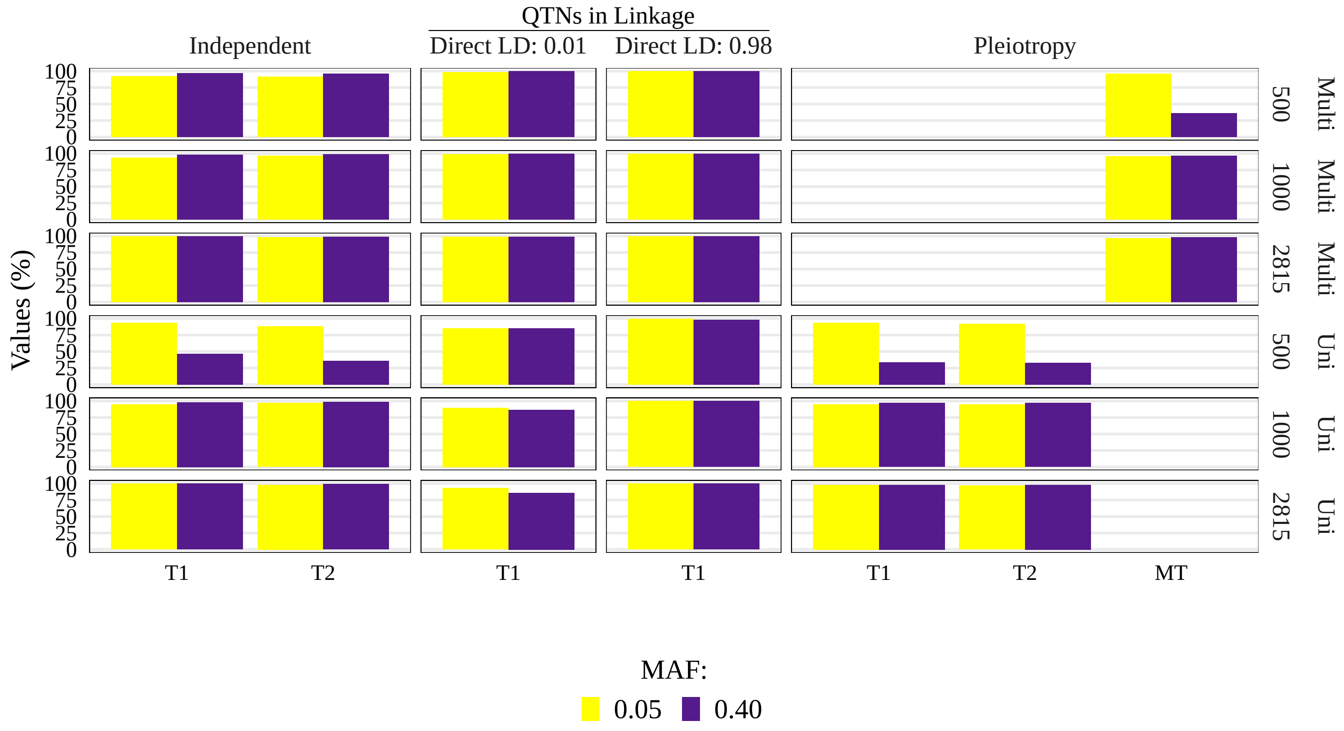

Figure S73: Error rate (measured as the detection of significant SNPs outside the window size) (Y-axis) in scenarios for which minor allele frequency (MAF) was directly controlled by a simulation input parameter. These values were obtained by multivariate (Multi) and univariate (Uni) GWAS, relative to the QTN controlling trait 1 (T1), trait 2 (T2) or, in the pleiotropic scenario, relative to the pleiotropic QTN (MT). This figure shows results for a narrow-sense heritability of 0.3 for both traits. A) Maize; B) Soybean. The false discovery rate rate was 0.05 and the window size was 10 kb for maize and 1 Mb for soybean.

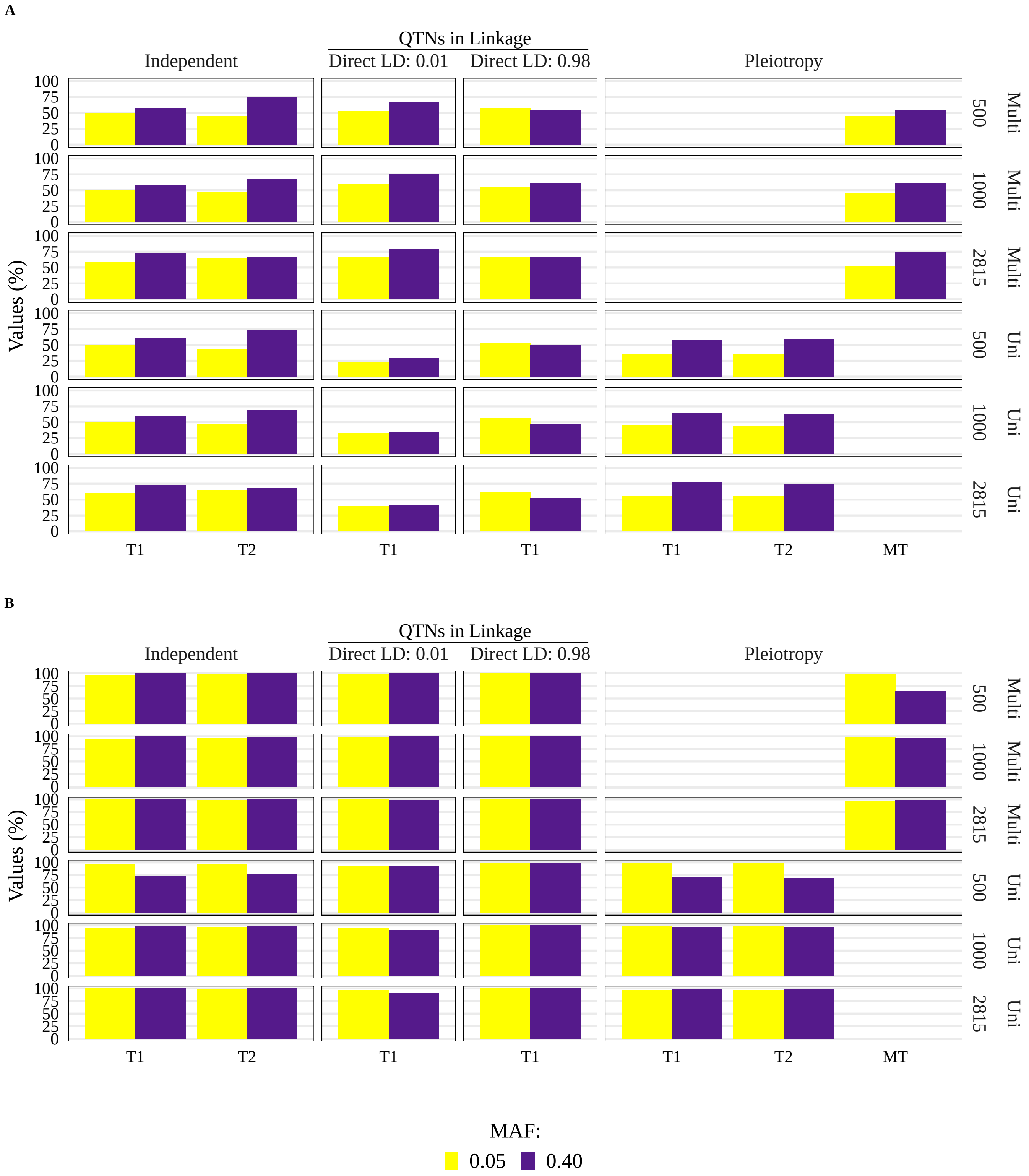

Figure S74: Error rate (measured as the detection of significant SNPs outside the window size) (Y-axis) in scenarios for which minor allele frequency (MAF) was directly controlled by a simulation input parameter. These values were obtained by multivariate (Multi) and univariate (Uni) GWAS, relative to the QTN controlling trait 1 (T1), trait 2 (T2) or, in the pleiotropic scenario, relative to the pleiotropic QTN (MT). This figure shows results for a narrow-sense heritability of 0.8 for both traits. A) Maize; B) Soybean. The false discovery rate rate was 0.05 and the window size was 10 kb for maize and 1 Mb for soybean.

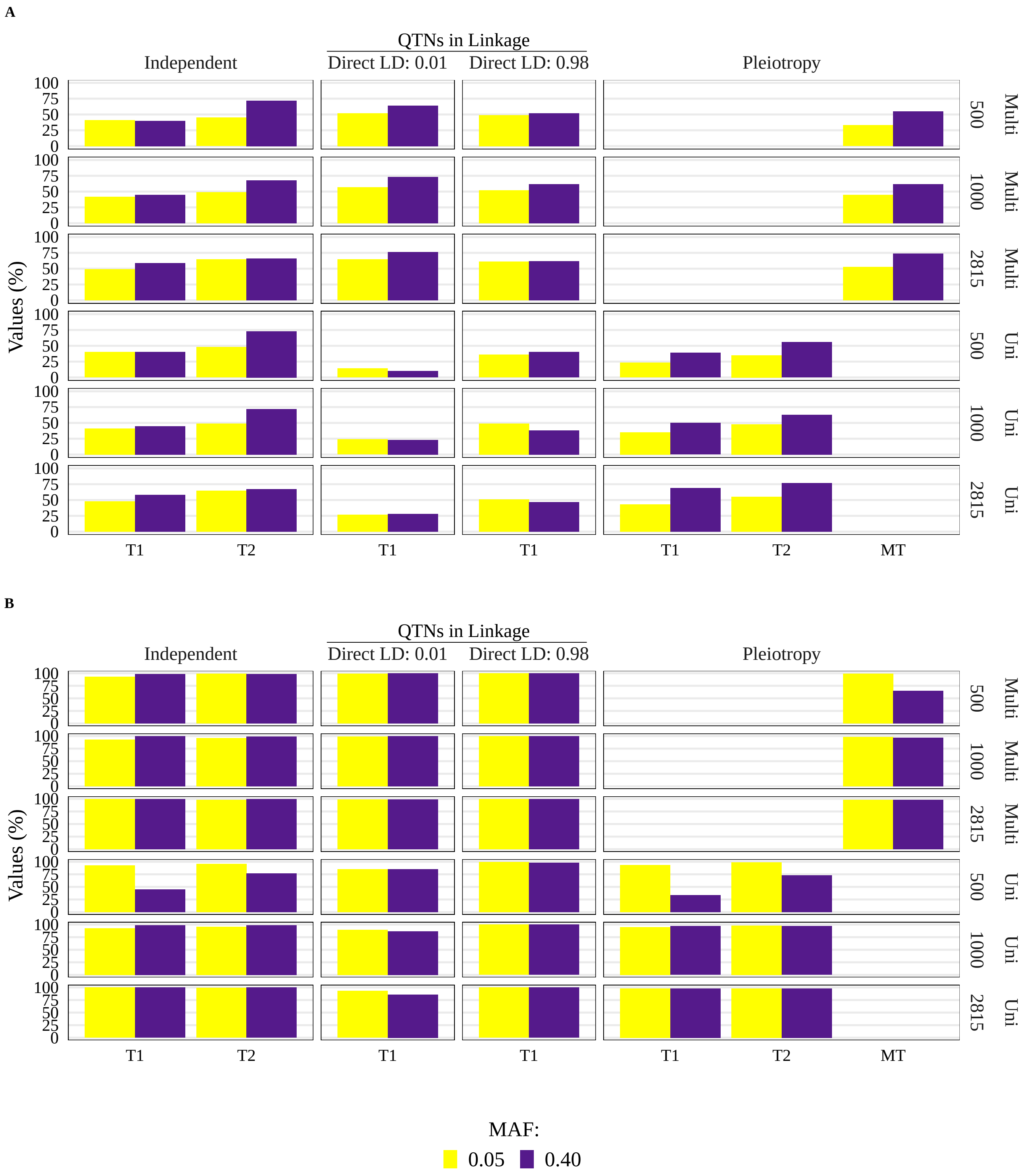

Figure S75: Error rate (measured as the detection of significant SNPs outside the window size) (Y-axis) in scenarios for which minor allele frequency (MAF) was directly controlled by a simulation input parameter. These values were obtained by multivariate (Multi) and univariate (Uni) GWAS, relative to the QTN controlling trait 1 (T1), trait 2 (T2) or, in the pleiotropic scenario, relative to the pleiotropic QTN (MT). This figure shows results for a narrow-sense heritability of 0.3 for trait 1 and 0.8 for trait 2. A) Maize; B) Soybean. The false discovery rate rate was 0.05 and the window size was 10 kb for maize and 1 Mb for soybean.

A

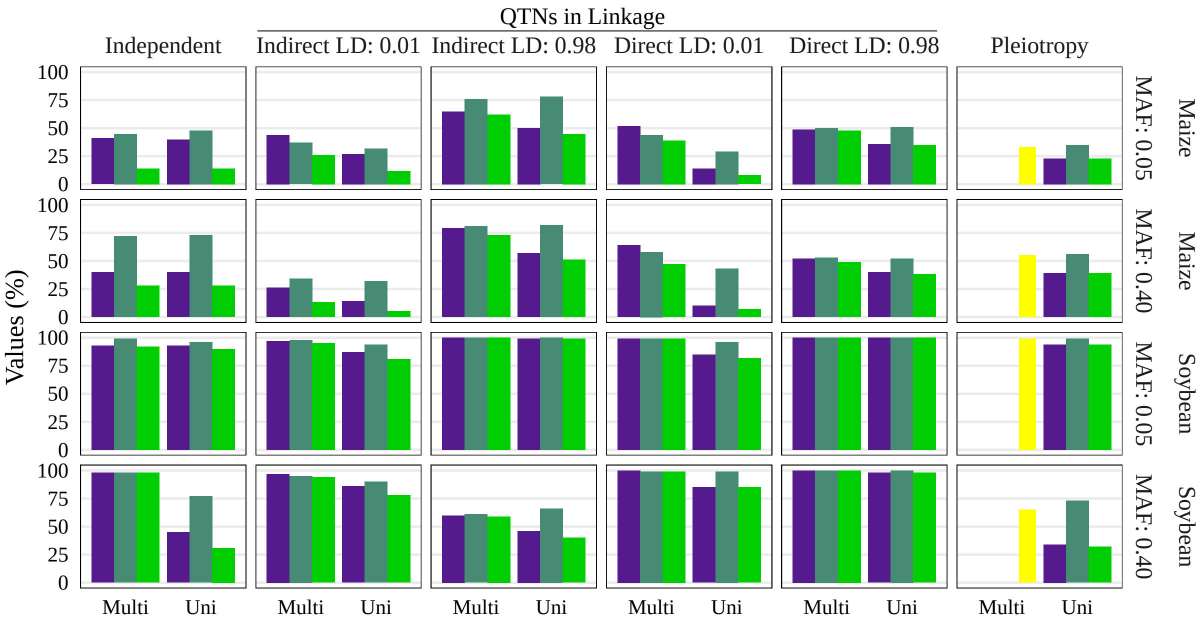

B

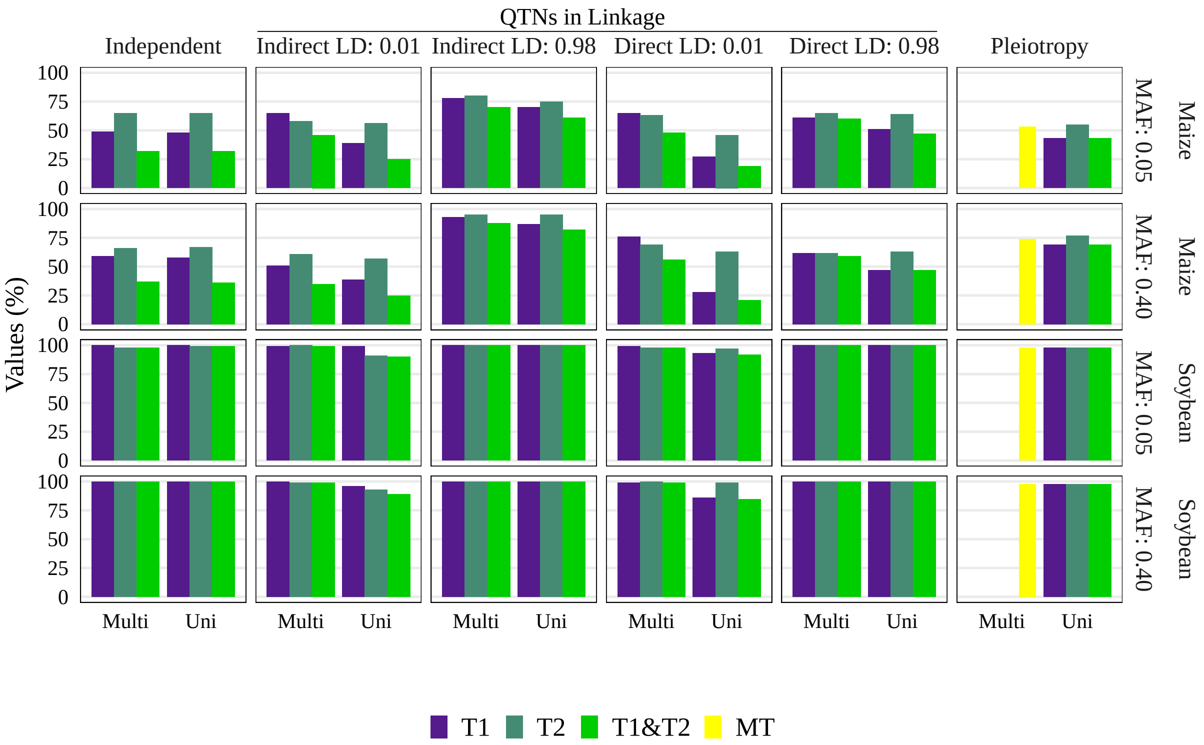

Figure S76: Error rate (measured as the detection of significant SNPs outside the window size) (Y-axis) achieved by multivariate (Multi) and univariate (Uni) GWAS (X-axis), relative to the QTN controlling trait 1 (T1), trait 2 (T2), and both QTN simultaneously (T1&T2) or, in the pleiotropic scenario, relative to the pleiotropic QTN (MT). The simulated genetic architecture is listed in the horizontal and vertical titles. These values were obtained with a narrow-sense heritability of 0.3 and 0.8 for traits 1 and 2, respectively. A) Sample size of 500; B) Sample size of 2815. MAF: minor allele frequencies. The false discovery rate rate was 0.05 and the window size was 10 kb for maize and 1 Mb for soybean.

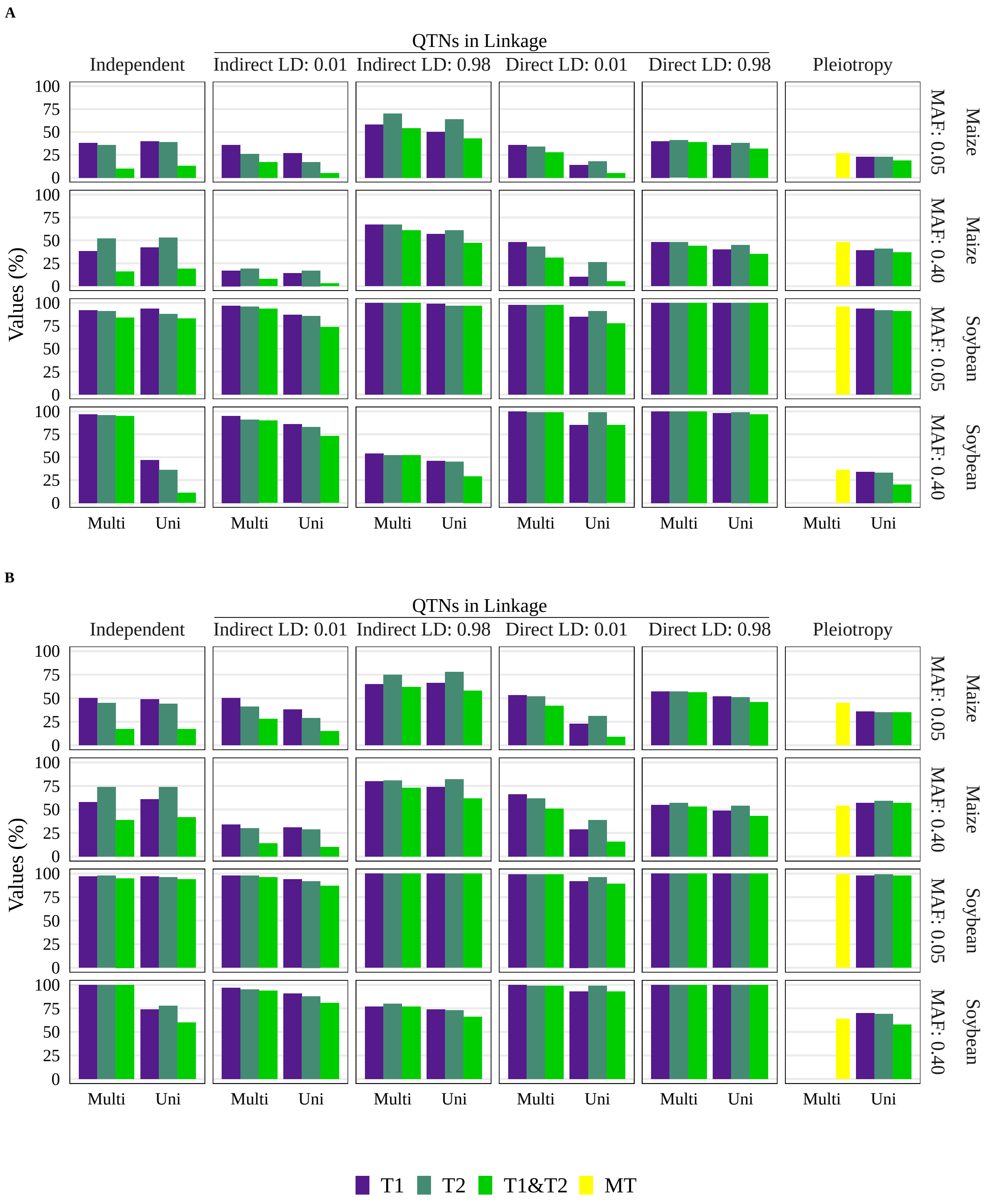

Figure S77: Error rate (measured as the detection of significant SNPs outside the window size) (Y-axis) achieved by multivariate (Multi) and univariate (Uni) GWAS (X-axis), relative to the QTN controlling trait 1 (T1), trait 2 (T2), and both QTN simultaneously (T1&T2) or, in the pleiotropic scenario, relative to the pleiotropic QTN (MT). The simulated genetic architecture is listed in the horizontal and vertical titles. These values were obtained with a sample size of 500; A) a narrow-sense heritability of 0.3 for both traits; B) a narrow-sense heritability of 0.8 for both traits. MAF: minor allele frequencies. The false discovery rate rate was 0.05 and the window size was 10 kb for maize and 1 Mb for soybean.

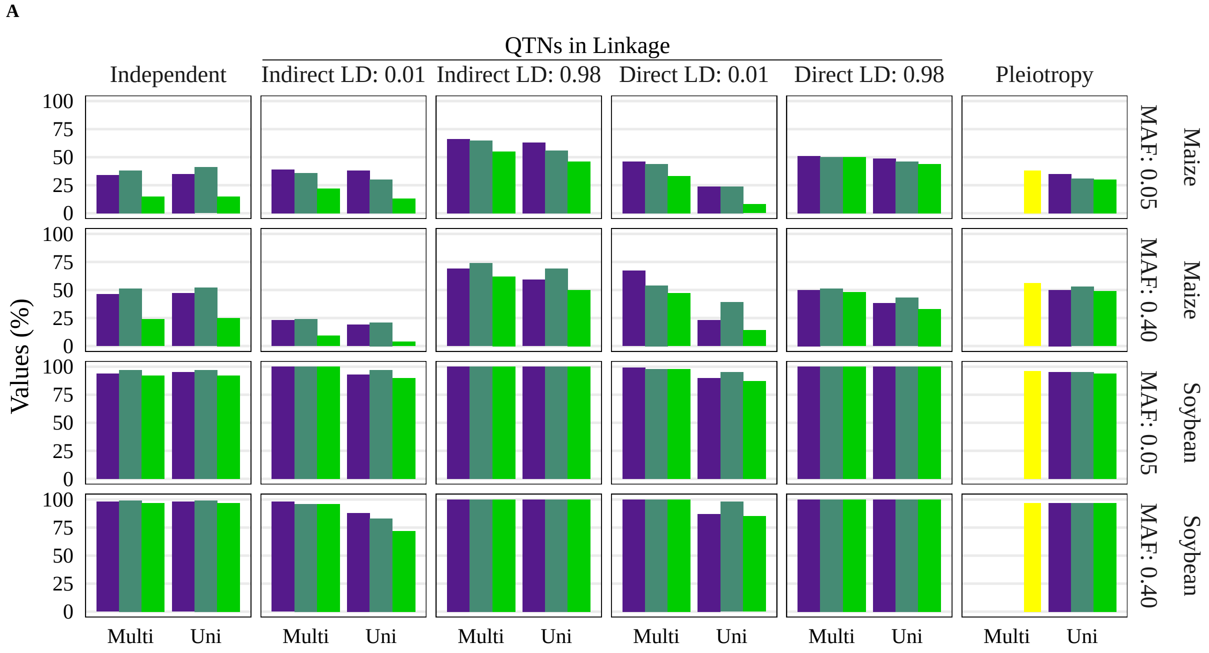

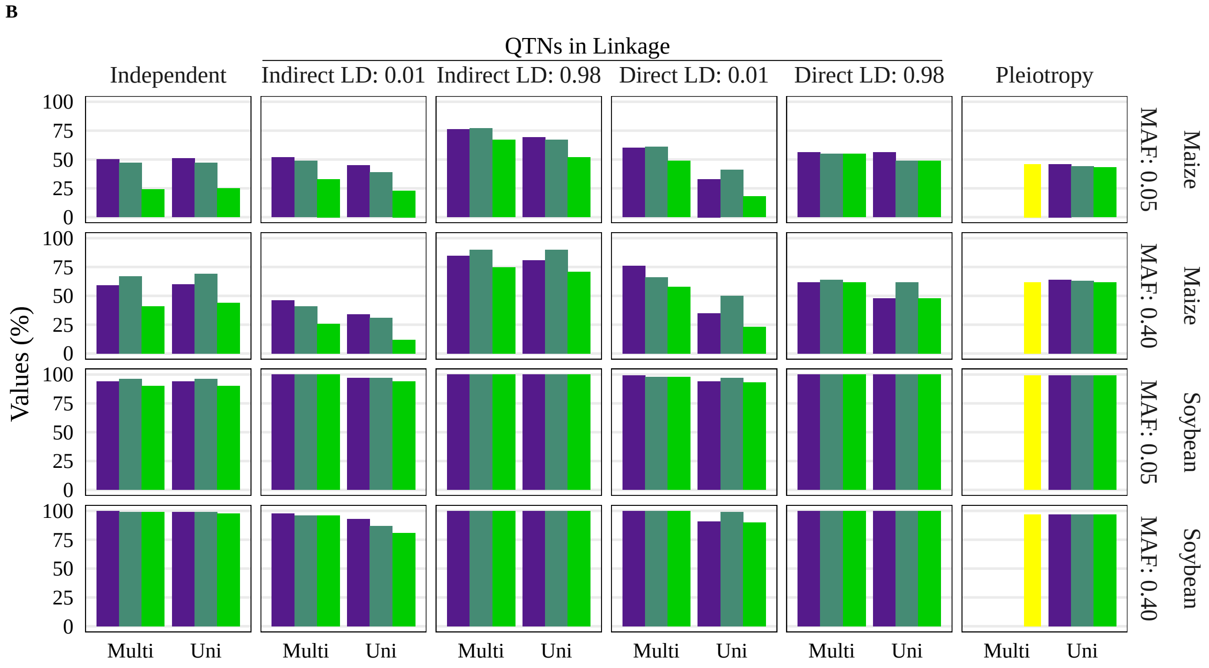

Figure S78: Error rate (measured as the detection of significant SNPs outside the window size) (Y-axis) achieved by multivariate (Multi) and univariate (Uni) GWAS (X-axis), relative to the QTN controlling trait 1 (T1), trait 2 (T2), and both QTN simultaneously (T1&T2) or, in the pleiotropic scenario, relative to the pleiotropic QTN (MT). The simulated genetic architecture is listed in the horizontal and vertical titles. These values were obtained with a sample size of 1, 000; A) a narrow-sense heritability of 0.3 for both traits; B) a narrow-sense heritability of 0.8 for both traits. MAF: minor allele frequencies. The false discovery rate rate was 0.05 and the window size was 10 kb for maize and 1 Mb for soybean.

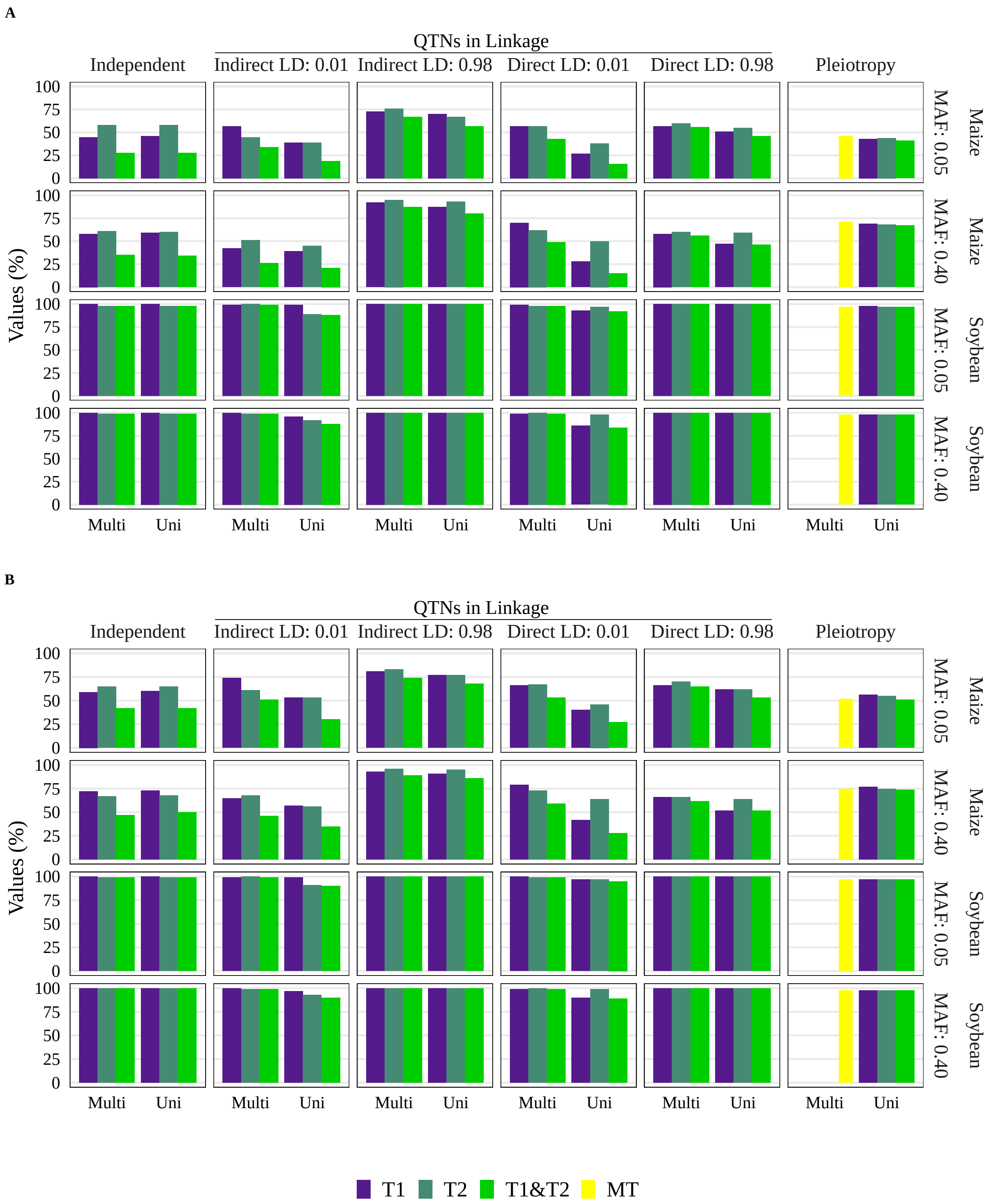

Figure S79: Error rate (measured as the detection of significant SNPs outside the window size) (Y-axis) achieved by multivariate (Multi) and univariate (Uni) GWAS (X-axis), relative to the QTN controlling trait 1 (T1), trait 2 (T2), and both QTN simultaneously (T1&T2) or, in the pleiotropic scenario, relative to the pleiotropic QTN (MT). The simulated genetic architecture is listed in the horizontal and vertical titles. These values were obtained with a sample size of 2, 815; A) a narrow-sense heritability of 0.3 for both traits; B) a narrow-sense heritability of 0.8 for both traits. MAF: minor allele frequencies. The false discovery rate rate was 0.05 and the window size was 10 kb for maize and 1 Mb for soybean.

Error rate with an FDR of 0.05 and window size of 1 Kb for maize and 10 kb for soybean

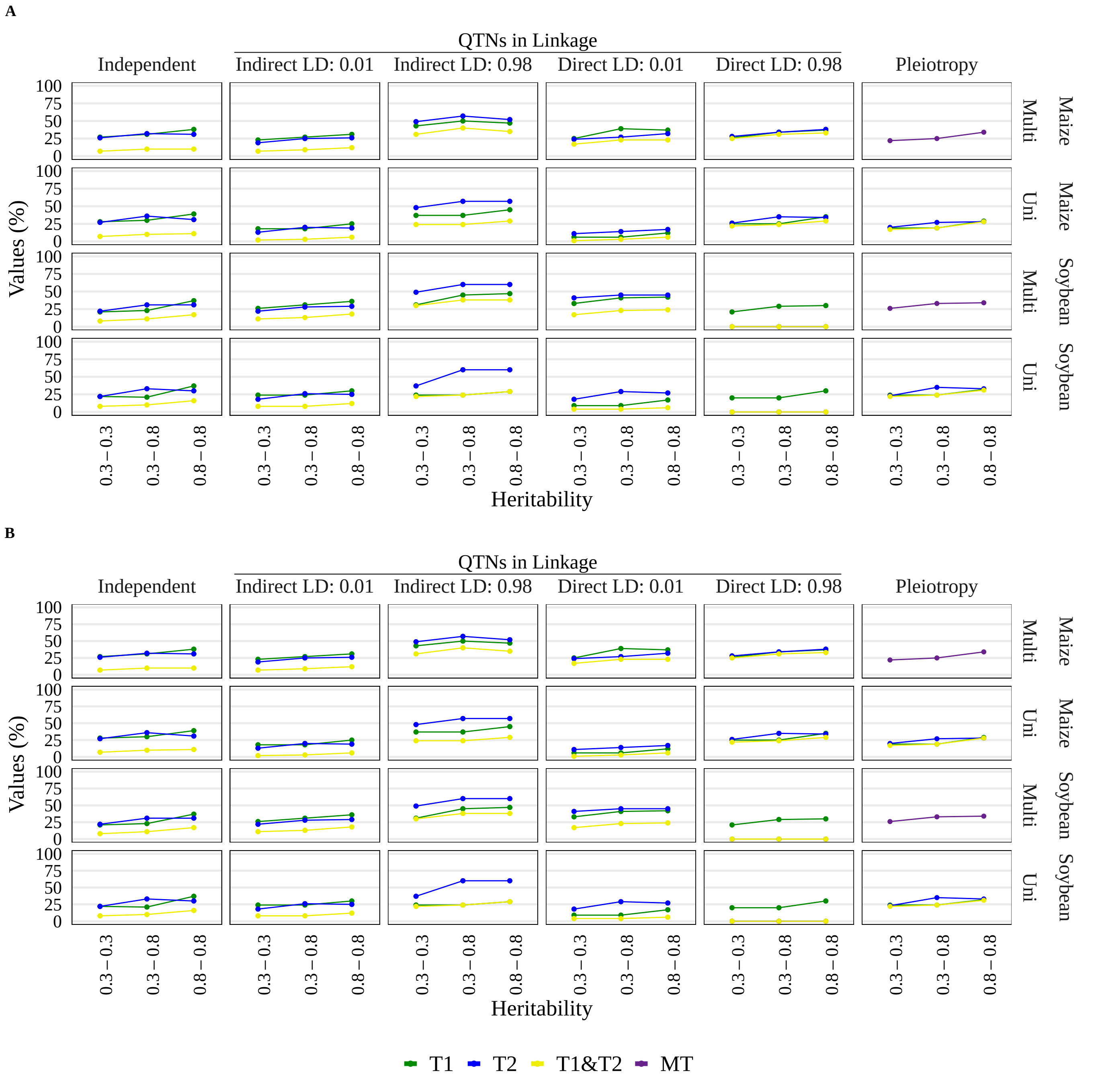

Figure S80: Error rate (measured as the detection of significant SNPs outside the window size) (Y-axis) achieved by multivariate (Multi) and univariate (Uni) GWAS, relative to the QTN controlling trait 1 (T1), trait 2 (T2), and both QTN simultaneously (T1&T2) or, in the pleiotropic scenario, relative to the pleiotropic QTN (MT). These values were obtained for maize and soybean with a sample size of 500. The X-axis displays the narrow-sense heritability for Trait 1 (bottom value) and Trait 2 (top value). A) inputted minor allele frequency (MAF) of 0.05; B) MAF of 0.4. The false discovery rate rate was 0.05 and the window size was 1 kb for maize and 10 Kb for soybean.

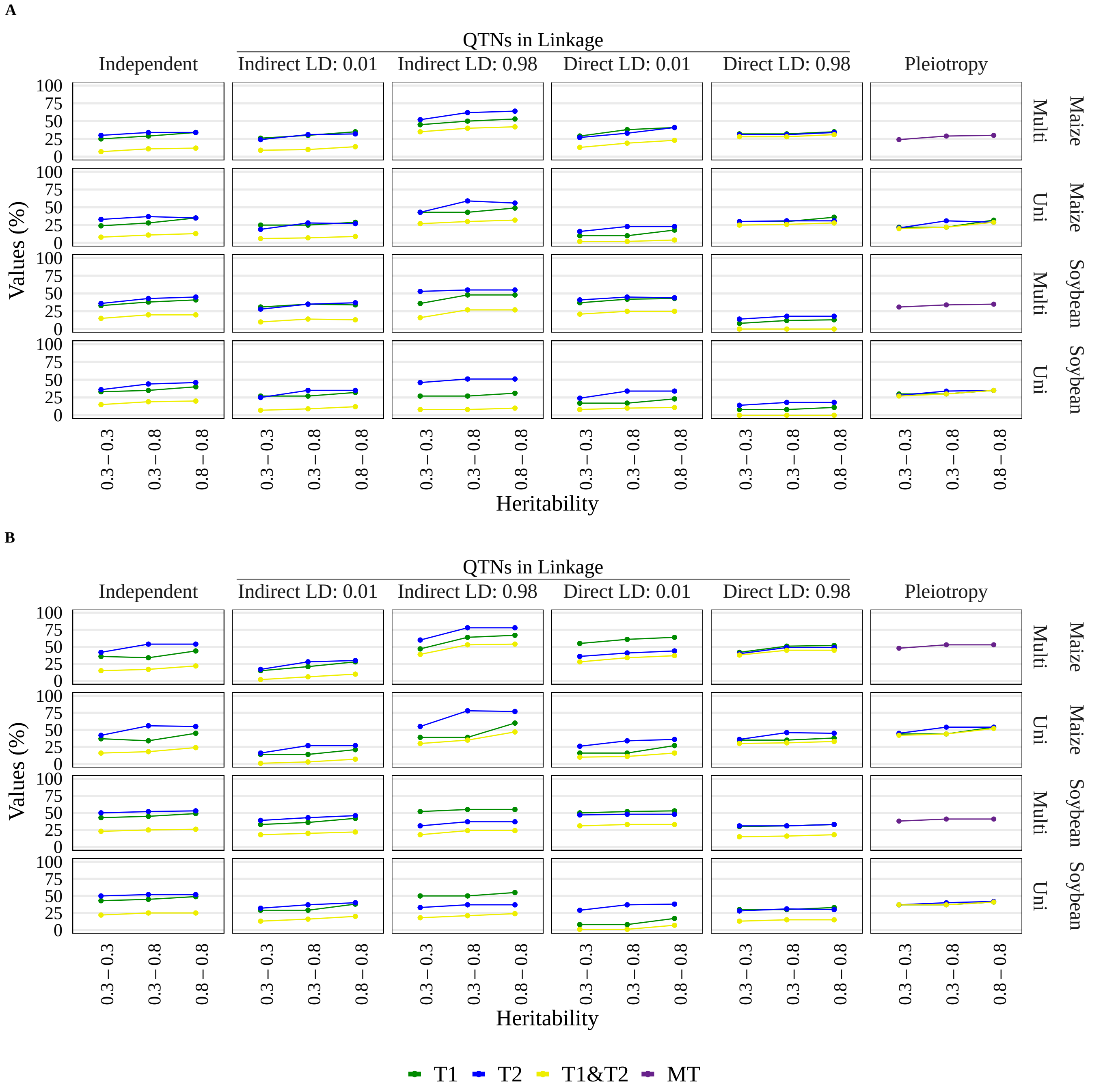

Figure S81: Error rate (measured as the detection of significant SNPs outside the window size) (Y-axis) achieved by multivariate (Multi) and univariate (Uni) GWAS, relative to the QTN controlling trait 1 (T1), trait 2 (T2), and both QTN simultaneously (T1&T2) or, in the pleiotropic scenario, relative to the pleiotropic QTN (MT). These values were obtained for maize and soybean with a sample size of $1,000$. The X-axis displays the narrow-sense heritability for Trait 1 (bottom value) and Trait 2 (top value). A) inputted minor allele frequency (MAF) of 0.05; B) MAF of 0.4. The false discovery rate rate was 0.05 and the window size was 1 kb for maize and 10 Kb for soybean.

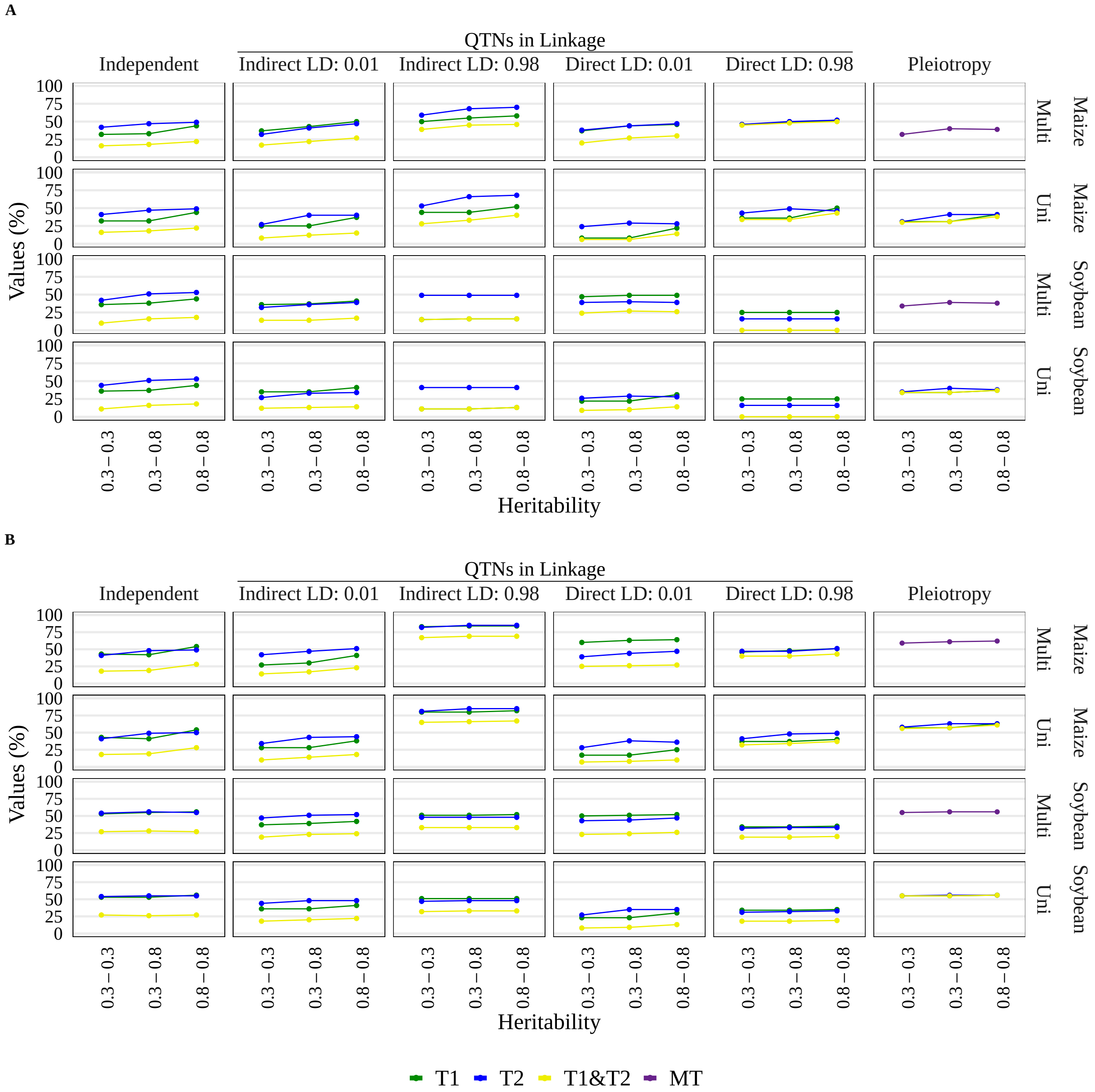

Figure S82: Error rate (measured as the detection of significant SNPs outside the window size) (Y-axis) achieved by multivariate (Multi) and univariate (Uni) GWAS, relative to the QTN controlling trait 1 (T1), trait 2 (T2), and both QTN simultaneously (T1&T2) or, in the pleiotropic scenario, relative to the pleiotropic QTN (MT). These values were obtained for maize and soybean with a sample size of $2,815$. The X-axis displays the narrow-sense heritability for Trait 1 (bottom value) and Trait 2 (top value). A) inputted minor allele frequency (MAF) of 0.05; B) MAF of 0.4. The false discovery rate rate was 0.05 and the window size was 1 kb for maize and 10 Kb for soybean.

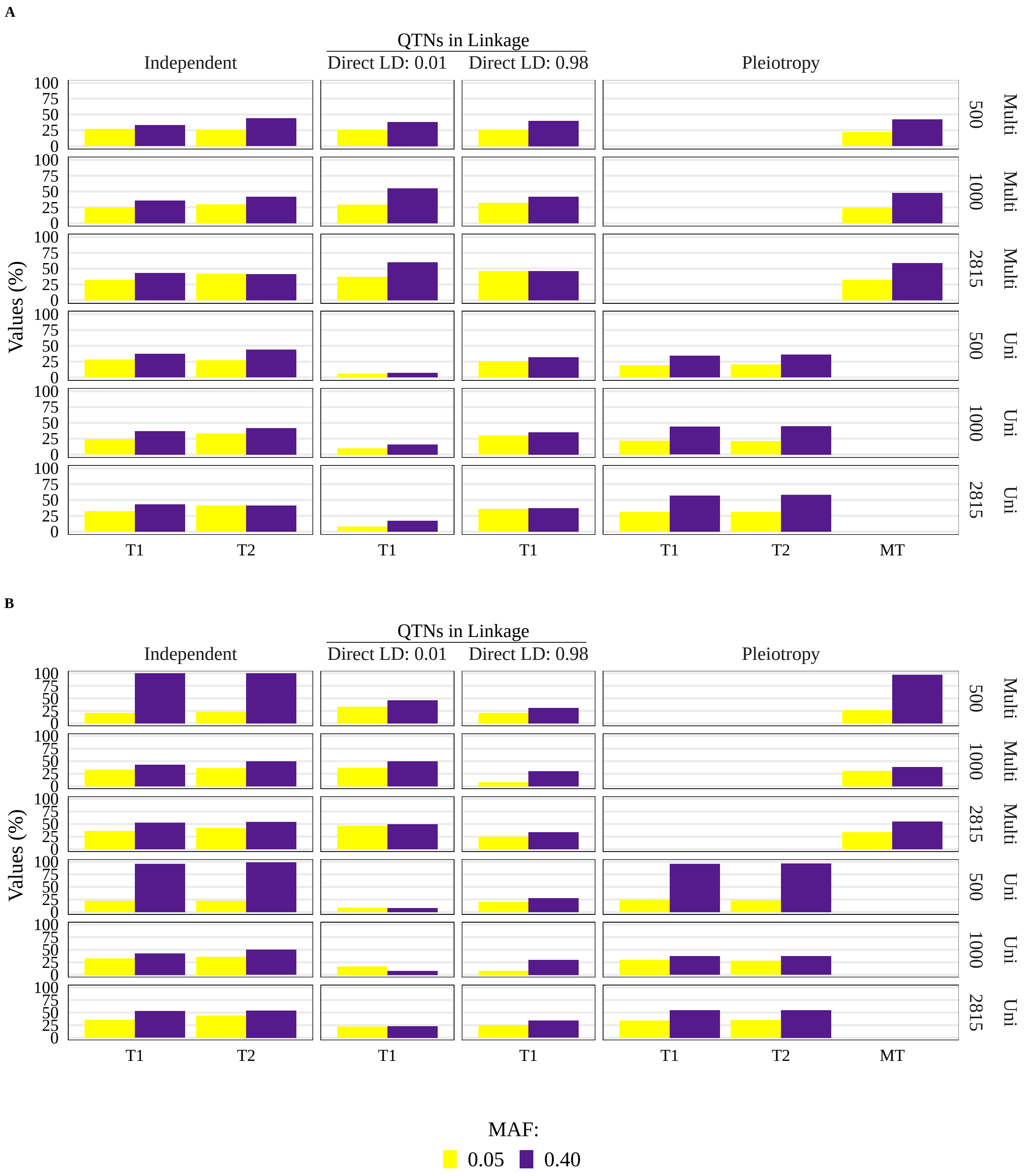

Figure S83: Error rate (measured as the detection of significant SNPs outside the window size) (Y-axis) in scenarios for which minor allele frequency (MAF) was directly controlled by a simulation input parameter. These values were obtained by multivariate (Multi) and univariate (Uni) GWAS, relative to the QTN controlling trait 1 (T1), trait 2 (T2) or, in the pleiotropic scenario, relative to the pleiotropic QTN (MT). This figure shows results for a narrow-sense heritability of 0.3 for both traits. A) Maize; B) Soybean. The false discovery rate rate was 0.05 and the window size was 1 kb for maize and 10 Kb for soybean.

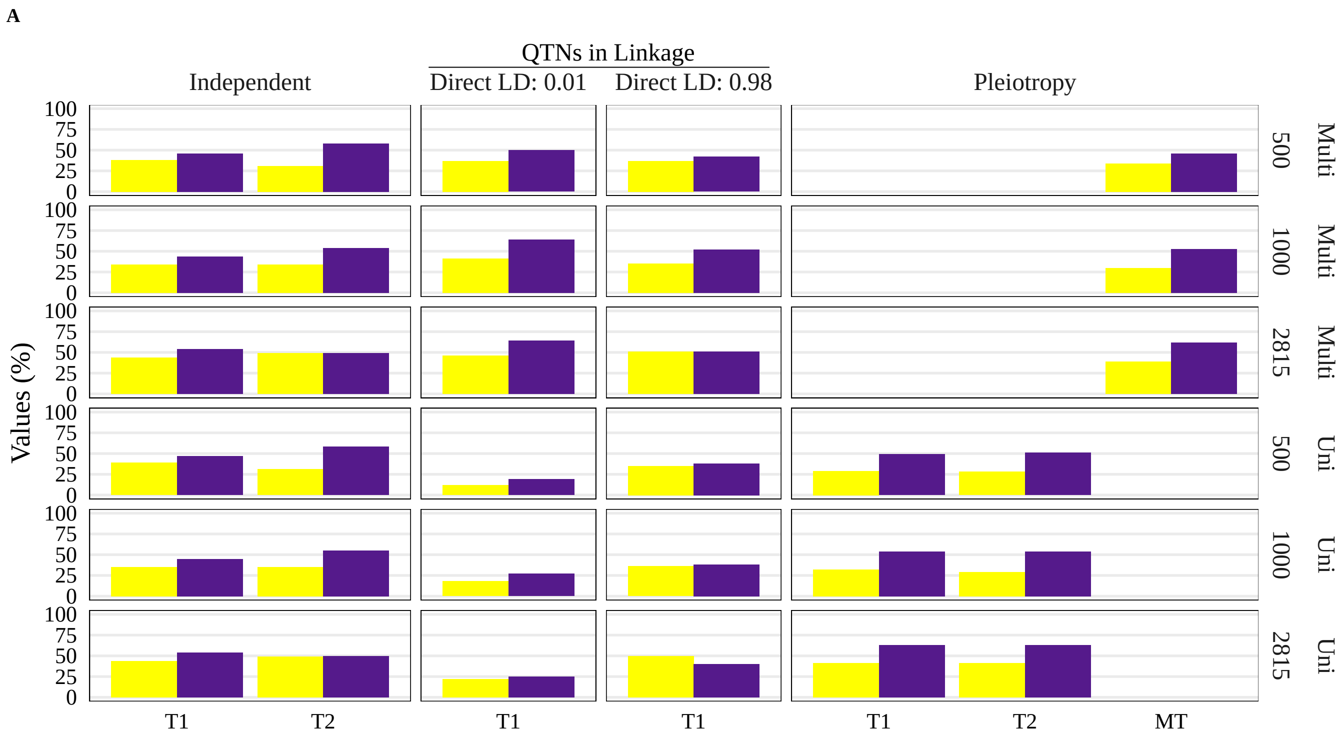

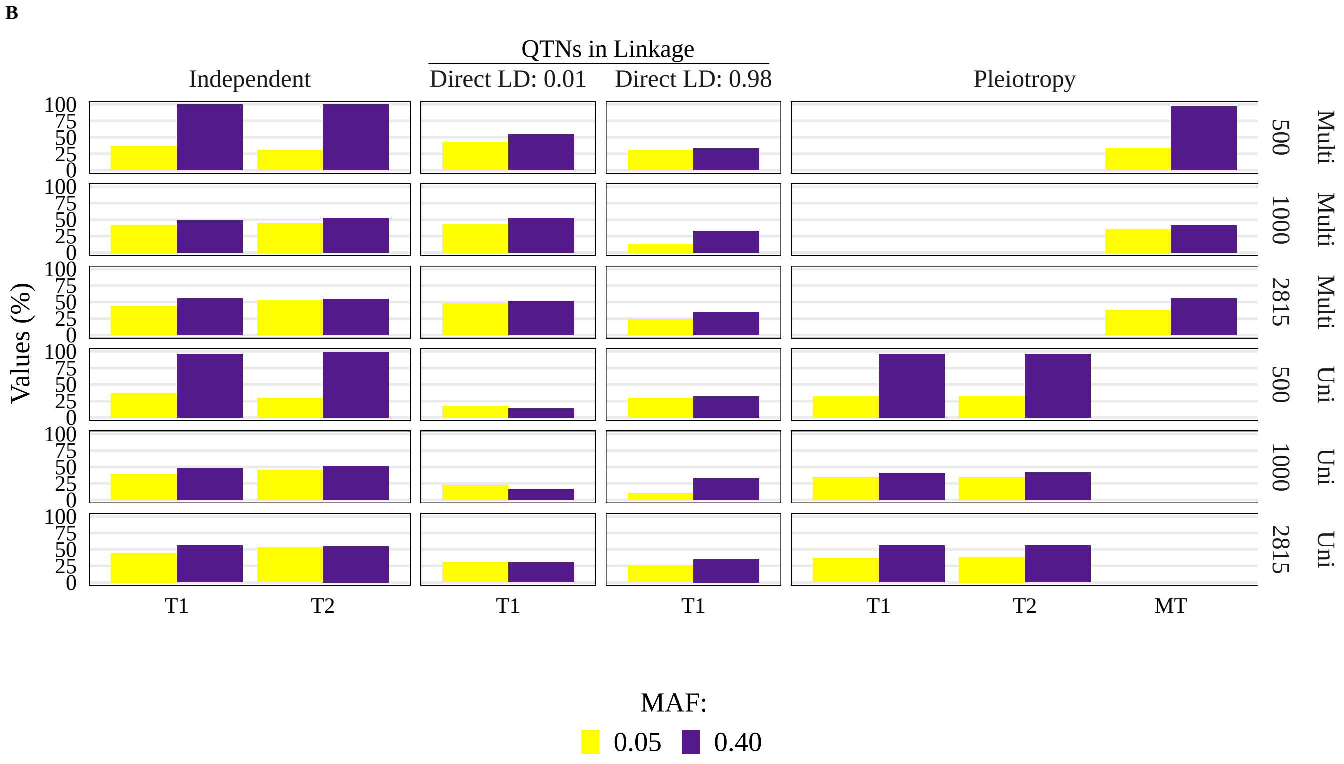

Figure S84: Error rate (measured as the detection of significant SNPs outside the window size) (Y-axis) in scenarios for which minor allele frequency (MAF) was directly controlled by a simulation input parameter. These values were obtained by multivariate (Multi) and univariate (Uni) GWAS, relative to the QTN controlling trait 1 (T1), trait 2 (T2) or, in the pleiotropic scenario, relative to the pleiotropic QTN (MT). This figure shows results for a narrow-sense heritability of 0.8 for both traits. A) Maize; B) Soybean. The false discovery rate rate was 0.05 and the window size was 1 kb for maize and 10 Kb for soybean.

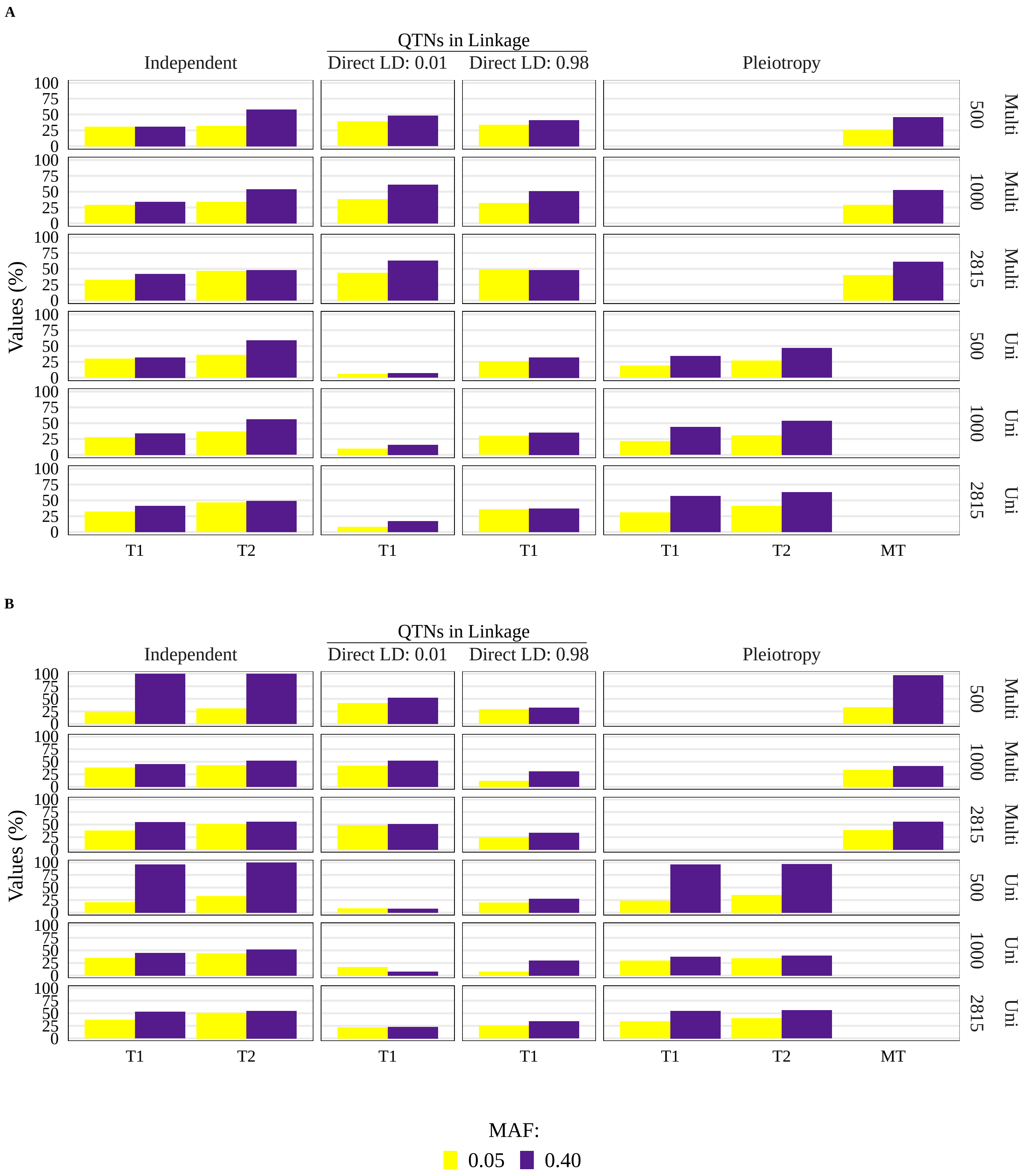

Figure S85: Error rate (measured as the detection of significant SNPs outside the window size) (Y-axis) in scenarios for which minor allele frequency (MAF) was directly controlled by a simulation input parameter. These values were obtained by multivariate (Multi) and univariate (Uni) GWAS, relative to the QTN controlling trait 1 (T1), trait 2 (T2) or, in the pleiotropic scenario, relative to the pleiotropic QTN (MT). This figure shows results for a narrow-sense heritability of 0.3 for trait 1 and 0.8 for trait 2. A) Maize; B) Soybean. The false discovery rate rate was 0.05 and the window size was 1 kb for maize and 10 Kb for soybean.

A

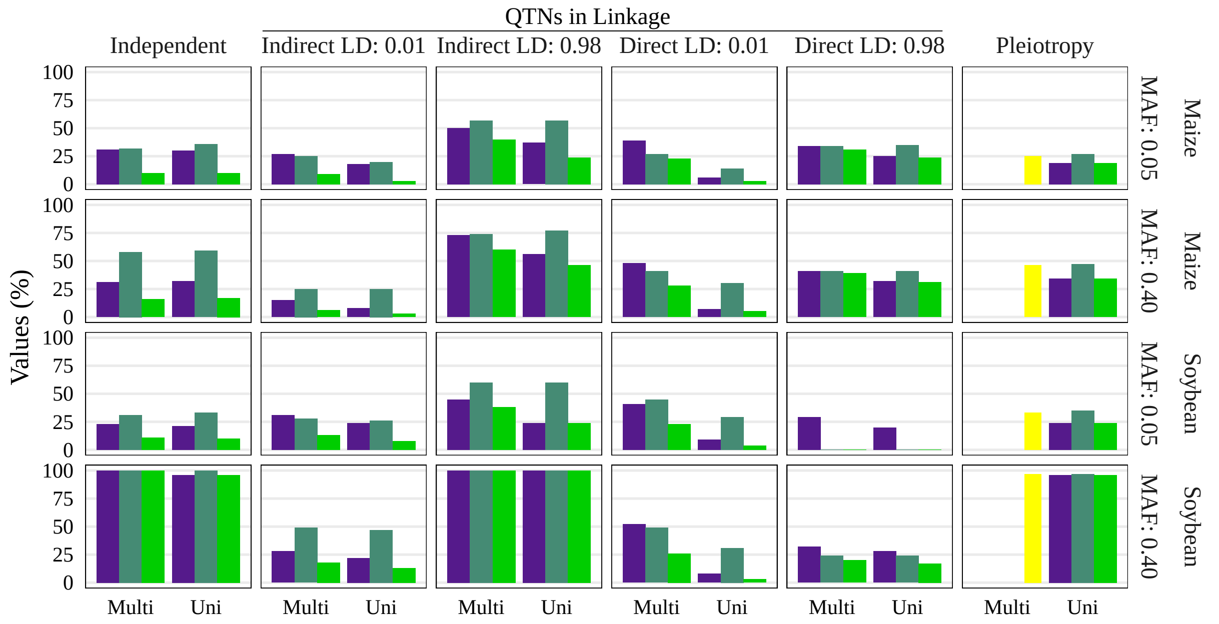

B

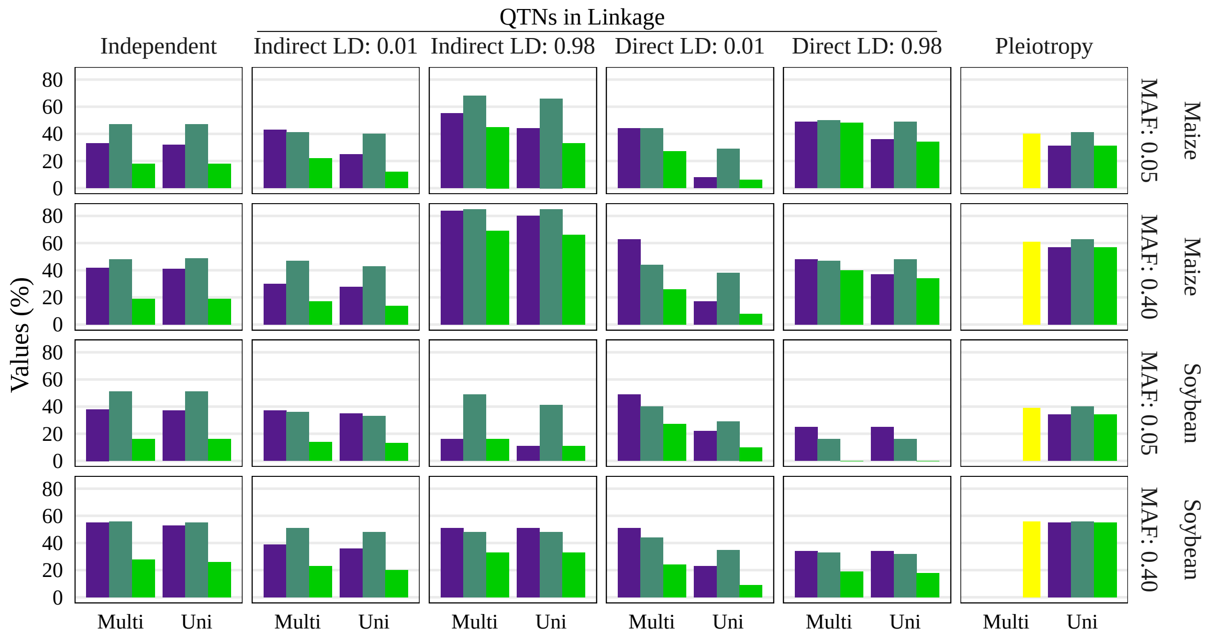

Figure S86: Error rate (measured as the detection of significant SNPs outside the window size) (Y-axis) achieved by multivariate (Multi) and univariate (Uni) GWAS (X-axis), relative to the QTN controlling trait 1 (T1), trait 2 (T2), and both QTN simultaneously (T1&T2) or, in the pleiotropic scenario, relative to the pleiotropic QTN (MT). The simulated genetic architecture is listed in the horizontal and vertical titles. These values were obtained with a narrow-sense heritability of 0.3 and 0.8 for traits 1 and 2, respectively. A) Sample size of 500; B) Sample size of 2815. MAF: minor allele frequencies. The false discovery rate rate was 0.05 and the window size was 1 kb for maize and 10 Kb for soybean.

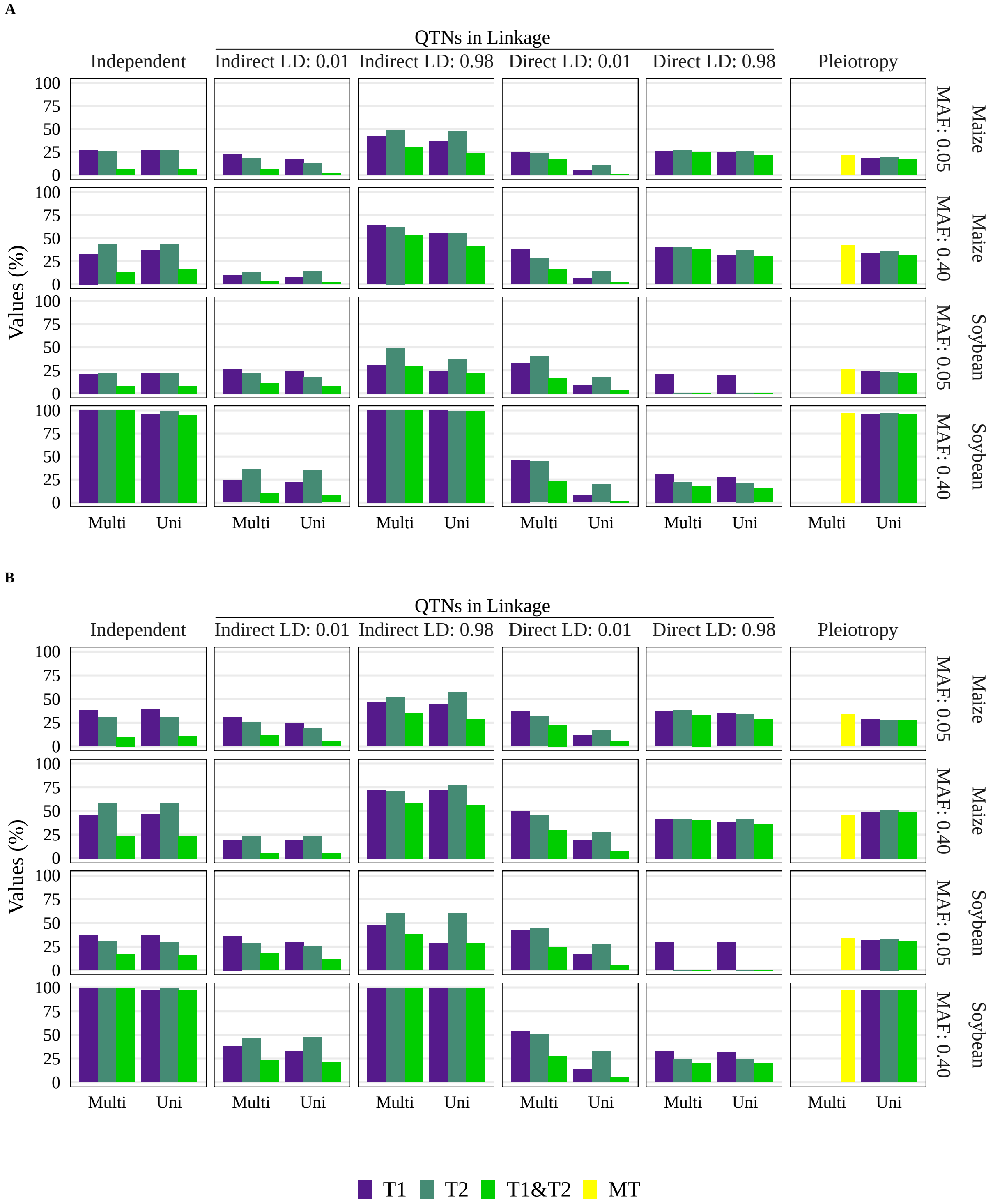

Figure S87: Error rate (measured as the detection of significant SNPs outside the window size) (Y-axis) achieved by multivariate (Multi) and univariate (Uni) GWAS (X-axis), relative to the QTN controlling trait 1 (T1), trait 2 (T2), and both QTN simultaneously (T1&T2) or, in the pleiotropic scenario, relative to the pleiotropic QTN (MT). The simulated genetic architecture is listed in the horizontal and vertical titles. These values were obtained with a sample size of 500; A) a narrow-sense heritability of 0.3 for both traits; B) a narrow-sense heritability of 0.8 for both traits. MAF: minor allele frequencies. The false discovery rate rate was 0.05 and the window size was 1 kb for maize and 10 Kb for soybean.

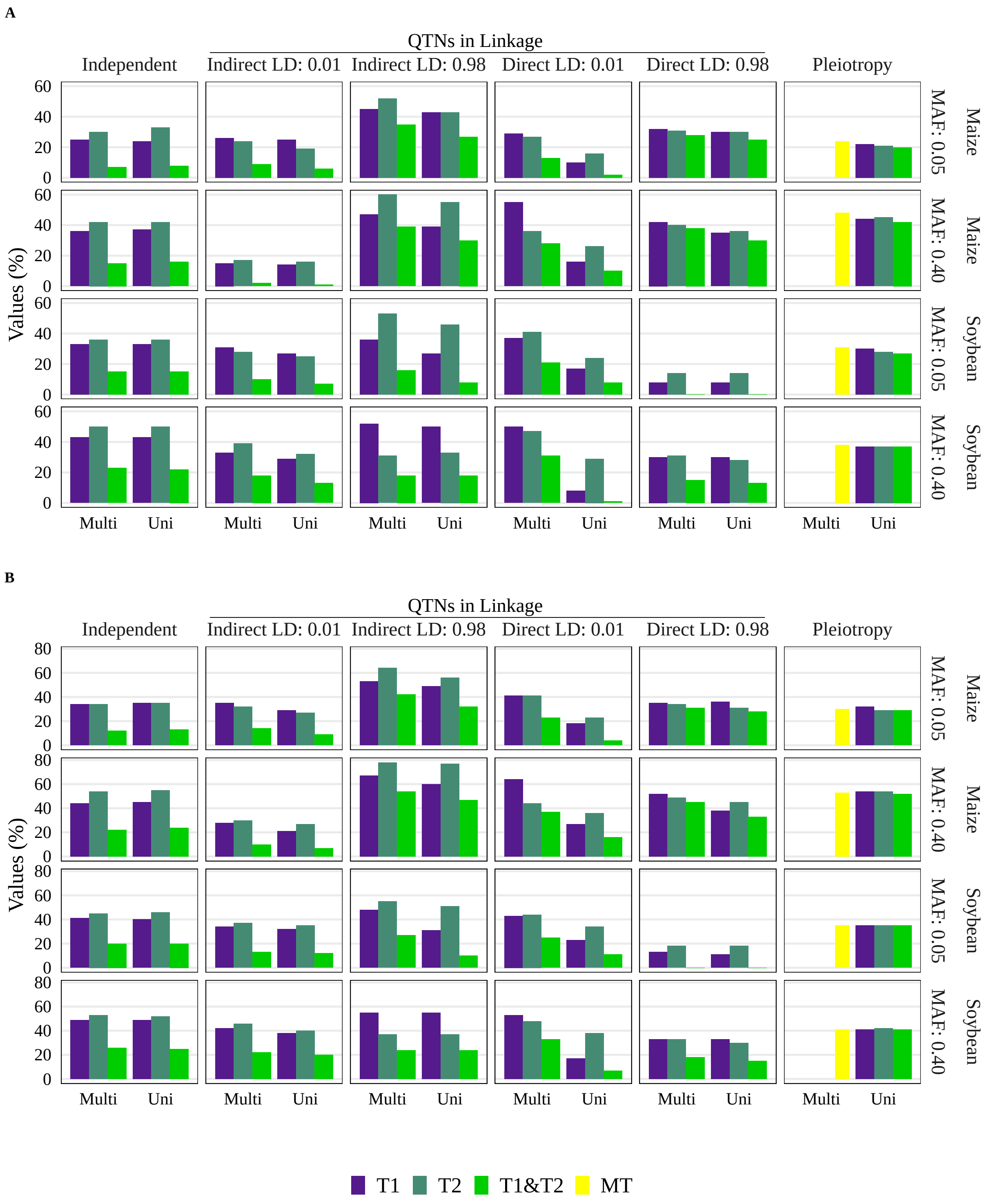

Figure S88: Error rate (measured as the detection of significant SNPs outside the window size) (Y-axis) achieved by multivariate (Multi) and univariate (Uni) GWAS (X-axis), relative to the QTN controlling trait 1 (T1), trait 2 (T2), and both QTN simultaneously (T1&T2) or, in the pleiotropic scenario, relative to the pleiotropic QTN (MT). The simulated genetic architecture is listed in the horizontal and vertical titles. These values were obtained with a sample size of 1, 000; A) a narrow-sense heritability of 0.3 for both traits; B) a narrow-sense heritability of 0.8 for both traits. MAF: minor allele frequencies. The false discovery rate rate was 0.05 and the window size was 1 kb for maize and 10 Kb for soybean.

A

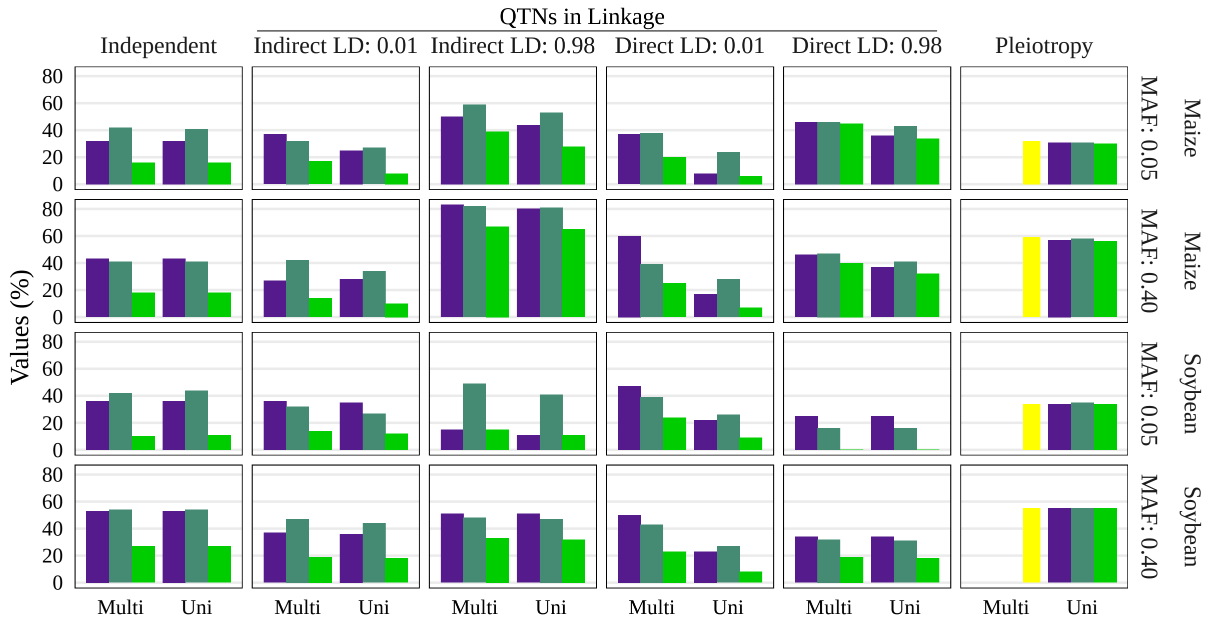

B

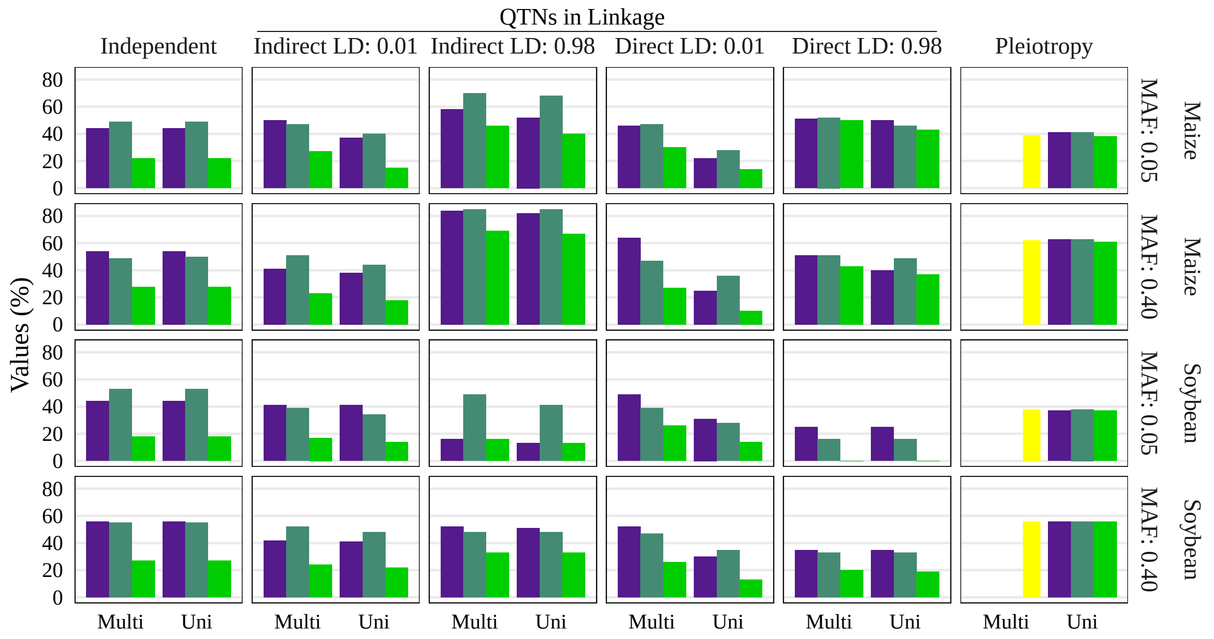

Figure S89: Error rate (measured as the detection of significant SNPs outside the window size) (Y-axis) achieved by multivariate (Multi) and univariate (Uni) GWAS (X-axis), relative to the QTN controlling trait 1 (T1), trait 2 (T2), and both QTN simultaneously (T1&T2) or, in the pleiotropic scenario, relative to the pleiotropic QTN (MT). The simulated genetic architecture is listed in the horizontal and vertical titles. These values were obtained with a sample size of 2, 815; A) a narrow-sense heritability of 0.3 for both traits; B) a narrow-sense heritability of 0.8 for both traits. MAF: minor allele frequencies. The false discovery rate rate was 0.05 and the window size was 1 kb for maize and 10 Kb for soybean.

Scripts

These R scripts can be found at: https://github.com/samuelbfernandes/fernandes_et_al_2020

Function to Simulate Phenotypes

Three functions were created to simulate each of the genetic architectures considered in this study: Independent traits (`run_simulation_ST`), Spurious Pleiotropy (`run_simulation_LD`), and Pleiotropy (`run_simulation_P`).

```
#------------- Independent traits --------------------
run_simulation_ST <-
  function(geno_file = NULL,
           list = NULL,
           out,
           h2) {
    pref <- gsub("../../|_mac.*", "", geno_file)
    new_dir <- paste0(out, pref, "_MAF", list$maf)
    dir.create(new_dir)
    qtn <- vector("list", nrow(h2))
    for (i in 1:nrow(h2)) {
      create_phenotypes(
        geno_file = geno_file,
        add_QTN_num = 1,
        h2 = h2[i, 1],
        add_effect = 0.1,
        rep = 100,
        seed = list$seed + as.numeric(gsub(".*_", "", pref)) + i,
        output_format = "multi-file",
        ntraits = 1,
        output_dir = paste0(new_dir, "/t1", i),
        model = "A",
        home_dir = getwd(),
        constraints = list(
          maf_above = list$maf - (list$maf * 0.1),
          maf_below = list$maf + (list$maf * 0.1)
        ),
        quiet = T,
        vary_QTN = T,
        verbose = F,
        out_geno = "plink"
      )
      create_phenotypes(
        geno_file = geno_file,
        add_QTN_num = 1,
        h2 = h2[i, 2],
        add_effect = 0.1,
        rep = 100,
```

```
      seed = round((list$seed + as.numeric(
        gsub(".*_", "", pref)
      )) / 2) + i,
      output_format = "multi-file",
      ntraits = 1,
      output_dir = paste0(new_dir, "/t2", i),
      model = "A",
      home_dir = getwd(),
      constraints = list(
        maf_above = list$maf - (list$maf * 0.1),
        maf_below = list$maf + (list$maf * 0.1)
      ),
      quiet = T,
      vary_QTN = T,
      verbose = F
    )
    qtn1 <-
      fread(paste0(new_dir, "/t1", i, "/Additive_selected_QTNs.txt"),
            data.table = F)
    qtn1$snp_type <- "QTN_for_trait_1"
    qtn1$h2 <- paste0(i, "_", h2[i, 1])
    qtn2 <-
      fread(paste0(new_dir, "/t2", i, "/Additive_selected_QTNs.txt"),
            data.table = F)
    qtn2$snp_type <- "QTN_for_trait_2"
    qtn2$h2 <-  paste0(i, "_", h2[i, 2])
    qtn[[i]] <- rbind(qtn1, qtn2)
  }
  qtn <- data.table::rbindlist(qtn)
  qtn <-
    qtn[order(qtn$rep),
        c("rep", "snp_type", "snp", "allele", "chr", "pos", "cm", "maf", "h2")]
  file.rename(paste0(new_dir, "/t11/", gsub(".txt", ".bed", geno_file)),
              paste0(new_dir, "/", gsub(".txt", ".bed", geno_file)))
  file.rename(paste0(new_dir, "/t11/", gsub(".txt", ".bim", geno_file)),
              paste0(new_dir, "/", gsub(".txt", ".bim", geno_file)))
  fwrite(
    qtn,
    file = paste0(new_dir, "/Additive_selected_QTNs.txt"),
    quote = F,
    sep = "\t",
    row.names = F
  )
  for (i in seq_len(100)) {
    t1 <-
      fread(
        paste0(
```

```
        new_dir,
        "/t11/Simulated_Data_Rep",
        i,
        "_Herit_",
        h2[1, 1],
        ".txt"
      ),
      data.table = F
    )
  t2 <-
    fread(
      paste0(
        new_dir,
        "/t21/Simulated_Data_Rep",
        i,
        "_Herit_",
        h2[1, 2],
        ".txt"
      ),
      data.table = F
    )
  fam1 <- data.frame(t1[, 1], t1[, 1], 0, 0, 0, t1[, 2])
  colnames(fam1) <- paste0("V", 1:6)
  fam1 <- merge(fam1,
                t2,
                by.x = "V1",
                by.y = "<Trait>",
                sort = F)
  colnames(fam1)[7] <- "V7"
  fwrite(
    fam1,
    file = paste0(
      new_dir,
      "/",
      "Simulated_Data__Rep",
      i,
      "_Herit_",
      h2[1, 1],
      "_",
      h2[1, 2],
      ".fam"
    ),
    quote = F,
    sep = "\t",
    row.names = F,
    col.names = F
  )
```

```
    t3 <-
      fread(
        paste0(
          new_dir,
          "/t12/Simulated_Data_Rep",
          i,
          "_Herit_",
          h2[2, 1],
          ".txt"
        ),
        data.table = F
      )
    t4 <-
      fread(
        paste0(
          new_dir,
          "/t22/Simulated_Data_Rep",
          i,
          "_Herit_",
          h2[2, 2],
          ".txt"
        ),
        data.table = F
      )
    fam2 <- data.frame(t3[, 1], t3[, 1], 0, 0, 0, t3[, 2])
    colnames(fam2) <- paste0("V", 1:6)
    fam2 <- merge(fam2,
                  t4,
                  by.x = "V1",
                  by.y = "<Trait>",
                  sort = F)
    colnames(fam2)[7] <- "V7"
    fwrite(
      fam2,
      file = paste0(
        new_dir,
        "/",
        "Simulated_Data__Rep",
        i,
        "_Herit_",
        h2[1, 2],
        "_",
        h2[2, 2],
        ".fam"
      ),
      quote = F,
      sep = "\t",
```

```
    row.names = F,
    col.names = F
  )
  t5 <-
    fread(
      paste0(
        new_dir,
        "/t13/Simulated_Data_Rep",
        i,
        "_Herit_",
        h2[3, 1],
        ".txt"
      ),
      data.table = F
    )
  t6 <-
    fread(
      paste0(
        new_dir,
        "/t23/Simulated_Data_Rep",
        i,
        "_Herit_",
        h2[3, 2],
        ".txt"
      ),
      data.table = F
    )
  fam3 <- data.frame(t5[, 1], t5[, 1], 0, 0, 0, t5[, 2])
  colnames(fam3) <- paste0("V", 1:6)
  fam3 <- merge(fam3,
                t6,
                by.x = "V1",
                by.y = "<Trait>",
                sort = F)
  colnames(fam3)[7] <- "V7"
  fwrite(
    fam3,
    file = paste0(
      new_dir,
      "/",
      "Simulated_Data__Rep",
      i,
      "_Herit_",
      h2[3, 1],
      "_",
      h2[3, 2],
      ".fam"
```

```
        ),
        quote = F,
        sep = "\t",
        row.names = F,
        col.names = F
      )
    }
    unlink(
      c(
        paste0(new_dir, "/t11"),
        paste0(new_dir, "/t12"),
        paste0(new_dir, "/t21"),
        paste0(new_dir, "/t22"),
        paste0(new_dir, "/t13"),
        paste0(new_dir, "/t23")
      ),
      recursive = T,
      force = T
    )
  }

#---------  Spurious Pleiotropy ------------------
run_simulation_LD <-
  function(geno_file = NULL,
           list = NULL,
           out,
           h2,
           ld_type) {
    pref <- gsub("../../|_mac.*", "", geno_file)
    create_phenotypes(
      geno_file = geno_file,
      add_QTN_num = 1,
      h2 = h2,
      add_effect = c(0.1, 0.1),
      rep = 100,
      seed = list$seed + as.numeric(gsub(".*_", "", pref)),
      output_format = "gemma",
      architecture = "LD",
      output_dir =
        paste0(out, pref, "_ld_", list$ld, "_MAF", list$maf, "_", ld_type),
      ld = list$ld,
      model = "A",
      home_dir = getwd(),
      constraints = list(
        maf_above = list$maf - (list$maf * 0.1),
        maf_below = list$maf + (list$maf * 0.1)
      ),
```

```r
      type_of_ld = ld_type,
      quiet = T,
      remove_QTN = TRUE,
      vary_QTN = TRUE,
      warning_file_saver = FALSE,
      verbose = F
    )
  }

#----------------  Pleiotropy  ------------------------
run_simulation_P <-
  function(geno_file = NULL,
           list = NULL,
           out,
           h2) {
    pref <- gsub("../../|_mac.*", "", geno_file)
    create_phenotypes(
      geno_file = geno_file,
      add_QTN_num = 1,
      h2 = h2,
      add_effect = c(0.1, 0.1),
      rep = 100,
      seed = list$seed + as.numeric(gsub(".*_", "", pref)),
      output_format = "gemma",
      architecture = "pleiotropic",
      ntraits = 2,
      output_dir = paste0(out, pref, "_MAF", list$maf, collapse = "_"),
      model = "A",
      home_dir = getwd(),
      constraints = list(
        maf_above = list$maf - (list$maf * 0.1),
        maf_below = list$maf + (list$maf * 0.1)
      ),
      quiet = T,
      remove_QTN = TRUE,
      vary_QTN = TRUE,
      warning_file_saver = FALSE
    )
  }
```

Function to run GEMMA

A function that calls GEMMA (`gemma`) and two other functions that run it were created to analyze Pleiotropy and Spurious Pleiotropy (`run_gemma`), and Independent traits (`run_gemma_ST`).

```
gemma <- function(gemma_path  = "PATH/TO/GEMMA",
                  path_out = getwd(),
                  geno = NULL,
                  d = NULL,
                  u = NULL,
                  lmm = 2,
                  miss = 0.05,
                  maf = 0.01,
                  r2 = 0.9999,
                  trait_col = 1,
                  out_name = NULL,
                  verbose = FALSE) {
  home_path <- getwd()
  setwd(path_out)
  system(
    command = paste(
      gemma_path,
      "--bfile",
      geno,
      "-lmm",
      lmm,
      "-miss",
      miss,
      "-maf",
      maf,
      "-r2",
      r2,
      "-n",
      paste(trait_col, collapse = " "),
      "-d",
      d,
      "-u",
      u,
      "-o",
      out_name
    ),
    ignore.stdout = !verbose,
    ignore.stderr = !verbose
  )
  setwd(home_path)
}
#---------- Runs GEMMA in Pleiotropic and Spurious Pleiotropy traits --------
run_gemma <-
```

```r
function(path = getwd(),
         pop,
         n_cores = parallel::detectCores()) {
  files <- dir(path)
  files_fam <- files[grepl("Simulated_Data_", files)]
  files_bed <- files[grepl(".bed|.bim", files)]
  dir.create(paste0(path, "gemma"))
  n_files <- length(files_fam)
  files_fam <- split(files_fam, 1:n_files)
  invisible(mclapply(files_fam, function(x) {
    genoname <- gsub(".fam", "", x)
    bfiles <-
      files_bed[gsub("Simulated_Data__Rep|_Herit.*", "", x) ==
                  gsub(".*rep_|.bim|.bed", "", files_bed)]
    #to avoid two copies of the same file at the same time
    prefix <- round(runif(1, min = 1, max = 10000))
    file.rename(paste0(path, x), paste0(path, "gemma/", x))
    file.copy(paste0(path, bfiles[1]), paste0(path, "B", prefix, bfiles[1]))
    file.copy(paste0(path, bfiles[2]), paste0(path, "B", prefix, bfiles[2]))
    file.rename(paste0(path, "B", prefix, bfiles[grepl(".bed", bfiles)]),
                paste0(path, "gemma/", genoname, ".bed"))
    file.rename(paste0(path, "B", prefix, bfiles[grepl(".bim", bfiles)]),
                paste0(path, "gemma/", genoname, ".bim"))
    gemma(
      geno = genoname,
      d = paste0(
        "../../../../kin_",
        gsub("_|_mac5.*", "", pop),
        ".eigenD.txt"
      ),
      u = paste0(
        "../../../../kin_",
        gsub("_|_mac5.*", "", pop),
        ".eigenU.txt"
      ),
      trait_col = c(1, 2),
      miss = 0.999,
      maf = 0.0001,
      r2 = 0.9999999,
      out_name = gsub("Simulated_Data__", "MT", genoname),
      path_out = paste0(path, "gemma")
    )
    gemma(
      geno = genoname,
      d = paste0(
        "../../../../kin_",
        gsub("_|_mac5.*", "", pop),
```

```
        ".eigenD.txt"
      ),
      u = paste0(
        "../../../../kin_",
        gsub("_|_mac5.*", "", pop),
        ".eigenU.txt"
      ),
      trait_col = 1,
      miss = 0.001,
      maf = 0.001,
      r2 = 0.999999,
      out_name = gsub("Simulated_Data__", "T1_", genoname),
      path_out = paste0(path, "gemma")
    )
    gemma(
      geno = genoname,
      d = paste0(
        "../../../../kin_",
        gsub("_|_mac5.*", "", pop),
        ".eigenD.txt"
      ),
      u = paste0(
        "../../../../kin_",
        gsub("_|_mac5.*", "", pop),
        ".eigenU.txt"
      ),
      trait_col = 2,
      miss = 0.001,
      maf = 0.001,
      r2 = 0.999999,
      out_name = gsub("Simulated_Data__", "T2_", genoname),
      path_out = paste0(path, "gemma")
    )
    unlink(c(
      paste0(path, "gemma/", genoname, ".bed"),
      paste0(path, "gemma/", genoname, ".bim"),
      paste0(path, "gemma/", genoname, ".fam")
    ),
    recursive = T,
    force = T)
  },
  mc.preschedule = FALSE,
  mc.cores = n_cores))
  unlink(paste0(path, files[grepl(".bed|.bim|.fam", files)]),
         recursive = T,
         force = T)
}
```

```r
#---- Runs GEMMA on two independent traits -------
run_gemma_ST <-
  function(path = getwd(),
           pop,
           n_cores = parallel::detectCores()) {
    files <- dir(path)
    files_fam <- files[grepl("Simulated_Data_", files)]
    bfiles <- files[grepl(".bed|.bim", files)]
    dir.create(paste0(path, "gemma"))
    n_files <- length(files_fam)
    files_fam <- split(files_fam, 1:n_files)
    invisible(mclapply(files_fam, function(x) {
      genoname <- gsub(".fam", "", x)
      #to avoid two copies of the same file at the same time:
      prefix <- round(runif(1, min = 1, max = 10000))
      file.rename(paste0(path, x), paste0(path, "gemma/", x))
      file.copy(paste0(path, bfiles[1]), paste0(path, "B", prefix, bfiles[1]))
      file.copy(paste0(path, bfiles[2]), paste0(path, "B", prefix, bfiles[2]))
      file.rename(paste0(path, "B", prefix, bfiles[grepl(".bed", bfiles)]),
                  paste0(path, "gemma/", genoname, ".bed"))
      file.rename(paste0(path, "B", prefix, bfiles[grepl(".bim", bfiles)]),
                  paste0(path, "gemma/", genoname, ".bim"))
      gemma(
        geno = genoname,
        d = paste0(
          "../../../../kin_",
          gsub("_|_mac5.*", "", pop),
          ".eigenD.txt"
        ),
        u = paste0(
          "../../../../kin_",
          gsub("_|_mac5.*", "", pop),
          ".eigenU.txt"
        ),
        trait_col = c(1, 2),
        miss = 0.999,
        maf = 0.0001,
        r2 = 0.9999999,
        out_name = gsub("Simulated_Data__", "MT", genoname),
        path_out = paste0(path, "gemma")
      )
      gemma(
        geno = genoname,
        d = paste0(
          "../../../../kin_",
          gsub("_|_mac5.*", "", pop),
          ".eigenD.txt"
```

```
      ),
      u = paste0(
        "../../../../kin_",
        gsub("_|_mac5.*", "", pop),
        ".eigenU.txt"
      ),
      trait_col = c(1),
      miss = 0.999,
      maf = 0.0001,
      r2 = 0.9999999,
      out_name = gsub("Simulated_Data__", "T1", genoname),
      path_out = paste0(path, "gemma")
    )
    gemma(
      geno = genoname,
      d = paste0(
        "../../../../kin_",
        gsub("_|_mac5.*", "", pop),
        ".eigenD.txt"
      ),
      u = paste0(
        "../../../../kin_",
        gsub("_|_mac5.*", "", pop),
        ".eigenU.txt"
      ),
      trait_col = c(2),
      miss = 0.999,
      maf = 0.0001,
      r2 = 0.9999999,
      out_name = gsub("Simulated_Data__", "T2", genoname),
      path_out = paste0(path, "gemma")
    )
    unlink(c(
      paste0(path, "gemma/", genoname, ".bed"),
      paste0(path, "gemma/", genoname, ".bim"),
      paste0(path, "gemma/", genoname, ".fam")
    ),
    recursive = T,
    force = T)
  },
  mc.preschedule = FALSE,
  mc.cores = n_cores))
  unlink(paste0(path, files[grepl(".bed|.bim|.fam", files)]),
         recursive = T,
         force = T)
}
```

Pipeline to simulate and analyze a pair of traits under the three Pleiotropy, Spurious Pleiotropy, and Independent traits. Notice that the seed numbers used for `settings[[21]]` and `settings[[24]]` generated at least one replicate pair of traits for which GEMMA resulted in an error due to highly colinear traits. Therefore, we simulated pairs of traits using a different seed number.

```
#------- Independent traits ------------
home <- "/HOME/DIRECTORY"
setwd(home)
library(simplePHENOTYPES)
library(parallel)
library(data.table)
library(tidyverse)
source("./scripts/run_simulation_ST.R")
source("./scripts/run_gemma_ST.R")
MAF <-  c(0.05, 0.4)
h2 <- matrix(c(0.3, 0.3, 0.8, 0.3, 0.8, 0.8), 3)
custom_a <- list(list(trait_1 = c(0.1),
                      trait_2 = c(0.1)))
data <- c("ames_500_mac5_ld09_numeric.txt",
          "ames_1000_mac5_ld09_numeric.txt",
          "ames_2815_mac5_ld09_numeric.txt",
          "soy_500_mac5_ld09_numeric.txt",
          "soy_1000_mac5_ld09_numeric.txt",
          "soy_2815_mac5_ld09_numeric.txt")
#seed = round(runif(12, 0, 2000000))
seed = c(
  531017,
  744248,
  1145707,
  1816416,
  403364,
  1796779,
  1889351,
  1321596,
  1258228,
  123573,
  411949,
  353114
)
grid <-
  expand.grid(
    maf = MAF,
    effect = custom_a,
    data = data,
    stringsAsFactors = F
  )
grid <- grid[order(grid$data,  grid$maf), ]
settings <-  data.frame(i = 1:12, seed = seed, grid)
```

```r
settings <- split(settings, settings$i)
out <- "./simulation/ST/"
for (x in settings) {
  pop <- x$data
  run_simulation_ST(
    geno_file = pop,
    list = x,
    out = out,
    h2 = h2
  )
  path  <-
    paste0(out, gsub("_mac5.*", "", pop), "_MAF", x$maf, "/")
  cat(paste("GEMMA: Setting", x$i, "\n"))
  run_gemma_ST(path = path,
               pop = pop,
               n_cores = detectCores())
}
# -------   correlation  ------------
setwd(out)
x <- dir()
a <- 1
correl <- vector("list", 12)
for (i in x){
  temp <- fread(paste0("./", i, "/correlation.txt"), data.table = F)
  colnames(temp) <- c("0.3_0.3", "0.3_0.8", "0.8_0.8")
  correl[[a]] <-  tibble(
    specie = gsub("_.*","", i),
    sample_size = gsub(".*_", "", gsub("_MAF.*","", i)),
    ld = "",
    maf = gsub(".*MAF|_.*","", i),
    type = "ST",
    pivot_longer(
      temp,
      names_to = "h2",
      values_to = "cor",
      cols = `0.3_0.3`:`0.8_0.8`
    ) )
  print(a)
  a <- a + 1
}
correl <- rbindlist(correl)
correl <- correl %>% select(specie, h2, sample_size,    ld, maf,    type,   cor)
fwrite(correl, "correlation_ST.txt", sep = "\t", quote = F, row.names = F)

#------------Spurious Pleiotropy---------------
setwd(home)
source("./scripts/run_simulation_LD.R")
```

```r
source("./scripts/run_gemma.R")
MAF <-  c(0.05, 0.4)
h2 <- matrix(c(0.3, 0.3, 0.8, 0.3, 0.8, 0.8), 3)
ld <- c(0.1, 0.9)
ld_type <- c("indirect", "direct")
data <- c(
  "ames_500_mac5_ld09_numeric.txt",
  "ames_1000_mac5_ld09_numeric.txt",
  "ames_2815_mac5_ld09_numeric.txt",
  "soy_500_mac5_ld09_numeric.txt",
  "soy_1000_mac5_ld09_numeric.txt",
  "soy_2815_mac5_ld09_numeric.txt"
)
#seed <- round(runif(48, 0, 2000000))
seed <- c(937099, 615532, 967541, 515345, 1624805, 1104645, 740641, 112766,
          560708, 1093117, 796976, 340524, 1525102, 1249993, 1338043, 1764331,
          250478, 409224, 1421608, 715050, 1076697, 718950, 54605, 1380581,
          1098193, 840203, 555448, 342840, 976612, 1540603, 1857010, 1763907,
          360814, 697384, 1258782, 1908315, 1979128, 1390548, 260578, 35,
          1206649, 661321, 982464, 1730241, 1560717, 1555169, 1768454, 1654607)
grid <-
  expand.grid(
    ld = ld,
    maf = MAF,
    ld_type = ld_type,
    data = data,
    stringsAsFactors = F
  )
grid <-
  grid[order(grid$data, rev(grid$ld_type), grid$ld, grid$maf), ]
settings <-  data.frame(i = 1:48, seed = seed, grid)
settings <- split(settings, settings$i)
out <- "./simulation/SP/"
for (x in settings) {
  cat(paste("Setting", x$i, "\n"))
  run_simulation_LD(
    geno_file = x$data,
    list = x,
    out = out,
    h2 = h2,
    ld_type = x$ld_type
  )
  path  <-
    paste0(out,
           gsub("_mac5.*", "", x$data),
           "_ld_",
           x$ld,
```

```
           "_MAF",
           x$maf,
           "_",
           x$ld_type,
           "/")
}
settings[[21]]$seed  <- 15273
cat(paste("Setting", settings[[21]]$i, "\n"))
run_simulation_P(
  geno_file = settings[[21]]$data,
  list = settings[[21]],
  out = out,
  h2 = h2
)
path  <-
  paste0(out, gsub("_mac5.*", "", settings[[21]]$data), "_MAF", settings[[21]]$maf, "/")
cat(paste("GEMMA: Setting", settings[[21]]$i, "\n"))
run_gemma_p(path = path,
            pop = settings[[21]]$data,
            n_cores = detectCores())
setwd("./simulation/")
x <- dir("./ames_500_ld_0.1_MAF0.05_direct/gemma/output")
reps <-
  names(
    table(gsub(".*Rep|_He.*", "", x))[table(gsub(".*Rep|_He.*", "", x)) < 18]
    )
unlink(
  paste0("./ames_500_ld_0.1_MAF0.05_direct/gemma/output/",
         x[gsub(".*Rep|_He.*", "", x) %in% reps]),
  force = T,
  recursive = T
)
x <- dir("./ames_500_ld_0.1_MAF0.05_direct/gemma/output")
total <- 1:100
missing <-
  total[!total %in% names(table(gsub(".*Rep|_He.*", "", x)))]
QTN <-
  read.table("./ames_500_ld_0.1_MAF0.05_direct/Additive_selected_QTNs.txt",
             h = T)
QTN <- QTN[!QTN$rep %in% missing,]
x2 <- dir("./ames_500_ld_0.1_MAF0.05_direct2/gemma/output/")
replace <-
  names(table(gsub(".*Rep|_He.*", "", x2))[
    table(gsub(".*Rep|_He.*", "", x2)) == 18])[1:length(missing)]
QTN2 <-
  read.table("./ames_500_ld_0.1_MAF0.05_direct2/Additive_selected_QTNs.txt",h=T)
QTN2 <- QTN2[QTN2$rep %in% replace,]
```

```r
QTN2$rep <- missing
for (i in seq_along(replace)) {
  files <- x2[grepl(paste0("Rep", replace[i], "_"), x2)]
  for (j in 1:18) {
    new_name <-
      paste0(gsub("Rep.*", "", files[j]),
             "Rep",
             missing[i],
             "_H",
             gsub(".*_H", "", files[j]))
    file.rename(
      paste0(
        "./ames_500_ld_0.1_MAF0.05_direct2/gemma/output/",
        files[j]
      ),
      paste0(
        "./ames_500_ld_0.1_MAF0.05_direct/gemma/output/",
        new_name
      )
    )
  }
}
QTN <- rbind(QTN, QTN2)
QTN <- QTN[order(QTN$rep),]
write.table(QTN, "./ames_500_ld_0.1_MAF0.05_direct/Additive_selected_QTNs.txt",
            quote = F, col.names = T, row.names = F, sep = "\t")
settings[[24]]$seed  <- 54605
out <- "./simulation/P/"
cat(paste("Setting", settings[[24]]$i, "\n"))
run_simulation_P(geno_file = settings[[24]]$data,
                 list = settings[[24]], out = out, h2 = h2)
path <-
  paste0(out, gsub("_mac5.*", "", settings[[24]]$data),
         "_MAF", settings[[24]]$maf, "/")
cat(paste("GEMMA: Setting", settings[[24]]$i, "\n"))
run_gemma(path = path, pop = settings[[24]]$data, n_cores = detectCores())
setwd("./simulation/")
x <- dir("./ames_500_ld_0.9_MAF0.4_direct/gemma/output")
reps <- names(
  table(gsub(".*Rep|_He.*","", x))[table(gsub(".*Rep|_He.*","", x))< 18]
  )
unlink(
  paste0("./ames_500_ld_0.9_MAF0.4_direct/gemma/output/",
         x[gsub(".*Rep|_He.*", "", x) %in% reps]),
  force = T,
  recursive = T
)
```

```r
x <- dir("./ames_500_ld_0.9_MAF0.4_direct/gemma/output")
total <- 1:100
missing <-
  total[!total %in% names(table(gsub(".*Rep|_He.*", "", x)))]
QTN <-
  read.table("./ames_500_ld_0.9_MAF0.4_direct/Additive_selected_QTNs.txt",
             h = T)
QTN <- QTN[!QTN$rep %in% missing,]
x2 <- dir("./ames_500_ld_0.9_MAF0.4_direct2/gemma/output/")
replace <-
  names(table(gsub(".*Rep|_He.*", "", x2))[
    table(gsub(".*Rep|_He.*", "", x2)) == 18])[1:length(missing)]
QTN2 <-
  read.table("./ames_500_ld_0.9_MAF0.4_direct2/Additive_selected_QTNs.txt",
             h = T)
QTN2 <- QTN2[QTN2$rep %in% replace, ]
QTN2$rep <- rep(missing, each = 2)
for(i in seq_along(replace)) {
  files <- x2[grepl(paste0("Rep", replace[i], "_"), x2)]
  for (j in 1:18) {
    new_name <-
      paste0(gsub("Rep.*", "", files[j]),
             "Rep",
             missing[i],
             "_H",
             gsub(".*_H", "", files[j]))
    file.rename(
      paste0(
        "./ames_500_ld_0.9_MAF0.4_direct2/gemma/output/",
        files[j]
      ),
      paste0(
        "./ames_500_ld_0.9_MAF0.4_direct/gemma/output/",
        new_name
      )
    )
  }
}
QTN <- rbind(QTN, QTN2)
QTN <- QTN[order(QTN$rep), ]
write.table(
  QTN,
  "./ames_500_ld_0.9_MAF0.4_direct/Additive_selected_QTNs.txt",
  quote = F,
  col.names = T,
  row.names = F,
  sep = "\t"
```

```r
)
# -------   correlation  ------------
setwd("./simulation/LD")
x <- dir()
correl <- tibble(
  specie = character(14400),
  h2 = character(14400),
  sample_size = character(14400),
  ld = character(14400),
  maf = character(14400),
  type = character(14400),
  cor = numeric(14400))
a <- 1
for (i in x){
 y <-  dir(paste0("./",i))[grepl("Simulated_Data", dir(paste0("./",i)))]
 for (ii in y){
   temp <- fread(paste0("./", i, "/", ii ), data.table = F)
   correl[a, "h2"] <- gsub(".*Herit_|.fam","", ii)
   correl[a, "cor"] <- cor(temp[,6], temp[,7])
   correl[a, "type"] <- gsub(".*_","", i)
   correl[a, "specie"] <- gsub("_.*","", i)
   correl[a, "sample_size"] <- gsub(".*_", "", gsub("_ld.*","", i))
   correl[a, "ld"] <- gsub(".*ld_|_MAF.*","", i)
   correl[a, "maf"] <- gsub(".*MAF|_.*","", i)
   a <- a + 1
   print(a)
 }
}
fwrite(correl, "correlation_LD.txt", sep = "\t", quote = F, row.names = F)

#------------ Pleiotropy --------------
setwd(home)
library(simplePHENOTYPES)
library(parallel)
library(data.table)
source("./scripts/run_simulation_P.R")
source("./scripts/run_gemma_p.R")
MAF <-  c(0.05, 0.4)
h2 <- matrix(c(0.3, 0.3, 0.8, 0.3, 0.8, 0.8), 3)
data <- c(
  "ames_500_mac5_ld09_numeric.txt",
  "ames_1000_mac5_ld09_numeric.txt",
  "ames_2815_mac5_ld09_numeric.txt",
  "soy_500_mac5_ld09_numeric.txt",
  "soy_1000_mac5_ld09_numeric.txt",
  "soy_2815_mac5_ld09_numeric.txt"
)
```

```r
#seed = round(runif(12, 0, 2000000))
seed <-
  c(
    1014956,
    613537,
    853815,
    1386204,
    170272,
    317557,
    549061,
    544610,
    1231659,
    859343,
    1303311,
    1135476
  )
grid <- expand.grid(maf = MAF,
                    data = data,
                    stringsAsFactors = F)
grid <- grid[order(grid$data, grid$maf), ]
settings <-  data.frame(i = 1:12, seed = seed, grid)
settings <- split(settings, settings$i)
out <- "./simulation/P/"
for (x in settings) {
  cat(paste("Setting", x$i, "\n"))
  run_simulation_P(
    geno_file = x$data,
    list = x,
    out = out,
    h2 = h2
  )
  path  <-
    paste0(out, gsub("_mac5.*", "", x$data), "_MAF", x$maf, "/")
  cat(paste("GEMMA: Setting", x$i, "\n"))
  run_gemma_p(path = path,
              pop = x$data,
              n_cores = detectCores())
}
# -------   correlation  ------------
library(tidyverse)
setwd("./simulation/P")
x <- dir()
correl <- tibble(
  specie = character(3600),
  h2 = character(3600),
  sample_size = character(3600),
  ld = character(3600),
```

```
  maf = character(3600),
  type = character(3600),
  cor = numeric(3600))
a <- 1
for (i in x){
  y <-  dir(paste0("./",i))[grepl("Simulated_Data", dir(paste0("./",i)))]
  for (ii in y){
    temp <- fread(paste0("./", i, "/", ii ), data.table = F)
    correl[a, "h2"] <- gsub(".*Herit_|.fam","", ii)
    correl[a, "cor"] <- cor(temp[,6], temp[,7])
    correl[a, "type"] <- "P"
    correl[a, "specie"] <- gsub("_.*","", i)
    correl[a, "sample_size"] <- gsub(".*_", "", gsub("_MAF.*","", i))
    correl[a, "maf"] <- gsub(".*MAF|_.*","", i)
    a <- a + 1
    print(a)
  }
}
fwrite(correl, "correlation_P.txt", sep = "\t", quote = F, row.names = F)
```

The scripts below were used to process the GWAS results and to count QTN detection in each of the scenarios.

```
#---------- Independent traits -----------
setwd(home)
library(data.table)
library(tidyverse)
library(SNPRelate)
path = "./simulation/ST/"
folder <- dir(path)[grep("ames_|soy_", dir(path))]
lfolder <- length(folder)
dist_threshold_ames <- 10000
dist_threshold_soy <- 1000000
count_total <- vector("list", lfolder)
total_33 <- tibble(
  specie = character(lfolder),
  sample_size = character(lfolder),
  maf = character(lfolder),
  h2 = "0.3_0.3",
  mt = numeric(lfolder),
  mtt1 = numeric(lfolder),
  mtt2 = numeric(lfolder),
  mtt1t2 = numeric(lfolder),
  t1 = numeric(lfolder),
  t2 = numeric(lfolder),
  t1_and_t2 = numeric(lfolder),
  af1 = numeric(lfolder),
  af2 = numeric(lfolder),
```

```
  chr1 = numeric(lfolder),
  chr2 = numeric(lfolder),
  ld_QTN = numeric(lfolder),
  dist_QTN = numeric(lfolder)
)
total_38 <- total_33
total_38$h2 <- "0.3_0.8"
total_88 <- total_33
total_88$h2 <- "0.8_0.8"
for (z in seq_along(folder)) {
  gemma_results <- dir(paste0(path, folder[z], "/gemma/output"))
  gemma_gwas_MT <-
    gemma_results[!grepl("log.txt", gemma_results) &
                    grepl("MTRep", gemma_results)]
  gemma_gwas_t1 <-
    gemma_results[!grepl("log.txt", gemma_results) &
                    grepl("T1_", gemma_results)]
  gemma_gwas_t2 <-
    gemma_results[!grepl("log.txt", gemma_results) &
                    grepl("T2_", gemma_results)]
  n_gwas <- length(gemma_gwas_MT)
  qtn <- fread(
    paste0(path, folder[z], "/Additive_selected_QTNs.txt"),
    header = TRUE,
    sep = "\t",
    data.table = F
  )
  if (gsub("_.*", "", folder[z]) == "soy") {
    qtn$chr <- as.numeric(gsub("Gm", "", qtn$chr))
  }
  d <- dir()[grepl(".gds", dir())]
  gds <- d[grepl(gsub("_MAF.*", "", folder[z]), d)]
  count <- tibble(
    specie = gsub("_.*", "", folder[z]),
    sample_size = gsub(".*_", "", gsub("_MAF.*", "", folder[z])),
    maf = gsub(".*MAF*", "", folder[z]),
    h2 = character(n_gwas),
    rep = numeric(n_gwas),
    mt = numeric(n_gwas),
    mtt1 = numeric(n_gwas),
    mtt2 = numeric(n_gwas),
    mtt1t2 = numeric(n_gwas),
    t1 = numeric(n_gwas),
    t2 = numeric(n_gwas),
    t1_and_t2 = numeric(n_gwas),
    af1 = numeric(n_gwas),
    af2 = numeric(n_gwas),
```

```
    chr1 = numeric(n_gwas),
    chr2 = numeric(n_gwas),
    ld_QTN = numeric(n_gwas),
    dist_QTN = numeric(n_gwas)
  )
  dist <-
    ifelse(gsub("_.*", "", folder[z]) == "ames",
           dist_threshold_ames,
           dist_threshold_soy)
  total_33$specie[z] <- gsub("_.*", "", folder[z])
  total_33$sample_size[z] <-
    gsub(".*_", "", gsub("_MAF.*", "", folder[z]))
  total_33$maf[z] <- gsub(".*MAF", "", folder[z])
  total_38$specie[z] <- total_33$specie[z]
  total_38$sample_size[z] <- total_33$sample_size[z]
  total_38$maf[z] <- total_33$maf[z]
  total_88$specie[z] <- total_33$specie[z]
  total_88$sample_size[z] <- total_33$sample_size[z]
  total_88$maf[z] <- total_33$maf[z]
  for (n in 1:n_gwas) {
    rep <- gsub("MTRep|_Herit.*", "", gemma_gwas_MT[n])
    h2 <- gsub(".*Herit_|.assoc.*", "", gemma_gwas_MT[n])
    gemma_mt <- fread(
      paste0(path, folder[z], "/gemma/output/", gemma_gwas_MT[n]),
      header = TRUE,
      sep = "\t",
      select = c("chr", "rs", "ps", "p_lrt"),
      data.table = F
    )
    gemma_mt$fdr <- p.adjust(gemma_mt$p_lrt , method = "BH")
    gemma_t1 <- fread(
      paste0(path, folder[z], "/gemma/output/", paste0("T1", gsub(
        "MT", "", gemma_gwas_MT[n]
      ))),
      header = TRUE,
      sep = "\t",
      select = c("chr", "rs", "ps", "p_lrt"),
      data.table = F
    )
    gemma_t1$fdr <- p.adjust(gemma_t1$p_lrt , method = "BH")
    gemma_t2 <- fread(
      paste0(path, folder[z], "/gemma/output/",  paste0("T2", gsub(
        "MT", "", gemma_gwas_MT[n]
      ))),
      header = TRUE,
      sep = "\t",
      select = c("chr", "rs", "ps", "p_lrt"),
```

```
    data.table = F
  )
  gemma_t2$fdr <- p.adjust(gemma_t2$p_lrt , method = "BH")
  genofile <- snpgdsOpen(gds)
  if (total_33$specie[z] == "ames") {
    snps <- read.gdsn(index.gdsn(genofile, "snp.rs.id"))
  } else {
    snps <- read.gdsn(index.gdsn(genofile, "snp.id"))
  }
  qtn_temp <- qtn[qtn$rep == rep,]
  if (h2 == "0.3_0.3") {
    qtn1 <- qtn_temp[1, ]
    qtn2 <- qtn_temp[2, ]
  } else if (h2 == "0.3_0.8") {
    qtn1 <- qtn_temp[3, ]
    qtn2 <- qtn_temp[4, ]
  } else {
    qtn1 <- qtn_temp[5, ]
    qtn2 <- qtn_temp[6, ]
  }
  qtn1_genotype <-
    read.gdsn(
      index.gdsn(genofile, "genotype"),
      start = c(1, which(snps %in% qtn1$snp)),
      count = c(-1, 1)
    )
  qtn2_genotype <-
    read.gdsn(
      index.gdsn(genofile, "genotype"),
      start = c(1, which(snps %in% qtn2$snp)),
      count = c(-1, 1)
    )
  count$ld_QTN[n] <-
    abs(SNPRelate::snpgdsLDpair(qtn1_genotype,
                                qtn2_genotype,
                                method = "composite"))
  snpgdsClose(genofile)
  gemma_mt <-
    gemma_mt %>%
    arrange(fdr) %>%
    filter(fdr < 0.1 &
             (chr == qtn1$chr |
                chr == qtn2$chr) & rs != qtn1$snp & rs != qtn2$snp) %>%
    mutate(
      dist_qtn1 = abs(ps - qtn1$pos),
      dist_qtn2 = abs(ps - qtn2$pos),
      rep = rep,
```

```r
      h2 = h2,
      model = "MT"
    )
  gemma_t1 <-
    gemma_t1 %>%
    arrange(fdr) %>%
    filter(fdr < 0.1 & chr == qtn1$chr & rs != qtn1$snp) %>%
    mutate(
      dist_qtn1 = abs(ps - qtn1$pos),
      dist_qtn2 = abs(ps - qtn2$pos),
      rep = rep,
      h2 = h2,
      model = "T1"
    )
  gemma_t2 <-
    gemma_t2 %>%
    arrange(fdr) %>%
    filter(fdr < 0.1 & chr == qtn2$chr & rs != qtn2$snp) %>%
    mutate(
      dist_qtn1 = abs(ps - qtn1$pos),
      dist_qtn2 = abs(ps - qtn2$pos),
      rep = rep,
      h2 = h2,
      model = "T2"
    )
  mt <- gemma_mt %>% filter(dist_qtn1 <= dist | dist_qtn2 <= dist)
  mtt1 <- gemma_mt %>% filter(dist_qtn1 <= dist)
  mtt2 <- gemma_mt %>% filter(dist_qtn2 <= dist)
  t1 <- gemma_t1 %>% filter(dist_qtn1 <= dist)
  t2 <- gemma_t2 %>% filter(dist_qtn2 <= dist)
  count$mt[n] <- as.numeric(nrow(mt) > 0)
  count$mtt1[n] <- as.numeric(nrow(mtt1) > 0)
  count$mtt2[n] <- as.numeric(nrow(mtt2) > 0)
  count$mtt1t2[n] <-
    as.numeric(nrow(mtt1) > 0 & nrow(mtt2) > 0)
  count$t1[n] <- as.numeric(nrow(t1) > 0)
  count$t2[n] <- as.numeric(nrow(t2) > 0)
  count$t1_and_t2[n] <- as.numeric(nrow(t1) > 0 & nrow(t2) > 0)
  count$h2[n] <- h2
  count$rep[n] <- rep
  count$af1[n] <- qtn1$maf
  count$af2[n] <- qtn2$maf
  count$chr1[n] <- qtn1$chr
  count$chr2[n] <- qtn2$chr
  count$dist_QTN[n] <- abs(qtn1$pos - qtn2$pos)
  print(n)
}
```

```
  print(folder[z])
  count_h33 <-  count %>% filter(h2 == "0.3_0.3")
  count_h38 <-  count %>% filter(h2 == "0.3_0.8")
  count_h88 <-  count %>% filter(h2 == "0.8_0.8")
  total_33$mt[z] <- count_h33 %>% select(mt) %>% sum
  total_33$mtt1[z] <- count_h33 %>% select(mtt1) %>% sum
  total_33$mtt2[z] <- count_h33 %>% select(mtt2) %>% sum
  total_33$mtt1t2[z] <- count_h33 %>% select(mtt1t2) %>% sum
  total_33$t1[z] <- count_h33 %>% select(t1) %>% sum
  total_33$t2[z] <- count_h33 %>% select(t2) %>% sum
  total_33$t1_and_t2[z] <- count_h33 %>% select(t1_and_t2) %>% sum
  total_33$af1[z] <-  count_h33 %>% summarise(af1 = median(af1))
  total_33$af2[z] <- count_h33 %>% summarise(af1 = median(af2))
  total_33$ld_QTN[z] <-
    count_h33 %>% summarise(ld_QTN = median(ld_QTN))
  total_33$dist_QTN[z] <-
    count_h33 %>% summarise(dist_QTN = median(dist_QTN))
  total_38$mt[z] <- count_h38 %>% select(mt) %>% sum
  total_38$mtt1[z] <- count_h38 %>% select(mtt1) %>% sum
  total_38$mtt2[z] <- count_h38 %>% select(mtt2) %>% sum
  total_38$mtt1t2[z] <- count_h38 %>% select(mtt1t2) %>% sum
  total_38$t1[z] <- count_h38 %>% select(t1) %>% sum
  total_38$t2[z] <- count_h38 %>% select(t2) %>% sum
  total_38$t1_and_t2[z] <- count_h38 %>% select(t1_and_t2) %>% sum
  total_38$af1[z] <-  count_h38 %>% summarise(af1 = median(af1))
  total_38$af2[z] <- count_h38 %>% summarise(af1 = median(af2))
  total_38$ld_QTN[z] <-
    count_h38 %>% summarise(ld_QTN = median(ld_QTN))
  total_38$dist_QTN[z] <-
    count_h38 %>% summarise(dist_QTN = median(dist_QTN))
  total_88$mt[z] <- count_h88 %>% select(mt) %>% sum
  total_88$mtt1[z] <- count_h88 %>% select(mtt1) %>% sum
  total_88$mtt2[z] <- count_h88 %>% select(mtt2) %>% sum
  total_88$mtt1t2[z] <- count_h88 %>% select(mtt1t2) %>% sum
  total_88$t1[z] <- count_h88 %>% select(t1) %>% sum
  total_88$t2[z] <- count_h88 %>% select(t2) %>% sum
  total_88$t1_and_t2[z] <- count_h88 %>% select(t1_and_t2) %>% sum
  total_88$af1[z] <-  count_h88 %>% summarise(af1 = median(af1))
  total_88$af2[z] <- count_h88 %>% summarise(af1 = median(af2))
  total_88$ld_QTN[z] <-
    count_h88 %>% summarise(ld_QTN = median(ld_QTN))
  total_88$dist_QTN[z] <-
    count_h88 %>% summarise(dist_QTN = median(dist_QTN))
  count_total[[z]] <- count
}
count_total <- rbindlist(count_total)
total <- bind_rows(total_33, total_38, total_88)
```

```r
fwrite(count_total, "total_count_all_ST.txt", sep = "\t", quote = F, row.names = F)
fwrite(total, "total_count_ST.txt", sep = "\t", quote = F, row.names = F)

#---------- Spurious Pleiotropy -----------
setwd(home)
path = "./simulation/LD/"
folder <- dir(path)[grep("ames_|soy_", dir(path))]
lfolder <- length(folder)
dist_threshold_ames <- 10000
dist_threshold_soy <- 1000000
count_total <- vector("list", lfolder)
total_33 <- tibble(
  specie = character(lfolder),
  sample_size = character(lfolder),
  ld = character(lfolder),
  maf = character(lfolder),
  eff = character(lfolder),
  type = character(lfolder),
  h2 = "0.3_0.3",
  mt = numeric(lfolder),
  mtt1 = numeric(lfolder),
  mtt2 = numeric(lfolder),
  mtt1t2 = numeric(lfolder),
  t1 = numeric(lfolder),
  t2 = numeric(lfolder),
  t1_and_t2 = numeric(lfolder),
  af1 = numeric(lfolder),
  af2 = numeric(lfolder),
  ld_QTN = numeric(lfolder),
  dist_QTN = numeric(lfolder))
total_38 <- total_33
total_38$h2 <- "0.3_0.8"
total_88 <- total_33
total_88$h2 <- "0.8_0.8"
for (z in seq_along(folder)) {
  gemma_results <- dir(paste0(path, folder[z], "/gemma/output"))
  gemma_gwas_MT <-
    gemma_results[!grepl("log.txt", gemma_results) &
                    grepl("MTRep", gemma_results)]
  gemma_gwas_t1 <-
    gemma_results[!grepl("log.txt", gemma_results) &
                    grepl("T1_", gemma_results)]
  gemma_gwas_t2 <-
    gemma_results[!grepl("log.txt", gemma_results) &
                    grepl("T2_", gemma_results)]
  n_gwas <- length(gemma_gwas_MT)
  qtn <- fread(
```

```r
    paste0(path, folder[z], "/Additive_selected_QTNs.txt"),
    header = TRUE,
    sep = "\t",
    data.table = F
  )
  ld_between_qtn <- fread(
    paste0(path, folder[z], "/LD_summary_Additive.txt"),
    header = TRUE,
    sep = "\t",
    data.table = F
  )
  d <- dir()[grepl(".gds", dir())]
  gds <- d[grepl(gsub("_ld.*", "", folder[z]), d)]
  data <- vector("list", n_gwas)
  count <- tibble(
    specie = gsub("_.*", "", folder[z]),
    sample_size = gsub(".*_", "", gsub("_ld.*", "", folder[z])),
    ld = gsub(".*ld_|_MAF.*", "", folder[z]),
    maf = gsub(".*MAF|_eff.*", "", folder[z]),
    eff = gsub(".*_eff_|_dir.*|_indir.*", "", folder[z]),
    type = gsub(".*_", "", folder[z]),
    h2 = character(n_gwas),
    rep = numeric(n_gwas),
    mt = numeric(n_gwas),
    mtt1 = numeric(n_gwas),
    mtt2 = numeric(n_gwas),
    mtt1t2 = numeric(n_gwas),
    t1 = numeric(n_gwas),
    t2 = numeric(n_gwas),
    t1_and_t2 = numeric(n_gwas),
    af1 = numeric(n_gwas),
    af2 = numeric(n_gwas),
    ld_QTN = numeric(n_gwas),
    dist_QTN = numeric(n_gwas)
  )
  indirect <- gsub(".*_", "", folder[z]) == "indirect"
  dist <-
    ifelse(gsub("_.*", "", folder[z]) == "ames",
           dist_threshold_ames,
           dist_threshold_soy)
  total_33$specie[z] <- gsub("_.*", "", folder[z])
  total_33$sample_size[z] <-
    gsub(".*_", "", gsub("_ld.*", "", folder[z]))
  total_33$ld[z] <- gsub(".*ld_|_MAF.*", "", folder[z])
  total_33$maf[z] <- gsub(".*MAF|_eff.*", "", folder[z])
  total_33$eff[z] <- gsub(".*_eff_|_dir.*|_indir.*", "", folder[z])
  total_33$type[z] <- gsub(".*_", "", folder[z])
```

```r
  total_38$specie[z] <- total_33$specie[z]
  total_38$sample_size[z] <- total_33$sample_size[z]
  total_38$ld[z] <- total_33$ld[z]
  total_38$maf[z] <- total_33$maf[z]
  total_38$eff[z] <- total_33$eff[z]
  total_38$type[z] <- total_33$type[z]
  total_88$specie[z] <- total_33$specie[z]
  total_88$sample_size[z] <- total_33$sample_size[z]
  total_88$ld[z] <- total_33$ld[z]
  total_88$maf[z] <- total_33$maf[z]
  total_88$eff[z] <- total_33$eff[z]
  total_88$type[z] <- total_33$type[z]
  for (n in 1:n_gwas) {
    rep <- gsub("MTRep|_Herit.*", "", gemma_gwas_MT[n])
    h2 <- gsub(".*Herit_|.assoc.*", "", gemma_gwas_MT[n])
    gemma_mt <- fread(
      paste0(path, folder[z], "/gemma/output/", gemma_gwas_MT[n]),
      header = TRUE,
      sep = "\t",
      select = c("chr", "rs", "ps", "p_lrt"),
      data.table = F
    )
    gemma_mt$fdr <- p.adjust(gemma_mt$p_lrt , method = "BH")
    gemma_t1 <- fread(
      paste0(path, folder[z], "/gemma/output/", paste0("T1_", gsub(
        "MT", "", gemma_gwas_MT[n]
      ))),
      header = TRUE,
      sep = "\t",
      select = c("chr", "rs", "ps", "p_lrt"),
      data.table = F
    )
    gemma_t1$fdr <- p.adjust(gemma_t1$p_lrt , method = "BH")
    gemma_t2 <- fread(
      paste0(path, folder[z], "/gemma/output/",  paste0("T2_", gsub(
        "MT", "", gemma_gwas_MT[n]
      ))),
      header = TRUE,
      sep = "\t",
      select = c("chr", "rs", "ps", "p_lrt"),
      data.table = F
    )
    gemma_t2$fdr <- p.adjust(gemma_t2$p_lrt , method = "BH")
    genofile <- snpgdsOpen(gds)
    if (total_33$specie[z] == "ames") {
      snps <- read.gdsn(index.gdsn(genofile, "snp.rs.id"))
    } else {
```

```
    snps <- read.gdsn(index.gdsn(genofile, "snp.id"))
  }
  qtn1 <-
    qtn[qtn$rep == rep &
          (qtn$snp_type == "QTN_for_trait_1" |
             qtn$snp_type == "QTN_upstream"),]
  qtn1_genotype <-
    read.gdsn(
      index.gdsn(genofile, "genotype"),
      start = c(1, which(snps %in% qtn1$snp)),
      count = c(-1, 1)
    )
  qtn2 <-
    qtn[qtn$rep == rep &
          (qtn$snp_type == "QTN_for_trait_2" |
             qtn$snp_type == "QTN_downstream"),]
  qtn2_genotype <-
    read.gdsn(
      index.gdsn(genofile, "genotype"),
      start = c(1, which(snps %in% qtn2$snp)),
      count = c(-1, 1)
    )
  snpgdsClose(genofile)
  gemma_mt <-
    gemma_mt %>%
    arrange(fdr) %>%
    filter(fdr < 0.1 & chr == qtn1$chr) %>%
    mutate(
      dist_qtn1 = abs(ps - qtn1$pos),
      dist_qtn2 = abs(ps - qtn2$pos),
      rep = rep,
      h2 = h2,
      model = "MT"
    )
  gemma_t1 <-
    gemma_t1 %>%
    arrange(fdr) %>%
    filter(fdr < 0.1 & chr == qtn1$chr) %>%
    mutate(
      dist_qtn1 = abs(ps - qtn1$pos),
      dist_qtn2 = abs(ps - qtn2$pos),
      rep = rep,
      h2 = h2,
      model = "T1"
    )
  gemma_t2 <-
    gemma_t2 %>%
```

```
    arrange(fdr) %>%
    filter(fdr < 0.1 & chr == qtn2$chr) %>%
    mutate(
      dist_qtn1 = abs(ps - qtn1$pos),
      dist_qtn2 = abs(ps - qtn2$pos),
      rep = rep,
      h2 = h2,
      model = "T2"
    )
  mt <- gemma_mt %>% filter(dist_qtn1 <= dist | dist_qtn2 <= dist)
  mtt1 <- gemma_mt %>% filter(dist_qtn1 <= dist)
  mtt2 <- gemma_mt %>% filter(dist_qtn2 <= dist)
  t1 <- gemma_t1 %>% filter(dist_qtn1 <= dist)
  t2 <- gemma_t2 %>% filter(dist_qtn2 <= dist)
  count$mt[n] <- as.numeric(nrow(mt) > 0)
  count$mtt1[n] <- as.numeric(nrow(mtt1) > 0)
  count$mtt2[n] <- as.numeric(nrow(mtt2) > 0)
  count$mtt1t2[n] <-
    as.numeric(nrow(mtt1) > 0 & nrow(mtt2) > 0)
  count$t1[n] <- as.numeric(nrow(t1) > 0)
  count$t2[n] <- as.numeric(nrow(t2) > 0)
  count$t1_and_t2[n] <- as.numeric(nrow(t1) > 0 & nrow(t2) > 0)
  count$h2[n] <- h2
  count$rep[n] <- rep
  count$af1[n] <- qtn1$maf
  count$af2[n] <- qtn2$maf
  count$ld_QTN[n] <- ifelse(ncol(ld_between_qtn) == 5,
                            ld_between_qtn[ld_between_qtn$rep %in% rep, 3],
                            ld_between_qtn[ld_between_qtn$rep %in% rep, 8])
  count$dist_QTN[n] <- abs(qtn1$pos - qtn2$pos)
  print(n)
  gdsfmt::showfile.gds(closeall = TRUE, verbose = F)
}
print(folder[z])
count_h33 <-  count %>% filter(h2 == "0.3_0.3")
count_h38 <-  count %>% filter(h2 == "0.3_0.8")
count_h88 <-  count %>% filter(h2 == "0.8_0.8")
total_33$mt[z] <- count_h33 %>% select(mt) %>% sum
total_33$mtt1[z] <- count_h33 %>% select(mtt1) %>% sum
total_33$mtt2[z] <- count_h33 %>% select(mtt2) %>% sum
total_33$mtt1t2[z] <- count_h33 %>% select(mtt1t2) %>% sum
total_33$t1[z] <- count_h33 %>% select(t1) %>% sum
total_33$t2[z] <- count_h33 %>% select(t2) %>% sum
total_33$t1_and_t2[z] <- count_h33 %>% select(t1_and_t2) %>% sum
total_33$af1[z] <-  count_h33 %>% summarise(af1 = median(af1))
total_33$af2[z] <- count_h33 %>% summarise(af1 = median(af2))
total_33$ld_QTN[z] <-
```

```
    count_h33 %>% summarise(ld_QTN = median(ld_QTN))
  total_33$dist_QTN[z] <-
    count_h33 %>% summarise(dist_QTN = median(dist_QTN))
  total_38$mt[z] <- count_h38 %>% select(mt) %>% sum
  total_38$mtt1[z] <- count_h38 %>% select(mtt1) %>% sum
  total_38$mtt2[z] <- count_h38 %>% select(mtt2) %>% sum
  total_38$mtt1t2[z] <- count_h38 %>% select(mtt1t2) %>% sum
  total_38$t1[z] <- count_h38 %>% select(t1) %>% sum
  total_38$t2[z] <- count_h38 %>% select(t2) %>% sum
  total_38$t1_and_t2[z] <- count_h38 %>% select(t1_and_t2) %>% sum
  total_38$af1[z] <-  count_h38 %>% summarise(af1 = median(af1))
  total_38$af2[z] <- count_h38 %>% summarise(af1 = median(af2))
  total_38$ld_QTN[z] <-
    count_h38 %>% summarise(ld_QTN = median(ld_QTN))
  total_38$dist_QTN[z] <-
    count_h38 %>% summarise(dist_QTN = median(dist_QTN))
  total_88$mt[z] <- count_h88 %>% select(mt) %>% sum
  total_88$mtt1[z] <- count_h88 %>% select(mtt1) %>% sum
  total_88$mtt2[z] <- count_h88 %>% select(mtt2) %>% sum
  total_88$mtt1t2[z] <- count_h88 %>% select(mtt1t2) %>% sum
  total_88$t1[z] <- count_h88 %>% select(t1) %>% sum
  total_88$t2[z] <- count_h88 %>% select(t2) %>% sum
  total_88$t1_and_t2[z] <- count_h88 %>% select(t1_and_t2) %>% sum
  total_88$af1[z] <-  count_h88 %>% summarise(af1 = median(af1))
  total_88$af2[z] <- count_h88 %>% summarise(af1 = median(af2))
  total_88$ld_QTN[z] <-
    count_h88 %>% summarise(ld_QTN = median(ld_QTN))
  total_88$dist_QTN[z] <-
    count_h88 %>% summarise(dist_QTN = median(dist_QTN))
  count_total[[z]] <- count
}
count_total <- rbindlist(count_total)
total <- bind_rows(total_33, total_38, total_88)
fwrite(count_total, "total_count_all.txt", sep = "\t", quote = F, row.names = F)
fwrite(total, "total_count.txt", sep = "\t", quote = F, row.names = F)

#---------- Pleiotropy -----------
setwd(home)
path = "./simulation/pleio/"
folder <- dir(path)[grep("ames_|soy_", dir(path))]
lfolder <- length(folder)
dist_threshold_ames <- 10000
dist_threshold_soy <- 1000000
count_total <- vector("list", lfolder)
total_33 <- tibble(
  specie = character(lfolder),
  sample_size = character(lfolder),
```

```r
  maf = character(lfolder),
  h2 = "0.3_0.3",
  mt = numeric(lfolder),
  t1 = numeric(lfolder),
  t2 = numeric(lfolder),
  t1_and_t2 = numeric(lfolder),
  af1 = numeric(lfolder))
total_38 <- total_33
total_38$h2 <- "0.3_0.8"
total_88 <- total_33
total_88$h2 <- "0.8_0.8"
for (z in seq_along(folder)) {
  gemma_results <- dir(paste0(path, folder[z], "/gemma/output"))
  gemma_gwas_MT <-
    gemma_results[!grepl("log.txt", gemma_results) &
                    grepl("MTRep", gemma_results)]
  gemma_gwas_t1 <-
    gemma_results[!grepl("log.txt", gemma_results) &
                    grepl("T1_", gemma_results)]
  gemma_gwas_t2 <-
    gemma_results[!grepl("log.txt", gemma_results) &
                    grepl("T2_", gemma_results)]
  n_gwas <- length(gemma_gwas_MT)
  qtn <- fread(
    paste0(path, folder[z], "/Additive_selected_QTNs.txt"),
    header = TRUE,
    sep = "\t",
    data.table = F
  )
  d <- dir()[grepl(".gds", dir())]
  gds <- d[grepl(gsub("_MAF.*", "", folder[z]), d)]
  count <- tibble(
    specie = gsub("_.*", "", folder[z]),
    sample_size = gsub(".*_", "", gsub("_MAF.*", "", folder[z])),
    maf = gsub(".*MAF", "", folder[z]),
    h2 = character(n_gwas),
    rep = numeric(n_gwas),
    mt = numeric(n_gwas),
    t1 = numeric(n_gwas),
    t2 = numeric(n_gwas),
    t1_and_t2 = numeric(n_gwas),
    af1 = numeric(n_gwas)
  )
  dist <-
    ifelse(gsub("_.*", "", folder[z]) == "ames",
           dist_threshold_ames,
           dist_threshold_soy)
```

```
total_33$specie[z] <- gsub("_.*", "", folder[z])
total_33$sample_size[z] <-
  gsub(".*_", "", gsub("_MAF.*", "", folder[z]))
total_33$maf[z] <- gsub(".*MAF", "", folder[z])
total_38$specie[z] <- total_33$specie[z]
total_38$sample_size[z] <- total_33$sample_size[z]
total_38$maf[z] <- total_33$maf[z]
total_88$specie[z] <- total_33$specie[z]
total_88$sample_size[z] <- total_33$sample_size[z]
total_88$maf[z] <- total_33$maf[z]
for (n in 1:n_gwas) {
  rep <- gsub("MTRep|_Herit.*", "", gemma_gwas_MT[n])
  h2 <- gsub(".*Herit_|.assoc.*", "", gemma_gwas_MT[n])
  gemma_mt <- fread(
    paste0(path, folder[z], "/gemma/output/", gemma_gwas_MT[n]),
    header = TRUE,
    sep = "\t",
    select = c("chr", "rs", "ps", "p_lrt"),
    data.table = F
  )
  gemma_mt$fdr <- p.adjust(gemma_mt$p_lrt , method = "BH")
  gemma_t1 <- fread(
    paste0(path, folder[z], "/gemma/output/", paste0("T1_", gsub(
      "MT", "", gemma_gwas_MT[n]
    ))),
    header = TRUE,
    sep = "\t",
    select = c("chr", "rs", "ps", "p_lrt"),
    data.table = F
  )
  gemma_t1$fdr <- p.adjust(gemma_t1$p_lrt , method = "BH")
  gemma_t2 <- fread(
    paste0(path, folder[z], "/gemma/output/",  paste0("T2_", gsub(
      "MT", "", gemma_gwas_MT[n]
    ))),
    header = TRUE,
    sep = "\t",
    select = c("chr", "rs", "ps", "p_lrt"),
    data.table = F
  )
  gemma_t2$fdr <- p.adjust(gemma_t2$p_lrt , method = "BH")
  genofile <- snpgdsOpen(gds)
  if (total_33$specie[z] == "ames") {
    snps <- read.gdsn(index.gdsn(genofile, "snp.rs.id"))
  } else {
    snps <- read.gdsn(index.gdsn(genofile, "snp.id"))
  }
```

```r
    qtn1 <- qtn[qtn$rep == rep,]
    qtn1_genotype <-
      read.gdsn(
        index.gdsn(genofile, "genotype"),
        start = c(1, which(snps %in% qtn1$snp)),
        count = c(-1, 1)
      )
    snpgdsClose(genofile)
    gemma_mt <-
      gemma_mt %>%
      arrange(fdr) %>%
      filter(fdr < 0.1 & chr == qtn1$chr) %>%
      mutate(
        dist_qtn1 = abs(ps - qtn1$pos),
        rep = rep,
        h2 = h2,
        model = "MT"
      )
    gemma_t1 <-
      gemma_t1 %>%
      arrange(fdr) %>%
      filter(fdr < 0.1 & chr == qtn1$chr) %>%
      mutate(
        dist_qtn1 = abs(ps - qtn1$pos),
        rep = rep,
        h2 = h2,
        model = "T1"
      )
    gemma_t2 <-
      gemma_t2 %>%
      arrange(fdr) %>%
      filter(fdr < 0.1 & chr == qtn1$chr) %>%
      mutate(
        dist_qtn1 = abs(ps - qtn1$pos),
        rep = rep,
        h2 = h2,
        model = "T2"
      )
    mt <- gemma_mt %>% filter(dist_qtn1 <= dist)
    t1 <- gemma_t1 %>% filter(dist_qtn1 <= dist)
    t2 <- gemma_t2 %>% filter(dist_qtn1 <= dist)
    count$mt[n] <- as.numeric(nrow(mt) > 0)
    count$t1[n] <- as.numeric(nrow(t1) > 0)
    count$t2[n] <- as.numeric(nrow(t2) > 0)
    count$t1_and_t2[n] <- as.numeric(nrow(t1) > 0 & nrow(t2) > 0)
    count$h2[n] <- h2
    count$rep[n] <- rep
```

```r
    count$af1[n] <- qtn1$maf
    print(n)
  }
  print(folder[z])
  count_h33 <-  count %>% filter(h2 == "0.3_0.3")
  count_h38 <-  count %>% filter(h2 == "0.3_0.8")
  count_h88 <-  count %>% filter(h2 == "0.8_0.8")
  total_33$mt[z] <- count_h33 %>% select(mt) %>% sum
  total_33$t1[z] <- count_h33 %>% select(t1) %>% sum
  total_33$t2[z] <- count_h33 %>% select(t2) %>% sum
  total_33$t1_and_t2[z] <- count_h33 %>% select(t1_and_t2) %>% sum
  total_33$af1[z] <-  count_h33 %>% summarise(af1 = median(af1))
  total_38$mt[z] <- count_h38 %>% select(mt) %>% sum
  total_38$t1[z] <- count_h38 %>% select(t1) %>% sum
  total_38$t2[z] <- count_h38 %>% select(t2) %>% sum
  total_38$t1_and_t2[z] <- count_h38 %>% select(t1_and_t2) %>% sum
  total_38$af1[z] <-  count_h38 %>% summarise(af1 = median(af1))
  total_88$mt[z] <- count_h88 %>% select(mt) %>% sum
  total_88$t1[z] <- count_h88 %>% select(t1) %>% sum
  total_88$t2[z] <- count_h88 %>% select(t2) %>% sum
  total_88$t1_and_t2[z] <- count_h88 %>% select(t1_and_t2) %>% sum
  total_88$af1[z] <-  count_h88 %>% summarise(af1 = median(af1))
  count_total[[z]] <- count
}
count_total <- rbindlist(count_total)
total <- bind_rows(total_33, total_38, total_88)
fwrite(count_total, "total_count_all_p.txt", sep = "\t", quote = F, row.names = F)
fwrite(total, "total_count_p.txt", sep = "\t", quote = F, row.names = F)
```
